# Intramolecular Carboxyamidation of Alkyne-Tethered *O*-Acylhydroxamates through Formation of Fe(III)-Nitrenoids

**DOI:** 10.1002/chem.202303428

**Published:** 2023-12-11

**Authors:** Siyuan Su, Yu Zhang, Peng Liu, Donald J. Wink, Daesung Lee

**Affiliations:** a Department of Chemistry, University of Illinois Chicago, 845 W. Taylor St., Chicago, Illinois 60607 (USA); b Department of Chemistry, University of Pittsburgh, 219 Parkman Avenue, Pittsburgh, PA 15260 (USA)

**Keywords:** acylhydroxamate, acyl migration, carboxyamidation, iron catalysis, nitrenoid

## Abstract

We developed intramolecular carboxyamidations of alkyne-tethered *O*-acylhydroxamates followed by either thermally induced spontaneous or 4-(dimethylamino)pyridine-catalyzed O⟶O or O⟶N acyl group migration. Under iron-catalyzed conditions, the carboxyamidation products were generated in high yield from both *Z*-alkene and arene-tethered substrates. DFT calculations indicate that the iron-catalyzed carboxyamidation proceeds via a stepwise mechanism involving iron-imidyl radical cyclization followed by intramolecular acyloxy transfer from the iron center to the alkenyl radical center to furnish the *cis*-carboxyamidation product. Upon treatment with 4-(dimethylamino)pyridine, the *Z*-alkene-tethered carboxyamidation products underwent selective O⟶O acyl migration to generate 2-acyloxy-5-acyl pyrroles. Thermal O⟶N acyl migration occurs during carboxyamidation if the *Z*-alkene linker contains an alkyl or an aryl substituent at the β-position of the carbonyl group. On the other hand, the arene linker-containing compounds selectively undergo O⟶N acyl migration to generate *N*-acyl-3-acylisoindolinones, and the corresponding O⟶O acyl migration forming isoindole derivatives was not observed.

## Introduction

**Scheme 1.**
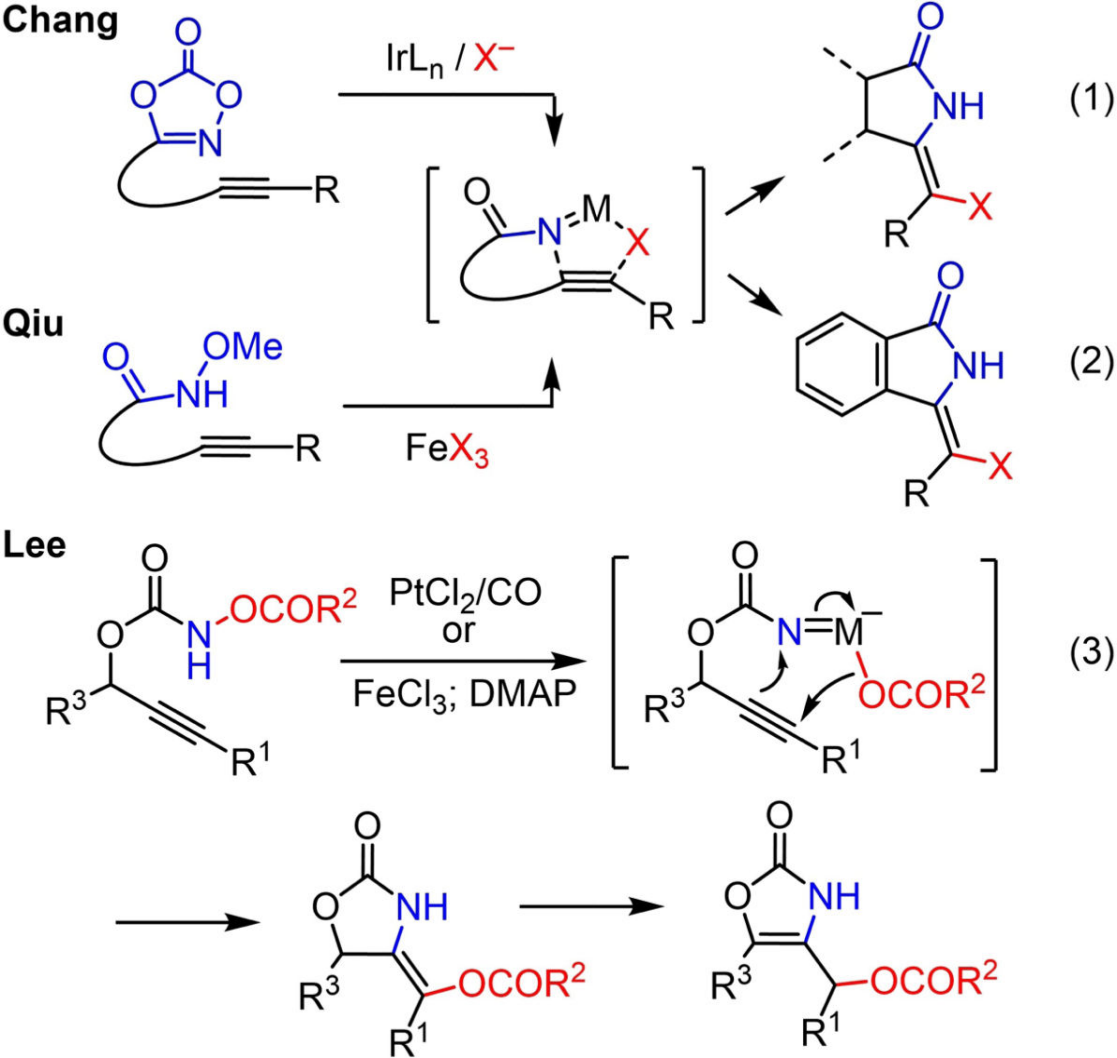
Representative difunctionalization of alkynes mediated by nitrenoid.

Oxyamination reactions of π-bonds are powerful tools to generate *N,O*-containing molecular structures from simple feed-stock materials.^[1]^ A variety of transition metal complexes including osmium,^[2]^ iron,^[3]^ copper,^[4]^ gold,^[5]^ platinum,^[6]^ palladium,^[7]^ rhodium,^[8]^ ruthenium,^[9]^ manganese^[10]^ and iridium^[11]^ have been used as a catalyst to promote the alkene oxyamination reactions. Notable recent alkene intramolecular oxyamination reactions are reported by Xu,^[3c,d]^ Chang,^[11a,c]^ and Rovis^[11b,d]^ which employed hydroxamic acid derivatives as the source of nitrogen and oxygen functionality. Compared to the oxyamination of alkenes, however, the corresponding oxyamination of alkynes are underdeveloped.^[12–15]^ Pioneering studies on intramolecular alkyne haloamination^[16,17]^ and oxyamination^[15]^ relying on metal-nitrenoid have been reported recently (Scheme 1).^[18]^ Chang and coworkers reported on the *Z*-selective haloamination reaction employing a dioxazolone functionality as a source of the acyl Ir-nitrenoid (Eq. (1)).^[16]^ Selective formation of *Z*-isomer was observed. To justify the stereochemical outcome, a novel ligand-participated nitrenoid-alkyne [3 + 2] cycloaddition mechanism is proposed. Qiu employed FeX_3_ as the catalyst to promote haloamination of alkyne-tethered *N*-methoxyamide (Eq. (2)).^[17]^ This reaction also proceeds through formation of nitrenoid as a crucial intermediate, which transfers nitrogen and halogen onto the alkyne to generate the product with *Z*-selectivity. We explored the platinum- and iron-catalyzed intramolecular carboxyamidation of alkyne-tethered *N*-acyloxycarbamates to form 2-(3*H*)oxazolones as the final products, which is the consequence of intramolecular alkyne carboxyamidation followed by isomerization of an exocyclic alkene to the corresponding endocyclic alkene (Eq. (3)).^[15]^

**Scheme 2.**
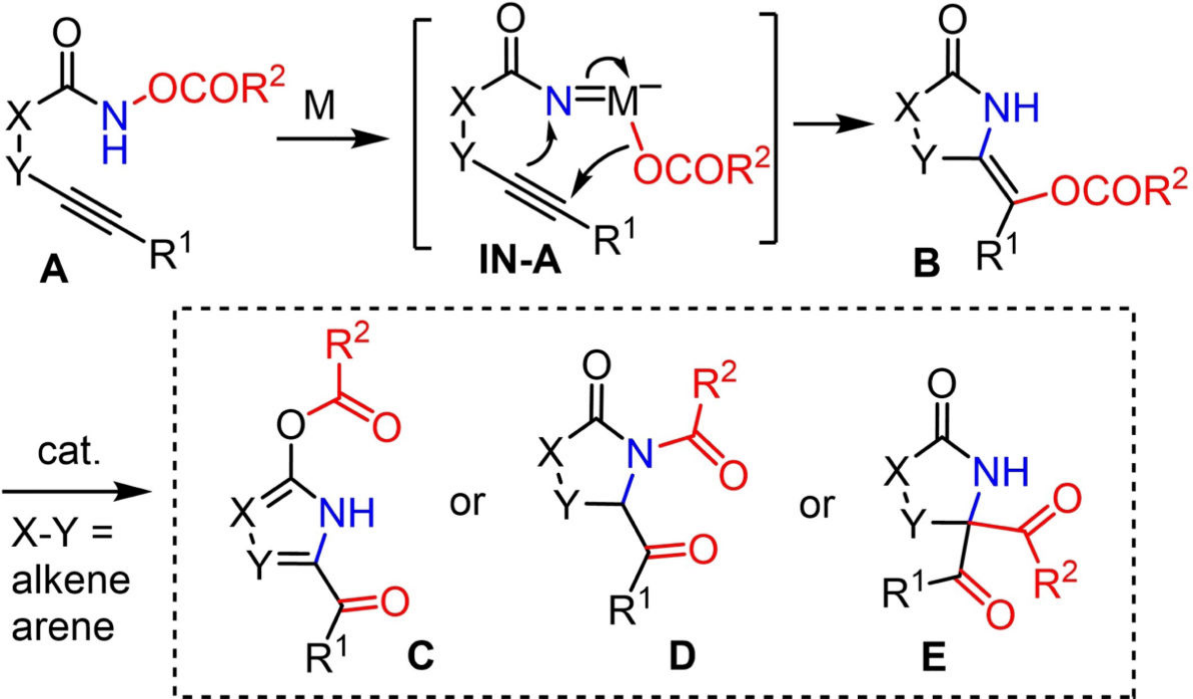
Alkyne carboxyamidation followed by acyl group migration.

Inspired by the novel carboxyamidation of alkyne-tethered *N*-acyloxycarbamates followed by alkene isomerization, we decided to extend our exploration to alkyne-tethered *O*-acylhydroxamates (Scheme 2). We expected that with suitable catalysts and conditions, acyl group migration instead of alkene migration will take place to generate different end products depending on the nature of the linker between the carbonyl group and the alkyne moiety. We anticipated *O*-acylhydroxamates **A** would interact with a catalyst and generate acyloxy group-ligated nitrenoid (**IN-A**). An intramolecular transfer of the ligated carboxylate through either a concerted formal [3 + 2] mechanism or a stepwise process will deliver carboxyamidation product **B**. Furthermore, due to the structural characteristics of the acyloxy-substituted enamide moiety, we predict that compound **B** would undergo migration of the acyl group to generate final product **C**, **D**, or **E** depending on the linker X—Y.^[19a]^ For example, if X—Y is a *Z*-alkene, the acyl group would migrate to the oxygen of the amide (O⟶O) so that substituted pyrrole derivative **C** would be generated. On the other hand, if X—Y is an arene linker the acyl group will be migrated to the nitrogen of the amide (O⟶N) to generate *N*-acylisoindolinones **D**. Also, alternative migration leading to product **E** (O⟶C) is plausible although less likely because of the sterically congested environment at the reaction center.^[19b–l]^

A unique feature of this carboxyamidation compared to the reported haloamidation-based difunctionalization is that the carboxylate extruded from the substrate will transfer onto the alkyne; thus, the external supply of nucleophiles is not required. Also, the enol carboxylate moiety in **B** would have a high tendency to transfer the acyl group to liberate the corresponding ketone; thus, divergent migration can be promoted under suitable conditions to generate various *N,O*-functionalized heterocyclic compounds.

## Results and Discussion

We commenced our exploration of carboxyamidation of *O*-acylhydroxamates by defining an optimal catalyst and reaction conditions. We employed disubstituted *Z*-alkenyl linked substrate **1 a** and examined its reactivity with platinum and iron complexes (Table 1). Although PtCl_2_/CO was effective for the carboxyamidation of alkyne-tethered *N*-acyloxycarbamate, only decomposition was observed for **1 a** with this catalyst (entry 1).^[13]^ On the other hand, FeBr_2_ and FeCl_3_ were effective for carboxyamidation of **1 a** to generate product **2 a** in excellent yield, but haloamidation products **2 a**’ and **2 a̋** were also produced as minor components (entries 2 and 3). Reaction with an excess amount of Bu_4_NBr (3 equiv.) as an external halide source provided a mixture of the carboxylate (**2 a**) and bromide-incorporated product (**2 a**’) in a 1 : 2.7 ratio (entry 4). However, the same reaction with Bu_4_NCl (3 equiv.) provided only a chloride-incorporated product **2 a̋** (entry 5). This demonstrates that displacement of the carboxylate on the nitrenoid intermediate is more efficient with chloride than bromide. To minimize the halide transfer from the catalyst FeX_3_ to the product, we employed Fe(acac)_3_. We expected that with the bidentate ligation and stronger acceptor capability of the acetylacetonate ligand the reaction should be more efficient with no ligand transfer. Indeed, the reaction of **1 a** with Fe(acac)_3_ provided **2 a** in 85 % at a lower temperature (70 °C) without acetylacetonate-transferred by-product (entry 6). The experiment without an iron catalyst led to only decomposed materials (entry 7).

With the optimal catalyst Fe(acac)_3_ and conditions (CH_3_CN, 70 °C) in hand, we examined other substrates with variations of substituent on the alkenyl tether or on the alkyne (Table 2). Replacing the phenyl group in **1 a** with butyl, cyclopropyl, and 1-cyclohexenyl group resulted in excellent yields of the corresponding products **2 b** (82 %), **2 c** (78 %), and **2 d** (81 %), respectively. However, a cyclohexene-fused product **2 e** was obtained in a somewhat lower yield (58 %). Installation of alkyl or aryl substituent at the β-position of the carbonyl group promotes spontaneous acyl migration. Thus, a mixture of **2 f** and **3 f** was obtained with an 86 : 14 ratio in 1.5 h (80 % yield). But after 24 h, complete conversion to **3 f** was observed with an identical yield. Similarly, a mixture of **2 g** and **3 g** was isolated with an 86 : 14 ratio in 0.5 h (78 %), but after 8 h complete conversion to **3 g** was achieved. In stark contrast, however, the trimethylsilyl group completely blocks the acyl migration, thus only **2 h** was isolated in 72 % yield without acylsilane **3 h** even at elevated temperature (100 °C, 4 h). The reaction of substrates bearing saturated tether provided carboxyamidation products **2 i** (58 %, *Z*/*E* = 2 : 1), **2 j** (76 %, *Z*/*E* = 5 : 1), and **2 p** (74 %, *Z*/*E* = 1.7 : 1) where the *Z*-selectivity deteriorated. The lower selectivity in this product type could be explained by the spontaneous *E*/*Z* isomerization mediated by the iron catalyst, as we observed variable isomeric ratios in different timeframes (details on page S-7 in Supporting Information).

Next, we explored the carboxyamidation of aryl-tethered *O*-acylhydroxamate **4**, which proceeds with high *Z*-selectivity to generate product **5** (Table 3). Substrates carrying a butyl group on the alkyne with varying acyl groups provided benzoate **5 a** (82 %), acetate **5 b** (72 %), and pivalate **5 c** (65 %), which formed only *Z*-alkene isomer. With a fixed benzoyl group, varying the substituent on the alkyne provided trimethylsilyl-substituted compound **5 d** (88 %), and terminal alkyne-derived product **5 e** (78 %). Benzoate and pivalate derivatives of an allyloxymethyl-containing substrates provided **5 f** (76 %) and **5 g** (65 %), while the corresponding 2-butynyloxymethyl-bearing substrate provided **5 h** (78 %) and **5 i** (64 %). Installation of an electron-withdrawing group such as fluoro or trifluoromethyl substituent on the aryl tether of **5 j** (74 %) and **5 k** (67 %) do not have a significant impact on the reaction efficiency. The phenyl-substituted compound **5 l** (85 %) was obtained in a similar yield compared to the corresponding alkyl-substituted counterpart, for example, **5 a** (82 %). Installation of a methoxy group on the aryl tether or the para position on the alkyne-substituted aryl group does not change the reaction profile; thus, **5 m** (90 %) and **5 n** (76 %) were obtained without events. On the other hand, replacing the methoxy group in **5 n** with a trifluoromethyl group completely changes the reaction pathway from *cis*-carboxyamidation to *trans*-hydroamidation, providing **5 o’** (54 %), the identity of which was confirmed by X-ray diffraction analysis. Elevating the temperature to 100 °C rendered the desired carboxyamidation product **5 o** successfully in 63 % yield. We believe this discrepancy is a result of a faster nucleophilic addition of the Fe-amide complex to the electron-deficient alkyne at 70 °C. However, a higher barrier is anticipated for the electron-deficient alkyne to react with the electrophilic Fe-nitrenoid to form **5 o**, thus an elevated temperature (100 °C) is required (details on pages S-7 and S-8 in Supporting Information).

Based on the spontaneous thermal O⟶N migration of the acyl group in **2 f** and **2 g** to form *N*-acyl lactams **3 f** and **3 g**, we anticipated that nucleophilic amine such as 4-(dimethylamino)pyridine (DMAP) should be able to catalyze the acyl migration for other alkenyl- and aryl-tethered carboxyamidation products. With this anticipation, we treated the alkenyl-tethered compounds **2** with DMAP (30 mol %, CH_2_Cl_2_, 35 °C) and found that O⟶O acyl migration occurred preferentially over the expected O⟶N acyl migration to generate 2-(5-acylpyrrolyl)-benzoates **6 a-d** (Table 4).^[20]^ The structure of product **6 b** was unambiguously confirmed by single crystal X-ray diffraction analysis (See Supporting Information for the details). Because of the preferred O⟶N acyl migration to form **3 f** and **3 g**, pyrrolyl benzoates **6 f** and **6 g** were not obtained. While the cyclohexene-fused compound **2 e** decomposed under the conditions, the trimethylsilyl group-containing **2 h** and saturated hydro-carbon-tethered carboxyamidation products **2 i** and **2 j** were recovered.

We further extended our exploration of DMAP-catalyzed acyl migration to the arene-tethered carboxyamidation products **5**, which undergo selective O⟶N acyl migration to produce *N*-acyl-3-acylisoindolinones (Table 5).^[20]^ From butyl-substituted (R=Bu) enol carboxylates **5 a** and **5 b**, smooth transfer of benzoyl and acetyl groups occurred to form **7 a** (88 %) and **7 b** (65 %). The structure of an acetyl group migrated product was unambiguously confirmed by single crystal X-ray diffraction analysis. The DMAP-catalyzed O⟶N benzoyl migration was successfully achieved with R=alkyl substituents to provide products **7 f** (82 %), **7 h** (80 %), **7 j** (80 %), and **7 k** (74 %). The migration with R=aryl substituents occurred uneventfully to deliver the corresponding products **7 l** (92 %), **7 m** (70 %), **7 n** (70 %), and **7 o** (78 %). From these reactions, no examples of O⟶O acyl migration to generate isoindole derivatives were observed.^[6b]^

**Scheme 3.**
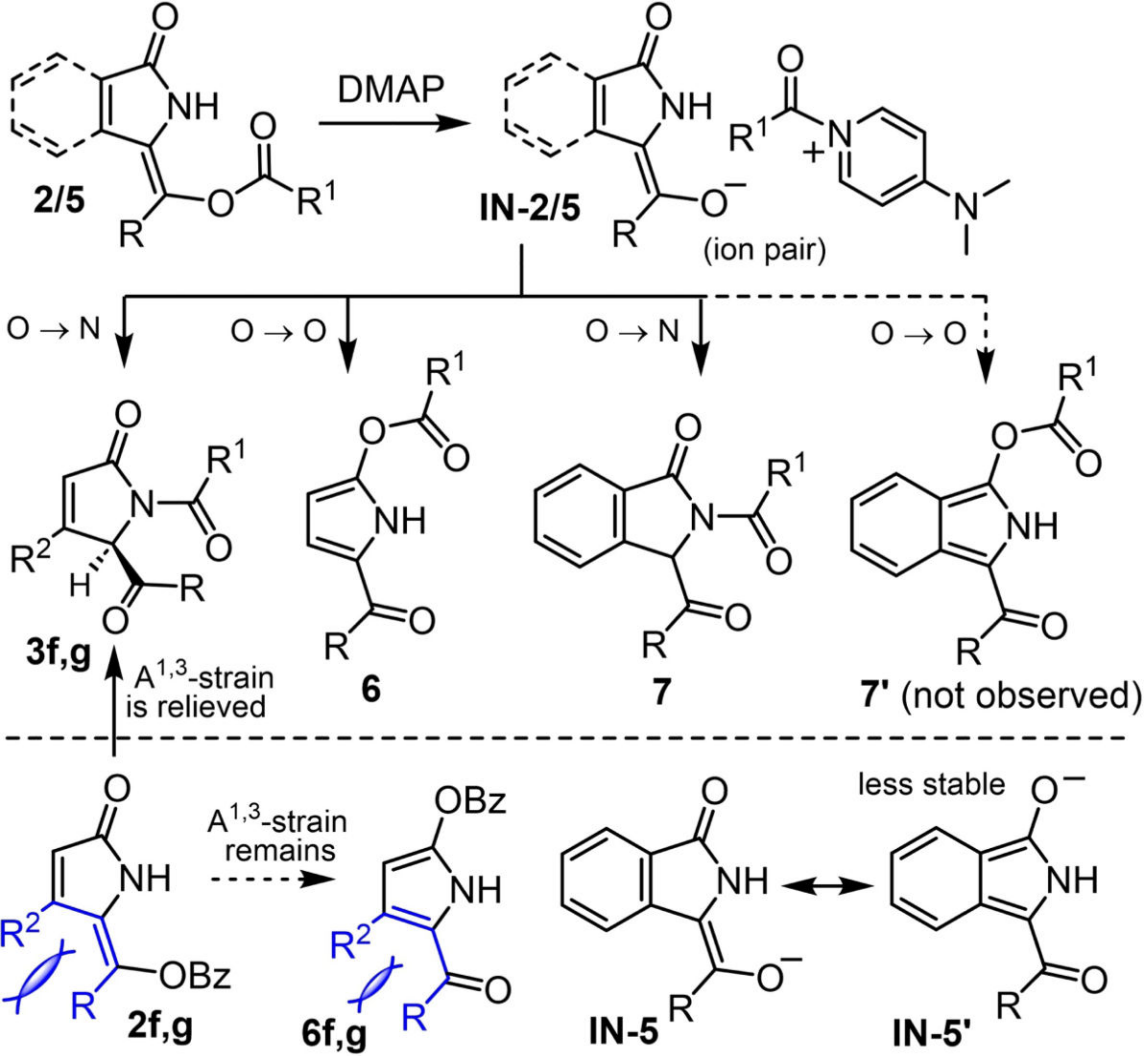
Mechanisms for O⟶O and O⟶N acyl migrations.

In terms of a mechanism for DMAP-catalyzed acyl migration, we propose that the enol carboxylates are attacked by DMAP to form ion pair **IN-2/5** with an extended enolate and a DMAP-acyl adduct (Scheme 3). Depending on the structure of the linker of the enolate and the amide moiety, either O⟶O or O⟶N acyl migration would be preferred. With an alkenyl tether that contains no extra substituent, O⟶O acyl migration from **2** to pyrrole **6** is favored over O⟶N migration because of the aromatic stabilization of a pyrrole unit. On the other hand, the severe A^1,3^-strain imposed by the β-substituent in **2 f**,**g** will be relieved by O⟶N acyl migration forming **3 f**,**g**, whereas the O⟶O acyl migration does not relieve the A^1,3^-strain, thus the corresponding pyrroles **6 f,g** were not detected.^[21]^ For arene-tethered compounds **5**, the less stable resonance forms **IN-5**’ compared to **IN-5** would disfavor trapping of the acyl group on the enolate oxygen to generate isoindoles **7**’,^[22]^ instead acyl trapping on the nitrogen in **IN-5** will be preferred to generate *N*-acyl-3-acylisoindolinones.

**Scheme 4.**
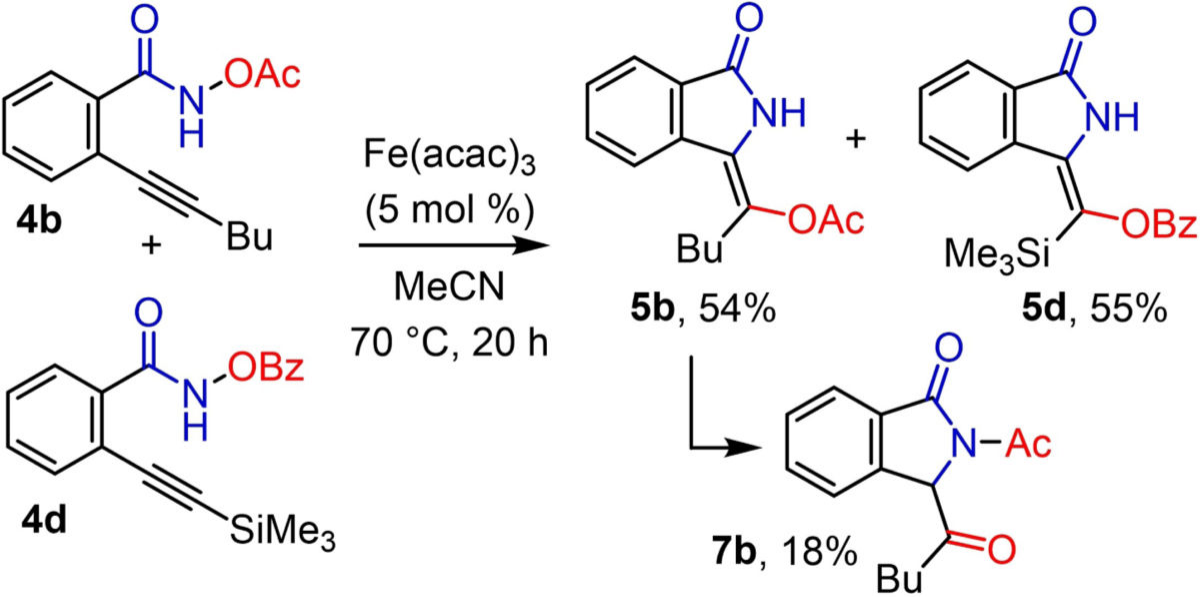
Mechanistic insight from crossover experiment for acyl group exchange.

To gain insight into the reaction mechanism of the iron-catalyzed carboxyamidation, a crossover experiment with *O*-acylhydroxamates **4 b** and **4 d** was carried out under the standard conditions (Scheme 4). Careful analysis of the reaction mixture revealed the carboxyamidation products **5 b** and **5 d** along with a small amount of O⟶N acetyl-migrated product **7 b**. Prolonged reaction time resulted in extensive conversion of **5 b** to **7 b**, yet there was no evidence for the formation of carboxylate exchange products. This result suggests that the carboxylate transfer from each reactant to the catalyst followed by carboxyamidation should occur in an intramolecular fashion. This interpretation, however, does not corroborate the experimental results in Table 1, which indicate that the carboxylate exchange with external nucleophiles readily happens.

**Scheme 5.**
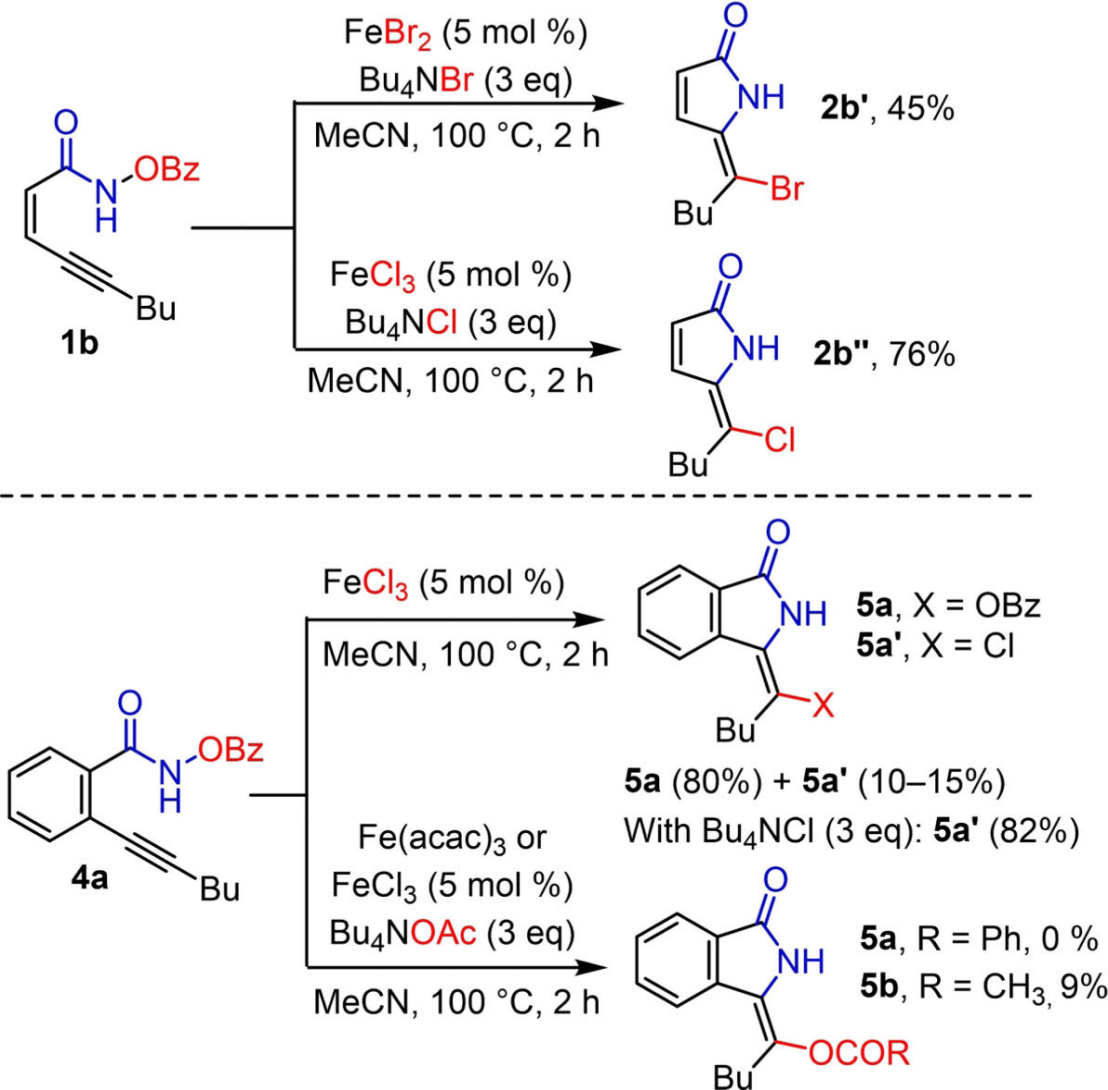
Carboxylate-halogen exchange and carboxylate crossover experiments.

To further probe the nucleophile exchange, we employed both alkene- and arene-tethered *O*-benzoylhydroxamates **1 b** and **4 a** and carried out carboxyamidation under the standard conditions in the presence of external nucleophiles (Scheme 5). Treating **1 b** with FeBr_2_ (5 mol %, CH_3_CN, 100 °C, 2 h) in the presence of Bu_4_NBr (3 equiv.) provided **2 b**’ (45 %) as the sole product. Similarly, treating **1 b** with FeCl_3_ (5 mol %, CH_3_CN, 100 °C, 2 h) in the presence of Bu_4_NCl (3 equiv.) provided **2 b̋** (76 %). Under the same conditions in the absence of an external chloride source, **4 a** gave a mixture of **5 a** (80 %) and **5 a**’ (10–15 %), whereas, in the presence of Bu_4_NCl (3 equiv.), only chloride incorporated product **5 a**’ (82 %) was obtained. The reaction of **4 a** with FeCl_3_ or Fe(acac)_3_ as the catalyst in the presence of Bu_4_OAc (3 equiv.) provided only acetate-incorporated product **5 b** without benzoate-containing product **5 a**. Low yield of the reaction was likely caused by the stronger basicity of Bu_4_OAc, which induced a predominant Lossen rearrangement to generate 2-(hex-1-yn-1-yl)aniline (see section 2.5 in the Supporting Information for details).

**Scheme 6.**
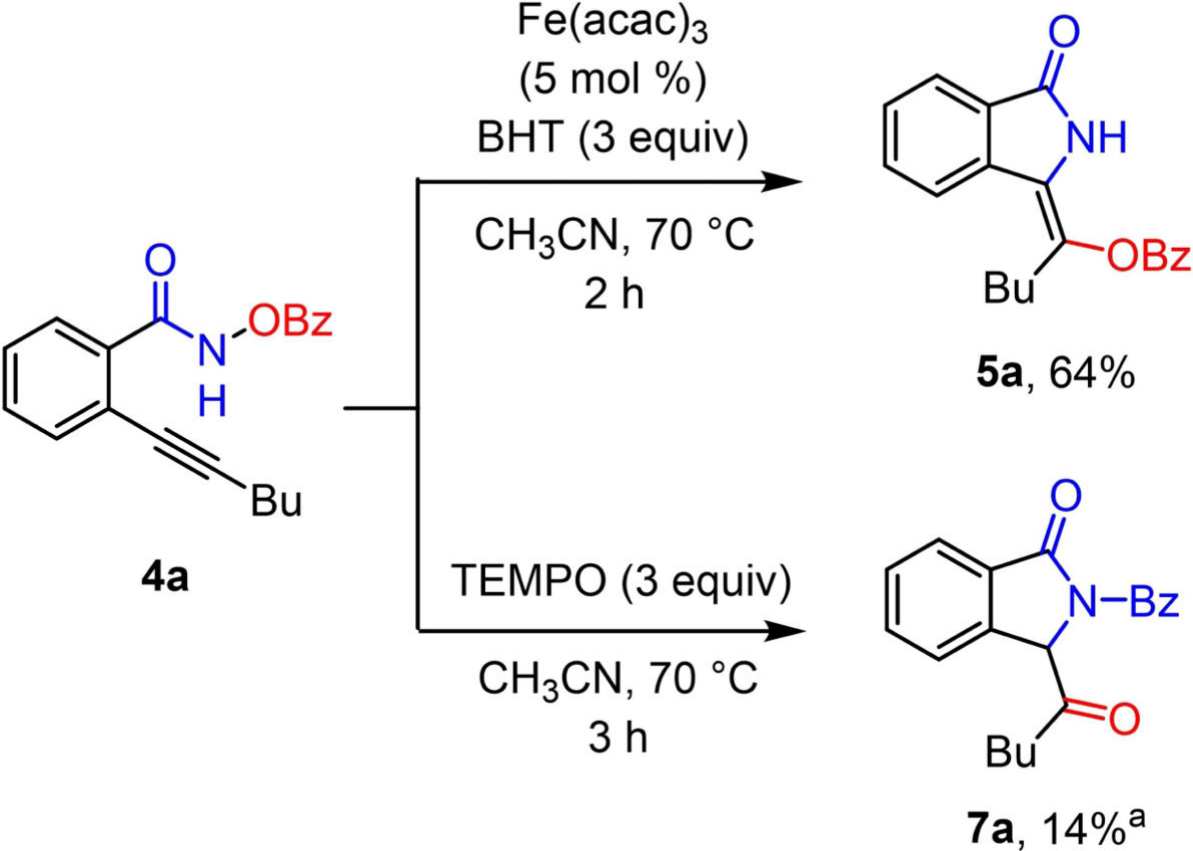
Attempts to trap the plausible radical intermediate. [a] 46 % of **4a** was recovered.

To gain insight into the nature of a putative acyl nitrenoid intermediate, BHT (2,6-di-*tert*-butyl-4-methylphenol) as a radical scavenger was employed (Scheme 6).^[23a]^ From the reaction, carboxyamidation product **5 a** was generated smoothly with slightly reduced yield (64 %) with no appreciable change in the required reaction time. On the other hand, the rate was significantly decreased by the additional 3 equivalents of TEMPO (2,2,6,6-tetramethyl-1-piperidinyloxy), giving only a small amount of **7 a** (14 %) along with 46 % of recovered **4 a** after 3 h. The reactivity difference between BHT and TEMPO could cause the discrepancy between these two results. Even though BHT is known to be an effective radical scavenger, the faster intramolecular C—N and C—O bond-forming events outcompeted the intermolecular hydrogen atom transfer from BHT to putative radical intermediates. On the other hand, aminoxy radical-containing TEMPO is expected to be more reactive to interact with radical intermediates, thus the carboxyamidation reaction was prohibited.

**Figure 1.**
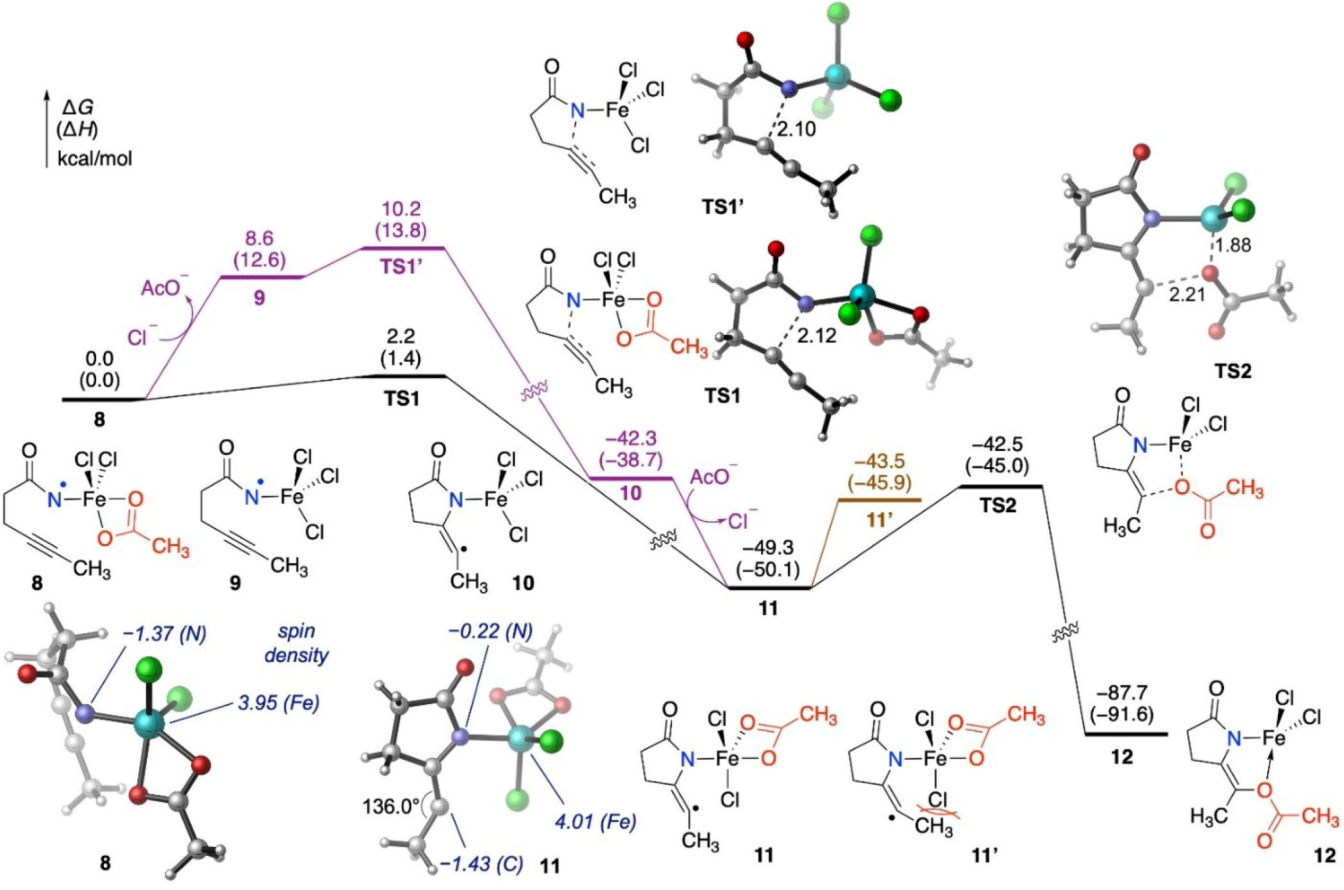
Computed reaction energy profile of the Fe-catalyzed carboxyamidation. All energies are with respect to the Fe-nitrenoid complex **8**.

We performed density functional theory (DFT) calculations to gain insights into the mechanism of the Fe-catalyzed intramolecular carboxyamidation (Figure 1). The calculations were carried out with alkyne-tethered *O*-acylhydroxamate **1 p** as a model substrate at the M06/SDD-6–311 +G(d,p)/SMD(CH_3_CN)//B3LYP D3(BJ)/SDD-6–31G(d) level of theory. The Fe-nitrenoid intermediate **8** generated from **1 p**^[23]^ is quartet spin state with trigonal bipyramidal geometry, whereas both the doublet and sextet states of **8** are much less stable (see Figure S1 in the Supporting Information). The Mulliken spin densities indicate that intermediate **8** is an antiferromagnetically coupled high-spin iron imidyl radical complex^[24]^ (see Figure S4 in the Supporting Information for the Mulliken spin densities of other species and transition states in the catalytic cycle). The substantial radical character on the nitrogen atom of the imidyl radical **8** promotes the intramolecular radical cyclization^[25]^ with the tethered alkyne via **TS1** to form alkenyl radical **11**. This radical cyclization step requires a low activation barrier of 2.2 kcal/mol and is highly exergonic. Several alternative pathways, including concerted [3 + 2]^[11a,16]^ and [5 + 2] cycloadditions, nitrene-alkyne metathesis (NAM) via [2 + 2] cycloaddition,^[12]^ and nucleometallation of a π-alkyne Fe(III) complex, were ruled out computationally (see Supporting Information for details). The computed spin density indicates that intermediate **11** is an alkenyl radical with substantial radical character on the C(sp^2^)-carbon, rather than the corresponding carbocation. Subsequent intramolecular acyloxy transfer from Fe to the carbon-centered radical occurs via a five-membered cyclic transition state (**TS2**), leading to iron amido complex **12**, which upon protodemetallation gives the *cis*-carboxyamidation product.

The key C—O bond forming step via acyloxy transfer is highly exergonic and kinetically facile with a low barrier of 6.8 kcal/mol with respect to **11**. This promotes the kinetic selectivity to form the *cis*-carboxyamidation product rather than the *trans*-isomer. The *E*/*Z* isomerization^[26]^ of the alkenyl radical intermediate **11** leads to a much less stable isomer **11**’ (5.8 kcal/mol higher energy than **11**) due to steric repulsions with the Fe(OAc)Cl_2_ moiety and the methyl group. Therefore, we expect the *E/Z* isomerization to form **11**’ and subsequent outersphere acyloxy addition is kinetically unfavorable compared to the intramolecular acyloxy transfer via **TS2**, which is consistent with the *cis* selectivity observed experimentally. A competing radical cyclization pathway involving iron trichloride nitrenoid **9** were also considered (purple pathway, Figure 1), because the alkenyl radical intermediate **10** may be an intermediate for a chlorine atom transfer to form a chloroamidation product. This trichloride pathway is less favorable than that with iron dichloride acetate nitrene **8** because of the stronger binding of acetate than chloride anion to the Fe center. Taken together, our DFT calculations indicate that the Fe-catalyzed carboxyamidation prefers a stepwise mechanism^[27]^ involving radical cyclization with an Fe imidyl intermediate, followed by intramolecular acyloxy transfer from Fe to an alkenyl radical intermediate to form a *cis*-carboxyamidation product.

## Conclusions

We developed iron(III)-catalyzed intramolecular carboxyamidation of alkyne-tethered *O*-acylhydroxamates followed by either thermally induced or 4-(dimethylamino)pyridine-catalyzed O⟶O or O⟶N acyl group migration. DFT calculations indicate that the iron-catalyzed carboxyamidation involves a nitrenoid-mediated stepwise radical cyclization/acyloxy transfer mechanism to form C—N and C—O bonds sequentially. Both *Z*-alkene and arene-tethered substrates are suitable for the carboxyamidation. Upon treating with 4-(dimethylamino)pyridine, the *Z*-alkene-tethered carboxyamidation products underwent selective O⟶O acyl migration to generate 2-acyloxy-5-acyl pyrroles. Thermal O⟶N acyl migration occurred during carboxyamidation if the alkenyl tether contains a β-alkyl or aryl substituent. This is most likely because of a severe A^1,3^-strain between the β-alkyl/aryl group and the substituent on the enol carboxylate. On the other hand, the arene tether-containing compounds selectively undergo O⟶N acyl migration to provide *N*-acyl-3-acylisoindolinones, and the corresponding O⟶O acyl migration forming isoindole derivatives was not observed. No example of O⟶C acyl migration to α-carbon of the ketone was observed, which is believed to be the consequence of the severe steric congestion at the reaction center to form a quaternary center. Compared to the reported difunctionalization of alkynes, a unique feature of the current carboxyamidation method is the recycling of the carboxylate extruded from the nitrenoid precursors, thus no external supply of nucleophile is needed. Also, the structure-dependent divergent acyl group migration of the enol carboxylate of carboxyamidation products would allow for the synthesis of various heterocycles functionalized with oxygen and nitrogen constituents.

## Experimental Section

### General procedure for the carboxyamidation reaction:

A Schlenk tube was charged with *O*-acylhydroxamates **1** or **4** (0.15 mmol) in freshly distilled acetonitrile (3 mL). FeCl_3_ (0.0075 mmol, 5 mol %) was then added and the mixture was degassed using freeze-pump-thaw techniques. The reaction was heated at 70 °C and monitored by TLC until completion. The resulting mixture was filtered through a short pad of celite and concentrated *in vacuo.* The crude material was then dissolved in dichloromethane (6 mL) and washed with saturated NaHCO_3_ solution (3 mL). The organic layers were dried over anhydrous Na_2_SO_4_, filtered, and concentrated *in vacuo.* The pure product was obtained by chromatography (SiO_2_) in 58–90 % yields.

### General procedure for the acyl group migration reactions:

To the carboxyamidation product **2** or **5** (0.1 mmol) in 2 mL of CH_2_Cl_2_ was added DMAP (30 mol %, 0.03 mmol). The reaction mixture was heated at 35–40 °C and monitored by TLC until completion. The resulting mixture was diluted with CH_2_Cl_2_ (3 mL) and washed with saturated NaHCO_3_ solution (3 mL). The organic layers were dried over anhydrous Na_2_SO_4_, filtered, and concentrated *in vacuo.* The crude material was purified by chromatography (SiO_2_) to give products in 58–94 % yields.

### Characterization of 2 a:

White solid (37.1 mg, 85 %); ^**1**^**H NMR** (500 MHz, CDCl_3_) δ 8.39 (brs, 1H), 8.17 (d, *J* = 7.0 Hz, 2H), 7.66 (t, *J* = 7.5 Hz, 1H), 7.52 (t, *J* = 7.3 Hz, 4H), 7.44–7.38 (m, 3H), 7.29 (d, *J* = 4.0 Hz, 1H), 6.15 (d, *J* = 4.1 Hz, 1H); ^**13**^**C NMR** (125 MHz, CDCl_3_) δ 170.68, 164.10, 136.03, 134.53, 134.20, 132.78, 130.43, 130.12, 129.64, 128.86, 128.77, 128.45, 128.13, 125.77; **HRMS** (ESI) calcd for C_18_H_14_NO_3_ [M+ H]^+^ 292.0974, found 292.0976.

### Characterization of 5 a:

White solid (39.5 mg, 82 %); ^**1**^**H NMR** (500 MHz, CDCl_3_) δ 9.11 (brs, 1H), 8.24–8.17 (m, 2H), 7.74 (d, *J* = 7.9 Hz, 1H), 7.68 (t, *J* = 7.5 Hz, 1H), 7.60 (td, *J* = 7.6, 1.2 Hz, 1H), 7.54 (t, *J* = 7.9 Hz, 3H), 7.43 (t, *J* = 7.5 Hz, 1H), 2.91 (t, *J* = 7.7 Hz, 2H), 1.67 (p, *J* = 7.5 Hz, 2H), 1.47 (h, *J* = 7.3 Hz, 2H), 0.94 (t, *J* = 7.3 Hz, 3H); ^**13**^**C NMR** (125 MHz, CDCl_3_) δ 167.18, 164.41, 135.27, 134.91, 133.79, 132.09, 130.84, 130.43, 129.08, 128.62, 128.41, 125.28, 123.99, 122.37, 30.39, 29.30, 22.47, 13.90; **HRMS** (ESI) calcd for C_20_H_20_NO_3_ [M +H]^+^ 322.1443, found 322.1443.

### Characterization of 3 f:

Light-yellow oil (34.2 mg, 80 %); ^**1**^**H NMR** (500 MHz, CDCl_3_) δ 7.65 (d, *J* = 7.4 Hz, 2H), 7.54 (t, *J* = 7.4 Hz, 1H), 7.43 (t, *J* = 7.4 Hz, 2H), 5.97 (s, 1H), 5.27 (s, 1H), 2.58 (dt, *J* = 18.0, Hz, 1H), 2.48 (dt, *J* = 17.9, 7.1 Hz, 1H), 2.09 (s, 3H), 1.60 (p, *J* = Hz, 2H), 1.30 (h, *J* = 7.6 Hz, 2H), 0.89 (t, *J* = 7.4 Hz, 3H); ^**13**^**C NMR** (125 MHz, CDCl_3_) δ 203.06, 168.84, 168.59, 157.42, 133.42, 132.20, 128.91, 127.85, 123.84, 73.21, 38.13, 25.15, 22.19, 15.00, 13.81; **HRMS** (ESI) calcd for C_17_H_20_NO_3_ [M+H]^+^ 286.1443, found 286.1440.

### Characterization of 6 a:

White solid (27.3 mg, 94 %); ^**1**^**H NMR** (500 MHz, CDCl_3_) δ 10.52 (brs, 1H), 8.19 (d, *J* = 6.9 Hz, 2H), 7.85 (d, *J* = 6.9 Hz, 2H), 7.67 (t, *J* = 7.4 Hz, 1H), 7.55 (t, *J* = 7.4 Hz, 1H), 7.51 (t, *J* = 7.8 Hz, 2H), 7.45 (t, *J* = 7.7 Hz, 2H), 6.86 (dd, *J* = 4.1, 2.6 Hz, 1H), 6.21 (dd, *J* = 4.1, 2.4 Hz, 1H); ^**13**^**C NMR** (125 MHz, CDCl_3_) δ 13 C NMR δ 184.27, 162.81, 143.52, 138.10, 134.29, 131.72, 130.40, 128.92, 128.82, 128.32, 128.14, 124.75, 119.89, 98.15.; **HRMS** (ESI) calcd for C_18_H_14_NO_3_ [M+ H]^+^ 292.0974, found 292.0974.

### Characterization of 7 a:

Light-yellow solid (28.2 mg, 88 %); ^**1**^**H NMR** (500 MHz, CDCl_3_) δ 7.90 (d, *J* = 7.7 Hz, 1H), 7.76–7.71 (m, 2H), 7.70 (dd, *J* = 7.6, 1.2 Hz, 1H), 7.62–7.54 (m, 3H), 7.48 (t, *J* = 7.7 Hz, 2H), 5.91 (s, 1H), 2.70–2.51 (m, 2H), 1.58 (p, *J* = 7.4 Hz, 2H), 1.26 (qd, *J* = , 5.4 Hz, 2H), 0.85 (t, *J* = 7.4 Hz, 3H); ^**13**^**C NMR** (125 MHz, CDCl_3_) δ 203.04, 170.17, 166.33, 139.00, 134.65, 133.78, 132.24, 130.64, 129.88, 128.89, 127.94, 125.97, 122.84, 68.08, 38.25, 25.20, 22.10, 13.75; ; **HRMS** (ESI) calcd for C_20_H_20_NO_3_ [M+H]^+^ 322.1443, found 322.1443.

Deposition Number(s) 2269723 (for **5o’**), 2269724 (for **6b**), 2269722 (for **7b**) contain(s) the supplementary crystallographic data for this paper. These data are provided free of charge by the joint Cambridge Crystallographic Data Centre and Fachinformationszentrum Karlsruhe Access Structures service.

## Supplementary Material

SI

## Figures and Tables

**Table 1.** Catalyst screening for carboxyamidation and haloamidation.

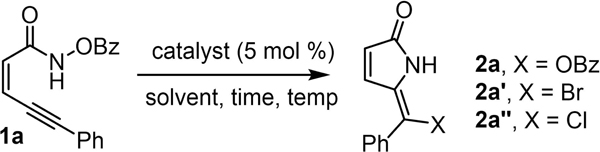						
entry	catalyst	solvent	temp [°C]	time [h]	Yields [%]^[a]^	**2 a : 2 a’**/**2a̋**

1	PtCl_2_/CO	(ClCH_2_)_2_	100	8	–^[b]^	–
2	FeBr_2_	CH_3_CN	100	1	91	8.1 : 1^[c]^
3	FeCl_3_	CH_3_CN	100	0.5	96	5.9 : 1^[d]^
4	FeBr_2_/Br^−^^[e]^	CH_3_CN	100	2	55	1 : 2.7
5	FeCl_3_/Cl^−^^[f]^	CH_3_CN	100	2	75	**2a̋** only
6	Fe(acac)_3_	CH_3_CN	70	1.5	85	**2 a** only
7	None	CH_3_CN	100	14	–^[b]^	–

[a] Isolated yield.

[b] Decomposed.

[c] Minor product is **2 a**’.

[d] Minor product is **2 a̋**.

[e] Br^‒^=Bu_4_NBr (3 equiv.).

[f] Cl^‒^=Bu_4_NCl (3 equiv.).

**Table 2.** Carboxyamidation with alkenyl and alkyl tethered substrates.

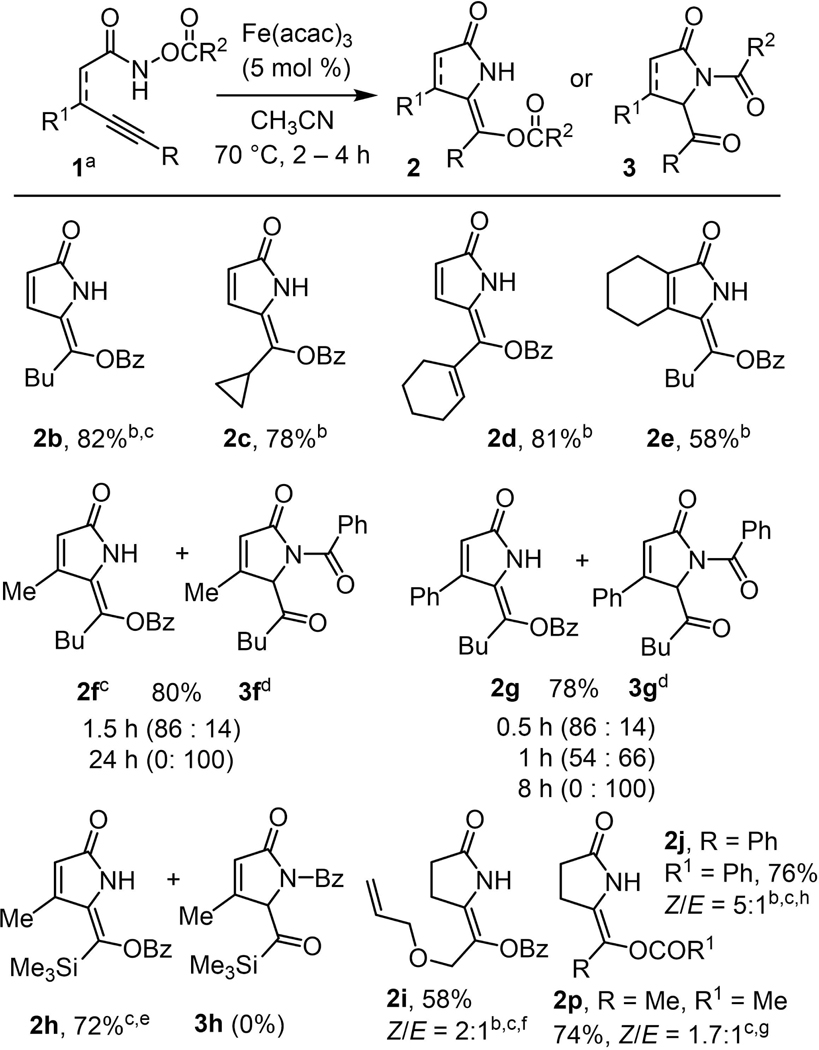

[a] 0.150 mmol scale.

[b] With FeCl_3_ (5 mol %), a similar yield of carboxyamidation product was obtained along with the chloride-incorporated product (10–15 %).

[c] Geometry of the alkene was confirmed by NOE experiment.

[d] Faster acyl migration occurred with FeCl_3_ under otherwise same conditions to give only **3 f** and **3 g**.

[e] Conversion of **2 h** to **3 h** was not observed at 100 °C (4 h).

[f] *E*/*Z* isomers were separated after Boc-protection.

[g] *E*/*Z* isomers could not be separated even after Boc-protection.

[h] Reaction time: 14 h.

**Table 3.** Carboxyamidation with aryl tethered substrates.

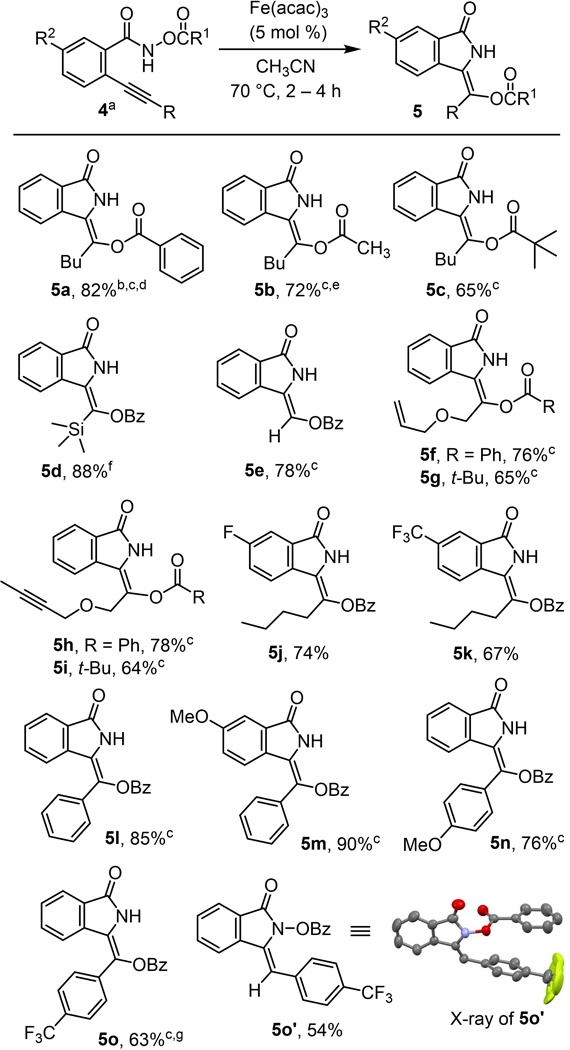

[a] 0.150 mmol scale, unless noted otherwise.

[b] An extended reaction time (24 h) did not induce benzoate migration to generate **7 a**.

[c] With FeCl_3_ (5 mol%), a similar yield of carboxyamidation product was obtained along with the chloride-incorporated product (10–15 %).

[d] Reaction with 1 mmol of substrate **4a** gave an 80% yield.

[e] After 16 h, a mixture of **5b** and the corresponding acetyl group-migrated product **7b** was obtained in a 3:1 ratio.

[f] Geometry of the alkene was confirmed by NOE experiment. [g] 100°C.

**Table 4.** DMAP-catalyzed O⟶O acyl migration.

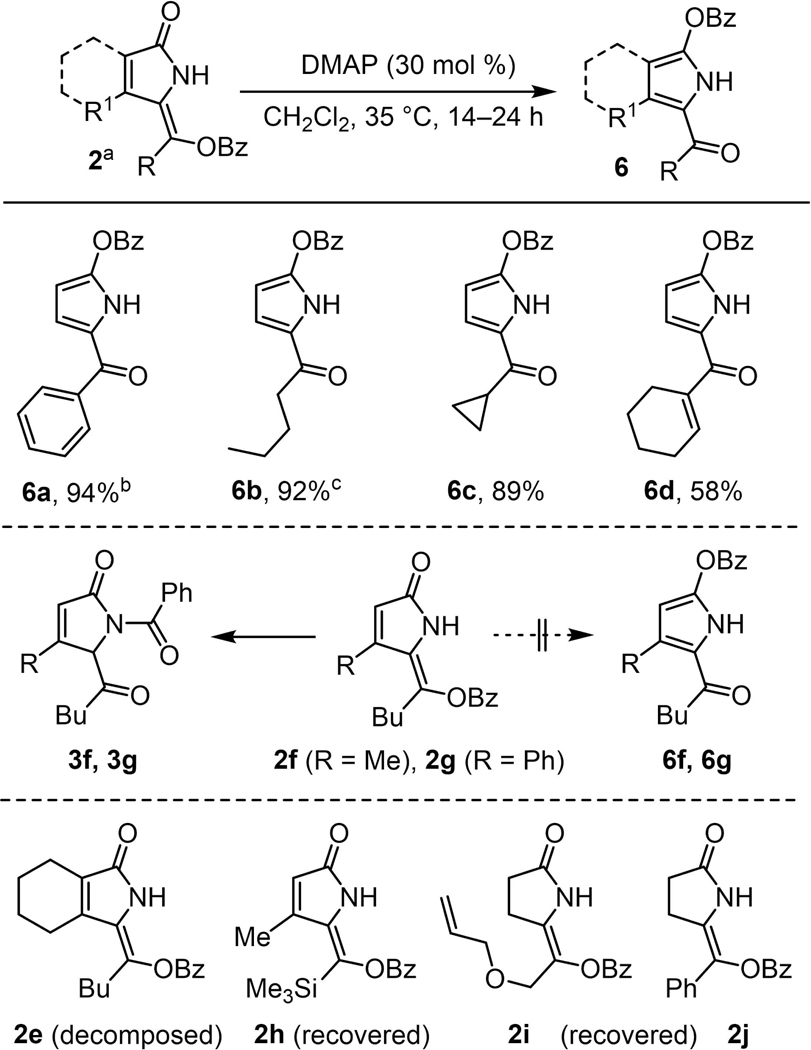

[a] 0.150 mmol scale.

[b] In CH_3_CN, 40 °C, 16 h.

[c] Confirmed by X-ray diffraction analysis (CCDC: 2269724).

**Table 5.** DMAP-catalyzed O⟶N acyl migration.

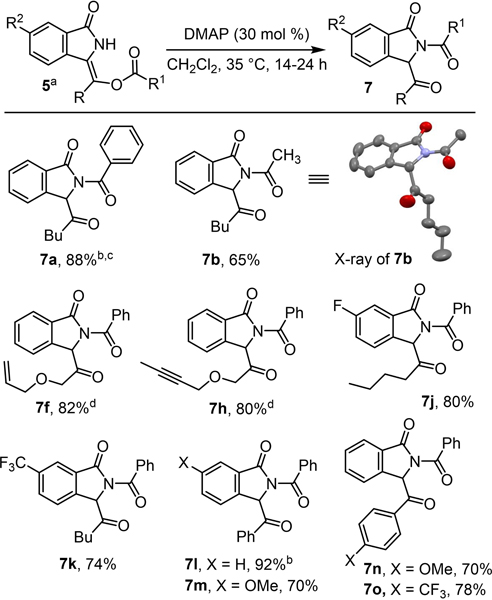

[a] 0.150 mmol scale, unless noted otherwise.

[b] In CH_3_CN, 40°C, 14 h.

[c] A reaction with 1 mmol of substrate **5a** gave 85% yield.

[d] The corresponding pivaloyl migration in **5g** and **5i** did not occur (starting material recovered).

## Data Availability

The data that support the findings of this study are available in the supplementary material of this article.
