# Supplementary material for: Intramolecular Carboxyamidation of Alkyne-Tethered O-Acylhydroxamates through Formation of Fe(III)-Nitrenoids
Source: Chemistry. Author manuscript; Available in PMC 2025 Feb 11. (PMC11812590; doi:10.1002/chem.202303428)
Supplement: SI [file NIHMS2037238-supplement-SI.pdf]

// Chemistry–A European Journal

Supporting Information

Intramolecular Carboxyamidation of Alkyne-Tethered *O*-Acylhydroxamates through Formation of Fe(III)-Nitrenoids

Siyuan Su, Yu Zhang, Peng Liu,* Donald J. Wink, and Daesung Lee*

Table of Contents

1. Materials and Methods S-2

2. Experimental Details S-2

3. Characterization Data of Substrates S-9

4. Characterization Data of Products S-13

5. X-Ray Crystallographic Data S-22

6. Computational Data S-25

7. References S-47

8. ^{1}**H** and ^{13}**C NMR** Spectra S-49

1. Material and Methods

All Reactions were carried out in oven-dried glassware under inert atmosphere unless otherwise noted. The carboxyamidation reactions were performed under argon using standard Schlenk techniques. All reagents and compounds were purchased from Aldrich, Acros, TCI America or Oakwood and used as received unless otherwise noted. Toluene, dichloromethane, triethylamine, and acetonitrile were distilled over calcium hydride (CaH_2) under nitrogen. Tetrahydrofuran was distilled over sodium and benzophenone under nitrogen. Column chromatography was performed using silica gel 60 Å (32–63 mesh) purchased from Silicycle Inc. Analytical thin layer chromatography (TLC) was performed on 0.25 mm E. Merck precoated silica gel 60 (particle size 0.040–0.063 mm). Yields refer to chromatographically and spectroscopically pure isolated compounds unless otherwise stated. ^{1}H and ^{13}C NMR spectra were recorded on a Bruker AV-500 spectrometer. ^{1}H NMR chemical shifts (δ) are reported in parts per million (ppm) downfield of TMS and are referenced relative to the residual proteated solvent peak ($CDCl_3$ (7.26 ppm)). ^{13}C NMR chemical shifts (δ) are reported in parts per million downfield of TMS and are referenced to the carbon resonance of the solvent ($CDCl_3$ (77.03 ppm)). Multiplicities are indicated by s (singlet), brs (broad singlet), d (doublet), t (triplet), q (quartet), p (quintet), h (sextet), dd (doublet of doublet), dt (doublet of triplet), m (multiplet) and so on. ^{1}H NMR signals that fall within a ca. 0.3 ppm range are generally reported as a multiplet, with a range of chemical shift values corresponding to the peak or center of the peak. Coupling constants, *J*, are reported in Hz (Hertz). Electrospray ionization (ESI) and Electron impact (EI) mass spectra were recorded on a Waters Micromass Q-Tof Ultima and Micromass 70-VSE, respectively in the University of Illinois at Urbana-Champaign

2. Experimental Details

2.1 Preparation of the carboxylic acids

2.1.1 General procedures:

Sonogashira coupling: To the aryl or vinyl halides (5 mmol) in 20 mL of THF was added $Pd(PPh_3)_2Cl_2$ (0.1 mmol, 2 mol %), CuI (0.15 mmol, 3 mol %), terminal alkyne (6.5 mmol, 1.3 equiv) and triethylamine (7 mL). The reaction mixture was allowed to stir at room temperature (for aryl or vinyl iodides) or heated at 60 °C (for aryl or vinyl bromides) and monitored by TLC until completion. The reaction mixture was then quenched with 20 mL of sat'd NH_4Cl solution and the organic layer was separated. The aqueous layer was extracted with EtOAc and the combined organic layers were washed with brine, dried over anhydrous Na_2SO_4, filtered, and concentrated *in vacuo*. The pure product was obtained by chromatography (SiO_2) in 60–85% yields.

Hydrolysis: To a suspension of LiOH (powder, 15 mmol, 3 equiv) in water (3 mL) and ethanol (6 mL) was added esters (5 mmol) in THF (21 mL). The reaction mixture was allowed to stir under room temperature for 8–12 h (monitored by TLC). Upon completion, the mixture was acidified with 1 N HCl (15–20 mL) and extracted with EtOAc. The combined organic layers were washed with brine, dried over anhydrous Na_2SO_4, filtered, and concentrated *in vacuo*. The pure product was obtained by chromatography (SiO_2) in 50–60% yields.

Jones oxidation: To a solution of aldehydes or primary alcohols (5 mmol) in acetone (35 mL) was added Jones reagent (2.7 M, 12.5 mmol, 2.5 equiv) at 0 °C. The reaction was kept at 0 °C and monitored by TLC until completion (30 min to 1 h). Water (35 mL) was added into the red solution and the mixture was concentrated *in vacuo* to a half of the volume. The dark green water layer was extracted with EtOAc, and the combined organic layers were washed with brine, dried over anhydrous Na_2SO_4, filtered, and concentrated *in vacuo*. The pure carboxylic acid was obtained by chromatography (SiO_2) in 55–74 % yields.

2.1.2 Preparation of carboxylic acids corresponding to substrates 1:

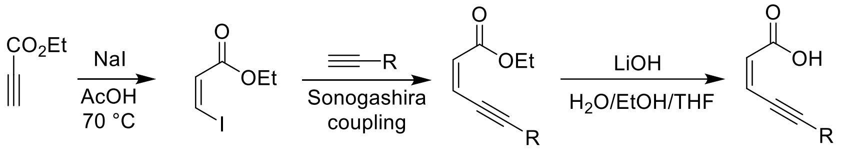

Vinyl iodide **S1** was prepared from ethyl propiolate according to the reported protocol.[1] The Sonogashira coupling and hydrolysis were performed following the general procedures to obtain carboxylic acids **S3** corresponding to **1a-1d**.

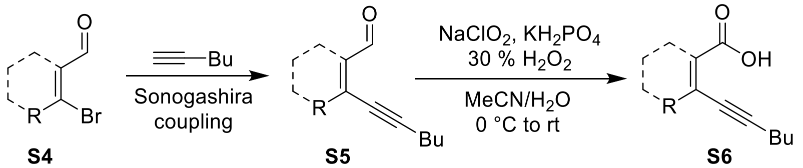

The bromoaldehydes **S4** was prepared from the corresponding ketones according to the reported protocol.[2] Sonogashira coupling was carried out following the general procedure, and carboxylic acids **S6** corresponding to **1e** and **1g** were prepared by the Pinnick oxidation.[3]

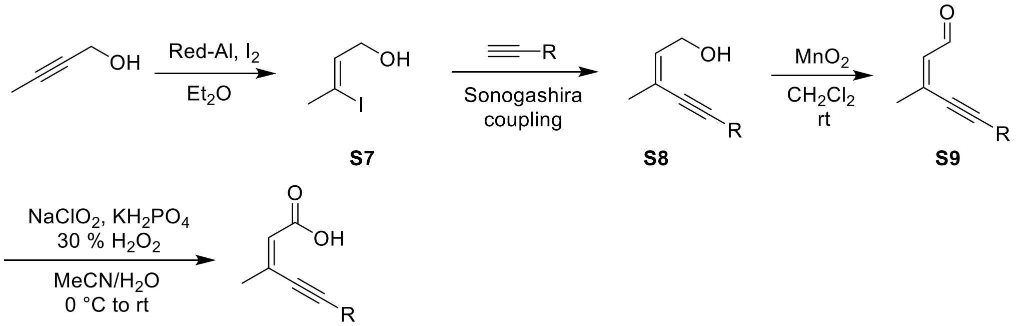

Vinyl iodide **S7** was prepared from 2-butyn-1-ol according to the reported protocol.[4] The Sonogashira coupling was performed following the general procedure to obtain **S8**. The alcohol group was first oxidized to aldehyde following the procedure described below, and the aldehyde was oxidized to carboxylic acids corresponding to **1f** and **1h** by the Pinnick oxidation.[3]

The alcohol **S8** (3 mmol) in CH_2Cl_2 (30 mL) was added MnO_2 (45 mmol, 15 equiv). The reaction was allowed to stir at room temperature and monitored by TLC until completion. The crude

material was obtained by filtration through a short pad of silica gel and concentration *in vacuo*. The crude of aldehyde **S9** was used in the next step without further purification.

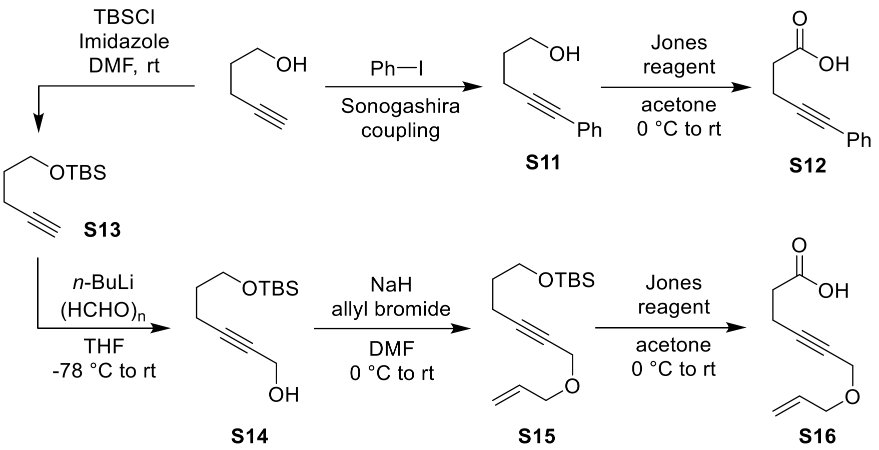

Carboxylic acids corresponding to **1i** and **1g** were prepared from 4-pentyn-1-ol.

The Sonogashira coupling with iodobenzene following the general procedure gave intermediate **S11**, which was then oxidized to carboxylic acid **S12** by Jones oxidation.

The TBS ether **S13** was prepared according to the reported procedure.[5] Propargyl alcohol **S14** and allyl ether **S15** were obtained following the documented reaction protocols.[6,7] The protected alcohol was converted to carboxylic acid **S16** by the Jones oxidation following the general procedure.

2.1.3 Preparation of carboxylic acids corresponding to substrates 4

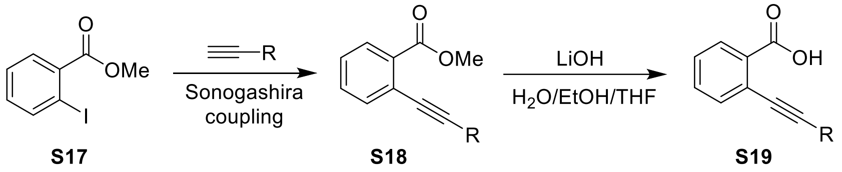

The ester **S17** was prepared from 2-iodobenzoic acid following the reported protocol.[8] The carboxylic acids **S19**, which correspond to substrates **4a-4c** and **4l** were prepared by Sonogashira coupling and Jones oxidation according to the general procedures.

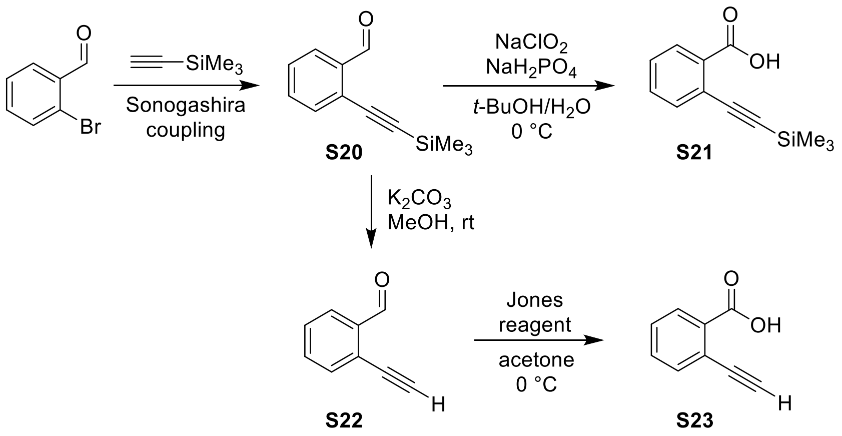

S20 was obtained from 2-bromobenzaldehyde by the Sonogashira coupling. The Pinnick oxidation was carried out to give the carboxylic acid **S21** corresponding to **4d**.[9]

Desilylation of **S20** was performed according to the literature,[10] the resulting aldehyde **S22** was converted to the carboxylic acid corresponding to **4e** by Jones oxidation following the general procedure.

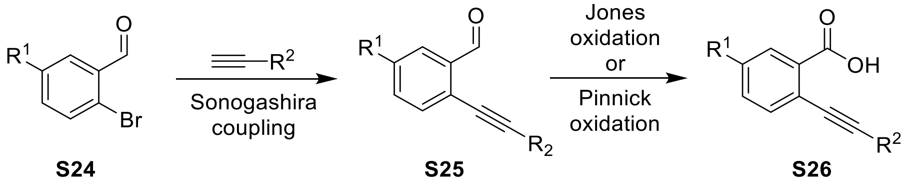

The aldehydes **S25** were prepared from various bromobenzaldehydes by the Sonogashira couplings. The carboxylic acids, corresponding to substrates **4f-4k**, **4m** and **4n**, were prepared following the general procedure of Jones oxidation. Carboxylic acid corresponding to **4o** was synthesized by the Pinnick oxidation.[9]

2.1 Preparation of *O*-acylhydroxamates.

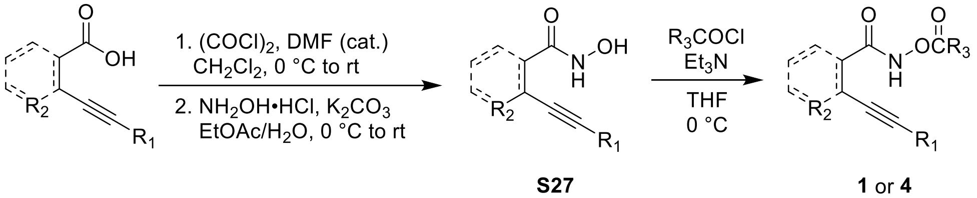

Hydroxamic acids **S27** were prepared from the prepared carboxylic acids according to reported procedure.[11]

To the hydroxamic acids **S27** (1.5 mmol) in THF (10 mL), Et_3N (1.43 mmol, 0.95 equiv) was added under 0 °C and acyl chloride (1.43 mmol, 0.95 equiv) was introduced subsequently. The reaction was monitored by TLC. Once the acyl chloride was consumed (approx. 5 min), water (10 mL) was poured into the reaction mixture. The water layer was extracted with EtOAc and the combined organic layers were washed with brine, dried over Na_2SO_4 and concentrated *in vacuo*. Chromatography (SiO_2) was performed to obtain the desired *O*-acylhydroxamates **1** or **4** in 62–94% yields.

2.2 General procedure for the carboxyamidation reactions.

A Schlenk tube was charged with *O*-acylhydroxamates **1** or **4** (0.15 mmol) in freshly distilled acetonitrile (3 mL). $FeCl_3$ (0.0075 mmol, 5 mol %) was then added and the mixture was degassed using freeze-pump-thaw techniques. The reaction was heated at 70 ℃ and monitored by TLC until completion. The resulting mixture was filtered through a short pad of celite and concentrated *in vacuo*. The crude material was then dissolved in dichloromethane (6 mL) and washed with saturated $NaHCO_3$ solution (3 mL). The organic layers were dried over anhydrous Na_2SO_4, filtered, and concentrated *in vacuo*. The pure product was obtained by chromatography (SiO_2) in 58–90% yields.

2.3 General procedure for the acyl group migration and the one-pot protocol.

To the carboxyamidation product **2** or **5** (0.1 mmol) in 2 mL of CH_2Cl_2 was added DMAP (30 mol %, 0.03 mmol). The reaction mixture was heated at 35–40 ℃ and monitored by TLC until completion. The resulting mixture was diluted with CH_2Cl_2 (3 mL) and washed with saturated $NaHCO_3$ solution (3 mL). The organic layers were dried over anhydrous Na_2SO_4, filtered, and concentrated *in vacuo*. The crude material was purified by chromatography (SiO_2) to give products in 58–94% yields.

For the one-pot protocol, carboxyamidation was performed as described in section **2.3**. Upon completion (monitored by TLC), the reaction mixture was cooled down to room temperature, and DMAP was directly added into the solution. The reaction was then warmed up to 35–40 ℃ and kept until completion (TLC monitoring). Identical workup and purification were performed.

2.4 Procedure for haloamidation and acetate exchange reactions.

Haloamidation: A Schlenk tube was charged with *O*-acylhydroxamates **S3** (0.15 mmol) in freshly distilled acetonitrile (3 mL). Iron catalyst $FeCl_3$ or $FeBr_2$ (0.0075 mmol, 5 mol %) and the corresponding Bu_4NCl or Bu_4NBr (0.45 mmol, 3 equiv) were then added, and the mixture was degassed using standard Schlenk techniques. The reaction was heated at 100 ℃ and monitored by TLC until completion. The resulting mixture was filtered through a short pad of celite and concentrated *in vacuo*. The crude material was then dissolved in dichloromethane (6 mL) and washed with sat'd $NaHCO_3$ solution (3 mL). The organic layers were dried over anhydrous Na_2SO_4, filtered, and concentrated *in vacuo*. The pure product was obtained by chromatography (SiO_2) in 45–82% yields.

Acetate exchange: The reaction follows the same procedure described above, except changing halogenation reagent Bu_4NCl or Bu_4NBr to Bu_4NOAc. The exchanged product **5b** (9%) was formed along with 55% of the aniline derivative **5a''**, which is likely a product of base-induced Losson rearrangement (see the mechanism below).

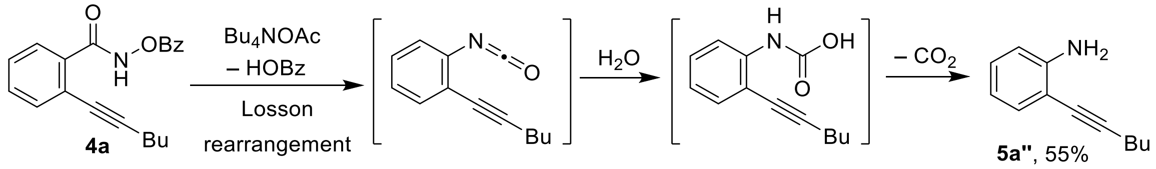

2.5 Evidence for *E*/*Z* isomerization of 2i, 2j, and 2p.

Substrate **1j** was subjected to carboxyamidation condition described above. One portion of reaction mixture was taken out after 2 hours, and the rest of materials was kept in reaction for another 1 hour. The crude NMR below demonstrated that variable reaction times correlate with a mixture of products with different ratios (after 2 hours, *Z* : *E* = 5 : 1, and after 3 hours, *Z* : *E* = 3.3 : 1). This indicates that the *cis*-selectivity of this reaction for alkyl-tethered substrate was deteriorated in the original reaction condition. This behavior has not been observed in other types of products.

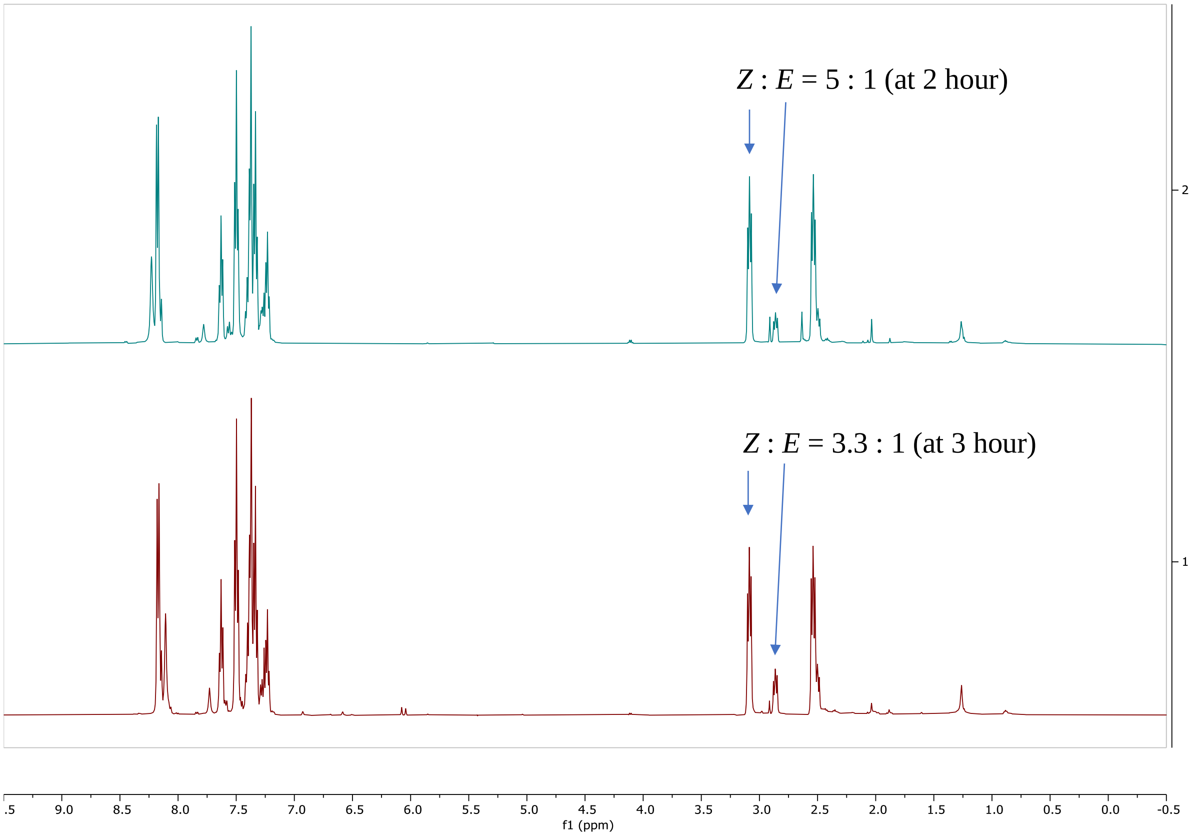

2.6 Plausible mechanistic pathways for the formation of 5o'.

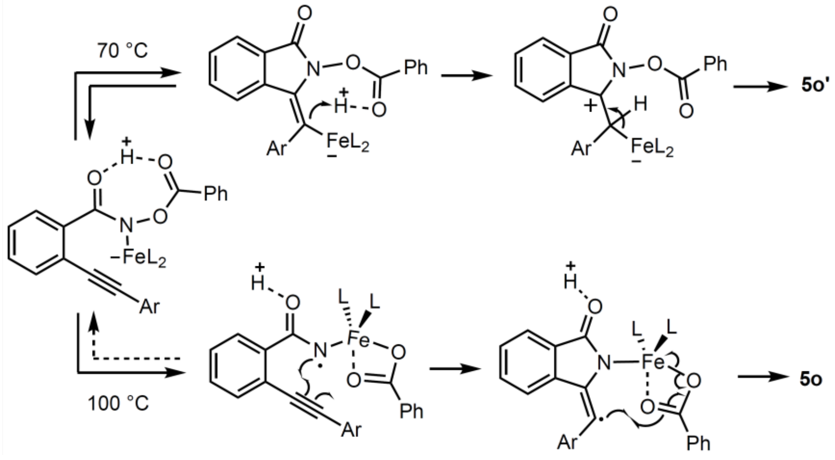

It is believed that the electron-withdrawing group (Ar = *p*-CF_3-C_6H_4) lowers the activation barrier for the direct nucleophilic addition of the Fe-amide complex. Thus, this reaction becomes favorable at 70 °C with a pre-equilibrium between the initial cyclization and ring-opening. On the other hand, at 100 °C, a radical-based reaction pathway becomes more favorable than the nucleophilic addition pathway. However, the electron-withdrawing group may increase the activation barrier for the radical process, compared to the other substrates, because of the electron-deficiency of the Fe-nitrenoid.

3. Characterization Data of Substrates

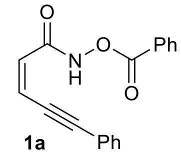

1a

Light-yellow solid (295 mg, 92%); **^{1}H NMR** (500 MHz, $CDCl_3$) δ 10.87 (brs, 1H), δ 8.13 (d, *J* = 7.9 Hz, 2H), 7.68–7.54 (m, 3H), 7.47 (t, *J* = 7.8 Hz, 2H), 7.42–7.32 (m, 3H), 6.39 (d, *J* = 11.9 Hz, 1H), 6.25 (d, *J* = 12.0 Hz, 1H); **^{13}C NMR** (125 MHz, $CDCl_3$) δ 164.48, 162.44, 134.27, 132.26, 130.07, 129.81, 128.95, 128.74, 128.57, 126.58, 121.59, 119.13, 102.50, 85.56; **HRMS** (ESI) calcd for $C_{18}H_{14}NO_3$ $[M+H]^+$ 292.0974, found 292.0974.

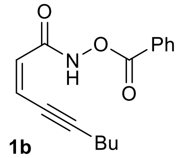

1b

White solid (286 mg, 90%); **^{1}H NMR** (500 MHz, $CDCl_3$) δ 11.17 (brs, 1H), 8.12 (dd, *J* = 8.4, 1.3 Hz, 2H), 7.62 (t, *J* = 7.5 Hz, 1H), 7.47 (t, *J* = 7.8 Hz, 2H), 6.16 (dt, *J* = 12.0, 2.2 Hz, 1H), 6.12 (d, *J* = 12.0 Hz, 1H), 2.54 (td, *J* = 7.1, 2.2 Hz, 2H), 1.60 (p, *J* = 7.2 Hz, 2H), 1.44 (h, *J* = 7.3 Hz, 2H), 0.91 (t, *J* = 7.3 Hz, 3H); **^{13}C NMR** (125 MHz, $CDCl_3$) δ 164.26, 162.30, 134.20, 129.98, 129.42, 128.72, 126.61, 119.15, 105.79, 30.14, 22.06, 19.66, 13.54; **HRMS** (ESI) calcd for $C_{16}H_{18}NO_3$ $[M+H]^+$ 272.1287, found 272.1285.

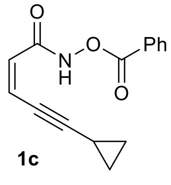

1c

Light-yellow solid (205 mg, 88%); **^{1}H NMR** (500 MHz, $CDCl_3$) δ 11.03 (brs, 1H), 8.08 (d, *J* = 7.0 Hz, 2H), 7.58 (t, *J* = 7.5 Hz, 1H), 7.43 (t, *J* = 7.9 Hz, 2H), 6.09 (s, 2H), 1.66–1.41 (m, 1H), 0.98–0.85 (m, 4H); 13C NMR (125 MHz, $CDCl_3$) δ 164.30, 162.43, 134.18, 129.97, 128.69, 128.40, 126.60, 119.65, 109.10, 73.20, 9.51, 0.77; **HRMS** (ESI) calcd for $C_{15}H_{14}NO_3$ $[M+H]^+$ 256.0974, found 256.0974.

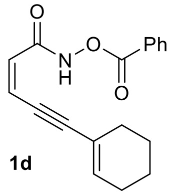

1d

Yellow solid (240 mg, 81%); **^{1}H NMR** (500 MHz, $CDCl_3$) δ 10.81 (brs, 1H), 8.09 (d, *J* = 8.3 Hz, 3H), 7.59 (t, *J* = 7.5 Hz, 2H), 7.44 (t, *J* = 7.8 Hz, 2H), 6.36 (s, 1H), 6.26 (d, *J* = 11.9 Hz, 1H), 6.12 (d, *J* = 11.9 Hz, 1H), 2.18 (s, 2H), 2.12 (s, 2H), 1.66–1.59 (m, 2H), 1.59–1.52 (m, 2H); **^{13}C NMR** (125 MHz, $CDCl_3$) δ 164.39, 162.74, 139.39, 134.16, 130.01, 128.68, 127.99, 126.67, 120.02, 119.48, 104.90, 83.57, 28.52, 26.01, 22.07, 21.22; **HRMS** (ESI) calcd for $C_{18}H_{18}NO_3$ $[M+H]^+$ 296.1287, found 296.1290.

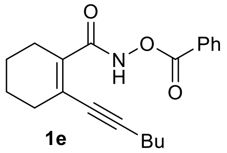

1e

Colorless oil (206 mg, 62%); **^{1}H NMR** (500 MHz, $CDCl_3$) δ 11.26 (s, 1H), 8.10 (d, *J* = 7.3 Hz, 2H), 7.58 (t, *J* = 7.5 Hz, 1H), 7.44 (t, *J* = 7.8 Hz, 2H), 2.52–2.39 (m, 4H), 2.34–2.25 (m, 2H), 1.66–1.58 (m, 4H), 1.53 (p, *J* = 7.1 Hz, 2H), 1.39 (h, *J* = 7.3 Hz, 2H), 0.86 (t, *J* = 7.3 Hz, 3H); **^{13}C NMR** (125 MHz, $CDCl_3$) δ 165.85, 164.42, 134.18, 133.99, 129.91, 128.63, 126.96, 125.36, 100.50, 80.31, 32.26, 30.35, 25.87, 22.01, 21.76, 21.59, 19.40, 13.55; **HRMS** (ESI) calcd for $C_{20}H_{24}NO_3$ $[M+H]^+$ 326.1756, found 326.1746.

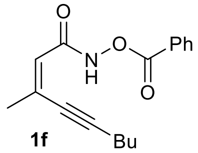

1f

White solid (255 mg, 80%); **^{1}H NMR** (500 MHz, $CDCl_3$) δ 11.06 (brs, 1H), 8.07 (d, *J* = 7.6 Hz, 2H), 7.56 (t, *J* = 7.5 Hz, 1H), 7.41 (t, *J* = 7.7 Hz, 2H), 5.98 (s, 1H), 2.48 (t, *J* = 7.2 Hz, 2H), 1.99 (s, 3H), 1.56 (p, *J* = 7.3 Hz, 2H), 1.40 (h, *J* = 7.4 Hz, 2H), 0.87 (t, *J* = 7.3 Hz, 3H); **^{13}C NMR** (125 MHz, $CDCl_3$) δ 164.40, 162.98, 134.05, 131.11, 129.91, 128.63, 126.78, 125.08, 104.37, 79.60, 30.19, 25.54, 22.02, 19.53, 13.52; **HRMS** (ESI) calcd for $C_{17}H_{20}NO_3$ $[M+H]^+$ 286.1443, found 286.1443.

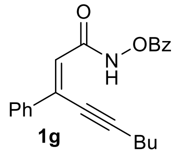
1g

Light-yellow solid (274 mg, 78%); **^{1}H NMR** (500 MHz, $CDCl_3$) δ 11.42 (brs, 1H), δ 8.13 (d, *J* = 7.7 Hz, 2H), 7.70–7.64 (m, 2H), 7.60 (t, *J* = 7.4 Hz, 1H), 7.45 (t, *J* = 7.6 Hz, 2H), 7.39–7.34 (m, 3H), 6.62 (s, 1H), 2.64 (t, *J* = 7.2 Hz, 2H), 1.67 (p, *J* = 7.3 Hz, 2H), 1.49 (h, *J* = 7.4 Hz, 2H), 0.93 (t, *J* = 7.4 Hz, 3H); **^{13}C NMR** (125 MHz, $CDCl_3$) δ 164.43, 163.14, 136.99, 134.15, 132.85, 130.00, 129.90, 128.71, 128.67, 127.09, 126.73, 123.47, 106.27, 78.31, 30.28, 22.20, 19.77, 13.61; **HRMS** (ESI) calcd for $C_{22}H_{22}NO_3$ $[M+H]^+$ 348.1600, found 348.1596.

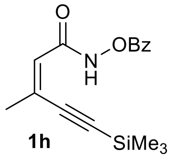
1h

White solid (293 mg, 88%); **^{1}H NMR** (500 MHz, $CDCl_3$) δ 11.03 (brs, 1H), 8.13 (d, *J* = 8.0 Hz, 2H), 7.62 (t, *J* = 7.5 Hz, 1H), 7.47 (t, *J* = 7.9 Hz, 2H), 6.08 (s, 1H), 2.07 (s, 3H), 0.26 (s, 9H); **^{13}C NMR** (125 MHz, $CDCl_3$) δ 164.31, 162.58, 134.12, 130.00, 129.42, 128.69, 127.69, 126.72, 108.38, 102.71, 24.94, -0.52; **HRMS** (ESI) calcd for $C_{16}H_{20}NO_3Si$ $[M+H]^+$ 302.1212, found 302.1205.

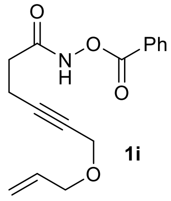
1i

Light-yellow solid (198 mg, 82%); **^{1}H NMR** (500 MHz, $CDCl_3$) δ 10.18 (brs, 1H), 8.00 (d, *J* = 7.6 Hz, 2H), 7.56 (t, *J* = 7.4 Hz, 1H), 7.40 (t, *J* = 7.6 Hz, 2H), 5.86 (ddt, *J* = 16.4, 10.8, 5.7 Hz, 1H), 5.26 (d, *J* = 17.2 Hz, 1H), 5.16 (d, *J* = 10.4 Hz, 1H), 4.10 (s, 2H), 4.01 (d, *J* = 5.7 Hz, 2H), 2.61–2.54 (m, 2H), 2.54–2.47 (m, 2H); **^{13}C NMR** (125 MHz, $CDCl_3$) δ 169.32, 164.57, 134.18, 133.97, 129.95, 128.69, 126.59, 117.91, 84.78, 77.22, 70.55, 57.61, 32.10, 14.81; **HRMS** (ESI) calcd for $C_{16}H_{18}NO_4$ $[M+H]^+$ 288.1236, found 288.1230.

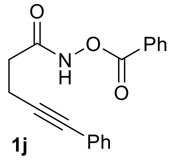
1j

White solid (187 mg, 80%); **^{1}H NMR** (500 MHz, $CDCl_3$) δ 9.99 (brs, 1H), 8.06 (d, *J* = 8.2 Hz, 2H), 7.58 (t, *J* = 7.5 Hz, 1H), 7.46–7.38 (m, 4H), 7.30–7.22 (m, 3H), 2.80 (t, *J* = 7.3 Hz, 2H), 2.64 (t, *J* = 7.6 Hz, 2H); **^{13}C NMR** (125 MHz, $CDCl_3$) δ 169.48, 164.73, 134.27, 131.71, 130.02, 128.73, 128.26, 127.95, 126.53, 123.35, 87.73, 81.90, 32.48, 15.48; **HRMS** (ESI) calcd for $C_{18}H_{16}NO_3$ $[M+H]^+$ 294.1130, found 294.1127.

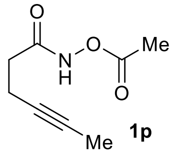
1p

White solid (190 mg, 82%); **^{1}H NMR** (500 MHz, $CDCl_3$) δ 9.57 (s, 1H), 2.54–2.48 (m, 2H), 2.49–2.41 (m, 2H), 2.24 (s, 3H), 1.81–1.76 (m, 3H); **^{13}C NMR** (125 MHz, $CDCl_3$) δ 169.58, 168.61, 32.93, 32.92, 18.31, 14.76, 3.45; **HRMS** (ESI) calcd for $C_8H_{12}NO_3$ $[M+H]^+$ 170.08117, found 170.08193.

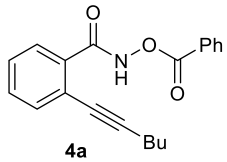
4a

Light-yellow solid (268 mg, 90%); **^{1}H NMR** (500 MHz, $CDCl_3$) δ 11.74 (brs, 1H), δ 8.17 (d, *J* = 7.7 Hz, 2H), 8.10 (d, *J* = 7.8 Hz, 1H), 7.63 (t, *J* = 7.3 Hz, 1H), 7.53–7.47 (m, 3H), 7.45–7.33 (m, 2H), 2.58 (t, *J* = 6.8 Hz, 2H), 1.64 (p, *J* = 7.0 Hz, 2H), 1.48 (h, *J* = 7.1 Hz, 2H), 0.93 (t, *J* = 7.4 Hz, 3H); **^{13}C NMR** (125 MHz, $CDCl_3$) δ 164.29, 163.70, 134.18, 133.62, 132.05, 131.54, 130.45, 130.03, 128.72, 128.28, 126.74, 120.85, 98.99, 79.37, 30.35, 22.11, 19.44, 13.62; **HRMS** (ESI) calcd for $C_{20}H_{20}NO_3$ $[M+H]^+$ 322.1443, found 322.1441.

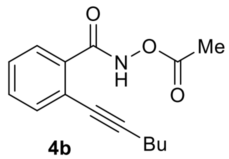

4b

White solid (176 mg, 78%); **^{1}H NMR** (500 MHz, $CDCl_3$) δ 11.40 (brs, 1H), 7.98 (d, *J* = 6.9 Hz, 1H), 7.42 (d, *J* = 7.6 Hz, 1H), 7.37 (td, *J* = 7.5, 1.7 Hz, 1H), 7.32 (td, *J* = 7.6, 1.6 Hz, 1H), 2.47 (t, *J* = 7.1 Hz, 2H), 2.23 (s, 3H), 1.56 (p, *J* = 7.2 Hz, 2H), 1.42 (h, *J* = 7.3 Hz, 2H), 0.89 (t, *J* = 7.3 Hz, 3H); **^{13}C NMR** (125 MHz, $CDCl_3$) δ 168.08, 163.56, 133.55, 131.90, 131.45, 130.27, 128.17, 120.77, 98.70, 79.21, 30.28, 22.03, 19.32, 18.36, 13.56; **HRMS** (ESI) calcd for $C_{15}H_{18}NO_3$ $[M+H]^+$ 260.1287, found 260.1286.

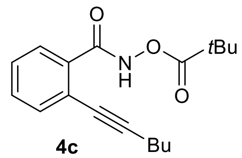

4c

Light-yellow solid (201 mg, 75%); **^{1}H NMR** (500 MHz, $CDCl_3$) δ 11.28 (brs, 1H), 8.03 (d, *J* = 7.5 Hz, 1H), 7.46 (d, *J* = 7.1 Hz, 1H), 7.43–7.34 (m, 2H), 2.52 (t, *J* = 7.1 Hz, 2H), 1.60 (p, *J* = 7.2 Hz, 2H), 1.45 (h, *J* = 7.3 Hz, 2H), 1.35 (s, 9H), 0.93 (t, *J* = 7.3 Hz, 3H); 13C NMR (125 MHz, $CDCl_3$) δ 175.91, 163.90, 133.52, 132.13, 131.40, 130.32, 128.22, 120.74, 98.62, 79.37, 38.44, 30.30, 27.07, 22.04, 19.35, 13.60. **HRMS** (ESI) calcd for $C_{18}H_{24}NO_3$ $[M+H]^+$ 302.1756, found 302.1752.

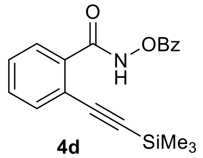

4d

Colorless oil (175 mg, 83%); **^{1}H NMR** (500 MHz, $CDCl_3$) δ 11.51 (brs, 1H), 8.11 (d, *J* = 7.8 Hz, 2H), 8.03 (d, *J* = 7.7 Hz, 1H), 7.58 (t, *J* = 7.5 Hz, 1H), 7.51 (dd, *J* = 7.5, 1.6 Hz, 1H), 7.43 (t, *J* = 8.0 Hz, 2H), 7.41–7.35 (m, 2H), 0.27 (s, 9H); **^{13}C NMR** (125 MHz, $CDCl_3$) δ 164.22, 163.77, 134.18, 133.74, 132.76, 131.51, 130.39, 129.99, 129.22, 128.73, 126.71, 119.91, 103.23, 102.74, -0.39. **HRMS** (ESI) calcd for $C_{19}H_{20}NO_3Si$ $[M+H]^+$ 338.1212, found 338.1210.

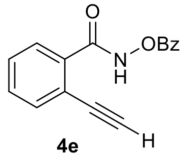

4e

White solid (195 mg, 75%); **^{1}H NMR** (500 MHz, $CDCl_3$) δ 11.22 (brs, 1H), 8.09 (d, *J* = 7.3 Hz, 2H), 8.02–7.90 (m, 1H), 7.59 (t, *J* = 7.5 Hz, 1H), 7.53 (dd, *J* = 6.3, 2.6 Hz, 1H), 7.47–7.36 (m, 4H), 3.63 (s, 1H); **^{13}C NMR** (125 MHz, $CDCl_3$) δ 164.47, 163.78, 134.24, 134.05, 133.41, 131.49, 130.08, 130.04, 129.43, 128.72, 126.57, 119.18, 84.50, 81.84; **HRMS** (ESI) calcd for $C_{16}H_{12}NO_3$ $[M+H]^+$ 266.0817, found 266.0814.

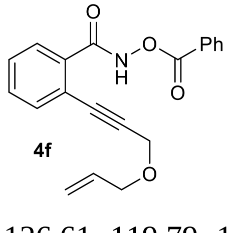

4f

White solid (189 mg, 81%); **^{1}H NMR** (500 MHz, $CDCl_3$) δ 11.29 (brs, 1H), 8.08 (d, *J* = 7.4 Hz, 2H), 7.94 (d, *J* = 7.2 Hz, 1H), 7.58 (t, *J* = 7.5 Hz, 1H), 7.49 (dd, *J* = 7.4, 1.7 Hz, 1H), 7.46–7.32 (m, 4H), 5.87 (ddt, *J* = 17.1, 10.2, 5.7 Hz, 1H), 5.29 (dd, *J* = 17.2, 1.6 Hz, 1H), 5.15 (dd, *J* = 10.4, 1.4 Hz, 1H), 4.46 (s, 2H), 4.11 (dt, *J* = 5.7, 1.4 Hz, 2H); **^{13}C NMR** (125 MHz, $CDCl_3$) δ 164.35, 163.88, 134.21, 133.90, 133.60, 132.99, 131.42, 130.00, 129.03, 128.70, 126.61, 119.79, 117.97, 92.56, 84.20, 70.95, 57.95; **HRMS** (ESI) calcd for $C_{20}H_{18}NO_4$ $[M+H]^+$ 336.1236, found 336.1228.

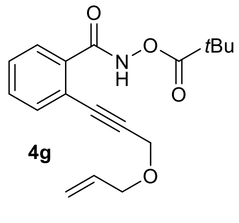

4g

Colorless oil (178 mg, 72%); **^{1}H NMR** (500 MHz, $CDCl_3$) δ 10.86 (brs, 1H), 7.93 (d, *J* = 7.5 Hz, 1H), 7.52–7.46 (m, 1H), 7.45–7.35 (m, 2H), 5.89 (ddt, *J* = 17.3, 10.4, 5.8 Hz, 1H), 5.31 (dd, *J* = 17.2, 1.6 Hz, 1H), 5.19 (dd, *J* = 10.4, 1.5 Hz, 1H), 4.43 (s, 2H), 4.09 (dd, *J* = 5.8, 1.4 Hz, 2H), 1.31 (s, 9H); **^{13}C NMR** (125 MHz, $CDCl_3$) δ 176.01, 163.89, 133.87, 133.57, 132.96, 131.38, 130.05, 129.06, 119.59, 118.06, 92.43, 84.21, 70.92, 57.86, 38.41, 27.04; **HRMS** (ESI) calcd for $C_{19}H_{22}NO_4$ $[M+H]^+$ 328.1549, found 328.1544.

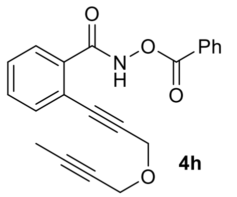

4h

White solid (192 mg, 82%); **^{1}H NMR** (500 MHz, $CDCl_3$) δ 11.25 (brs, 1H), 8.12 (d, *J* = 7.0 Hz, 2H), 7.99 (dd, *J* = 7.2, 2.0 Hz, 1H), 7.61 (t, *J* = 7.4 Hz, 1H), 7.53 (dd, *J* = 7.5, 1.6 Hz, 1H), 7.49–7.37 (m, 4H), 4.57 (s, 2H), 4.27 (q, *J* = 2.3 Hz, 2H), 1.77 (t, *J* = 2.4 Hz, 3H); **^{13}C NMR** (125 MHz, $CDCl_3$) δ 164.39, 163.76, 134.22, 133.68, 132.91, 131.50, 130.20, 130.04, 129.17, 128.71, 126.62, 119.58, 92.19, 84.51, 83.49, 74.29, 57.64, 57.19, 3.58; **HRMS** (ESI) calcd for $C_{21}H_{18}NO_4$ $[M+H]^+$ 348.1236, found 348.1231.

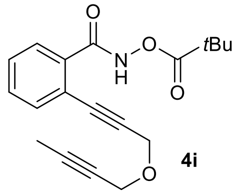

4i

White solid (185 mg, 73%); **^{1}H NMR** (500 MHz, $CDCl_3$) δ 10.78 (brs, 1H), 7.89 (d, *J* = 7.3 Hz, 1H), 7.47 (dd, *J* = 7.4, 1.6 Hz, 1H), 7.43–7.34 (m, 2H), 4.49 (s, 2H), 4.21 (q, *J* = 2.3 Hz, 2H), 1.80 (t, *J* = 2.4 Hz, 3H), 1.30 (s, 9H); **^{13}C NMR** (125 MHz, $CDCl_3$) δ 176.01, 163.91, 133.56, 132.98, 131.36, 130.00, 129.08, 119.52, 91.88, 84.40, 83.40, 74.30, 57.58, 57.14, 38.40, 27.01, 3.61; **HRMS** (ESI) calcd for $C_{19}H_{22}NO_4$ $[M+H]^+$ 328.1549, found 328.1544.

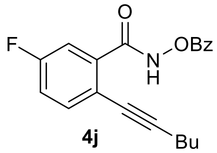

4j

White solid (262 mg, 82%); **^{1}H NMR** (500 MHz, $CDCl_3$) δ 11.85 (brs, 1H), 8.14 (d, *J* = 7.4 Hz, 2H), 7.80 (dd, *J* = 9.5, 2.8 Hz, 1H), 7.62 (t, *J* = 7.5 Hz, 1H), 7.53–7.40 (m, 3H), 7.13 (td, *J* = 8.1, 2.8 Hz, 1H), 2.56 (t, *J* = 7.1 Hz, 2H), 1.63 (p, *J* = 7.2 Hz, 2H), 1.47 (h, *J* = 7.4 Hz, 2H), 0.92 (t, *J* = 7.4 Hz, 3H). **^{13}C NMR** (125 MHz, $CDCl_3$) δ 164.12, 162.04 (d, *J* = 250.7 Hz), 162.00, 135.68 (d, *J* = 7.8 Hz), 134.33 (d, *J* = 8.6 Hz), 134.25, 130.00, 128.74, 126.55, 118.97 (d, *J* = 22.0 Hz), 117.37 (d, *J* = 24.5 Hz), 116.96 (d, *J* = 3.7 Hz), 98.84, 78.42, 30.29, 22.10, 19.37, 13.58. **HRMS** (ESI) calcd for $C_{20}H_{19}NO_3F$ $[M+H]^+$ 340.1349, found 340.1341.

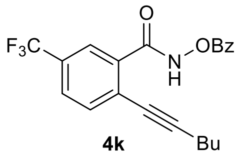

4k

Light-yellow oil (201 mg, 80%); **^{1}H NMR** (500 MHz, $CDCl_3$) δ 11.77 (brs, 1H), 8.35 (s, 1H), 8.14 (d, *J* = 7.2 Hz, 2H), 7.67–7.56 (m, 3H), 7.47 (t, *J* = 7.7 Hz, 2H), 2.59 (t, *J* = 7.2 Hz, 2H), 1.65 (p, *J* = 7.3 Hz, 2H), 1.47 (h, *J* = 7.4 Hz, 2H), 0.92 (t, *J* = 7.4 Hz, 3H); **^{13}C NMR** (125 MHz, $CDCl_3$) δ 164.14, 162.02, 134.30, 134.18, 132.86, 130.18 (q, *J* = 33.6 Hz), 130.00, 128.75, 127.85 (q, J = 3.6 Hz), 127.47 (d, *J* = 4.3 Hz), 126.44, 124.54, 123.42 (q, *J* = 272.7 Hz), 101.97, 78.37, 30.13, 22.10, 19.48, 13.52; **HRMS** (ESI) calcd for $C_{21}H_{19}NO_3F_3$ $[M+H]^+$ 390.1317, found 390.1322.

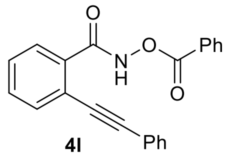

4l

White solid (258 mg, 94%); **^{1}H NMR** (500 MHz, $CDCl_3$) δ 11.22 (brs, 1H), 8.14 (d, *J* = 7.7 Hz, 2H), 8.03 (d, *J* = 7.7 Hz, 1H), 7.66–7.57 (m, 4H), 7.46 (t, *J* = 6.9 Hz, 3H), 7.40 (t, *J* = 7.7 Hz, 1H), 7.37–7.31 (m, 3H); **^{13}C NMR** (125 MHz, $CDCl_3$) δ 164.49, 134.22, 133.64, 132.46, 131.95, 131.57, 130.25, 130.11, 129.23, 128.85, 128.73, 128.52, 126.71, 122.07, 120.50, 96.27, 87.30; **HRMS** (ESI) calcd for $C_{22}H_{16}NO_3$ $[M+H]^+$ 342.1130, found 342.1130.

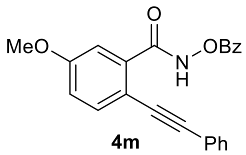

4m

White solid (260 mg, 88%); **^{1}H NMR** (500 MHz, $CDCl_3$) δ 11.33 (brs, 1H), 8.16 (d, *J* = 7.7 Hz, 2H), 7.66–7.58 (m, 4H), 7.54 (d, *J* = 8.6 Hz, 1H), 7.48 (t, *J* = 7.7 Hz, 2H), 7.39–7.32 (m, 3H), 7.01 (dd, *J* = 8.7, 2.7 Hz, 1H), 3.83 (s, 3H); **^{13}C NMR** (125 MHz, $CDCl_3$) δ 164.40, 163.88, 159.91, 135.20, 134.21, 133.76, 131.74, 130.11, 128.92, 128.73, 128.48, 126.72, 122.30, 118.62,

114.56, 112.32, 95.03, 87.36, 55.61; **HRMS** (ESI) calcd for $C_{23}H_{18}NO_4$ [M+H]$^+$ 372.1236, found 372.1230.

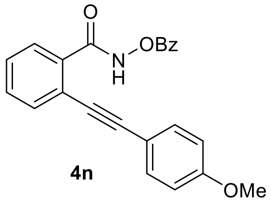

4n

White solid (205 mg, 82%); ^{1}**H NMR** (500 MHz, $CDCl_3$) δ 11.28 (brs, 1H), 8.16 (d, *J* = 8.1 Hz, 2H), 8.08 (d, *J* = 7.9 Hz, 1H), 7.65–7.59 (m, 2H), 7.57 (d, *J* = 8.9 Hz, 2H), 7.48 (t, *J* = 8.0 Hz, 3H), 7.41 (td, *J* = 7.5, 1.3 Hz, 1H), 6.88 (d, *J* = 8.9 Hz, 2H), 3.80 (s, 3H); ^{13}**C NMR** (125 MHz, $CDCl_3$) δ 164.48, 164.28, 160.40, 134.19, 133.51, 133.48, 132.01, 131.58, 130.40, 130.10, 128.72, 128.53, 126.77, 120.73, 114.20, 113.96, 96.77, 86.28, 55.34; **HRMS** (ESI) calcd for $C_{23}H_{18}NO_4$ [M+H]$^+$ 372.1236, found 372.1230.

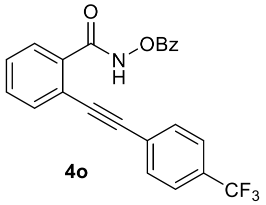

4o

White solid (270 mg, 80%); ^{1}**H NMR** (500 MHz, $CDCl_3$) δ 11.04 (brs, 1H), 8.18 (d, *J* = 7.3 Hz, 2H), 8.04 (d, *J* = 7.6 Hz, 1H), 7.77 (d, *J* = 8.0 Hz, 2H), 7.71–7.62 (m, 4H), 7.58–7.46 (m, 4H); ^{13}**C NMR** (125 MHz, $CDCl_3$) δ 164.55, 164.18, 134.33, 133.75, 132.90, 132.21, 131.56, 130.67 (q, *J* = 32.5 Hz), 130.15, 130.07, 129.33, 128.76, 126.52, 125.92, 125.36 (q, *J* = 3.8 Hz), 123.85 (q, *J* = 272.2 Hz), 119.90, 94.35, 89.19; **HRMS** (ESI) calcd for $C_{23}H_{15}NO_3F_3$ [M+H]$^+$ 410.1004, found 410.0995.

4. Characterization Data of Products

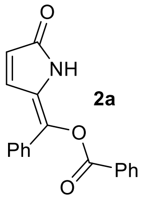

2a

White solid (37.1 mg, 85%); ^{1}**H NMR** (500 MHz, $CDCl_3$) δ 8.39 (brs, 1H), 8.17 (d, *J* = 7.0 Hz, 2H), 7.66 (t, *J* = 7.5 Hz, 1H), 7.52 (t, *J* = 7.3 Hz, 4H), 7.44–7.38 (m, 3H), 7.29 (d, *J* = 4.0 Hz, 1H), 6.15 (d, *J* = 4.1 Hz, 1H); ^{13}**C NMR** (125 MHz, $CDCl_3$) δ 170.68, 164.10, 136.03, 134.53, 134.20, 132.78, 130.43, 130.12, 129.64, 128.86, 128.77, 128.45, 128.13, 125.77; **HRMS** (ESI) calcd for $C_{18}H_{14}NO_3$ [M+H]$^+$ 292.0974, found 292.0976.

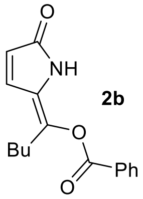

2b

White solid (33.4 mg, 82%); ^{1}**H NMR** (500 MHz, $CDCl_3$) δ 8.56 (brs, 1H), 8.17 (d, *J* = 7.2 Hz, 2H), 7.67 (t, *J* = 7.5 Hz, 1H), 7.53 (t, *J* = 7.7 Hz, 2H), 7.27 (d, *J* = 5.7 Hz, 1H), 6.07 (d, *J* = 5.5 Hz, 1H), 2.70 (t, *J* = 7.5 Hz, 2H), 1.57 (p, *J* = 7.5 Hz, 2H), 1.40 (h, *J* = 7.5 Hz, 2H), 0.94 (t, *J* = 7.4 Hz, 3H); ^{13}**C NMR** (125 MHz, $CDCl_3$) δ 171.08, 163.94, 137.46, 134.30, 133.97, 130.33, 129.45, 128.64, 124.58, 29.36, 22.15, 13.75; **HRMS** (ESI) calcd for $C_{16}H_{18}NO_3$ [M+H]$^+$ 272.1287, found 272.1290.

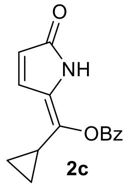

2c

Light-yellow solid (29.9 mg, 78%); ^{1}**H NMR** (500 MHz, $CDCl_3$) δ 8.23 (brs, 1H), 8.08 (d, *J* = 7.7 Hz, 2H), 7.64 (t, *J* = 7.6 Hz, 1H), 7.49 (t, *J* = 7.7 Hz, 2H), 7.40 (d, *J* = 4.0 Hz, 1H), 6.05 (d, *J* = 3.9 Hz, 1H), 2.11–2.00 (m, 1H), 0.94–0.85 (m, 2H), 0.84–0.77 (m, 2H); ^{13}**C NMR** (125 MHz, $CDCl_3$) δ 170.64, 163.88, 136.85, 134.57, 134.08, 130.30, 129.59, 128.70, 128.29, 124.24, 11.55, 6.16; **HRMS** (ESI) calcd for $C_{15}H_{14}NO_3$ [M+H]$^+$ 256.0974, found 256.0974.

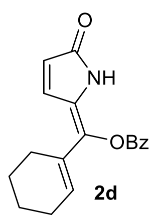

2d

Light-yellow solid (35.9 mg, 81%); **^{1}H NMR** (500 MHz, $CDCl_3$) δ 8.14 (d, *J* = 6.4 Hz, 2H), 8.01 (brs, 1H), 7.64 (t, *J* = 7.5 Hz, 1H), 7.50 (t, *J* = 7.8 Hz, 2H), 7.35 (dd, *J* = 5.8, 1.8 Hz, 1H), 6.15 (t, *J* = 4.2 Hz, 1H), 6.04 (dd, *J* = 5.8, 1.9 Hz, 1H), 2.26–2.18 (m, 4H), 1.73–1.66 (m, 2H), 1.66–1.58 (m, 2H); **^{13}C NMR** (125 MHz, $CDCl_3$) δ 170.59, 163.96, 137.01, 135.56, 134.08, 133.02, 130.34, 130.28, 129.01, 128.74, 128.44, 124.23, 26.29, 25.93, 22.18, 21.64; **HRMS** (ESI) calcd for $C_{18}H_{18}NO_3$ $[M+H]^+$ 296.1287, found 296.1287.

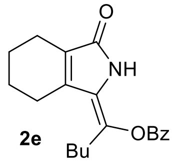

2e

White solid (28.3 mg, 58%); **^{1}H NMR** (500 MHz, $CDCl_3$) δ 8.11 (d, *J* = 7.9 Hz, 2H), 7.65 (t, *J* = 7.3 Hz, 1H), 7.50 (t, *J* = 7.7 Hz, 2H), 7.37 (s, 1H), 2.68 (t, *J* = 7.5 Hz, 2H), 2.63–2.55 (m, 2H), 2.30–2.21 (m, 2H), 1.86–1.77 (m, 2H), 1.76–1.67 (m, 2H), 1.54 (p, *J* = 8.0, 7.6 Hz, 2H), 1.38 (dq, *J* = 14.8, 7.3 Hz, 2H), 0.91 (t, *J* = 7.3 Hz, 3H); **^{13}C NMR** (125 MHz, $CDCl_3$) δ 169.50, 164.16, 141.92, 135.59, 134.01, 133.50, 130.16, 128.74, 128.68, 29.67, 29.59, 24.66, 22.47, 22.41, 21.31, 20.52, 13.89; **HRMS** (ESI) calcd for $C_{20}H_{24}NO_3$ $[M+H]^+$ 326.1756, found 326.1748.

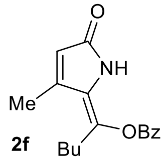

2f

Light-yellow oil (29.5 mg, 69%); **^{1}H NMR** (500 MHz, $CDCl_3$) δ 8.10 (d, *J* = 7.5 Hz, 2H), 7.65 (t, *J* = 7.4 Hz, 1H), 7.51 (q, *J* = 7.7, 6.7 Hz, 3H), 5.85 (s, 1H), 2.73 (t, *J* = 7.7 Hz, 2H), 2.31 (s, 3H), 1.56 (p, *J* = 7.6 Hz, 2H), 1.39 (h, *J* = 7.3 Hz, 2H), 0.91 (t, *J* = 7.3 Hz, 3H); **^{13}C NMR** (125 MHz, $CDCl_3$) δ 169.13, 163.99, 146.49, 137.43, 134.08, 130.20, 130.11, 128.74, 128.56, 124.14, 29.71, 29.55, 22.39, 16.32, 13.84; **HRMS** (ESI) calcd for $C_{17}H_{20}NO_3$ $[M+H]^+$ 286.1443, found 286.1441.

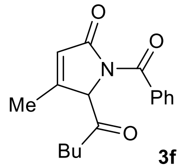

3f

Light-yellow oil (34.2 mg, 80%); **^{1}H NMR** (500 MHz, $CDCl_3$) δ 7.65 (d, *J* = 7.4 Hz, 2H), 7.54 (t, *J* = 7.4 Hz, 1H), 7.43 (t, *J* = 7.4 Hz, 2H), 5.97 (s, 1H), 5.27 (s, 1H), 2.58 (dt, *J* = 18.0, 7.3 Hz, 1H), 2.48 (dt, *J* = 17.9, 7.1 Hz, 1H), 2.09 (s, 3H), 1.60 (p, *J* = 7.4 Hz, 2H), 1.30 (h, *J* = 7.6 Hz, 2H), 0.89 (t, *J* = 7.4 Hz, 3H); **^{13}C NMR** (125 MHz, $CDCl_3$) δ 203.06, 168.84, 168.59, 157.42, 133.42, 132.20, 128.91, 127.85, 123.84, 73.21, 38.13, 25.15, 22.19, 15.00, 13.81; **HRMS** (ESI) calcd for $C_{17}H_{20}NO_3$ $[M+H]^+$ 286.1443, found 286.1440.

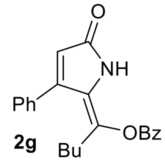

2g

Light-yellow oil (34.9 mg, 67%); **^{1}H NMR** (500 MHz, $CDCl_3$) δ 8.12 (d, *J* = 6.8 Hz, 2H), 7.93 (s, 1H), 7.66 (t, *J* = 7.4 Hz, 1H), 7.52 (t, *J* = 7.7 Hz, 2H), 7.47–7.42 (m, 3H), 7.41–7.37 (m, 2H), 6.04 (s, 1H), 2.23 (t, *J* = 7.7 Hz, 2H), 1.33 (p, *J* = 7.6 Hz, 2H), 1.04 (h, *J* = 7.5 Hz, 2H), 0.66 (t, *J* = 7.4 Hz, 3H); **^{13}C NMR** (125 MHz, $CDCl_3$) δ 168.71, 163.83, 150.57, 139.72, 134.12, 133.76, 130.24, 129.25, 129.10, 128.78, 128.52, 128.31, 124.53, 29.32, 29.20, 22.10, 13.51; **HRMS** (ESI) calcd for $C_{22}H_{22}NO_3$ $[M+H]^+$ 348.1600, found 348.1593.

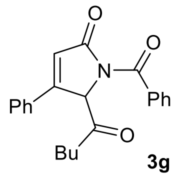

3g

Light-yellow solid (40.6 mg, 78%); **^{1}H NMR** (500 MHz, $CDCl_3$) δ 7.71–7.68 (m, 2H), 7.67 (dd, *J* = 7.9, 1.8 Hz, 2H), 7.59–7.54 (m, 1H), 7.52–7.43 (m, 5H), 6.46 (d, *J* = 1.3 Hz, 1H), 5.95 (d, *J* = 1.3 Hz, 1H), 2.53 (dt, *J* = 17.7, 7.3 Hz, 1H), 2.43 (dt, *J* = 17.7, 7.2 Hz, 1H), 1.45 (p, *J* = 7.3 Hz, 2H), 1.10 (hd, *J* = 7.2, 2.3 Hz, 2H), 0.76 (t, *J* = 7.4 Hz, 3H); **^{13}C NMR** (125 MHz, $CDCl_3$) δ 202.57, 169.07, 168.47, 157.17, 133.34, 132.34, 131.79, 129.78, 129.27, 129.05, 127.91, 127.67, 121.22, 70.77, 37.19, 25.20, 21.93, 13.67; **HRMS** (ESI) calcd for $C_{22}H_{22}NO_3$ $[M+H]^+$ 348.1600, found 348.1596.

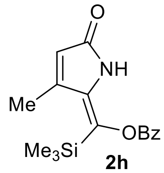

2h

Light-yellow oil (32.6 mg, 72%); **^{1}H NMR** (500 MHz, $CDCl_3$)δ 8.09 (d, *J* = 7.4 Hz, 2H), 7.64 (t, *J* = 6.8 Hz, 1H), 7.60–7.41 (m, 3H), 5.97 (s, 1H), 2.31 (s, 3H), 0.35 (s, 9H); **^{13}C NMR** (125 MHz, $CDCl_3$) δ 169.34, 165.47, 146.94, 142.14, 141.80, 133.82, 129.94, 128.73, 127.99, 124.63, 15.42, 0.19; **HRMS** (ESI) calcd for $C_{16}H_{20}NO_3Si$ $[M+H]^+$ 302.1212, found 302.1209.

2i-(*Z*)

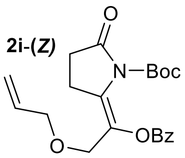

White solid (22.6 mg, 39%); **^{1}H NMR** (500 MHz, $CDCl_3$) δ 8.08 (d, *J* = 6.5 Hz, 1H), 7.57 (t, *J* = 7.4 Hz, 1H), 7.43 (t, *J* = 7.6 Hz, 2H), 5.87 (ddt, *J* = 16.4, 10.8, 5.6 Hz, 1H), 5.25 (dd, *J* = 17.2, 1.7 Hz, 1H), 5.16 (dd, *J* = 10.5, 1.5 Hz, 1H), 4.34 (s, 2H), 4.02 (d, *J* = 5.7 Hz, 2H), 2.88 (t, *J* = 7.9 Hz, 2H), 2.62 (t, *J* = 7.9 Hz, 2H), 1.23 (s, 9H); **^{13}C NMR** (125 MHz, $CDCl_3$) δ 173.29, 164.38, 148.49, 134.41, 133.44, 130.15, 129.56, 128.43, 128.36, 128.01, 117.66, 84.79, 71.12, 66.13, 30.63, 27.49, 22.86; **HRMS** (ESI) calcd for $C_{21}H_{25}NO_6Na$ $[M+Na]^+$ 410.1580, found 410.1570.

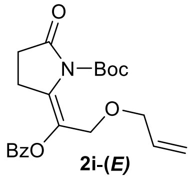

2i-(*E*)

White solid (11 mg, 19%); **^{1}H NMR** (500 MHz, $CDCl_3$) δ 8.10 (d, *J* = 7.2 Hz, 2H), 7.62 (t, *J* = 7.4 Hz, 1H), 7.48 (t, *J* = 7.7 Hz, 2H), 5.85 (ddt, *J* = 16.3, 10.7, 5.6 Hz, 1H), 5.23 (dd, *J* = 17.1, 1.8 Hz, 1H), 5.14 (d, *J* = 10.9 Hz, 1H), 4.19 (s, 2H), 3.97 (d, *J* = 6.0 Hz, 2H), 2.72 (t, *J* = 7.8 Hz, 2H), 2.56 (t, *J* = 7.9 Hz, 2H), 1.59 (s, 9H); **^{13}C NMR** (125 MHz, $CDCl_3$) δ 173.92, 164.67, 148.19, 134.33, 133.67, 131.10, 130.07, 129.72, 129.12, 128.61, 117.44, 84.51, 71.20, 67.25, 30.61, 27.92, 23.17; **HRMS** (ESI) calcd for $C_{21}H_{25}NO_6Na$ $[M+Na]^+$ 410.1580, found 410.1575.

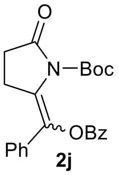

2j

White solid (44.8 mg, 76%); **^{1}H NMR** (500 MHz, $CDCl_3$); **(*Z*)-alkene**: δ 8.11 (d, *J* = 7.0 Hz, 2H), 7.58 (t, *J* = 7.4 Hz, 1H), 7.47–7.40 (m, 4H), 7.34 (t, *J* = 7.4 Hz, 2H), 7.31–7.27 (m, 1H), 2.93 (t, *J* = 7.8 Hz, 2H), 2.59 (t, *J* = 7.8 Hz, 2H), 1.24 (s, 9H); **(*E*)-alkene**: δ 8.15 (d, *J* = 7.1 Hz, 2H), 7.63 (t, *J* = 7.3 Hz, 1H), 7.49 (t, *J* = 7.7 Hz, 2H), 7.47–7.40 (m, 1H), 7.34 (m, 2H), 7.27–7.24 (m, 2H), 2.78 (t, *J* = 7.8 Hz, 2H), 2.68 (t, *J* = 7.8 Hz, 2H), 1.15 (s, 9H); **^{13}C NMR** (125 MHz, $CDCl_3$, as a mixture) δ 174.35, 173.06, 164.74, 163.92, 148.55, 147.20, 134.96, 134.41, 133.80, 133.47, 132.97, 130.17, 130.10, 129.71, 129.11, 128.87, 128.80, 128.69, 128.54, 128.45, 128.26, 127.16, 126.99, 126.31, 84.62, 84.38, 31.58, 31.35, 27.53, 27.30, 25.28, 24.29; **HRMS** (ESI) calcd for $C_{18}H_{16}NO_3$ $[M-Boc+H]^+$ 294.1130, found 294.1134.

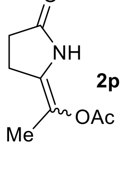

2p

White solid (18.8 mg, 74%); **^{1}H NMR** (500 MHz, $CDCl_3$); **(*Z*)-alkene**: δ 9.38 (s, 1H), 2.73–2.68 (m, 2H), 2.55–2.50 (m, 2H), 2.22 (s, 3H), 1.93 (t, *J* = 1.7 Hz, 3H); **(*E*)-alkene**: δ 9.45 (s, 1H), 2.64–2.57 (m, 2H), 2.49–2.42 (m, 2H), 2.14 (s, 3H), 1.83 (t, *J* = 1.4 Hz, 3H); **^{13}C NMR** (125 MHz, $CDCl_3$, as a mixture) δ 178.69, 177.77, 169.71, 169.52, 128.89, 126.20, 124.41, 122.07, 29.86, 29.54, 21.66, 21.61, 20.78, 20.67, 15.35, 14.80; **HRMS** (ESI) calcd for $C_8H_{12}NO_3$ $[M+H]^+$ 170.08117, found 170.08186.

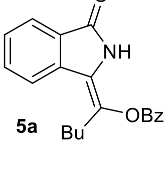

5a

White solid (39.5mg, 82%); **^{1}H NMR** (500 MHz, $CDCl_3$) δ 9.11 (brs, 1H), 8.24–8.17 (m, 2H), 7.74 (d, *J* = 7.9 Hz, 1H), 7.68 (t, *J* = 7.5 Hz, 1H), 7.60 (td, *J* = 7.6, 1.2 Hz, 1H), 7.54 (t, *J* = 7.9 Hz, 3H), 7.43 (t, *J* = 7.5 Hz, 1H), 2.91 (t, *J* = 7.7 Hz, 2H), 1.67 (p, *J* = 7.5 Hz, 2H), 1.47 (h, *J* = 7.3 Hz, 2H), 0.94 (t, *J* = 7.3 Hz, 3H); **^{13}C NMR** (125 MHz, $CDCl_3$) δ 167.18, 164.41, 135.27, 134.91, 133.79, 132.09, 130.84,

130.43, 129.08, 128.62, 128.41, 125.28, 123.99, 122.37, 30.39, 29.30, 22.47, 13.90; **HRMS** (ESI) calcd for $C_{20}H_{20}NO_3$ $[M+H]^+$ 322.1443, found 322.1443.

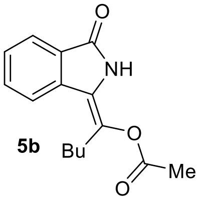

5b

Light-yellow solid (28 mg, 72%); **^{1}H NMR** (500 MHz, $CDCl_3$) δ 10.35 (d, *J* = 41.6 Hz, 1H), 7.83 (d, *J* = 7.6 Hz, 1H), 7.72 (d, *J* = 7.9 Hz, 1H), 7.60 (t, *J* = 7.7 Hz, 1H), 7.47 (t, *J* = 7.5 Hz, 1H), 2.81 (t, *J* = 7.7 Hz, 2H), 2.47 (s, 3H), 1.64 (p, *J* = 7.7 Hz, 2H), 1.45 (h, *J* = 7.4 Hz, 2H), 0.96 (t, *J* = 7.4 Hz, 3H); **^{13}C NMR** (125 MHz, $CDCl_3$) δ 169.32, 168.10, 135.62, 134.99, 132.06, 131.08, 128.35, 125.37, 123.76, 122.42, 30.46, 29.23, 22.47, 20.95, 13.90; **HRMS** (ESI) calcd for $C_{15}H_{18}NO_3$ $[M+H]^+$ 260.1287, found 260.1284.

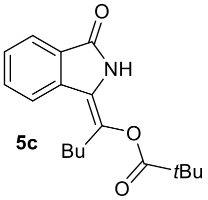

5c

White solid (29.4 mg, 65%); **^{1}H NMR** (500 MHz, $CDCl_3$) δ 7.88 (d, *J* = 7.6 Hz, 1H), 7.81 (brs, 1H), 7.72 (d, *J* = 8.0 Hz, 1H), 7.61 (t, *J* = 7.7 Hz, 1H), 7.49 (t, *J* = 7.1 Hz, 1H), 2.79 (t, *J* = 7.6 Hz, 2H), 1.61 (p, *J* = 7.3 Hz, 2H), 1.48–1.40 (m, 2H), 1.38 (s, 9H), 0.95 (t, *J* = 7.4 Hz, 3H); **^{13}C NMR** (125 MHz, $CDCl_3$) δ 176.20, 166.66, 135.32, 134.95, 132.24, 130.55, 128.52, 124.51, 124.11, 122.39, 39.41, 30.07, 29.21, 27.20, 22.40, 13.89; **HRMS** (ESI) calcd for $C_{18}H_{24}NO_3$ $[M+H]^+$ 302.1756, found 302.1756.

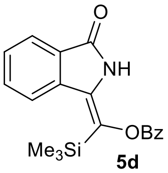

5d

Colorless oil (44.5 mg, 88%); **^{1}H NMR** (500 MHz, $CDCl_3$) δ 8.87 (brs, 1H), 8.18 (d, *J* = 6.9 Hz, 2H), 7.78 (d, *J* = 7.8 Hz, 1H), 7.71–7.58 (m, 3H), 7.56–7.45 (m, 3H), 0.42 (s, 9H); **^{13}C NMR** (125 MHz, $CDCl_3$) δ 167.09, 165.79, 138.89, 136.49, 134.92, 133.57, 131.75, 130.97, 130.14, 129.13, 129.04, 128.59, 123.99, 123.02, -1.00; **HRMS** (ESI) calcd for $C_{19}H_{20}NO_3Si$ $[M+H]^+$ 338.1212, found 338.1205.

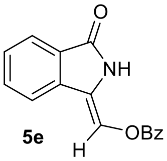

5e

Light-yellow oil (31 mg, 78%); **^{1}H NMR** (500 MHz, DMSO) δ 11.18 (s, 1H), 8.38 (d, *J* = 6.9 Hz, 2H), 8.05 (d, *J* = 7.7 Hz, 1H), 8.02 (s, 1H), 7.74 (t, *J* = 8.2 Hz, 2H), 7.65 (t, *J* = 6.9 Hz, 1H), 7.59 (t, *J* = 7.8 Hz, 2H), 7.53 (t, *J* = 7.4 Hz, 1H); **^{13}C NMR** (125 MHz, DMSO) δ 167.66, 162.78, 136.46, 134.82, 132.67, 130.91, 129.77, 129.47, 129.30, 128.22, 123.50, 123.28, 121.00, 117.34; **HRMS** (ESI) calcd for $C_{16}H_{12}NO_3$ $[M+H]^+$ 266.0817, found 266.0813.

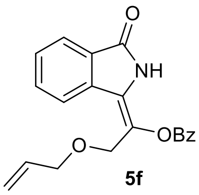

5f

White solid (38.2 mg, 76%); **^{1}H NMR** (500 MHz, $CDCl_3$) δ 8.87 (brs, 1H), 8.21 (d, *J* = 6.9 Hz, 2H), 7.81 (d, *J* = 7.8 Hz, 1H), 7.68 (t, *J* = 7.5 Hz, 1H), 7.65–7.60 (m, 2H), 7.54 (t, *J* = 7.8 Hz, 2H), 7.49 (t, *J* = 7.5 Hz, 1H), 5.91 (ddt, *J* = 17.3, 10.3, 5.7 Hz, 1H), 5.27 (dd, *J* = 17.2, 1.6 Hz, 1H), 5.19 (dd, *J* = 10.4, 1.4 Hz, 1H), 4.71 (s, 2H), 4.12 (dt, *J* = 5.9, 1.4 Hz, 2H); **^{13}C NMR** (125 MHz, $CDCl_3$) δ 167.42, 164.59, 134.30, 134.05, 133.98, 132.44, 130.71, 130.51, 130.18, 129.30, 128.82, 128.71, 128.65, 124.01, 123.46, 118.09, 71.29, 66.25; **HRMS** (ESI) calcd for $C_{20}H_{18}NO_4$ $[M+H]^+$ 336.1236, found 336.1230.

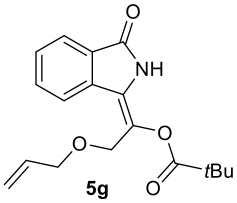

5g

White solid (30.7 mg, 65%); **^{1}H NMR** (500 MHz, $CDCl_3$) δ 8.17 (brs, 1H), 7.87 (d, *J* = 7.6 Hz, 1H), 7.77 (d, *J* = 8.0 Hz, 1H), 7.63 (t, *J* = 7.7 Hz, 1H), 7.52 (t, *J* = 7.5 Hz, 1H), 5.97–5.85 (m, 1H), 5.29 (dd, *J* = 17.2, 1.6 Hz, 1H), 5.22 (dd, *J* = 10.4, 1.4 Hz, 1H), 4.59 (s, 2H), 4.06 (d, *J* = 5.7 Hz, 2H), 1.39 (s, 9H); **^{13}C NMR** (125 MHz, $CDCl_3$) δ 176.44, 167.16, 134.36, 134.06, 132.46,

130.56, 130.35, 129.25, 127.87, 124.05, 123.43, 117.95, 71.13, 65.91, 39.36, 27.16; **HRMS** (ESI) calcd for $C_{18}H_{22}NO_4$ $[M+H]^+$ 316.1549, found 316.1544.

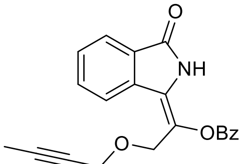

5h

White solid (40.6 mg, 78%); **^{1}H NMR** (500 MHz, $CDCl_3$) δ 9.36 (brs, 1H), 8.22 (d, *J* = 7.3 Hz, 2H), 7.93 (d, *J* = 7.9 Hz, 1H), 7.67 (t, *J* = 7.5 Hz, 1H), 7.64–7.57 (m, 1H), 7.53 (t, *J* = 7.8 Hz, 3H), 7.46 (t, *J* = 7.4 Hz, 1H), 4.79 (s, 2H), 4.24 (q, *J* = 2.3 Hz, 2H), 1.79 (t, *J* = 2.2 Hz, 3H); **^{13}C NMR** (125 MHz, $CDCl_3$) δ 167.74, 164.69, 134.29, 133.82, 132.35, 130.78, 130.61, 129.63, 129.52, 129.26, 128.91, 128.53, 123.90, 123.57, 83.69, 74.68, 65.37, 57.86, 3.55; **HRMS** (ESI) calcd for $C_{21}H_{18}NO_4$ $[M+H]^+$ 348.1236, found 348.1234.

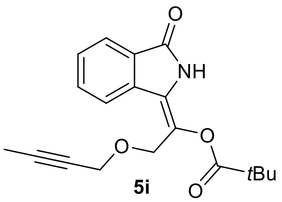

5i

Light-yellow solid (31.4 mg, 64%); **^{1}H NMR** (500 MHz, $CDCl_3$) δ 8.24 (s, 1H), 7.89 (d, *J* = 7.9 Hz, 1H), 7.86 (d, *J* = 7.6 Hz, 1H), 7.62 (t, *J* = 7.7 Hz, 1H), 7.52 (t, *J* = 7.6 Hz, 1H), 4.67 (s, 2H), 4.19 (q, *J* = 2.3 Hz, 2H), 1.87 (t, *J* = 2.3 Hz, 3H), 1.39 (s, 9H); **^{13}C NMR** (125 MHz, $CDCl_3$) δ 176.53, 167.23, 134.35, 132.44, 130.56, 129.79, 129.29, 128.49, 123.98, 123.60, 83.54, 74.64, 65.01, 57.71, 39.37, 27.12, 3.62; **HRMS** (ESI) calcd for $C_{19}H_{22}NO_4$ $[M+H]^+$ 328.1549, found 328.1546.

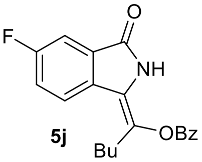

5j

White solid (37.7 mg, 74%); **^{1}H NMR** (500 MHz, $CDCl_3$) δ 8.21 (d, *J* = 6.8 Hz, 2H), 7.77–7.66 (m, 2H), 7.56 (t, *J* = 7.8 Hz, 2H), 7.28 (td, *J* = 8.7, 2.6 Hz, 1H), 6.97 (dd, *J* = 7.5, 2.5 Hz, 1H), 2.87 (t, *J* = 7.7 Hz, 2H), 1.66 (p, *J* = 7.6 Hz, 2H), 1.46 (h, *J* = 7.4 Hz, 2H), 0.94 (t, *J* = 7.3 Hz, 3H); **^{13}C NMR** (125 MHz, $CDCl_3$) δ 166.43 (d, *J* = 3.2 Hz), 164.49, 162.64 (d, *J* = 249.7 Hz), 135.38, 133.76, 133.08 (d, *J* = 8.8 Hz), 130.77 (d, *J* = 2.4 Hz), 130.57, 129.24, 128.53, 124.88, 124.00 (d, *J* = 8.4 Hz), 119.61 (d, *J* = 24.0 Hz), 110.55 (d, *J* = 23.6 Hz), 30.45, 29.26, 22.46, 13.88; **HRMS** (ESI) calcd for $C_{20}H_{19}NO_3F$ $[M+H]^+$ 340.1349, found 340.1345.

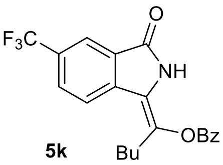

5k

White solid (39.1 mg, 67%); **^{1}H NMR** (500 MHz, $CDCl_3$) δ 10.21 (s, 1H), 8.21 (d, *J* = 7.2 Hz, 2H), 7.87–7.80 (m, 2H), 7.73 (t, *J* = 7.5 Hz, 1H), 7.57 (t, *J* = 7.8 Hz, 2H), 7.50 (s, 1H), 2.92 (t, *J* = 7.7 Hz, 2H), 1.69 (p, *J* = 7.6 Hz, 2H), 1.48 (p, *J* = 7.3 Hz, 2H), 0.95 (t, *J* = 7.3 Hz, 3H); **^{13}C NMR** (125 MHz, $CDCl_3$) δ 166.26, 164.32, 137.66, 133.94, 131.37, 130.58, 130.52 (q, *J* = 33.2 Hz), 130.13, 129.03, 128.77 (q, *J* = 3.8 Hz), 128.56, 124.99, 123.71 (q, *J* = 272.6 Hz), 123.00, 121.21 (q, *J* = 2.5 Hz), 30.70, 29.27, 22.47, 13.86; **HRMS** (ESI) calcd for $C_{21}H_{19}NO_3F_3$ $[M+H]^+$ 390.1317, found 390.1309.

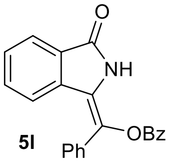

5l

White solid (43.5 mg, 85%); **^{1}H NMR** (500 MHz, $CDCl_3$) δ 9.50 (brs, 1H), 8.21 (d, *J* = 7.7 Hz, 2H), 7.74–7.67 (m, 2H), 7.65 (d, *J* = 7.5 Hz, 1H), 7.52 (t, *J* = 7.7 Hz, 2H), 7.49–7.44 (m, 4H), 7.36 (t, *J* = 7.3 Hz, 1H), 7.32 (t, *J* = 7.5 Hz, 1H), 7.12 (d, *J* = 7.7 Hz, 1H); **^{13}C NMR** (125 MHz, $CDCl_3$) δ 167.72, 164.50, 135.02, 133.76, 133.39, 131.81, 131.64, 130.64, 130.52, 130.07, 129.90, 129.13, 128.95, 128.85, 128.56, 126.33, 123.66, 122.27; **HRMS** (ESI) calcd for $C_{22}H_{16}NO_3$ $[M+H]^+$ 342.1130, found 342.1127.

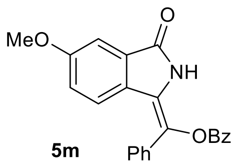

5m

White solid (50.1 mg, 90%); **^{1}H NMR** (500 MHz, CD_2Cl_2) δ 9.09 (brs, 1H), 8.20 (d, *J* = 6.9 Hz, 2H), 7.74–7.64 (m, 3H), 7.54 (t, *J* = 7.8 Hz, 2H), 7.49 (dd, *J* = 4.9, 1.8 Hz, 3H), 7.11 (d, *J* = 8.7 Hz, 1H), 7.03 (d, *J* = 2.5 Hz, 1H), 6.92 (dd, *J* = 8.7, 2.5 Hz, 1H), 3.84 (s, 3H); **^{13}C NMR** (125 MHz, CD_2Cl_2) δ 167.03, 164.46, 160.87, 133.75, 133.53, 132.31, 130.34, 130.19, 129.88, 129.66, 129.17, 128.74, 128.54, 127.51, 125.91, 123.47, 119.96, 106.18, 55.75; **HRMS** (ESI) calcd for $C_{23}H_{18}NO_4$ $[M+H]^+$ 372.1236, found 372.1227.

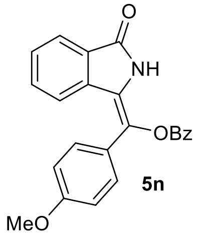

5n

White solid (42.3 mg, 76%); **^{1}H NMR** (500 MHz, $CDCl_3$) δ 9.18 (brs, 1H), 8.19 (d, *J* = 6.8 Hz, 2H), 7.69–7.59 (m, 3H), 7.56–7.48 (m, 3H), 7.40–7.30 (m, 2H), 7.14 (d, *J* = 7.5 Hz, 1H), 6.99 (d, *J* = 8.7 Hz, 2H), 3.86 (s, 3H); **^{13}C NMR** (125 MHz, $CDCl_3$) δ 167.53, 164.52, 160.76, 135.20, 133.78, 131.79, 131.66, 131.55, 130.54, 130.46, 129.13, 128.81, 128.58, 125.76, 125.53, 123.66, 122.24, 114.28, 55.38; **HRMS** (ESI) calcd for $C_{23}H_{18}NO_4$ $[M+H]^+$ 372.1236, found 372.1228.

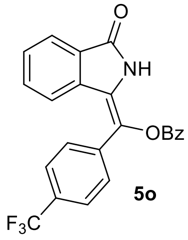

5o

White solid (38.7 mg, 63%); **^{1}H NMR** (500 MHz, $CDCl_3$) δ 9.90 (brs, 1H), 8.21 (d, *J* = 7.6 Hz, 2H), 7.85 (d, *J* = 8.3 Hz, 2H), 7.74 (d, *J* = 8.3 Hz, 2H), 7.69 (t, *J* = 7.5 Hz, 1H), 7.54 (t, *J* = 7.8 Hz, 2H), 7.51–7.44 (m, 1H), 7.39 (dt, *J* = 15.2, 7.3 Hz, 2H), 7.14 (d, *J* = 7.2 Hz, 1H); **^{13}C NMR** (125 MHz, $CDCl_3$) δ 168.00, 164.50, 137.13, 134.50, 133.96, 132.11, 131.63 (q, *J* = 32.7 Hz), 130.72, 130.59, 130.40, 129.95, 129.46, 128.81, 128.62, 127.42, 125.88 (q, *J* = 3.7 Hz), 123.89, 123.83 (q, *J* = 272.0 Hz), 122.07. **HRMS** (ESI) calcd for $C_{23}H_{15}NO_3F_3$ $[M+H]^+$ 410.1004, found 410.1000.

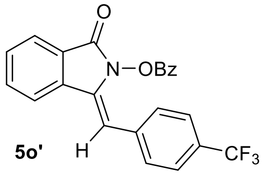

5o'

Light-yellow solid (33.1 mg, 54%); **^{1}H NMR** (500 MHz, $CDCl_3$) δ 7.96 (d, *J* = 7.6 Hz, 1H), 7.80 (d, *J* = 7.7 Hz, 1H), 7.72 (td, *J* = 7.6, 1.1 Hz, 1H), 7.64–7.60 (m, 3H), 7.53 (tt, *J* = 7.4, 1.3 Hz, 1H), 7.47 (d, *J* = 8.0 Hz, 2H), 7.38 (d, *J* = 8.1 Hz, 2H), 7.30 (t, *J* = 7.9 Hz, 2H), 6.72 (s, 1H); **^{13}C NMR** (125 MHz, $CDCl_3$) δ 164.29, 162.75, 137.13, 134.76, 134.38, 133.94, 133.40, 130.15, 129.81, 129.77, 129.33 (q, *J* = 32.5 Hz), 128.35, 125.67, 125.24, 124.79 (q, *J* = 3.7 Hz), 124.33, 123.82 (q, *J* = 271.0 Hz), 120.06, 105.05. **HRMS** (ESI) calcd for $C_{23}H_{15}NO_3F_3$ $[M+H]^+$ 410.1004, found 410.1000.

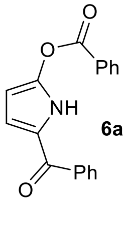

6a

White solid (27.3 mg, 94%); **^{1}H NMR** (500 MHz, $CDCl_3$) δ 10.52 (brs, 1H), 8.19 (d, *J* = 6.9 Hz, 2H), 7.85 (d, *J* = 6.9 Hz, 2H), 7.67 (t, *J* = 7.4 Hz, 1H), 7.55 (t, *J* = 7.4 Hz, 1H), 7.51 (t, *J* = 7.8 Hz, 2H), 7.45 (t, *J* = 7.7 Hz, 2H), 6.86 (dd, *J* = 4.1, 2.6 Hz, 1H), 6.21 (dd, *J* = 4.1, 2.4 Hz, 1H); **^{13}C NMR** (125 MHz, $CDCl_3$) δ 184.27, 162.81, 143.52, 138.10, 134.29, 131.72, 130.40, 128.92, 128.82, 128.32, 128.14, 124.75, 119.89, 98.15; **HRMS** (ESI) calcd for $C_{18}H_{14}NO_3$ $[M+H]^+$ 292.0974, found 292.0974.

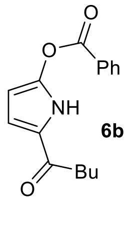

6b

White solid (25 mg, 92%); **^{1}H NMR** (500 MHz, $CDCl_3$) δ 10.26 (brs, 1H), 8.18 (dd, *J* = 8.3, 1.4 Hz, 2H), 7.69–7.62 (m, 1H), 7.52 (t, *J* = 7.8 Hz, 3H), 6.89 (dd, *J* = 4.1, 2.4 Hz, 1H), 6.11 (dd, *J* = 4.1, 2.1 Hz, 1H), 2.74–2.66 (m, 2H), 1.66 (p, *J* = 7.6 Hz, 2H), 1.36 (h, *J* = 7.4 Hz, 2H), 0.90 (t, *J* = 7.3 Hz, 3H); **^{13}C NMR** (125 MHz, $CDCl_3$) δ 190.57, 162.92, 142.71, 134.23, 130.34, 128.77, 128.23, 125.66, 116.18, 97.51, 36.99,

27.55, 22.57, 13.86; **HRMS** (ESI) calcd for $C_{16}H_{18}NO_3$ $[M+H]^+$ 272.1287, found 272.1288.

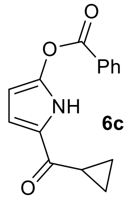
6c

White solid (22.7 mg, 89%); **^{1}H NMR** (500 MHz, $CDCl_3$) δ 10.33 (brs, 1H), 8.18 (d, *J* = 7.1 Hz, 2H), 7.66 (t, *J* = 7.3 Hz, 1H), 7.53 (t, *J* = 7.6 Hz, 2H), 7.01 (dd, *J* = 4.1, 2.7 Hz, 1H), 6.15 (dd, *J* = 4.1, 2.5 Hz, 1H), 2.42 (tt, *J* = 8.1, 4.5 Hz, 1H), 1.17–1.09 (m, 2H), 0.95–0.87 (m, 2H); **^{13}C NMR** (125 MHz, $CDCl_3$) δ 189.57, 162.90, 142.59, 134.22, 130.35, 128.76, 128.25, 126.15, 115.95, 97.63, 16.33, 10.36; **HRMS** (ESI) calcd for $C_{15}H_{14}NO_3$ $[M+H]^+$ 256.0974, found 256.0974.

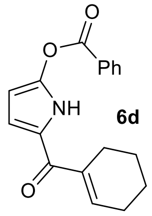
6d

White solid (17.1 mg, 58%); **^{1}H NMR** (500 MHz, $CDCl_3$) δ 10.19 (brs, 1H), 8.20–8.15 (m, 2H), 7.66 (t, *J* = 7.3 Hz, 1H), 7.52 (t, *J* = 7.7 Hz, 2H), 6.79–6.76 (m, 1H), 6.75–6.72 (m, 1H), 6.11 (dd, *J* = 4.0, 2.2 Hz, 1H), 2.35 (s, 2H), 2.25 (dt, *J* = 4.6, 2.2 Hz, 2H), 1.72–1.61 (m, 4H); **^{13}C NMR** (125 MHz, $CDCl_3$) δ 186.05, 162.89, 142.58, 137.87, 137.57, 134.19, 130.34, 128.76, 128.28, 124.64, 117.66, 97.34, 25.75, 24.48, 22.10, 21.77; **HRMS** (ESI) calcd for $C_{18}H_{18}NO_3$ $[M+H]^+$ 296.1287, found 296.1284.

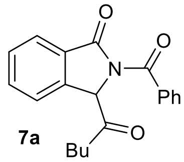
7a

Light-yellow solid (28.2 mg, 88%); **^{1}H NMR** (500 MHz, $CDCl_3$) δ 7.90 (d, *J* = 7.7 Hz, 1H), 7.76–7.71 (m, 2H), 7.70 (dd, *J* = 7.6, 1.2 Hz, 1H), 7.62–7.54 (m, 3H), 7.48 (t, *J* = 7.7 Hz, 2H), 5.91 (s, 1H), 2.70–2.51 (m, 2H), 1.58 (p, *J* = 7.4 Hz, 2H), 1.26 (qd, *J* = 7.5, 5.4 Hz, 2H), 0.85 (t, *J* = 7.4 Hz, 3H); **^{13}C NMR** (125 MHz, $CDCl_3$) δ 203.04, 170.17, 166.33, 139.00, 134.65, 133.78, 132.24, 130.64, 129.88, 128.89, 127.94, 125.97, 122.84, 68.08, 38.25, 25.20, 22.10, 13.75; ; **HRMS** (ESI) calcd for $C_{20}H_{20}NO_3$ $[M+H]^+$ 322.1443, found 322.1443.

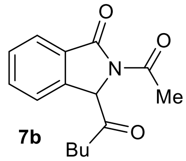
7b

Light-brown solid (16.9 mg, 65%); **^{1}H NMR** (500 MHz, $CDCl_3$) δ 7.94 (d, *J* = 7.7 Hz, 1H), 7.67 (td, *J* = 7.6, 1.2 Hz, 1H), 7.59–7.51 (m, 2H), 5.63 (s, 1H), 2.70 (s, 3H), 2.55–2.40 (m, 2H), 1.52 (p, *J* = 7.4 Hz, 2H), 1.26–1.16 (m, 2H), 0.82 (t, *J* = 7.4 Hz, 3H); **^{13}C NMR** (125 MHz, $CDCl_3$) δ 203.04, 171.01, 167.27, 138.75, 134.62, 130.74, 129.80, 125.81, 122.76, 67.33, 38.00, 25.09, 24.66, 22.04, 13.72; **HRMS** (ESI) calcd for $C_{15}H_{18}NO_3$ $[M+H]^+$ 260.1287, found 260.1285.

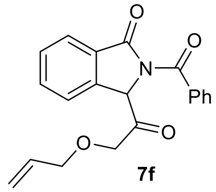
7f

Light-yellow oil (27.5 mg, 82%); **^{1}H NMR** (500 MHz, $CDCl_3$) δ 7.88 (d, *J* = 7.6 Hz, 1H), 7.76–7.66 (m, 4H), 7.60–7.52 (m, 2H), 7.47 (t, *J* = 7.6 Hz, 2H), 6.38 (s, 1H), 5.96 (ddt, *J* = 16.4, 10.9, 5.7 Hz, 1H), 5.36 (dd, *J* = 17.2, 1.6 Hz, 1H), 5.28 (dd, *J* = 10.5, 1.1 Hz, 1H), 4.37 (d, *J* = 2.7 Hz, 2H), 4.25–4.14 (m, 2H); **^{13}C NMR** (125 MHz, $CDCl_3$) δ 200.93, 170.04, 166.23, 138.99, 134.60, 133.81, 133.26, 132.09, 130.91, 129.71, 128.84, 127.88, 125.93, 123.53, 118.60, 73.53, 72.77, 64.92; **HRMS** (ESI) calcd for $C_{20}H_{18}NO_4$ $[M+H]^+$ 336.1236, found 336.1231.

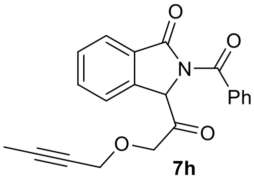
7h

Colorless oil (27.8 mg, 80%); **^{1}H NMR** (500 MHz, $CDCl_3$) δ 7.88 (d, *J* = 7.7 Hz, 1H), 7.76 (d, *J* = 7.7 Hz, 1H), 7.71 (dd, *J* = 21.7, 7.6 Hz, 3H), 7.56 (q, *J* = 7.9 Hz, 2H), 7.47 (t, *J* = 7.6 Hz, 2H), 6.37 (s, 1H), 4.49 (d, *J* = 16.8 Hz, 1H), 4.43 (d, *J* = 16.9 Hz, 1H), 4.37 (dd, *J* = 15.4, 2.4 Hz, 1H), 4.30 (dd, *J* = 15.4, 2.4 Hz, 1H), 1.87 (t, *J* = 2.3 Hz, 3H); **^{13}C NMR** (125 MHz, $CDCl_3$) δ 200.50, 169.97, 166.24, 138.96, 134.59, 133.83, 132.07, 130.89, 129.70,

128.84, 127.87, 125.88, 123.67, 84.54, 73.66, 72.63, 64.91, 59.49, 3.62; **HRMS** (ESI) calcd for $C_{21}H_{18}NO_4$ $[M+H]^+$ 348.1236, found 348.1237.

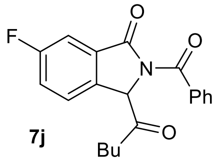

7j

White solid (27.1 mg, 80%); **^{1}H NMR** (500 MHz, $CDCl_3$) δ 7.72 (d, *J* = 7.4 Hz, 2H), 7.62–7.53 (m, 3H), 7.48 (t, *J* = 7.6 Hz, 2H), 7.41 (td, *J* = 8.5, 2.4 Hz, 1H), 5.87 (s, 1H), 2.69–2.52 (m, 2H), 1.58 (p, *J* = 7.4 Hz, 2H), 1.25 (qt, *J* = 11.1, 5.6 Hz, 2H), 0.85 (t, *J* = 7.4 Hz, 3H); **^{13}C NMR** (125 MHz, $CDCl_3$) δ 202.81, 169.98, 165.29 (d, *J* = 3.1 Hz), 163.60 (d, *J* = 251.2 Hz), 134.58 (d, *J* = 1.2 Hz), 133.47, 132.79 (d, *J* = 8.5 Hz), 132.44, 128.92, 128.00, 124.70 (d, *J* = 8.4 Hz), 122.44 (d, *J* = 23.9 Hz), 112.43 (d, *J* = 23.6 Hz), 67.59, 38.27, 25.16, 22.11, 13.75; **HRMS** (ESI) calcd for $C_{20}H_{19}NO_3F$ $[M+H]^+$ 340.1349, found 340.1345.

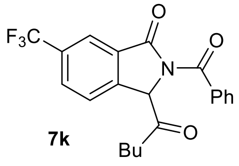

7k

White solid (38.8 mg, 74%); **^{1}H NMR** (500 MHz, $CDCl_3$) δ 10.21 (brs, 1H), 8.21 (d, *J* = 7.2 Hz, 2H), 7.87–7.80 (m, 2H), 7.73 (t, *J* = 7.5 Hz, 1H), 7.57 (t, *J* = 7.8 Hz, 2H), 7.50 (s, 1H), 2.92 (t, *J* = 7.7 Hz, 2H), 1.69 (p, *J* = 7.6 Hz, 2H), 1.48 (p, *J* = 7.3 Hz, 2H), 0.95 (t, *J* = 7.3 Hz, 3H); **^{13}C NMR** (125 MHz, $CDCl_3$) δ 166.26, 164.32, 137.66, 133.94, 130.58, 130.53 (q, *J* = 32.9 Hz), 129.03, 128.77 (q, *J* = 3.8 Hz), 128.56, 124.99, 123.74 (q, *J* = 265 Hz), 122.81, 121.21 (q, *J* = 3.8 Hz)), 30.70, 29.27, 22.47, 13.86; **HRMS** (ESI) calcd for $C_{21}H_{19}NO_3F_3$ $[M+H]^+$ 390.1317, found 390.1306.

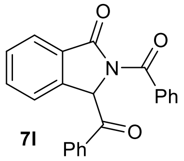

7l

White solid (31.4 mg, 92%); **^{1}H NMR** (500 MHz, $CDCl_3$) δ 8.15 (d, *J* = 7.4 Hz, 2H), 7.92 (d, *J* = 6.9 Hz, 1H), 7.78 (d, *J* = 7.5 Hz, 2H), 7.72 (t, *J* = 7.4 Hz, 1H), 7.61 (t, *J* = 7.7 Hz, 2H), 7.55 (dq, *J* = 15.2, 7.3 Hz, 3H), 7.47 (t, *J* = 7.6 Hz, 2H), 7.25 (d, *J* = 3.5 Hz, 1H), 6.97 (s, 1H); **^{13}C NMR** (125 MHz, $CDCl_3$) δ 192.12, 169.95, 166.38, 139.52, 135.35, 134.34, 134.26, 133.99, 132.04, 131.07, 129.71, 129.24, 129.17, 128.94, 127.87, 126.06, 123.01, 63.66; **HRMS** (ESI) calcd for $C_{22}H_{16}NO_3$ $[M+H]^+$ 342.1130, found 342.1129.

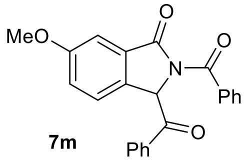

7m

Light-yellow oil (26 mg, 70%); **^{1}H NMR** (500 MHz, $CDCl_3$) δ 8.12 (dd, *J* = 8.4, 1.3 Hz, 2H), 7.80–7.74 (m, 2H), 7.70 (t, *J* = 7.5 Hz, 1H), 7.62–7.52 (m, 3H), 7.46 (t, *J* = 7.7 Hz, 2H), 7.34 (d, *J* = 2.4 Hz, 1H), 7.13 (d, *J* = 8.5 Hz, 1H), 7.09 (dd, *J* = 8.5, 2.4 Hz, 1H), 6.88 (s, 1H), 3.83 (s, 3H); **^{13}C NMR** (125 MHz, $CDCl_3$) δ 192.27, 169.86, 166.47, 161.03, 135.35, 134.27, 134.03, 132.46, 132.01, 131.85, 129.21, 129.10, 128.96, 127.85, 123.97, 122.73, 108.23, 63.28, 55.78; **HRMS** (ESI) calcd for $C_{23}H_{18}NO_4$ $[M+H]^+$ 372.1236, found 372.1230.

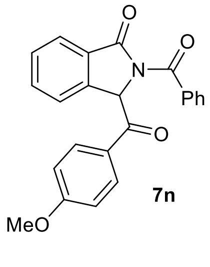

7n

Light-yellow solid (26 mg, 70%); **^{1}H NMR** (500 MHz, $CDCl_3$) δ 8.16 (d, *J* = 8.7 Hz, 2H), 7.90 (d, *J* = 7.0 Hz, 1H), 7.79 (d, *J* = 7.1 Hz, 2H), 7.58–7.50 (m, 3H), 7.47 (t, *J* = 7.6 Hz, 2H), 7.28 (d, *J* = 7.5 Hz, 1H), 7.08 (d, *J* = 8.4 Hz, 2H), 6.95 (s, 1H), 3.93 (s, 3H); **^{13}C NMR** (125 MHz, $CDCl_3$) δ 190.24, 169.98, 166.52, 164.59, 139.94, 134.14, 131.98, 131.64, 131.10, 129.57, 128.96, 128.08, 127.85, 125.95, 122.99, 114.49, 63.28, 55.69; **HRMS** (ESI) calcd for $C_{23}H_{18}NO_4$ $[M+H]^+$ 372.1236, found 372.1229.

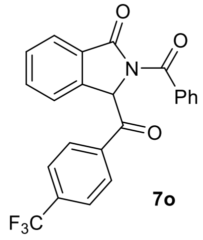

7o

Light-yellow solid (31.9 mg, 78%);**^{1}H NMR** (500 MHz, $CDCl_3$) δ 8.22 (d, *J* = 8.3 Hz, 2H), 7.94 (d, *J* = 7.3 Hz, 1H), 7.86 (d, *J* = 8.3 Hz, 2H), 7.74 (d, *J* = 8.1 Hz, 2H), 7.62–7.51 (m, 3H), 7.47 (t, *J* = 7.7 Hz, 2H), 7.23 (d, *J* = 7.3 Hz, 1H), 6.90 (s, 1H); **^{13}C NMR** (125 MHz, $CDCl_3$) δ 191.79, 169.89, 166.06, 138.99, 138.25, 135.44 (q, *J* = 32.8 Hz), 134.49, 133.65, 132.21, 131.08, 129.99, 129.39, 128.92, 127.90, 123.41 (q, *J* = 271 Hz), 126.29, 122.81, 63.77; **HRMS** (ESI) calcd for $C_{23}H_{15}NO_3F_3$ $[M+H]^+$ 410.1004, found 410.0997.

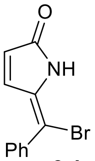

2a'

Light-yellow solid (15 mg, 40%); **^{1}H NMR** (500 MHz, $CDCl_3$) δ 8.03 (brs, 1H), 7.48–7.43 (m, 2H), 7.43–7.37 (m, 3H), 7.04 (dd, *J* = 5.5, 1.5 Hz, 1H), 6.29 (dd, *J* = 5.5, 1.5 Hz, 1H); **^{13}C NMR** (125 MHz, $CDCl_3$) δ 170.93, 138.44, 136.40, 135.61, 130.22, 129.69, 128.56, 126.21, 109.76; **HRMS** (ESI) calcd for $C_{11}H_9NOBr$ $[M+H]^+$ 249.9868, found 249.9871.

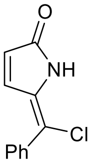

2a''

Light-yellow solid (23.1 mg, 75%); **^{1}H NMR** (500 MHz, $CDCl_3$) δ 8.20 (brs, 1H), 7.52–7.46 (m, 2H), 7.46–7.39 (m, 3H), 7.11 (d, *J* = 5.8 Hz, 1H), 6.26 (d, *J* = 5.8 Hz, 1H); **^{13}C NMR** (125 MHz, $CDCl_3$) δ 170.84, 136.31, 135.79, 135.01, 129.78, 129.63, 128.63, 125.94, 118.57; **HRMS** (ESI) calcd for $C_{11}H_9NOCl$ $[M+H]^+$ 206.0373, found 206.0378.

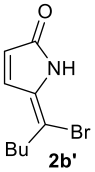

2b'

Light-yellow solid (15.5 mg, 45%); **^{1}H NMR** (500 MHz, $CDCl_3$) δ 7.63 (s, 1H), 7.19 (dd, *J* = 5.8, 1.7 Hz, 1H), 6.23 (dd, *J* = 5.8, 1.8 Hz, 1H), 2.69 (t, *J* = 7.3 Hz, 2H), 1.62 (p, *J* = 7.4 Hz, 2H), 1.33 (h, *J* = 7.4 Hz, 2H), 0.94 (t, *J* = 7.4 Hz, 3H); **^{13}C NMR** (125 MHz, $CDCl_3$) δ 170.86, 137.45, 133.01, 125.41, 115.28, 35.54, 30.97, 21.62, 13.78; **HRMS** (ESI) calcd for $C_9H_{13}NOBr$ $[M+H]^+$ 230.0181, found 230.0187.

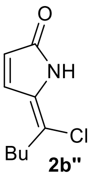

2b''

Light-red solid (21.1 mg, 76%); **^{1}H NMR** (500 MHz, $CDCl_3$) δ 8.25 (brs, 1H), 7.18 (dd, *J* = 5.7, 1.7 Hz, 1H), 6.17 (dd, *J* = 5.8, 1.8 Hz, 1H), 2.58 (t, *J* = 7.3 Hz, 2H), 1.62 (p, *J* = 7.5 Hz, 2H), 1.38–1.28 (m, 2H), 0.93 (t, *J* = 7.3 Hz, 3H) ; **^{13}C NMR** (125 MHz, $CDCl_3$) δ 171.17, 135.55, 133.33, 125.02, 122.44, 33.87, 30.04, 21.72, 13.74; **HRMS** (ESI) calcd for $C_9H_{13}NOCl$ $[M+H]^+$ 186.0686, found 186.0692.

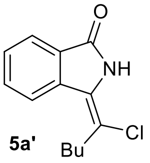

5a'

White solid (29 mg, 82%); **^{1}H NMR** (500 MHz, $CDCl_3$) δ 7.90 (d, *J* = 7.6 Hz, 1H), 7.81 (brs, 1H), 7.72 (d, *J* = 7.9 Hz, 1H), 7.66–7.59 (m, 1H), 7.51 (t, *J* = 7.2 Hz, 1H), 2.91 (t, *J* = 7.5 Hz, 2H), 1.74 (p, *J* = 7.6 Hz, 2H), 1.47 (dq, *J* = 15.4, 7.7 Hz, 2H), 0.98 (t, *J* = 7.4 Hz, 3H); **^{13}C NMR** (125 MHz, $CDCl_3$) δ 166.52, 134.91, 132.52, 131.05, 130.39, 128.87, 124.29, 122.53, 118.95, 34.71, 30.03, 22.11, 13.92; **HRMS** (ESI) calcd for $C_{13}H_{15}NOCl$ $[M+H]^+$ 236.0842, found 236.0845.

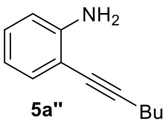

5a''

Orange solid (13 mg, 50%); **^{1}H NMR** (500 MHz, $CDCl_3$) δ 7.24 (d, *J* = 7.6 Hz, 1H), 7.07 (t, *J* = 7.0 Hz, 1H), 6.71–6.63 (m, 2H), 2.48 (t, *J* = 7.1 Hz, 2H), 1.62 (p, *J* = 7.1 Hz, 2H), 1.50 (h, *J* = 7.2 Hz, 2H), 0.96 (t, *J* = 7.4 Hz, 3H); **^{13}C NMR** (125 MHz, $CDCl_3$) δ 147.62, 132.04, 128.80, 117.86, 114.15, 109.02, 95.76, 76.99,

31.07, 22.08, 19.36, 13.66; **HRMS** (ESI) calcd for $C_{12}H_{16}N$ $[M+H]^+$ 174.1283, found 174.1287.

5. X-Ray Crystallographic Data

All X-Ray data can be obtained free of charge from The Cambridge Crystallographic Data Centre via www.ccdc.cam.ac.uk/data_request/cif.

Compound 5o'

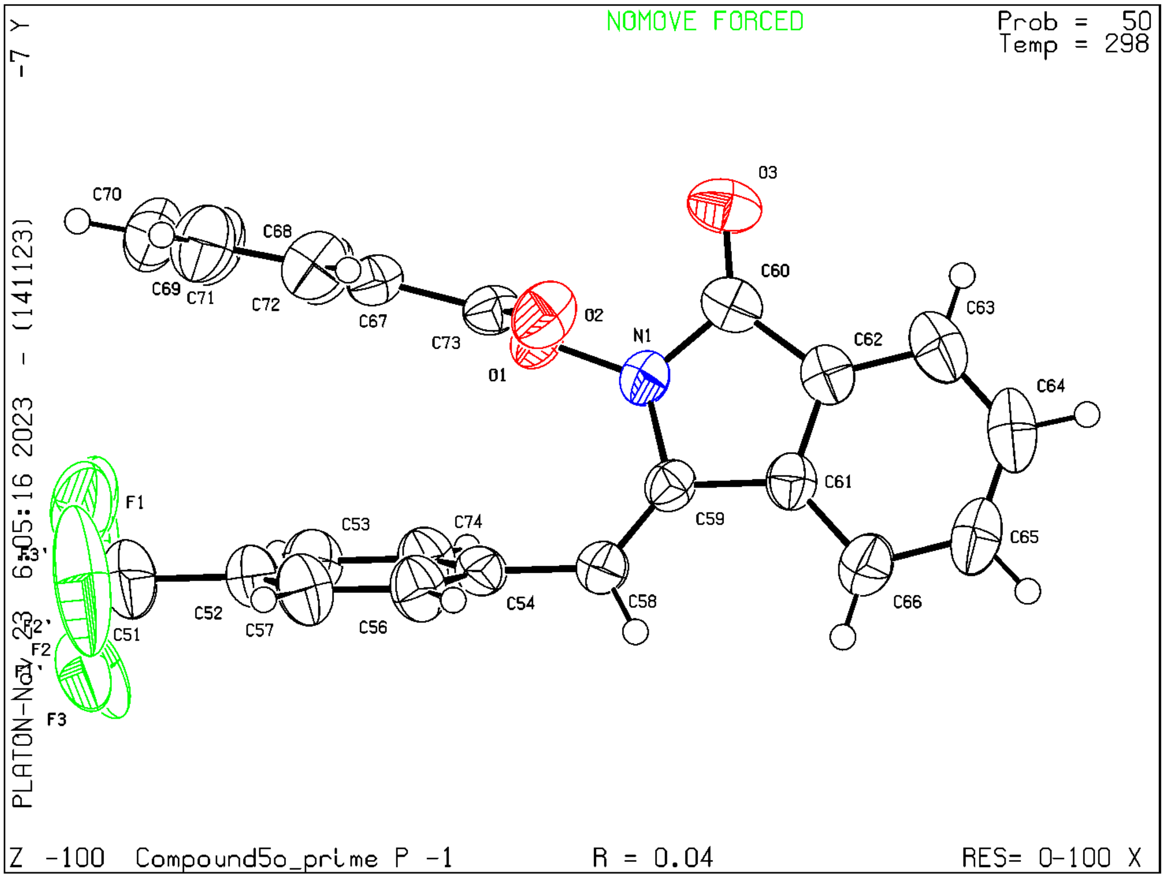

The crystal data of **5o'** has been deposited in CCDC with number 2269723. **Chemical formula**: $C_{23}H_{14}F_3NO_3$; **Formula weight**: 409.35; **Temperature**: 298 K; **Wavelength**: 0.71073 Å; **Crystal size**: 0.47 × 0.33 × 0.20 mm; **Crystal system**: Triclinic; **Space group**: *P*-1; ***a, b, c* (Å)**: 7.8488 (6), 9.6423 (7), 14.1343 (11); **α, β, γ (°)**: 71.226 (3), 73.977 (3), 76.858 (3); ***V* (Å³)**: 962.09 (13); ***Z***: 2; **Absorption coefficient**: 0.11 mm^{-1}; **F(000)**: 420.

Compound 6b

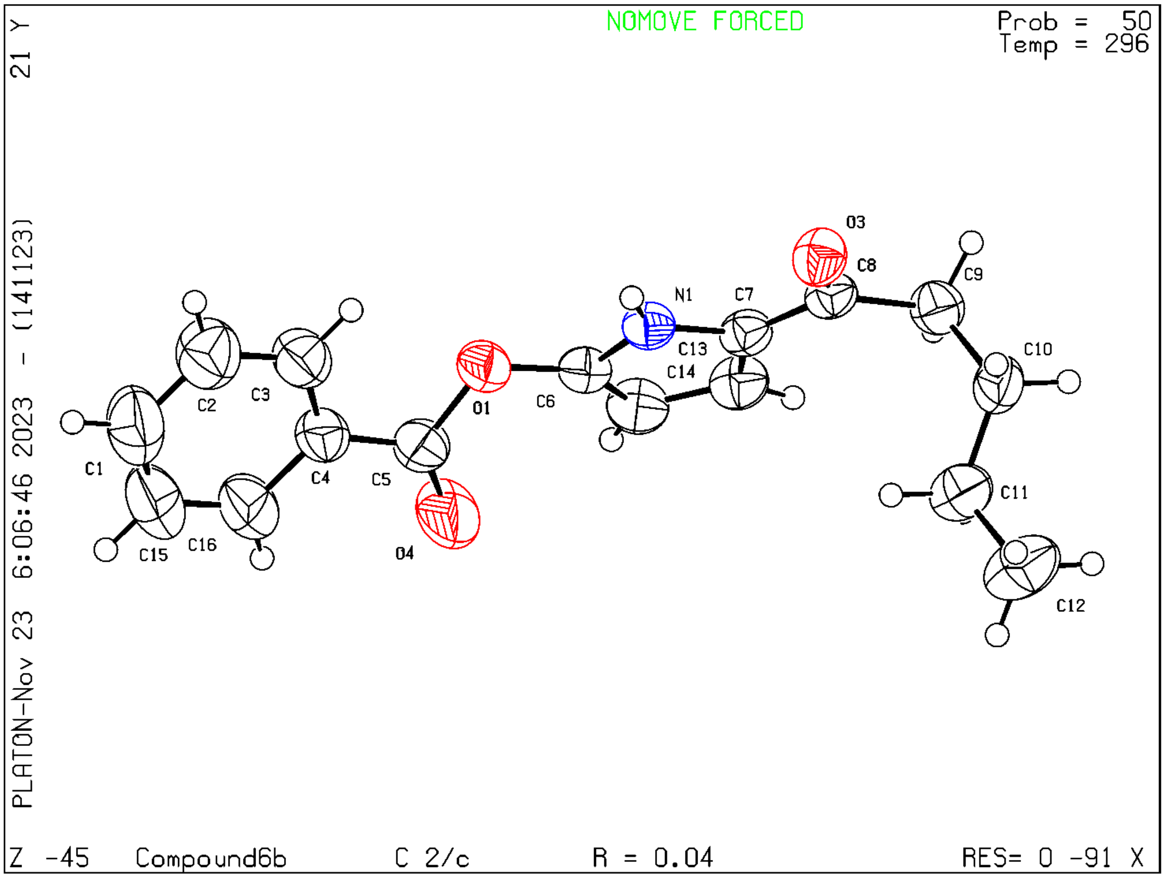

The crystal data of **6b** has been deposited in CCDC with number 2269724. **Chemical formula**: $C_{16}H_{17}NO_3$; **Formula weight**: 271.32; **Temperature**: 296 K; **Wavelength**: 0.71073 Å; **Crystal size**: 0.33 × 0.33 × 0.17 mm; **Crystal system**: Monoclinic; **Space group**: *C*2/*c*; ***a, b, c* (Å)**: 30.41 (3), 4.813 (4), 21.88 (2); **α, β, γ (°)**: 90, 114.256 (9), 90; ***V* (Å^3)**: 2921 (5); ***Z***: 8; **Absorption coefficient**: 0.09 mm^{-1}; **F(000)**: 1144.

Compound 7b

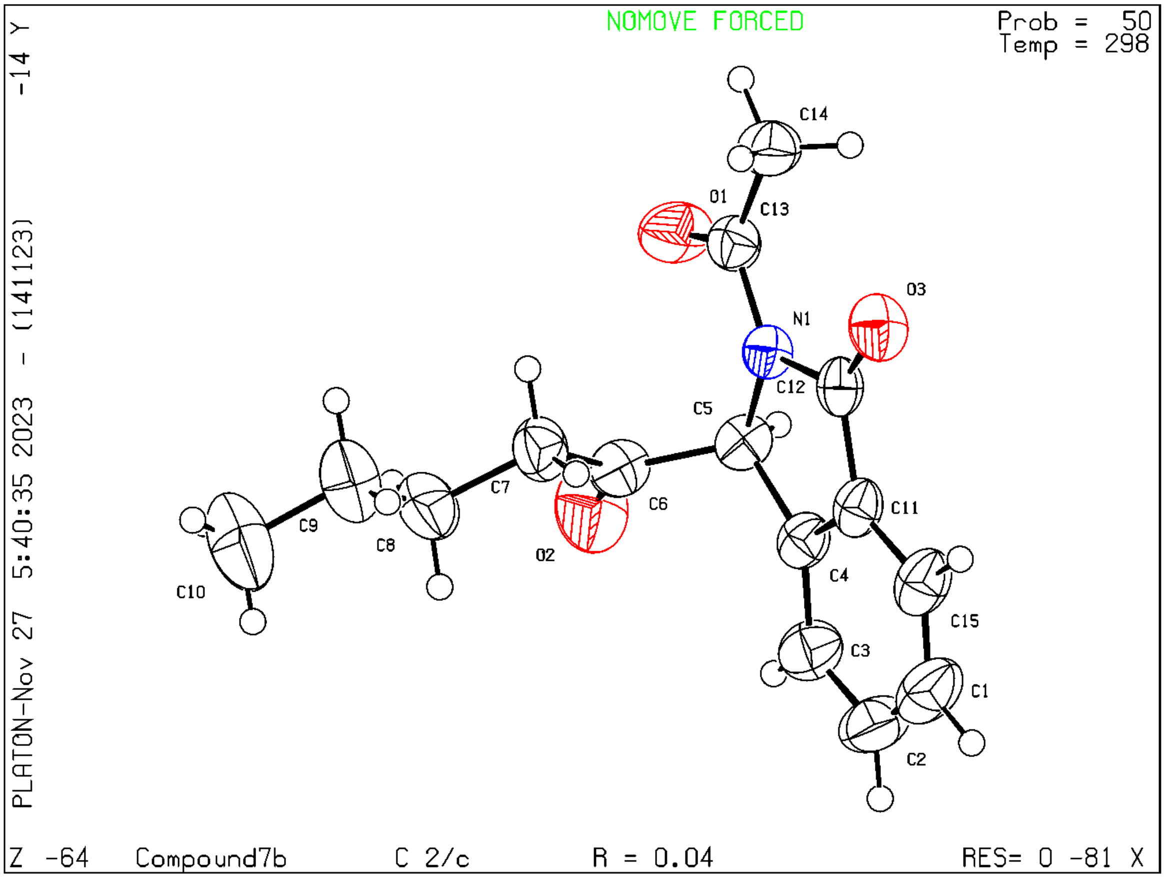

The crystal data of **7b** has been deposited in CCDC with number 2269722. **Chemical formula**: $C_{15}H_{17}NO_3$; **Formula weight**: 259.29; **Temperature**: 298 K; **Wavelength**: 0.71073 Å; **Crystal size**: 0.60 × 0.24 × 0.11 mm; **Crystal system**: Monoclinic; **Space group**: *C*2/*c*; ***a*, *b*, *c* (Å)**: 31.908 (5), 5.7559 (10), 16.822 (3); **α, β, γ (°)**: 90, 113.951 (2), 90; ***V* (Å^3)**: 2823.5 (8); ***Z***: 8; **Absorption coefficient**: 0.09 mm^{-1}; **F(000)**: 1104.

6. Computational Details

6.1 Computational Methods

All density functional theory (DFT) calculations were carried out using the Gaussian 16 program[12]. Geometries of intermediates and transition states were optimized using the B3LYP functional[13,14] with Grimme's DFT-D3(BJ) dispersion correction[15] with a mixed basis set of SDD for Fe and 6-31G(d) for other atoms. Vibrational frequency calculations were performed to confirm if each optimized structure is a local minimum or a transition state structure. Geometries of **16**, **TS3** and **TS4** were obtained through constrained optimization using modredundant keyword. Each optimized transition state structure **TS1**, **TS1'**, and **TS2** has only one imaginary (negative) frequency, and all minima have no imaginary frequencies. The M06 functional[16] with a mixed basis set of SDD for Fe and 6-311+G(d,p) for other atoms was used for single-point energy calculations in CH3CN solvent using the SMD continuum solvation model[17]. The reported Gibbs free energies and enthalpies include thermal corrections at 298K.

6.2 Spin State of 8, 11 and 12

The relative energies of different spin states of **8**, **11** and **12** are shown in Figure S1. The geometries of doublet, quartet and sextet of **8**, **11** and **12** were located. Quartet was found to be the most stable spin state.

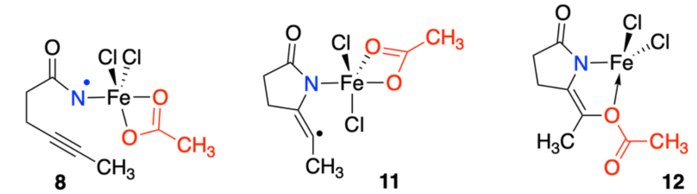

ΔG (kcal/mol)	Quartet	Sextet	Doublet
**8**	0.0	20.3	31.4
**11**	–49.3	–19.5	–18.1
**12**	–87.7	–87.4	–44.6

Figure S1. Relative energies of **8**, **11** and **12** at different spin states.

6.3 Conformers of 8 and 11

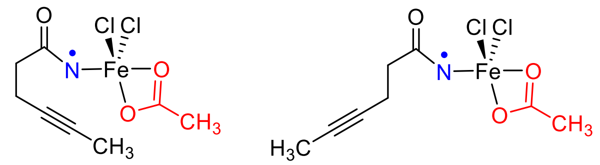

ΔG (ΔH) kcal/mol	**8**	**8a**
	0.0	2.8
	(0.0)	(5.8)

Figure S2. The bent (*gauche*) conformer of **8** is more stable than the linear (*anti*) conformer

8a, possibly due to stabilizing dispersion interactions.

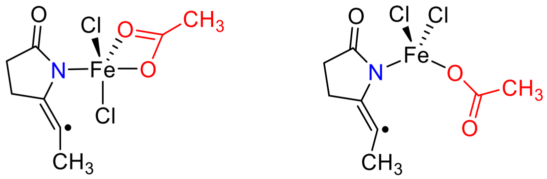

	**11**	**11a**
Δ*G* (Δ*H*) kcal/mol	0.0 (0.0)	6.0 (4.6)

Figure S3. Conformers of **11**.

6.4 Spin Densities

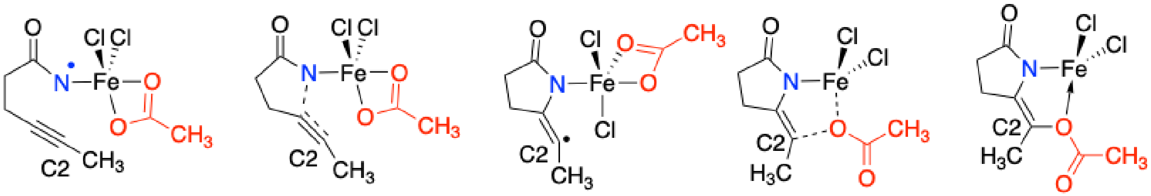

	**8**	**TS1**	**11**	**TS2**	**12**
ρ(Fe)	3.953	4.052	4.011	3.983	3.800
ρ(N)	-1.371	-1.257	-0.219	-0.232	-0.274
ρ(C2)	-0.121	-0.508	-1.431	-1.314	-0.476

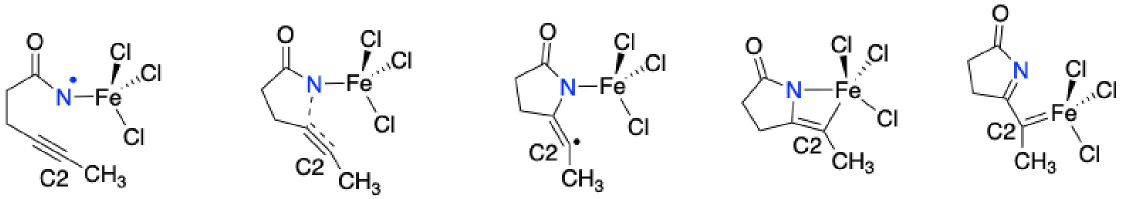

	**9**	**TS1'**	**10**	**13**	**14**
ρ(Fe)	3.922	3.901	3.935	2.855	3.586
ρ(N)	-1.294	-1.134	-0.205	0.080	-0.266
ρ(C2)	-0.294	-0.440	-1.552	-0.029	-1.097

Figure S4. Mulliken spin densities (ρ) of all species in the quartet spin state.

6.5 Competing Pathways

The nitrene-alkyne metathesis pathway to generate the corresponding iron alkylidene **14** via a stepwise [2+2] cycloaddition was considered (Figure S5). The cycloaddition to form azametallacyclobutene **13** from the alkenyl radical intermediate **10** is endergonic by 44.8 kcal/mol, which is significantly less favorable than the acyloxy transfer from the alkenyl radical intermediate **11** (*via* **TS2**).

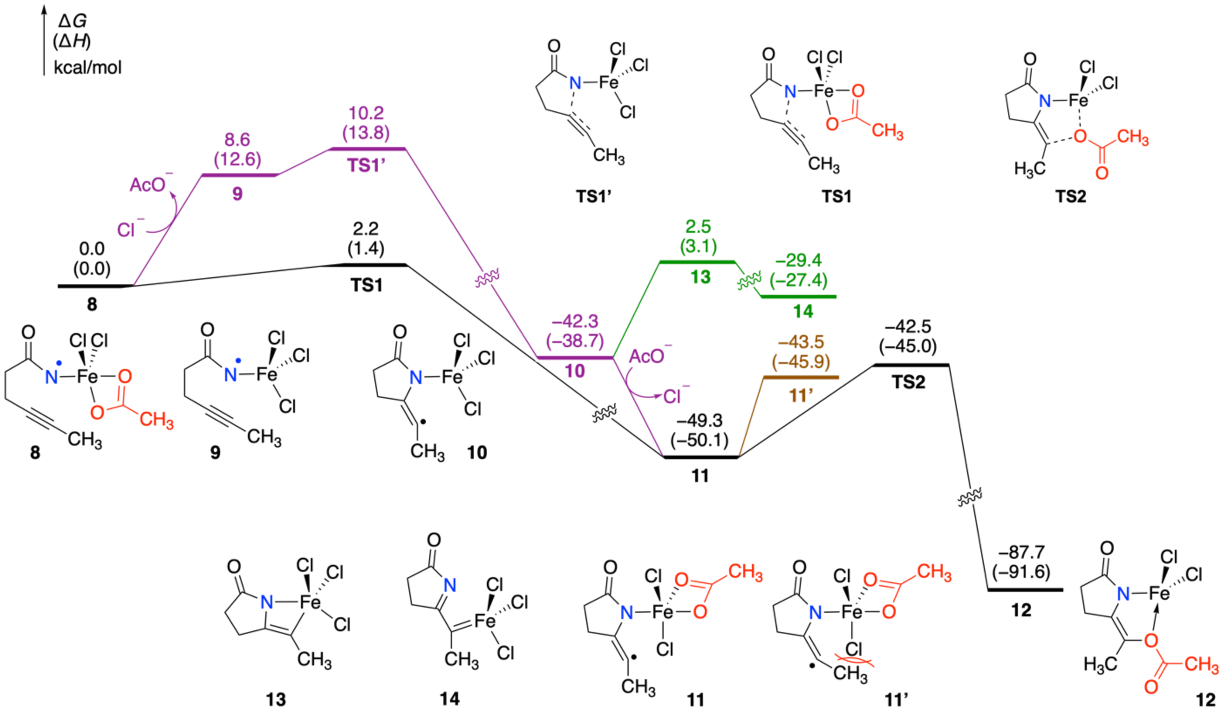

Figure S5. Computed energy profile of the nitrene-alkyne metathesis pathway (green).

Next, we considered the intramolecular nucleometallation of a π-alkyne iron(III) complex, which upon dissociation of an acetic acid could form iron alkylidene **14**. Because of the weak nucleophilicity of the hydroxamate nitrogen, the nucleometallation product **16** cannot be located in our geometry optimization. By using constrained geometry optimization, intermediate **16** is estimated to be 8.7 kcal/mol less stable than **15**, suggesting that this is less likely the most favorable pathway leading to the carboxyamidation product.

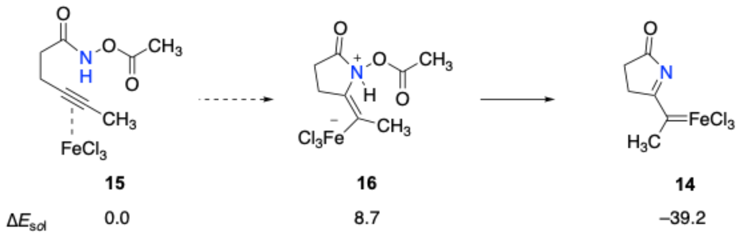

Figure S6. Computational results of a nucleometallation pathway. Complex **16** is computed using constrained geometry optimization.

The concerted [3+2] and [5+2] cycloaddition pathways can be ruled out based on the estimated activation barriers using structures obtained from constrained geometry optimization. The estimated activation barriers of [3+2] (**TS3**) and [5+2] (**TS4**) cycloadditions are 15.3 and 15.4 kcal/mol less favorable, respectively, than the intramolecular radical cyclization (**TS1**).

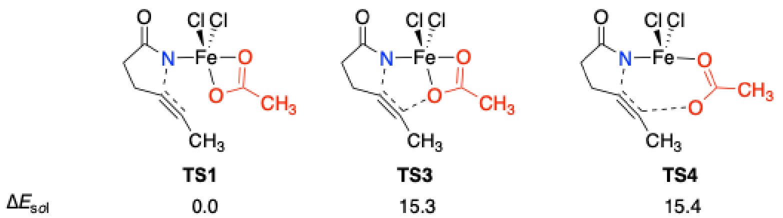

Figure S7. Computational results of concerted [3+2] and [5+2] cycloadditions. In the geometry optimization of **TS3** and **TS4**, the forming C–O bonds are constrained at 2.06 and 2.12 Å, respectively.

6.6 Cartesian Coordinates for Computed Structures

10
B3LYP SCF energy: -1867.44535717 a.u.
B3LYP enthalpy: -1867.306446 a.u.
B3LYP free energy: -1867.371641 a.u.
M06 SCF energy in solution: -1867.30548654 a.u.
M06 enthalpy in solution: -1867.166575 a.u.
M06 free energy in solution: -1867.231770 a.u.

Cartesian coordinates

ATOM	X	Y	Z
C	-1.297931	2.007073	0.000012
O	-0.585758	2.976223	-0.000571
C	-2.828181	1.965401	0.000589
H	-3.196216	2.498878	-0.880353
H	-3.195379	2.497931	0.882471
N	-0.805827	0.661029	0.000242
C	-3.186216	0.472867	0.000094
H	-3.757230	0.165721	-0.882069
H	-3.757806	0.165165	0.881677
C	-2.319711	-2.817239	-0.000031
H	-3.410090	-2.664984	-0.000374
H	-2.048443	-3.413863	-0.881660
H	-2.049009	-3.414004	0.881669
C	-1.819086	-0.205185	0.000231
C	-1.578581	-1.565555	0.000244
Cl	1.067236	-1.346556	1.827456
Cl	1.068136	-1.343391	-1.829661
Fe	1.119618	-0.101643	-0.000078
Cl	2.618109	1.467988	0.002011

11a
B3LYP SCF energy: -1635.72451400 a.u.

B3LYP enthalpy: -1635.531501 a.u.
B3LYP free energy: -1635.603402 a.u.
M06 SCF energy in solution: -1635.51449776 a.u.
M06 enthalpy in solution: -1635.321485 a.u.
M06 free energy in solution: -1635.393386 a.u.

Cartesian coordinates

ATOM	X	Y	Z
C	-1.670059	-1.756069	0.491551
O	-1.071321	-2.787671	0.674997
C	-3.140324	-1.452546	0.771504
H	-3.345648	-1.631151	1.830616
H	-3.761982	-2.145139	0.196365
N	-1.053008	-0.594743	-0.053746
Fe	0.972877	-0.550559	-0.265240
C	-3.319611	0.012683	0.350606
H	-3.582935	0.667319	1.188230
H	-4.083117	0.151754	-0.421495
C	-2.406225	2.801772	-1.113810
H	-3.487144	2.663087	-0.960785
H	-2.070074	3.667164	-0.530390
H	-2.240549	3.043666	-2.172690
C	-1.940581	0.399005	-0.186368
C	-1.623713	1.628040	-0.730628
O	1.274850	1.266238	-0.601381
C	1.115660	2.254345	0.257128
O	0.009550	2.654905	0.625135
C	2.399491	2.866721	0.762894
H	2.194668	3.766720	1.344101
H	2.905970	2.125957	1.392165
H	3.061358	3.091587	-0.078588
Cl	1.614272	-1.789609	-1.948275
Cl	1.814743	-0.946550	1.729313

11-doublet
B3LYP SCF energy: -1635.70467498 a.u.
B3LYP enthalpy: -1635.511406 a.u.
B3LYP free energy: -1635.581928 a.u.
M06 SCF energy in solution: -1635.47594209 a.u.
M06 enthalpy in solution: -1635.282673 a.u.
M06 free energy in solution: -1635.353195 a.u.

Cartesian coordinates

ATOM X Y Z

C	1.198448	-1.982721	0.333374
O	0.312294	-2.790650	0.226877
C	2.666076	-2.229283	0.677848
H	3.072786	-2.974271	-0.011234
H	2.724374	-2.648785	1.686746
N	1.014619	-0.578953	0.131431
Fe	-0.724575	0.172765	-0.266469
C	3.336139	-0.852783	0.557084
H	4.092240	-0.815685	-0.234136
H	3.818620	-0.524246	1.483227
C	3.332581	2.417106	-0.018178
H	4.301904	1.995337	0.289902
H	3.445079	2.829191	-1.031233
H	3.090646	3.262628	0.638082
C	2.175798	0.075429	0.210818
C	2.247963	1.438554	0.004488
Cl	-0.202744	2.093366	0.873743
Cl	-0.437204	-0.212851	-2.421786
O	-2.588394	0.685215	-0.408528
C	-2.922177	-0.150813	0.512362
O	-1.983196	-0.852870	1.002764
C	-4.336709	-0.237817	0.987567
H	-4.499081	0.538144	1.744932
H	-5.024474	-0.055097	0.158602
H	-4.520698	-1.214775	1.438926

11-quartet
B3LYP SCF energy: -1635.72882557 a.u.
B3LYP enthalpy: -1635.535983 a.u.
B3LYP free energy: -1635.609982 a.u.
M06 SCF energy in solution: -1635.52171660 a.u.
M06 enthalpy in solution: -1635.328874 a.u.
M06 free energy in solution: -1635.402873 a.u.

Cartesian coordinates

ATOM	X	Y	Z
C	1.002397	-2.029018	0.150947
O	0.030786	-2.658972	0.491689
C	2.405035	-2.551286	-0.157525
H	2.349582	-3.236372	-1.008842
H	2.772941	-3.121878	0.699529
N	0.984207	-0.615576	-0.015313
Fe	-0.802996	0.444746	0.347853
C	3.226893	-1.288939	-0.452256

H	3.677732	-1.287959	-1.449688
H	4.033819	-1.125950	0.269750
C	3.634896	1.971433	-0.889183
H	4.533790	1.357650	-1.055260
H	3.441982	2.558786	-1.797105
H	3.852603	2.694602	-0.091019
C	2.192543	-0.164620	-0.339843
C	2.460454	1.176056	-0.542805
O	-1.530008	-0.673644	-1.328403
C	-2.706495	-0.383410	-0.948805
O	-2.826416	0.335329	0.105958
C	-3.923672	-0.869698	-1.677660
H	-4.621112	-0.040218	-1.824852
H	-3.647363	-1.313403	-2.635207
H	-4.428127	-1.620340	-1.059094
Cl	-0.142435	2.420250	-0.433935
Cl	-0.629601	0.326568	2.551795

11-sextet
B3LYP SCF energy: -1635.70258275 a.u.
B3LYP enthalpy: -1635.509423 a.u.
B3LYP free energy: -1635.581389 a.u.
M06 SCF energy in solution: -1635.47653454 a.u.
M06 enthalpy in solution: -1635.283375 a.u.
M06 free energy in solution: -1635.355341 a.u.

Cartesian coordinates

ATOM	X	Y	Z
C	2.285486	-1.176253	-0.000094
O	2.035807	-2.354009	0.000244
C	3.650724	-0.491622	-0.000816
H	4.208644	-0.815241	-0.884070
H	4.210786	-0.816820	0.880461
N	1.275905	-0.167338	-0.000049
Fe	-0.654715	-0.572480	0.000013
C	3.338844	1.013233	0.000612
H	3.727746	1.532306	-0.881119
H	3.726777	1.530220	0.884020
C	1.189806	3.631067	-0.000180
H	2.248646	3.933950	0.002974
H	0.706562	4.070128	-0.883585
H	0.700999	4.071028	0.879674
C	1.810077	1.052141	0.000103
C	1.026694	2.183018	-0.000003

O	-1.850984	1.287871	0.000181
C	-2.817130	0.482613	0.000138
O	-2.556747	-0.787294	-0.000013
C	-4.254131	0.915222	0.000043
H	-4.756028	0.505521	0.882416
H	-4.321952	2.004021	0.001502
H	-4.755031	0.508082	-0.884099
Cl	-0.470785	-0.920512	2.176859
Cl	-0.471141	-0.920452	-2.176875

11'
B3LYP SCF energy: -1635.72851079 a.u.
B3LYP enthalpy: -1635.535703 a.u.
B3LYP free energy: -1635.607001 a.u.
M06 SCF energy in solution: -1635.51506388 a.u.
M06 enthalpy in solution: -1635.322256 a.u.
M06 free energy in solution: -1635.393554 a.u.

Cartesian coordinates

ATOM	X	Y	Z
C	0.800977	-2.045627	0.367735
O	-0.197977	-2.396821	0.946398
C	1.992528	-2.890415	-0.065902
H	1.673493	-3.542360	-0.885672
H	2.309713	-3.530963	0.760596
N	1.043056	-0.678438	0.022532
Fe	-0.621553	0.613555	0.172807
C	3.045321	-1.861770	-0.495989
H	3.391646	-1.996884	-1.525372
H	3.932091	-1.861456	0.145480
C	2.762575	2.120680	-0.443464
H	2.259491	2.537993	-1.326247
H	2.128144	2.347890	0.427265
H	3.730854	2.622956	-0.325158
C	2.308305	-0.529573	-0.370205
C	2.935236	0.683638	-0.585179
O	-1.603803	-0.914398	-1.081154
C	-2.621175	-0.706460	-0.366340
O	-2.526127	0.172912	0.575487
C	-3.911340	-1.446408	-0.555538
H	-4.743278	-0.736841	-0.585489
H	-3.881677	-2.040103	-1.470254
H	-4.068250	-2.104947	0.305918
Cl	-0.489016	2.141089	-1.425461

Cl	0.069925	1.521464	2.095558

12-doublet
B3LYP SCF energy: -1635.76059753 a.u.
B3LYP enthalpy: -1635.564860 a.u.
B3LYP free energy: -1635.633429 a.u.
M06 SCF energy in solution: -1635.52253661 a.u.
M06 enthalpy in solution: -1635.326799 a.u.
M06 free energy in solution: -1635.395368 a.u.

Cartesian coordinates

ATOM	X	Y	Z
C	2.722513	-0.145730	-0.203073
O	3.206810	-1.250706	-0.236458
C	3.437187	1.204042	-0.231334
H	3.976843	1.301640	-1.178106
H	4.178207	1.236814	0.571561
N	1.319071	0.079966	-0.129847
Fe	0.003567	-1.380312	0.052069
C	2.312473	2.241704	-0.071528
H	2.280930	2.965635	-0.892008
H	2.401180	2.812084	0.859611
C	-0.604536	3.304111	0.180327
H	0.302117	3.891757	0.337110
H	-1.126488	3.674527	-0.707473
H	-1.281171	3.461504	1.026726
C	1.059364	1.384213	-0.053834
C	-0.255863	1.862575	0.044394
O	-3.022911	2.117240	-0.486185
C	-2.577642	1.071157	-0.110749
O	-1.186057	0.878961	0.091526
C	-3.291873	-0.206846	0.185457
H	-2.967903	-0.610412	1.149714
H	-4.366624	-0.024003	0.173415
H	-3.027470	-0.945681	-0.580829
Cl	-0.310200	-1.565114	2.236869
Cl	-0.777348	-2.004935	-1.918845

12-quartet
B3LYP SCF energy: -1635.80451513 a.u.
B3LYP enthalpy: -1635.608925 a.u.
B3LYP free energy: -1635.677837 a.u.

M06 SCF energy in solution: -1635.59067931 a.u.
M06 enthalpy in solution: -1635.395089 a.u.
M06 free energy in solution: -1635.464001 a.u.

Cartesian coordinates

ATOM	X	Y	Z
C	2.734944	-0.619496	-0.174011
O	3.014243	-1.789006	-0.270436
C	3.686231	0.578749	-0.147315
H	4.250377	0.606504	-1.084083
H	4.407715	0.449299	0.663819
N	1.395787	-0.147964	-0.064989
Fe	-0.323966	-1.278977	0.003239
C	2.768267	1.798838	0.039128
H	2.862970	2.532376	-0.768028
H	2.958336	2.327624	0.979615
C	0.079047	3.350451	0.353170
H	1.068597	3.755346	0.575183
H	-0.320416	3.844884	-0.538060
H	-0.598459	3.596765	1.177159
C	1.383577	1.175242	0.046807
C	0.170871	1.876665	0.165399
O	-2.454182	2.636115	-0.498836
C	-2.254956	1.532440	-0.085789
O	-0.925404	1.069786	0.179500
C	-3.229929	0.439624	0.200694
H	-2.996214	-0.057456	1.146490
H	-4.235331	0.861995	0.209404
H	-3.152574	-0.315033	-0.592005
Cl	-0.870454	-1.854355	2.083443
Cl	-1.170602	-1.642315	-2.028685

12-sextet
B3LYP SCF energy: -1635.80233138 a.u.
B3LYP enthalpy: -1635.606787 a.u.
B3LYP free energy: -1635.676784 a.u.
M06 SCF energy in solution: -1635.58914153 a.u.
M06 enthalpy in solution: -1635.393597 a.u.
M06 free energy in solution: -1635.463594 a.u.

Cartesian coordinates

ATOM	X	Y	Z
C	2.744046	-0.597211	-0.226743
O	3.043696	-1.763124	-0.313294

C	3.676478	0.617306	-0.219848
H	4.207708	0.662678	-1.175273
H	4.426930	0.495465	0.565374
N	1.402252	-0.146215	-0.107875
Fe	-0.341234	-1.294815	0.085730
C	2.742514	1.819004	0.002060
H	2.822992	2.578507	-0.782079
H	2.926932	2.321549	0.958311
C	0.033951	3.334000	0.308030
H	1.012560	3.743613	0.566758
H	-0.345343	3.842490	-0.584132
H	-0.668210	3.557923	1.117991
C	1.367794	1.173925	0.000147
C	0.143930	1.864690	0.098125
O	-2.462180	2.607726	-0.659978
C	-2.269731	1.508904	-0.229791
O	-0.948932	1.051435	0.069054
C	-3.248981	0.417991	0.051635
H	-3.063591	-0.023732	1.035536
H	-4.257614	0.826849	-0.015491
H	-3.119874	-0.377820	-0.692669
Cl	-0.804143	-1.549970	2.251606
Cl	-1.142142	-1.917016	-1.894907

13
B3LYP SCF energy: -1867.41224746 a.u.
B3LYP enthalpy: -1867.272772 a.u.
B3LYP free energy: -1867.333263 a.u.
M06 SCF energy in solution: -1867.23937278 a.u.
M06 enthalpy in solution: -1867.099897 a.u.
M06 free energy in solution: -1867.160388 a.u.

Cartesian coordinates

ATOM	X	Y	Z
C	-2.556531	0.838913	0.001552
O	-2.851872	2.006574	0.003893
C	-3.503516	-0.377530	-0.001127
H	-4.146153	-0.316722	-0.883798
H	-4.147845	-0.319616	0.880474
N	-1.231973	0.336743	0.000597
C	-2.595012	-1.626396	-0.002537
H	-2.732901	-2.257715	-0.886121
H	-2.732797	-2.259388	0.879888
C	0.827656	-2.698572	-0.005326

H	0.134370	-3.550466	-0.007222
H	1.481376	-2.757392	-0.884512
H	1.481151	-2.761133	0.873778
C	-1.218128	-1.000522	-0.001795
C	0.098823	-1.429689	-0.002725
Cl	0.705926	0.611937	2.168495
Cl	2.976154	0.103991	0.000141
Fe	0.814912	0.417526	0.000801
Cl	0.705809	0.620528	-2.166099

14
B3LYP SCF energy: -1867.43525650 a.u.
B3LYP enthalpy: -1867.296504 a.u.
B3LYP free energy: -1867.359267 a.u.
M06 SCF energy in solution: -1867.28728427 a.u.
M06 enthalpy in solution: -1867.148532 a.u.
M06 free energy in solution: -1867.211295 a.u.

Cartesian coordinates

ATOM	X	Y	Z
C	3.156268	-0.589538	-0.000013
O	3.764555	-1.629654	-0.000038
C	3.752742	0.827602	-0.000127
H	4.387481	0.950143	-0.882273
H	4.387875	0.950199	0.881723
N	1.724400	-0.518869	0.000145
C	2.532206	1.752310	0.000074
H	2.474626	2.396925	-0.883123
H	2.474894	2.396798	0.883384
C	-0.503538	2.540605	0.000283
H	0.333824	3.249832	0.000390
H	-1.129276	2.725889	-0.879601
H	-1.129384	2.725742	0.880122
C	1.375113	0.739817	0.000298
C	0.019093	1.153685	0.000199
Cl	-0.867742	-1.214178	1.927644
Cl	-3.181996	0.706908	0.000041
Fe	-1.245484	-0.308355	-0.000043
Cl	-0.867675	-1.213662	-1.927949

15
B3LYP SCF energy: -2096.48779671 a.u.

B3LYP enthalpy: -2096.277440 a.u.
B3LYP free energy: -2096.355930 a.u.
M06 SCF energy in solution: -2096.26512810 a.u.
M06 enthalpy in solution: -2096.054771 a.u.
M06 free energy in solution: -2096.133261 a.u.

Cartesian coordinates

ATOM	X	Y	Z
C	2.310932	-1.386553	0.265304
O	2.249590	-0.833365	1.343934
C	1.231745	-2.299845	-0.292006
H	0.644393	-2.661428	0.553149
H	1.658050	-3.159312	-0.823456
C	0.310042	-1.526933	-1.271130
H	-0.527457	-2.169242	-1.553917
H	0.857406	-1.263686	-2.183234
C	-0.169699	-0.271882	-0.662374
C	-0.109663	0.828387	-0.110275
C	0.047670	2.148951	0.478511
H	-0.552202	2.889016	-0.060675
H	-0.247153	2.149268	1.531580
H	1.109214	2.413056	0.393478
N	3.303011	-1.087397	-0.668661
H	3.672510	-1.830141	-1.250639
O	4.390755	-0.355117	-0.161072
C	4.114245	0.993136	-0.048896
O	3.097392	1.506085	-0.435871
C	5.261449	1.665821	0.654431
H	5.139647	1.519728	1.733677
H	6.220474	1.232144	0.361113
H	5.238747	2.734003	0.435246
Fe	-2.242872	0.003248	-0.076018
Cl	-2.874347	0.951298	-1.920023
Cl	-2.177782	-1.940867	0.951502
Cl	-3.412710	1.120546	1.407547

16

B3LYP SCF energy: -2096.46165528 a.u.
B3LYP enthalpy: -2096.252981 a.u.
B3LYP free energy: -2096.327110 a.u.
M06 SCF energy in solution: -2096.25125952 a.u.
M06 enthalpy in solution: -2096.042585 a.u.
M06 free energy in solution: -2096.116714 a.u.
Imaginary frequency: -271.9558 cm-1

Cartesian coordinates

ATOM	X	Y	Z
C	2.972081	-1.414009	-0.102531
O	4.160000	-1.465018	-0.022331
C	1.894296	-2.230349	0.549658
H	1.780149	-1.827162	1.563714
H	2.217095	-3.270993	0.629255
C	0.576707	-2.055279	-0.220497
H	-0.275995	-2.346701	0.397665
H	0.553047	-2.676311	-1.124903
C	0.417663	-0.629966	-0.575075
C	-0.340326	0.408825	-0.670256
C	-0.142016	1.789834	-1.200964
H	-0.931133	2.023560	-1.919918
H	-0.228990	2.521580	-0.392407
H	0.830369	1.887734	-1.694483
N	2.309121	-0.368437	-1.011086
H	2.427818	-0.622733	-1.994482
O	2.929247	0.895885	-0.942161
C	2.900904	1.414167	0.352749
O	2.515440	0.757384	1.280738
C	3.389341	2.830154	0.338395
H	4.245880	2.942610	-0.330837
H	2.585195	3.480042	-0.025172
H	3.653448	3.122181	1.355037
Fe	-2.110285	0.021123	0.151836
Cl	-2.573289	-1.478511	-1.448872
Cl	-1.579692	-0.782893	2.162237
Cl	-3.200119	1.944333	0.069432

8a

B3LYP SCF energy: -1635.65188692 a.u.
B3LYP enthalpy: -1635.460064 a.u.
B3LYP free energy: -1635.539955 a.u.
M06 SCF energy in solution: -1635.43162806 a.u.
M06 enthalpy in solution: -1635.239805 a.u.
M06 free energy in solution: -1635.319696 a.u.

Cartesian coordinates

ATOM	X	Y	Z
C	0.987918	1.900954	-0.516664
O	0.966810	3.083907	-0.862808
C	2.169748	1.170784	0.050859

H	1.845868	0.477723	0.830786
H	2.841822	1.914853	0.486502
N	-0.202408	1.243154	-0.791402
Fe	-1.303912	-0.099058	0.184360
C	2.901913	0.379988	-1.069906
H	3.202566	1.079797	-1.860629
H	2.200536	-0.333557	-1.520241
C	6.167368	-1.608913	0.481883
H	7.094592	-1.314718	-0.024330
H	6.288040	-1.392378	1.550067
H	6.056761	-2.694356	0.372974
C	4.063875	-0.325339	-0.542177
C	5.015503	-0.903279	-0.071466
O	-3.057646	-1.058476	0.648833
C	-3.590090	-0.600597	-0.417355
O	-2.853569	0.156820	-1.141546
C	-4.985958	-0.954918	-0.819738
H	-4.945490	-1.832644	-1.475896
H	-5.581271	-1.206496	0.059962
H	-5.438172	-0.131959	-1.377302
Cl	-0.952667	1.043704	2.040818
Cl	0.066014	-1.779171	-0.159870

8-doublet
B3LYP SCF energy: -1635.63271248 a.u.
B3LYP enthalpy: -1635.440664 a.u.
B3LYP free energy: -1635.514071 a.u.
M06 SCF energy in solution: -1635.39289885 a.u.
M06 enthalpy in solution: -1635.200850 a.u.
M06 free energy in solution: -1635.274257 a.u.

Cartesian coordinates

ATOM	X	Y	Z
C	-2.023758	-0.965350	-1.020660
O	-2.061521	-1.544158	-2.095676
C	-3.163294	-0.814872	-0.041245
H	-2.786425	-0.998734	0.969113
H	-3.933869	-1.550690	-0.284127
N	-0.862558	-0.270796	-0.688291
Fe	0.741816	-0.397090	0.246148
C	-3.715317	0.632078	-0.144333
H	-4.417763	0.812764	0.677389
H	-4.277584	0.740851	-1.079958
C	-0.532777	3.267928	-0.045460

H	-0.202465	3.422008	0.986552
H	0.314090	2.854166	-0.602517
H	-0.802624	4.234949	-0.485924
C	-2.617876	1.597499	-0.106522
C	-1.659547	2.348252	-0.088380
O	2.625836	-0.967867	-0.296568
C	2.746721	0.147613	-0.889190
O	1.702348	0.904243	-0.878887
C	4.017021	0.595180	-1.534648
H	4.577600	1.201445	-0.813047
H	4.622854	-0.269613	-1.811628
H	3.800565	1.215497	-2.407632
Cl	0.001956	-2.353095	0.971626
Cl	0.787218	0.739540	2.126118

8-quartet
B3LYP SCF energy: -1635.66142473 a.u.
B3LYP enthalpy: -1635.469464 a.u.
B3LYP free energy: -1635.544694 a.u.
M06 SCF energy in solution: -1635.44102870 a.u.
M06 enthalpy in solution: -1635.249068 a.u.
M06 free energy in solution: -1635.324298 a.u.

Cartesian coordinates

ATOM	X	Y	Z
C	-2.083182	-0.846112	-0.906989
O	-2.328115	-1.393302	-1.972635
C	-3.019064	-0.740992	0.272098
H	-2.454108	-0.883788	1.197921
H	-3.789467	-1.511280	0.186389
N	-0.882499	-0.137198	-0.825814
Fe	0.739108	-0.509145	0.174097
C	-3.644087	0.682421	0.258686
H	-4.165267	0.860169	1.206303
H	-4.392467	0.742645	-0.540775
C	-0.516205	3.302040	-0.368784
H	-0.161589	3.699825	0.588897
H	0.311709	2.750209	-0.829009
H	-0.782175	4.141422	-1.021192
C	-2.599060	1.681001	0.044637
C	-1.649671	2.413951	-0.162669
O	2.794969	-0.499981	0.309398
C	2.867054	0.487094	-0.494588
O	1.764817	0.917984	-0.977799

C	4.178678	1.129478	-0.826238
H	4.414363	1.862764	-0.045689
H	4.972382	0.379239	-0.836013
H	4.119245	1.646265	-1.785925
Cl	0.436129	-2.693419	0.075179
Cl	0.141983	0.320465	2.122740

8-sextet
B3LYP SCF energy: -1635.64068021 a.u.
B3LYP enthalpy: -1635.448185 a.u.
B3LYP free energy: -1635.522263 a.u.
M06 SCF energy in solution: -1635.41031867 a.u.
M06 enthalpy in solution: -1635.217823 a.u.
M06 free energy in solution: -1635.291901 a.u.

Cartesian coordinates

ATOM	X	Y	Z
C	2.197553	-1.127437	0.109355
O	2.183377	-1.960419	1.007421
C	3.409552	-0.757962	-0.718529
H	3.140362	-0.822419	-1.778679
H	4.204807	-1.476417	-0.505028
N	1.045142	-0.414886	-0.179483
Fe	-0.736964	-0.490640	0.225442
C	3.859262	0.687415	-0.390059
H	4.668164	0.975342	-1.072114
H	4.274632	0.713564	0.625412
C	0.578414	3.186348	-0.583924
H	-0.290120	2.620890	-0.939659
H	0.343203	3.568189	0.415654
H	0.734110	4.039592	-1.255017
C	2.736474	1.617000	-0.489807
C	1.757178	2.334271	-0.542526
O	-2.670206	-0.464441	0.534796
C	-2.861642	0.463150	-0.340216
O	-1.843482	0.909184	-0.947712
C	-4.251195	0.953620	-0.608850
H	-4.827370	0.148689	-1.078057
H	-4.741916	1.204185	0.336127
H	-4.227129	1.821240	-1.269656
Cl	-0.766194	-2.338143	-0.973119
Cl	-0.254086	0.623729	2.064130

9

B3LYP SCF energy: -1867.37617174 a.u.
B3LYP enthalpy: -1867.238740 a.u.
B3LYP free energy: -1867.304739 a.u.
M06 SCF energy in solution: -1867.22216590 a.u.
M06 enthalpy in solution: -1867.084734 a.u.
M06 free energy in solution: -1867.150733 a.u.

Cartesian coordinates

ATOM	X	Y	Z
C	-1.442645	-1.690733	-0.163714
O	-0.883085	-2.528990	-0.865832
N	-0.695873	-0.634925	0.323525
Fe	1.128582	-0.012159	0.085359
Cl	1.354552	1.804880	1.303922
Cl	2.501237	-1.607932	0.632808
Cl	1.046045	0.462078	-2.060011
C	-2.920391	-1.686404	0.175754
H	-3.461991	-1.707776	-0.776549
H	-3.170618	-2.608056	0.711318
C	-3.286499	-0.428565	0.988913
C	-2.591755	0.732158	0.426164
C	-2.109813	1.737454	-0.078551
C	-1.462921	2.894768	-0.668529
H	-2.187742	3.506530	-1.219213
H	-0.687958	2.564889	-1.371582
H	-0.981678	3.505938	0.100986
H	-2.993860	-0.555333	2.037994
H	-4.370519	-0.269177	0.975237

TS1

B3LYP SCF energy: -1635.65540372 a.u.
B3LYP enthalpy: -1635.464974 a.u.
B3LYP free energy: -1635.538978 a.u.
M06 SCF energy in solution: -1635.43726703 a.u.
M06 enthalpy in solution: -1635.246837 a.u.
M06 free energy in solution: -1635.320841 a.u.
Imaginary frequency: -295.4744 cm^{-1}

Cartesian coordinates

ATOM	X	Y	Z
C	2.332088	-0.936616	-0.435927
O	2.376964	-2.025423	0.116387

C	3.483867	-0.223813	-1.116836
H	3.260212	-0.137529	-2.186931
H	4.406250	-0.794473	-0.993918
N	1.171275	-0.164075	-0.440086
Fe	-0.703890	-0.606637	0.193714
C	3.553699	1.177532	-0.476833
H	4.091402	1.884487	-1.117190
H	4.096310	1.119423	0.474616
C	0.113263	3.227086	0.470839
H	0.312679	4.306171	0.470784
H	-0.682934	3.013677	-0.250999
H	-0.230340	2.933004	1.469358
C	2.186514	1.680427	-0.210880
C	1.301388	2.478885	0.111564
O	-1.424110	0.879645	-1.088706
C	-2.609911	0.711921	-0.623721
O	-2.756525	-0.126168	0.314253
C	-3.774162	1.461877	-1.201553
H	-4.600914	1.489037	-0.489657
H	-3.473604	2.472936	-1.487473
H	-4.107309	0.941792	-2.107363
Cl	-0.152512	-0.126210	2.286863
Cl	-0.909881	-2.673534	-0.497015

TS1'

B3LYP SCF energy: -1867.37434530 a.u.
B3LYP enthalpy: -1867.237826 a.u.
B3LYP free energy: -1867.303064 a.u.
M06 SCF energy in solution: -1867.21933886 a.u.
M06 enthalpy in solution: -1867.082820 a.u.
M06 free energy in solution: -1867.148058 a.u.
Imaginary frequency: -270.8222 cm^{-1}

Cartesian coordinates

ATOM	X	Y	Z
C	-1.331989	-1.632428	-0.684373
O	-1.108138	-2.427760	-1.575505
C	-2.362100	-1.726250	0.413077
H	-1.832673	-1.754845	1.372563
H	-2.959991	-2.632681	0.301789
N	-0.656632	-0.391071	-0.680555
C	-3.204533	-0.434786	0.318809
H	-3.677859	-0.201818	1.277858
H	-4.003233	-0.565056	-0.420984

C	-1.479310	3.192211	-0.557964
H	-1.897350	3.643718	-1.463989
H	-1.655928	3.864123	0.290888
H	-0.391322	3.093243	-0.682285
C	-2.352091	0.705049	-0.093603
C	-2.048682	1.886935	-0.299567
Cl	1.957493	1.826419	-0.781740
Cl	0.387308	0.245452	2.153912
Fe	1.071813	0.001627	0.065791
Cl	2.283726	-1.793861	-0.171982

TS2

B3LYP SCF energy: -1635.71943391 a.u.
B3LYP enthalpy: -1635.527697 a.u.
B3LYP free energy: -1635.598972 a.u.
M06 SCF energy in solution: -1635.51248128 a.u.
M06 enthalpy in solution: -1635.320744 a.u.
M06 free energy in solution: -1635.392019 a.u.
Imaginary frequency: -189.7350 cm^{-1}

Cartesian coordinates

ATOM	X	Y	Z
C	2.556934	-0.910263	-0.118628
O	2.551080	-2.114196	-0.182738
C	3.759256	0.031659	-0.105211
H	4.355060	-0.135478	-1.006946
H	4.391399	-0.212312	0.753518
N	1.360308	-0.136712	-0.041198
Fe	-0.539321	-0.900257	0.032633
C	3.149212	1.438289	-0.028927
H	3.395527	2.061366	-0.894872
H	3.453452	1.992668	0.864741
C	0.664373	3.602092	0.165310
H	1.677884	4.031619	0.165407
H	0.087078	4.017423	-0.669669
H	0.134346	3.925283	1.070463
C	1.644160	1.170837	0.008988
C	0.670520	2.146637	0.086611
O	-1.134032	0.869804	0.204495
C	-2.255324	1.600992	0.029142
O	-2.224412	2.806007	-0.093118
C	-3.513802	0.760921	0.010409
H	-4.386506	1.410924	-0.066357
H	-3.481473	0.074726	-0.843178

H	-3.573092	0.152221	0.918677
Cl	-0.888697	-2.057236	1.863310
Cl	-1.178748	-1.736569	-1.896671

TS3
B3LYP SCF energy: -1635.62938681 a.u.
M06 SCF energy in solution: -1635.41289087 a.u.
Imaginary frequency: -177.9875 cm^{-1}

Cartesian coordinates

ATOM	X	Y	Z
C	-2.572321	-0.003911	-0.579746
O	-3.246918	-0.640434	-1.378152
C	-3.081509	0.977482	0.452271
H	-2.798157	0.613938	1.445920
H	-4.168756	1.061321	0.390882
N	-1.189098	-0.024058	-0.673348
Fe	0.314257	-0.978389	0.058218
C	-2.364747	2.313697	0.160889
H	-2.456471	2.999277	1.010900
H	-2.837731	2.801129	-0.701281
C	1.394093	3.360884	-0.291037
H	1.022724	4.382770	-0.144005
H	2.116957	3.141391	0.500230
H	1.909858	3.316471	-1.255051
C	-0.931355	2.099141	-0.144050
C	0.261975	2.436493	-0.259800
O	1.395129	0.762711	-0.678609
C	2.480429	0.242699	-0.214927
O	2.388808	-0.898041	0.323416
C	3.804983	0.946179	-0.294736
H	4.599461	0.208894	-0.428461
H	3.817579	1.672236	-1.109262
H	3.992814	1.468329	0.650810
Cl	0.169931	-3.009240	-0.764759
Cl	-0.363503	-0.760709	2.160797

TS4
B3LYP SCF energy: -1635.63295726 a.u.
M06 SCF energy in solution: -1635.41272660 a.u.
Imaginary frequency: -183.7243 cm^{-1}

Cartesian coordinates

ATOM	X	Y	Z
C	-2.393023	0.176247	-0.725067
O	-2.949749	1.207504	-0.358168
C	-3.080917	-1.088272	-1.190909
H	-2.715935	-1.357367	-2.189027
H	-4.162521	-0.943229	-1.228556
N	-1.034265	0.015003	-0.555049
Fe	0.356951	1.110141	0.152345
C	-2.657876	-2.166343	-0.167770
H	-2.913427	-3.169925	-0.525941
H	-3.200637	-2.005122	0.772367
C	0.956606	-3.156947	1.216150
H	0.471547	-3.868087	1.896997
H	1.656987	-3.685774	0.567120
H	1.512413	-2.433629	1.823710
C	-1.203633	-2.092712	0.084959
C	-0.074268	-2.465648	0.451064
O	1.440870	-1.623306	-0.772115
C	2.253501	-0.693053	-0.905441
O	2.016484	0.547449	-0.593962
C	3.634121	-0.945157	-1.472697
H	4.383786	-0.708613	-0.709708
H	3.736295	-1.985556	-1.783835
H	3.811145	-0.275764	-2.319892
Cl	0.308810	0.598514	2.295586
Cl	0.085988	3.225164	-0.314800

7. References

(1) Paterson, I.; Paquet, T. Total Synthesis and Configurational Validation of (+)-Phorbaside A. *Org. Lett.* **2010**, *12*, 2158–2161.
(2) Bhunia, S.; Ghorpade, S.; Huple, D. B.; Liu, R.-S. Gold-Catalyzed Oxidative Cyclizations of Cis-3-En-1-Ynes To Form Cyclopentenone Derivatives. *Angew. Chem. Int. Ed.* **2012**, *51*, 2939–2942.
(3) Kálai, T.; Bognár, B.; Zsolnai, D.; Berente, Z.; Hideg, K. Synthesis of Nitroxide-Annulated Carbocycles and Heterocycles. *Synthesis* **2012**, *44*, 3655–3660.
(4) Chen, L.; Riaz Ahmed, K. B.; Huang, P.; Jin, Z. Design, Synthesis, and Biological Evaluation of Truncated Superstolide A. *Angew. Chem. Int. Ed.* **2013**, *52*, 3446–3449.
(5) Falck, J. R.; He, A.; Fukui, H.; Tsutsui, H.; Radha, A. Synthesis and Stereochemical Assignment of FR252921, a Promising Immunosuppressant. *Angew. Chem. Int. Ed.* **2007**, *46*, 4527–4529.
(6) Girard, A.-L.; Lhermet, R.; Fressigné, C.; Durandetti, M.; Maddaluno, J. Influence of the Acetylenic Substituent on the Intramolecular Carbolithiation of Alkynes. *Eur. J. Org. Chem.* **2012**, *2012*, 2895–2905.
(7) Gupta, S.; Su, S.; Zhang, Y.; Liu, P.; Wink, D. J.; Lee, D. Ruthenabenzene: A Robust Precatalyst. *J. Am. Chem. Soc.* **2021**. *143*, 7490–7500.
(8) Soltani, Y.; Wilkins, L. C.; Melen, R. L. Stoichiometric and Catalytic C−C and C−H Bond Formation with $B(C_6F_5)_3$ via Cationic Intermediates. *Angew. Chem. Int. Ed.* **2017**, *56*, 11995–11999.
(9) Liu, Z.; Du, Q.; Zhai, H.; Li, Y. Relay Catalysis of Rh (II) and Cobaloxime: Stereoselective Synthesis of Spiroindanones from N-Sulfonyl-1,2,3-Triazoles. *Org. Lett.* **2018**, *20*, 7514–7517.
(10) Yamada, T.; Park, K.; Tachikawa, T.; Fujii, A.; Rudolph, M.; Hashmi, A. S. K.; Sajiki, H. Gold-Catalyzed Cyclization of 2-Alkynylaldehyde Cyclic Acetals via Hydride Shift for the Synthesis of Indenone Derivatives. *Org. Lett.* **2020**, *22*, 1883–1888.
(11) Hong, S. Y.; Son, J.; Kim, D.; Chang, S. Ir(III)-Catalyzed Stereoselective Haloamidation of Alkynes Enabled by Ligand Participation. *J. Am. Chem. Soc.* **2018**, *140*, 12359–12363.
(12) Frisch, M. J.; Trucks, G. W.; Schlegel, H. B.; Scuseria, G. E.; Robb, M. A.; Cheeseman, J. R.; Scalmani, G.; Barone, V.; Petersson, G. A.; Nakatsuji, H.; Li, X.; Caricato, M.; Marenich, A. V.; Bloino, J.; Janesko, B. G.; Gomperts, R.; Mennucci, B.; Hratchian, H. P.; Ortiz, J. V.; Izmaylov, A. F.; Sonnenberg, J. L.; Williams; Ding, F.; Lipparini, F.; Egidi, F.; Goings, J.; Peng, B.; Petrone, A.; Henderson, T.; Ranasinghe, D.; Zakrzewski, V. G.; Gao, J.; Rega, N.; Zheng, G.; Liang, W.; Hada, M.; Ehara, M.; Toyota, K.; Fukuda, R.; Hasegawa, J.; Ishida, M.; Nakajima, T.; Honda, Y.; Kitao, O.; Nakai, H.; Vreven, T.; Throssell, K.; Montgomery Jr., J. A.; Peralta, J. E.; Ogliaro, F.; Bearpark, M. J.; Heyd, J. J.; Brothers, E. N.; Kudin, K. N.; Staroverov, V. N.; Keith, T. A.; Kobayashi, R.; Normand, J.; Raghavachari, K.; Rendell, A. P.; Burant, J. C.; Iyengar, S. S.; Tomasi, J.; Cossi, M.; Millam, J. M.; Klene, M.; Adamo, C.; Cammi, R.; Ochterski, J. W.; Martin, R. L.; Morokuma, K.; Farkas, O.; Foresman, J. B.; Fox, D. J. Gaussian 16 Rev. C.01, Wallingford, CT, 2016.
(13) Lee, C.; Yang, W.; Parr, R. G. Development of the Colle-Salvetti correlation-energy formula into a functional of the electron density. *Phys. Rev. B* **1988**, *37*, 785-789.
(14) Becke, A. D. Density-functional thermochemistry. III. The role of exact exchange. *J. Chem. Phys.* **1993**, *98*, 5648-5652.
(15) Grimme, S.; Ehrlich, S.; and Goerigk, L. Effect of the damping function in dispersion corrected density functional theory. *J. Comp. Chem.* **2011**, *32*, 1456-1465.

(16) Zhao, Y.; Truhlar, D. G. The M06 suite of density functionals for main group thermochemistry, thermochemical kinetics, noncovalent interactions, excited states, and transition elements: two new functionals and systematic testing of four M06-class functionals and 12 other functionals. *Theor. Chem. Acc.* **2008**, *120*, 215-241.
(17) Marenich, A. V.; Cramer, C. J.; Truhlar, D. G. Universal solvation model based on solute electron density and on a continuum model of the solvent defined by the bulk dielectric constant and atomic surface tensions. *J. Phys. Chem. B* **2009**, *113*, 6378-6396.

8. ^{1}H and ^{13}C NMR Spectra

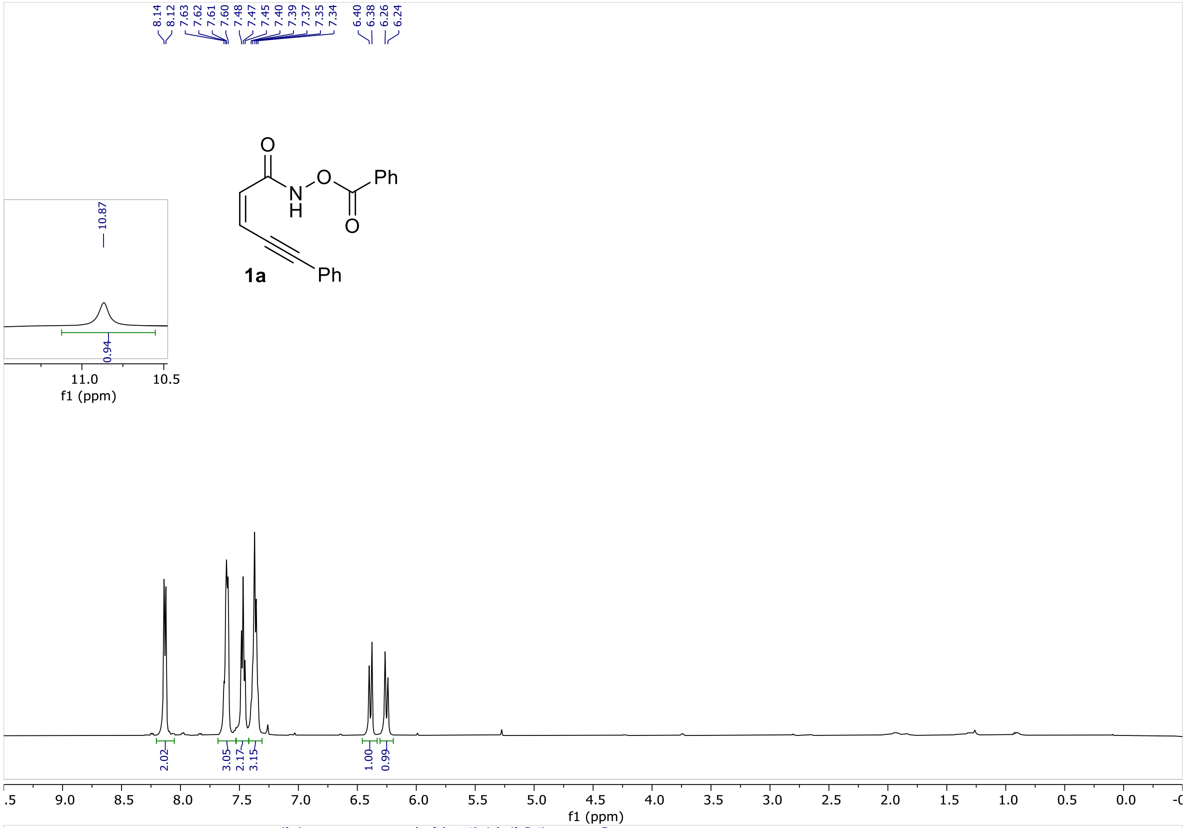

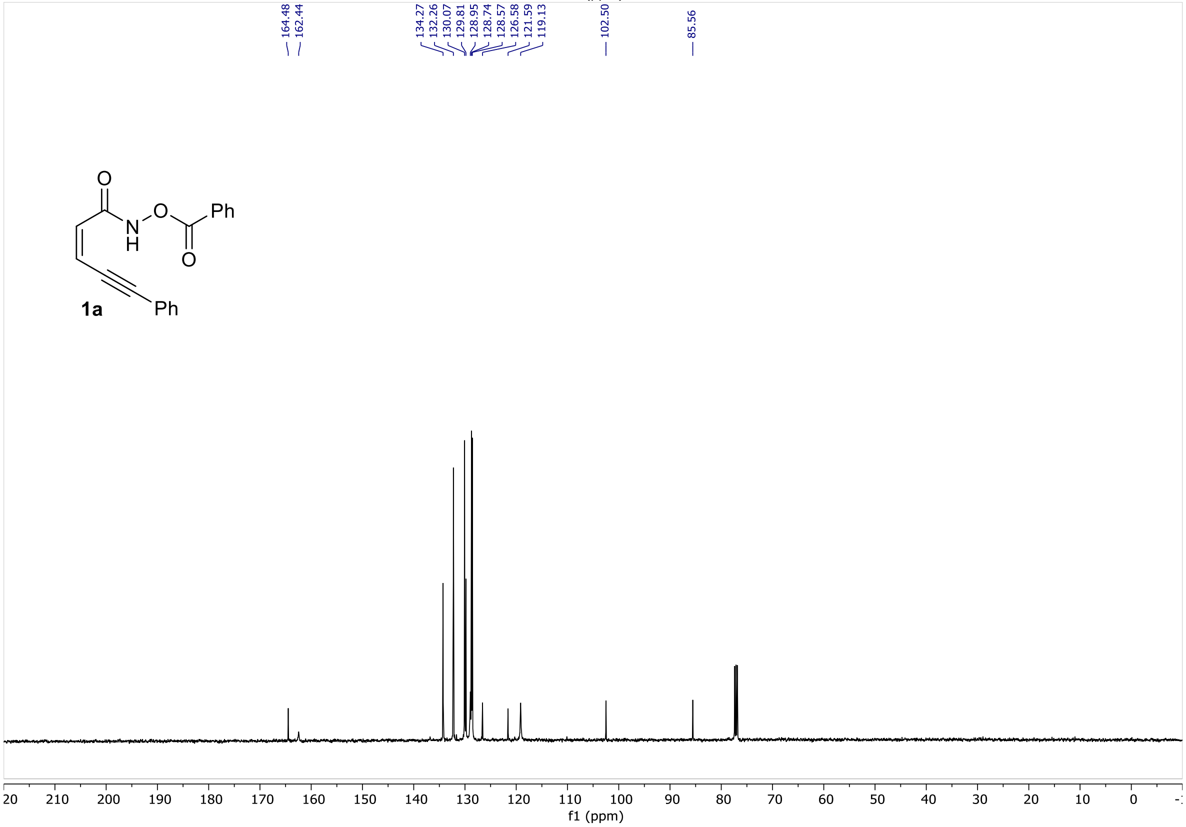

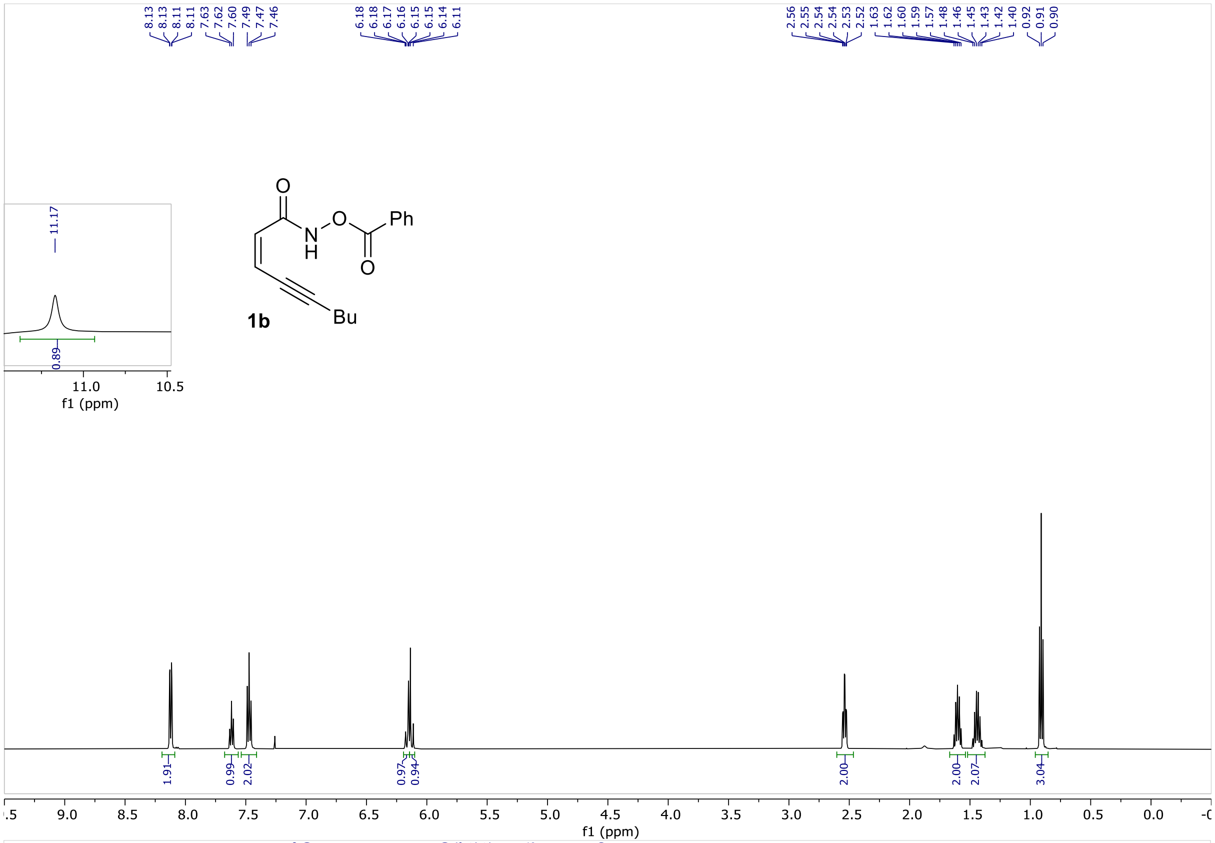
1b
Ph
Bu
f1 (ppm)

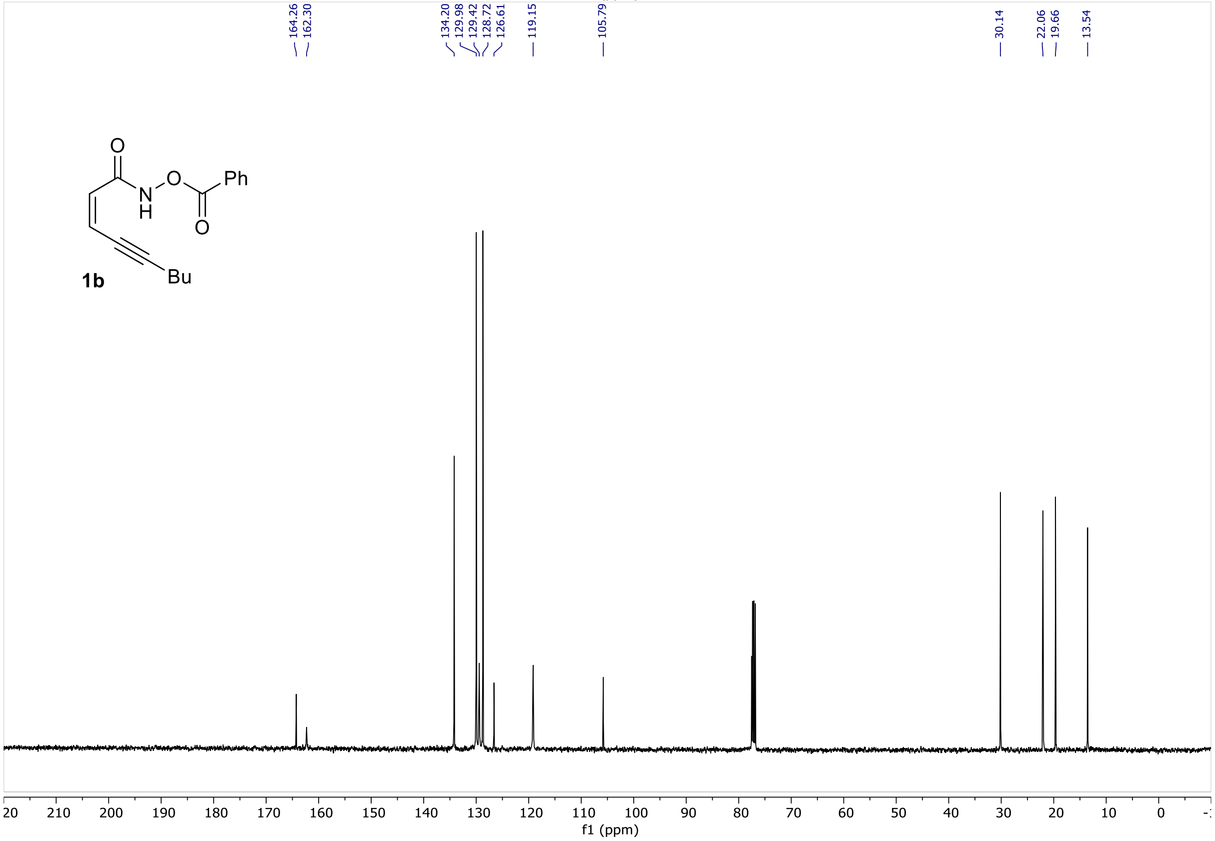
1b
Ph
Bu
f1 (ppm)

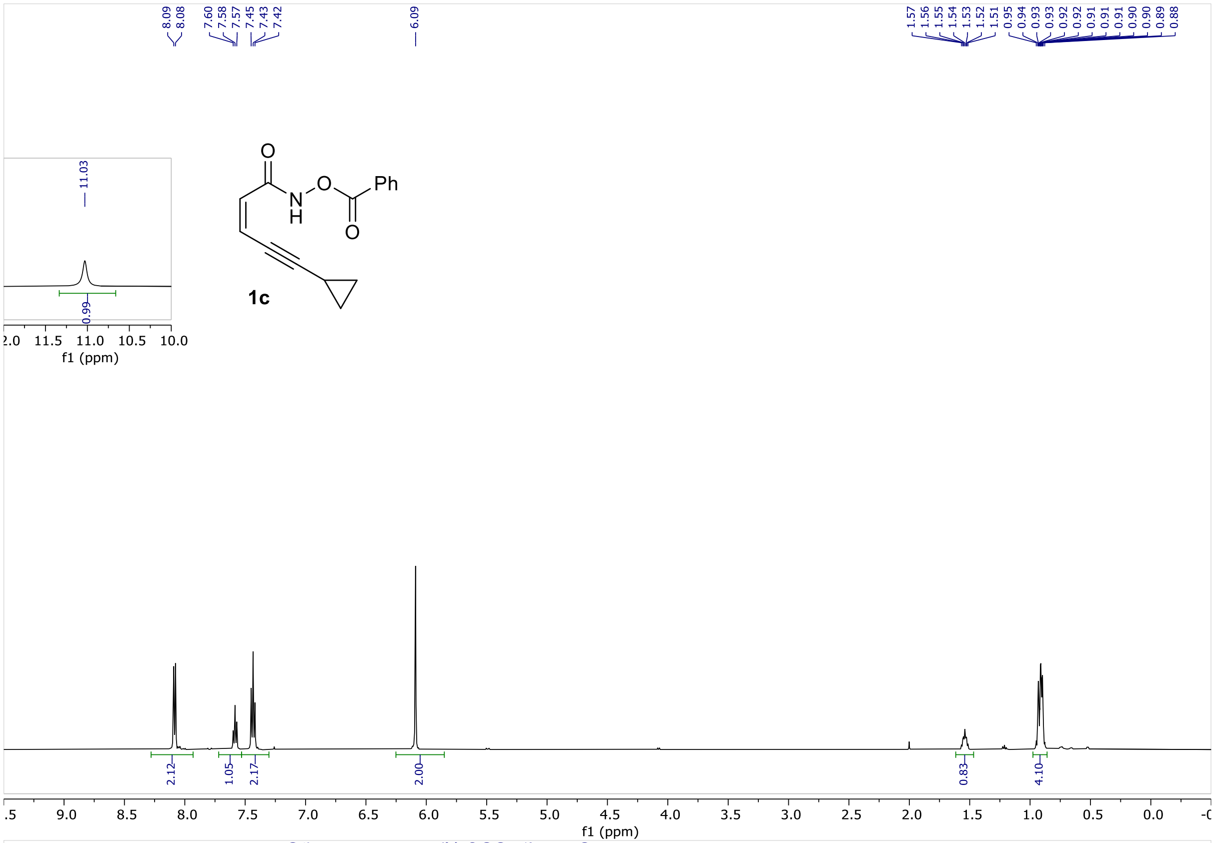

1c
f1 (ppm)

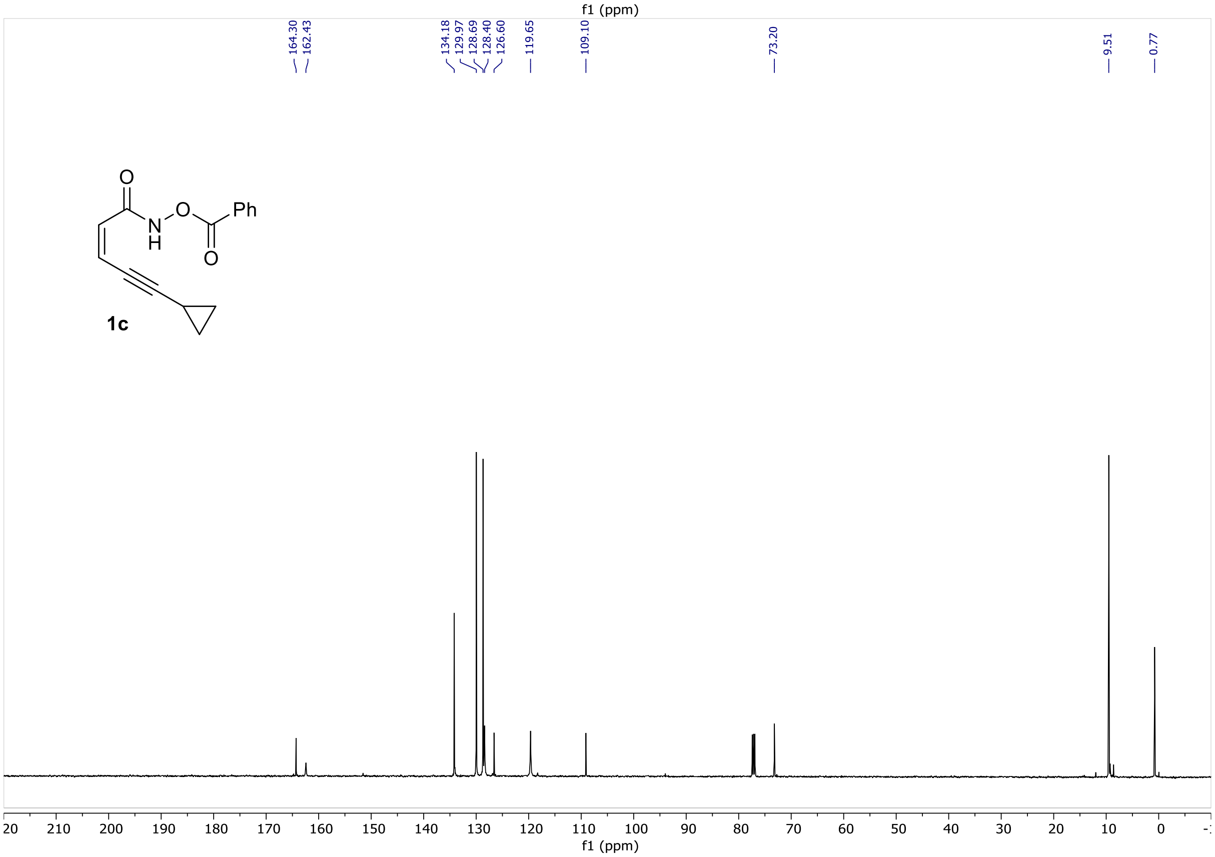

1c
f1 (ppm)

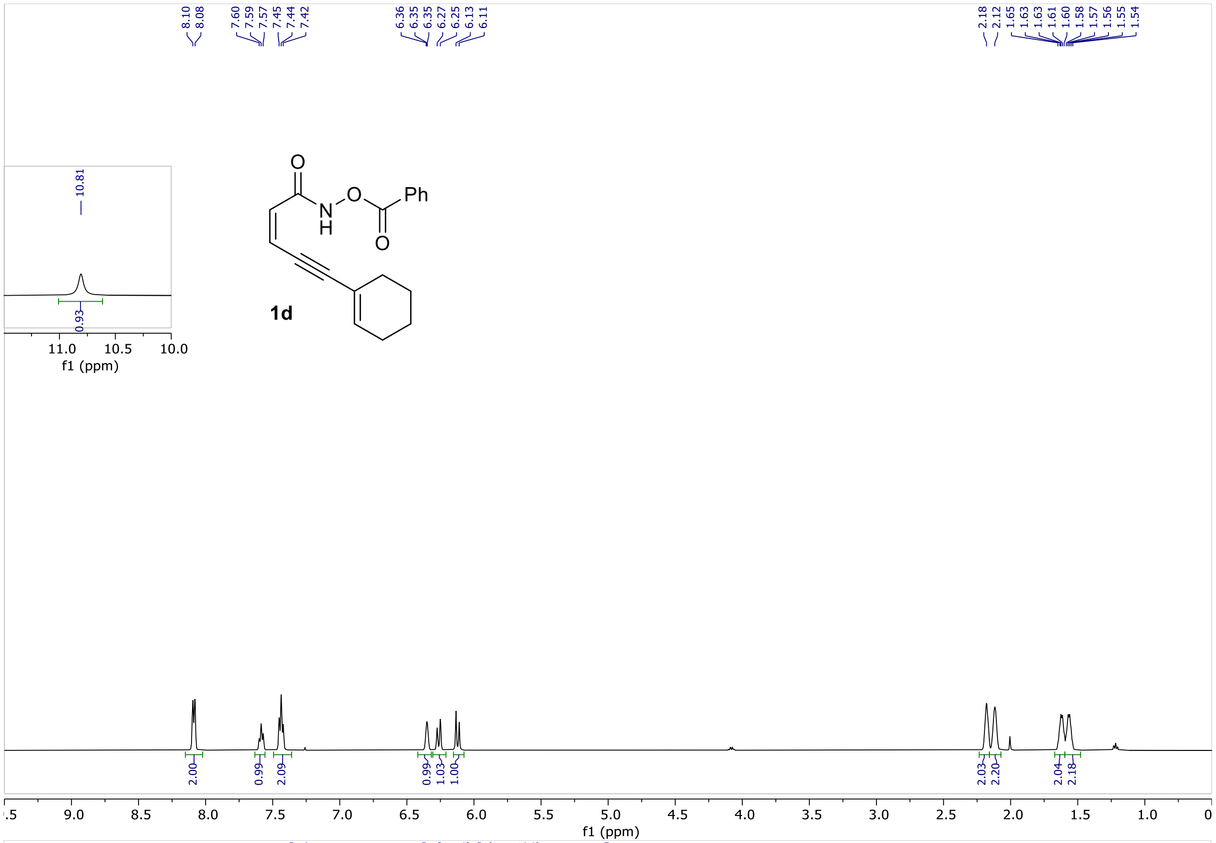

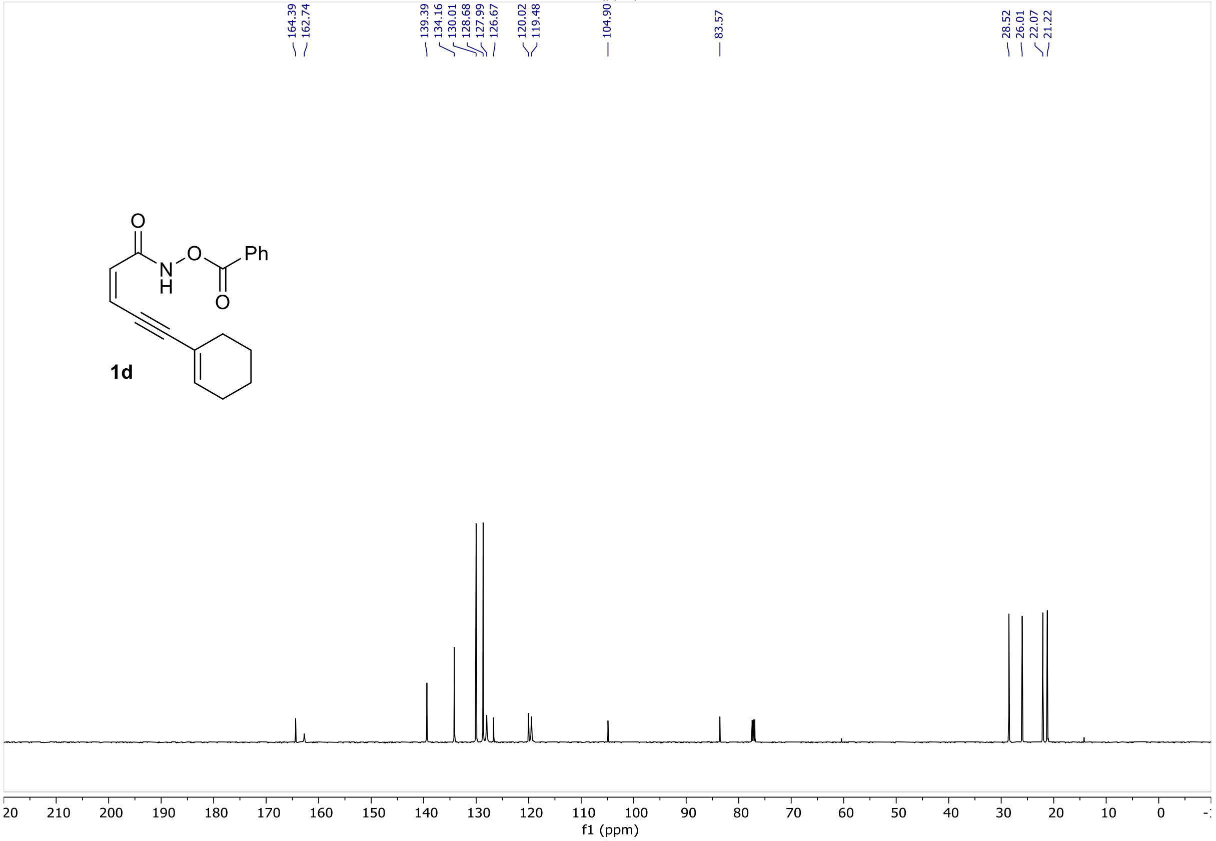

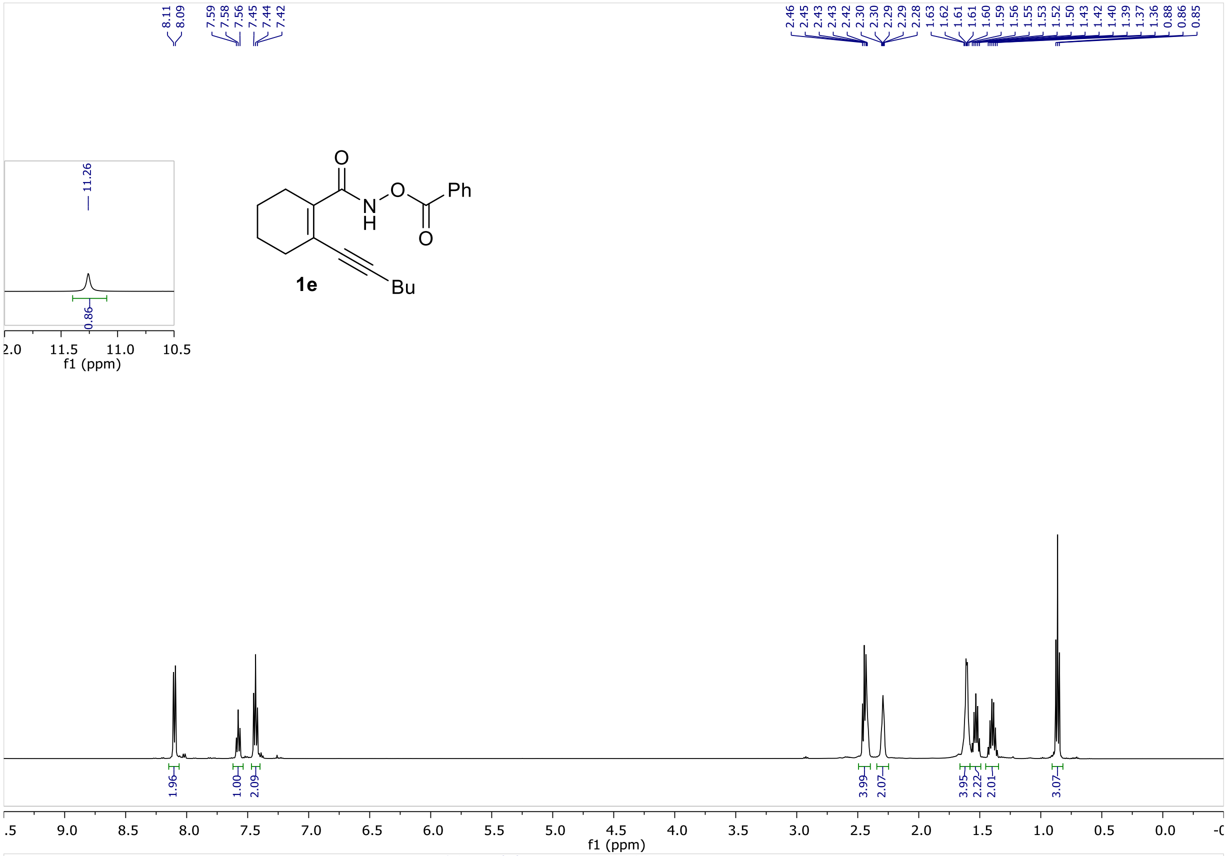
8.11
8.09
7.59
7.58
7.56
7.45
7.44
7.42
2.46
2.45
2.43
2.43
2.42
2.30
2.30
2.29
2.29
2.28
1.63
1.62
1.61
1.61
1.60
1.59
1.56
1.55
1.53
1.52
1.50
1.43
1.42
1.40
1.39
1.37
1.36
0.88
0.86
0.85
11.26
0.86
f1 (ppm)
Ph
O
N
H
O
O
1e
Bu
1.96
1.00
2.09
3.99
2.07
3.95
2.22
2.01
3.07
f1 (ppm)

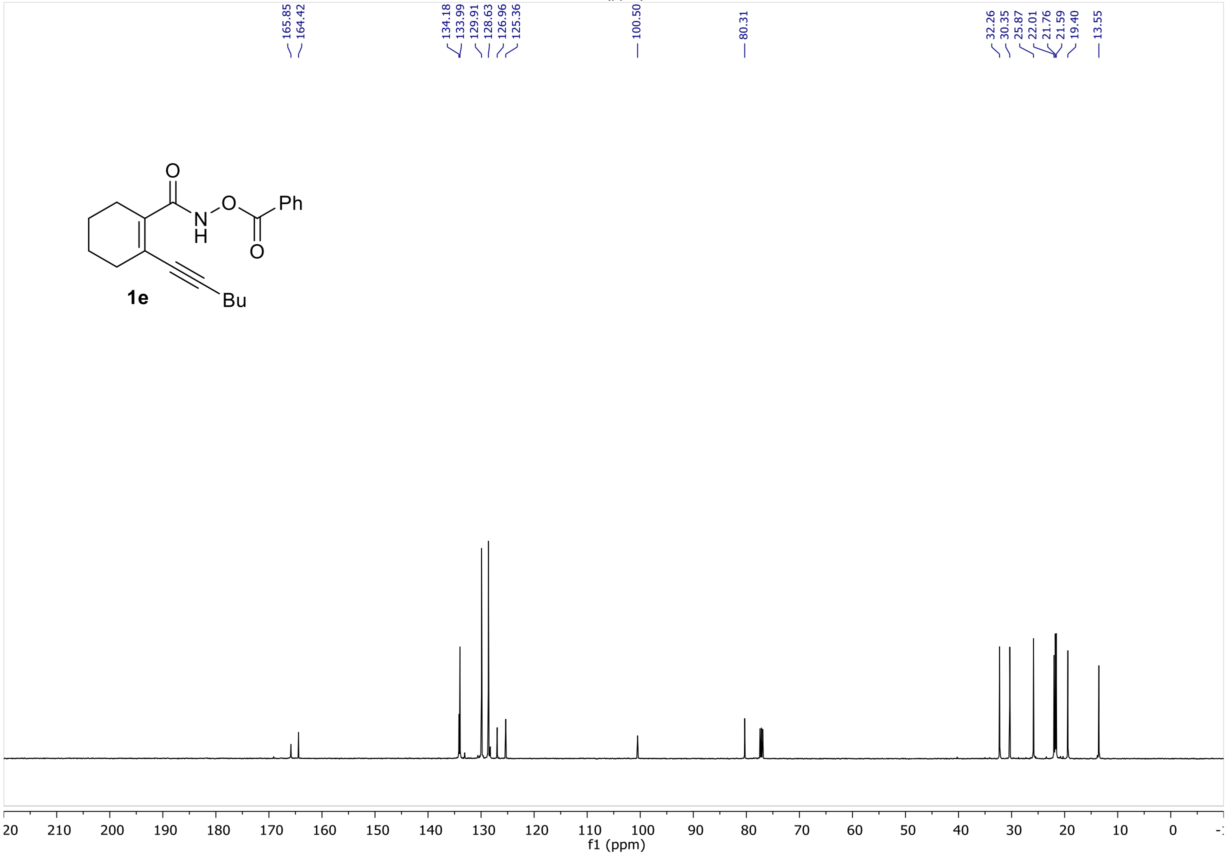
165.85
164.42
134.18
133.99
129.91
128.63
126.96
125.36
100.50
80.31
32.26
30.35
25.87
22.01
21.76
21.59
19.40
13.55
1e
Bu
Ph
f1 (ppm)

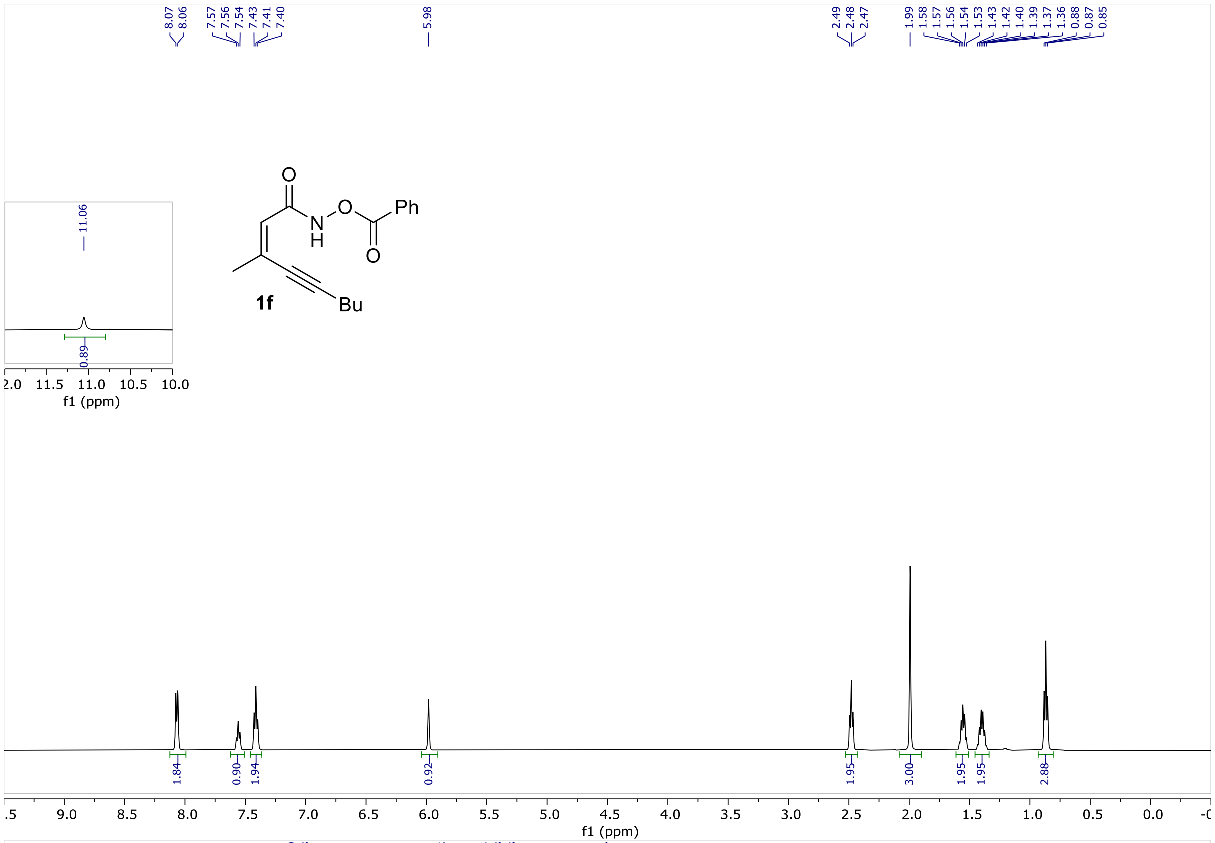
8.07
8.06
7.57
7.56
7.54
7.43
7.41
7.40
5.98
2.49
2.48
2.47
1.99
1.58
1.57
1.56
1.54
1.53
1.43
1.42
1.40
1.39
1.37
1.36
0.88
0.87
0.85
O
N
H
O
O
Ph
Bu
1f
11.06
0.89
f1 (ppm)
1.84
0.90
1.94
0.92
1.95
3.00
1.95
1.95
2.88
f1 (ppm)

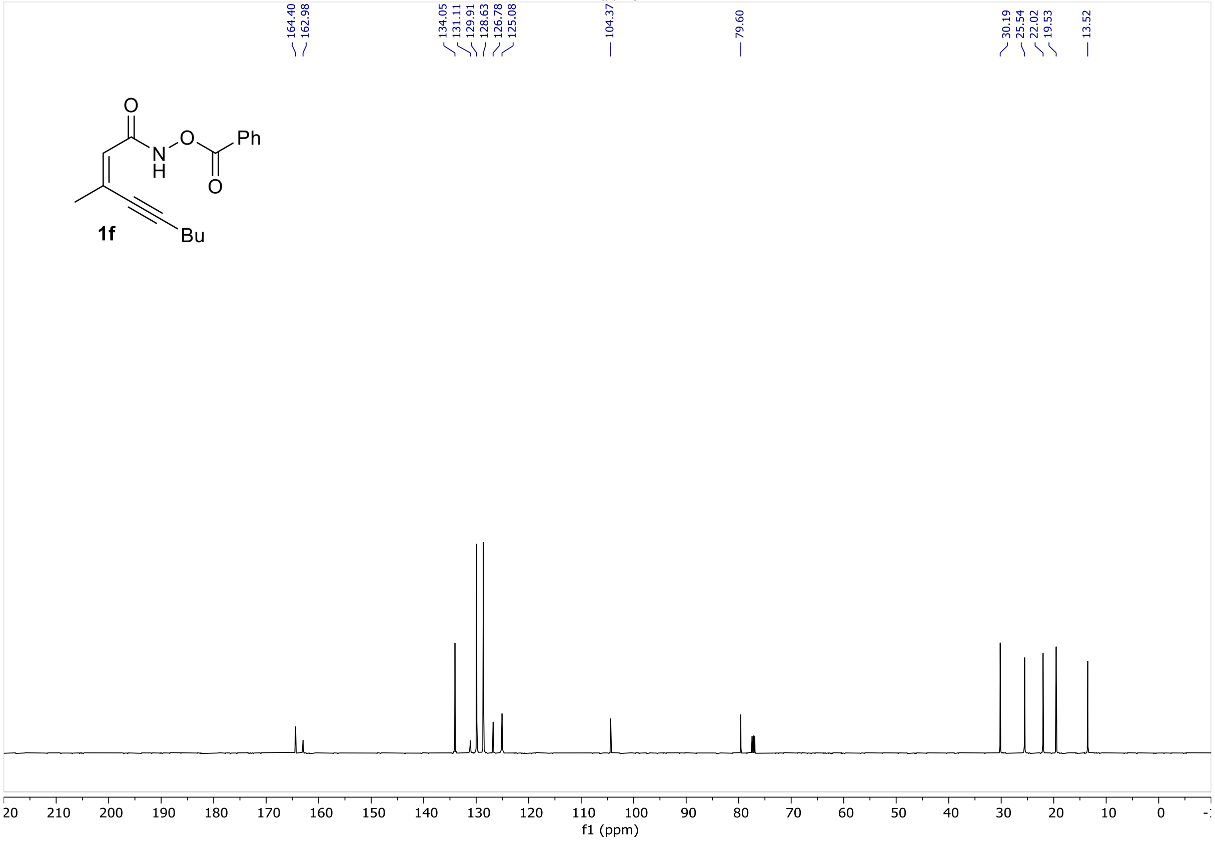
164.40
162.98
134.05
131.11
129.91
128.63
126.78
125.08
104.37
79.60
30.19
25.54
22.02
19.53
13.52
O
N
H
O
O
Ph
Bu
1f
f1 (ppm)

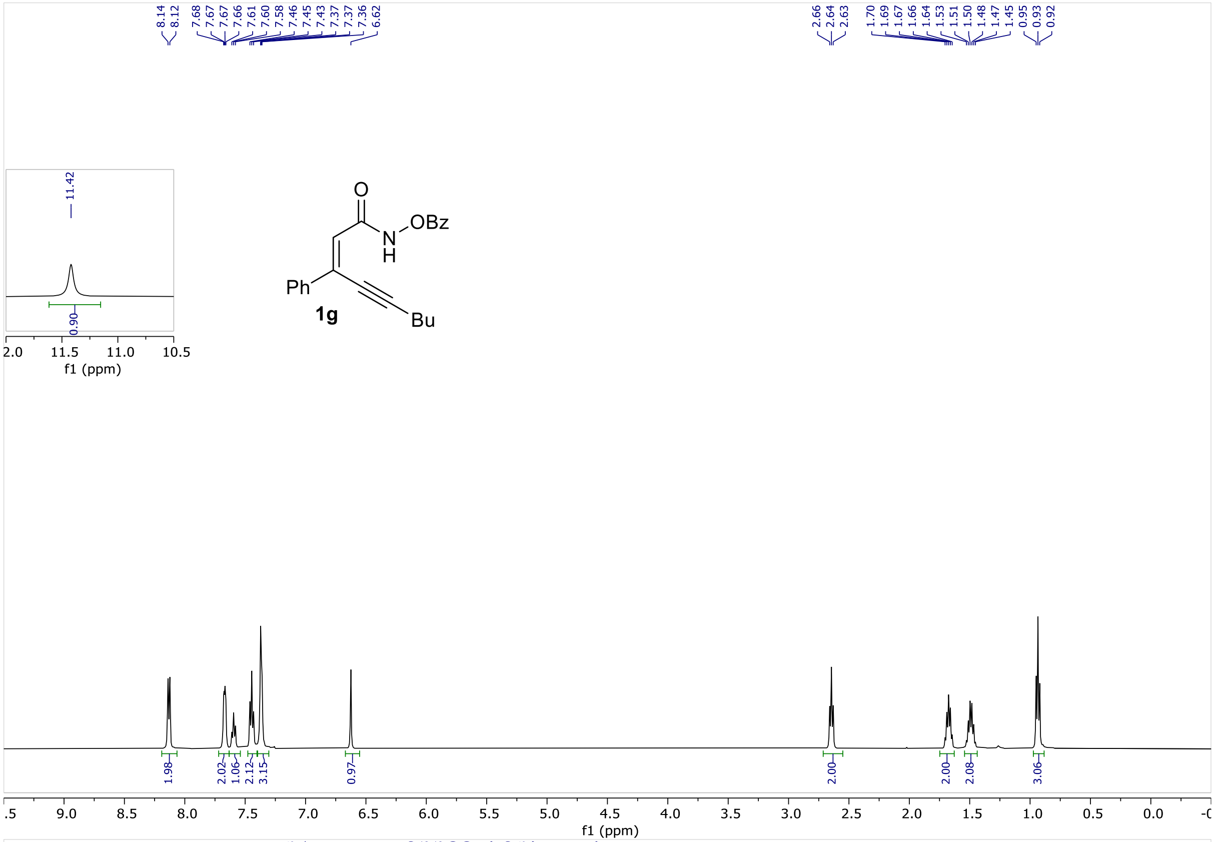
8.14
8.12
7.68
7.67
7.67
7.66
7.61
7.60
7.58
7.46
7.45
7.43
7.37
7.37
7.36
6.62
2.66
2.64
2.63
1.70
1.69
1.67
1.66
1.64
1.53
1.51
1.50
1.48
1.47
1.45
0.95
0.93
0.92
11.42
0.90
f1 (ppm)
O
N
OBz
H
Ph
1g
Bu
1.98
2.02
1.06
2.12
3.15
0.97
2.00
2.00
2.08
3.06
f1 (ppm)

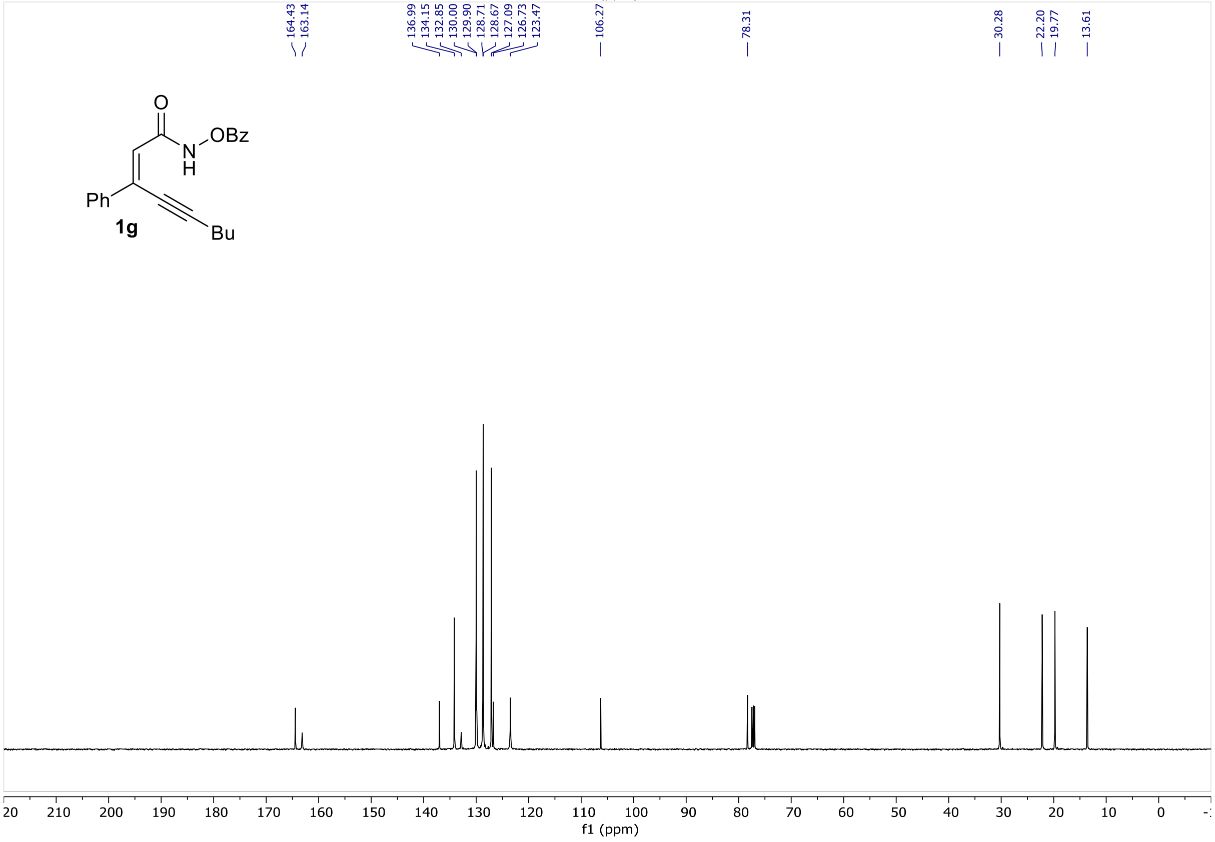
164.43
163.14
136.99
134.15
132.85
130.00
129.90
128.71
128.67
127.09
126.73
123.47
106.27
78.31
30.28
22.20
19.77
13.61
O
N
OBz
H
Ph
1g
Bu
f1 (ppm)

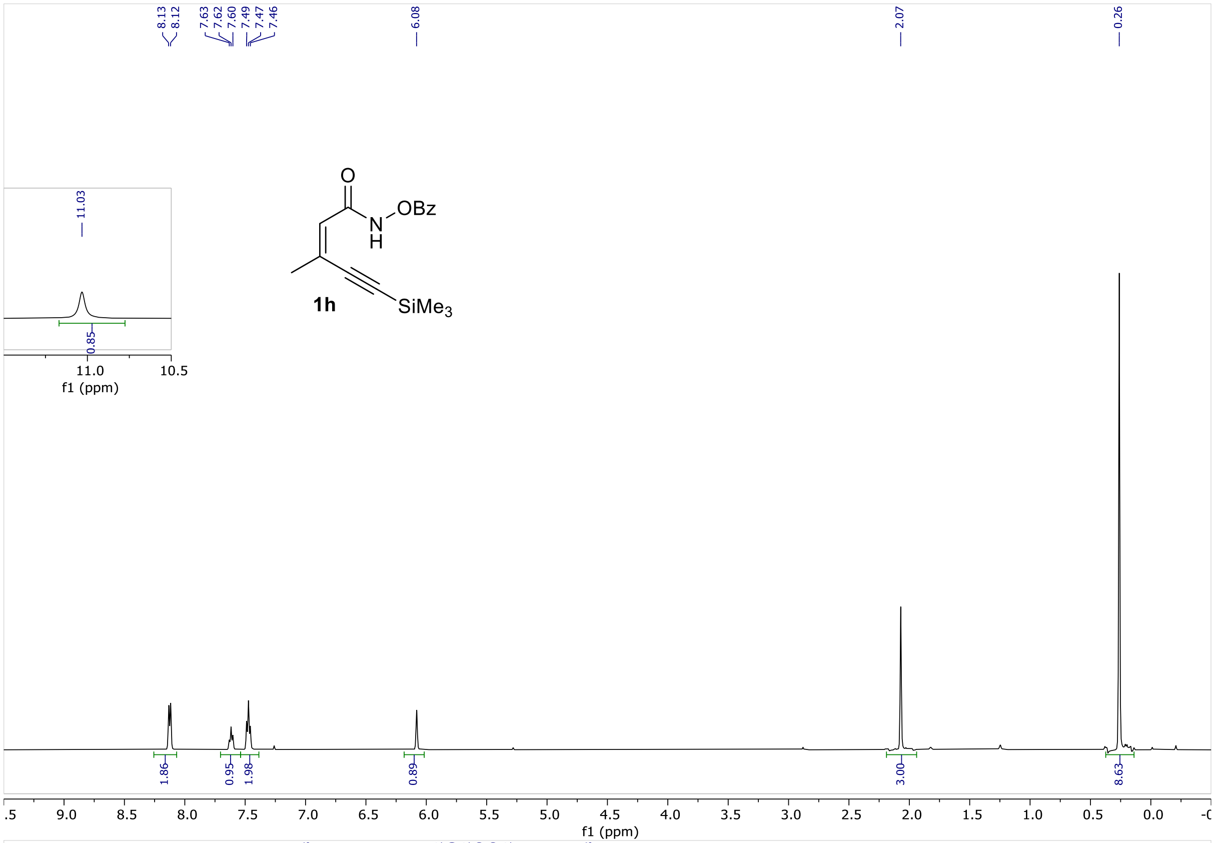
8.13
8.12
7.63
7.62
7.60
7.49
7.47
7.46
6.08
2.07
0.26
11.03
0.85
11.0
10.5
f1 (ppm)
OBz
1h
SiMe3
1.86
0.95
1.98
0.89
3.00
8.63
f1 (ppm)

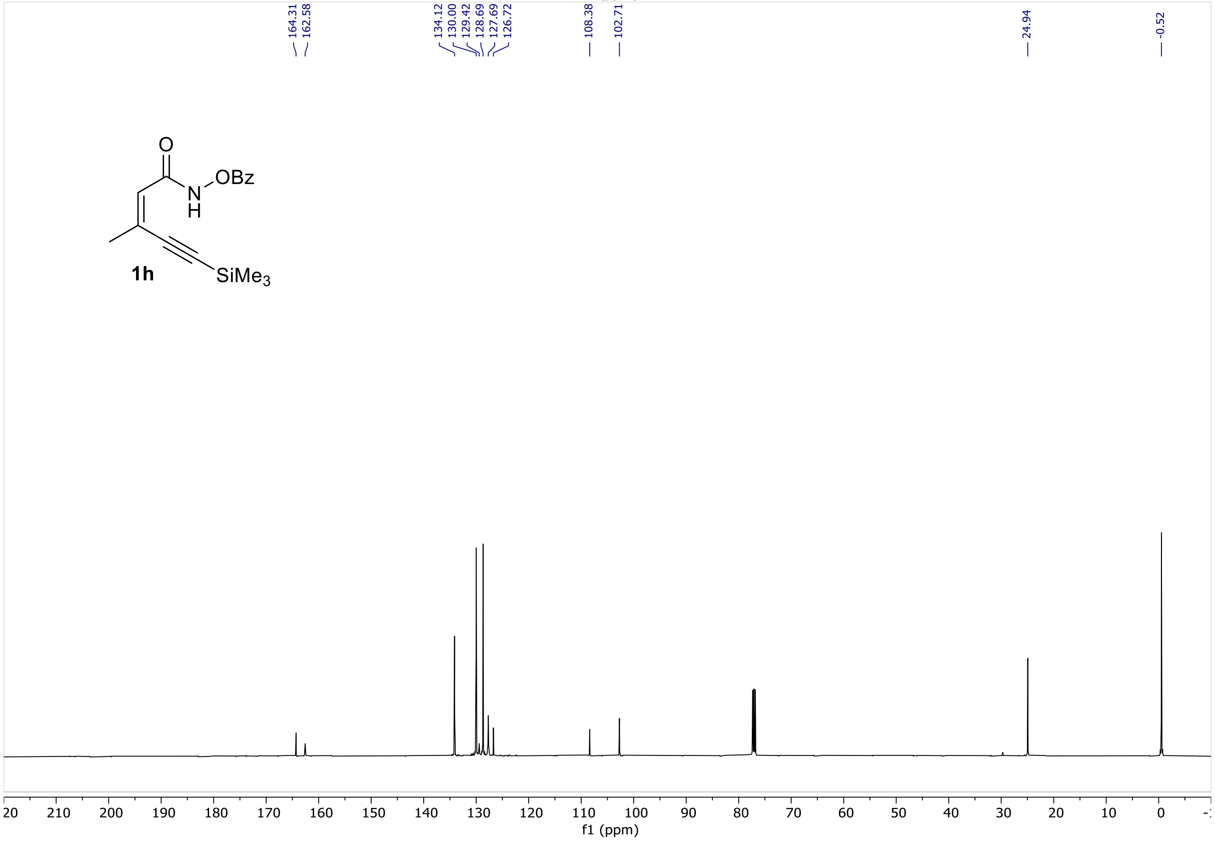
164.31
162.58
134.12
130.00
129.42
128.69
127.69
126.72
108.38
102.71
24.94
-0.52
OBz
1h
SiMe3
f1 (ppm)

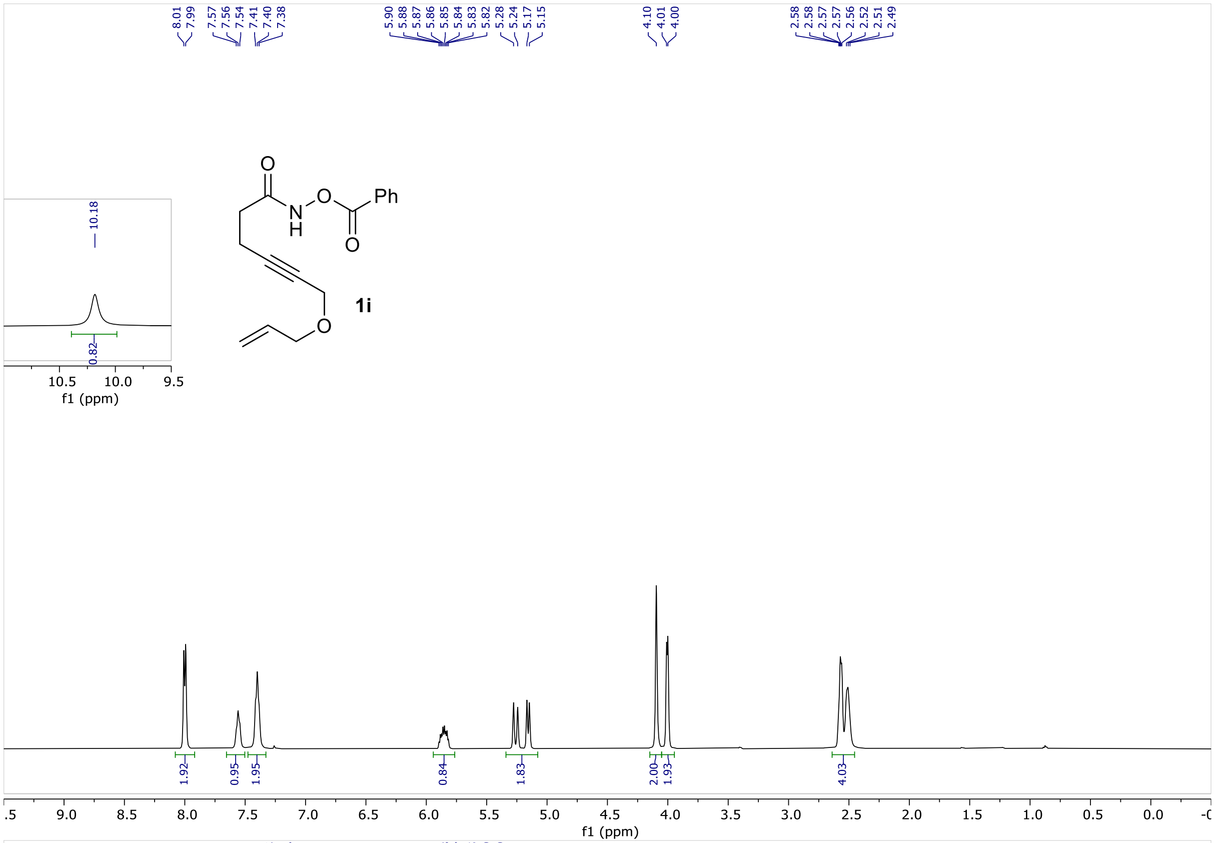
8.01
7.99
7.57
7.56
7.54
7.41
7.40
7.38
5.90
5.88
5.87
5.86
5.85
5.84
5.83
5.82
5.28
5.24
5.17
5.15
4.10
4.01
4.00
2.58
2.58
2.57
2.57
2.56
2.52
2.51
2.49
10.18
0.82
10.5
10.0
9.5
f1 (ppm)
O
N
H
O
Ph
O
O
1i
1.92
0.95
1.95
0.84
1.83
2.00
1.93
4.03
9.0
8.5
8.0
7.5
7.0
6.5
6.0
5.5
5.0
4.5
4.0
3.5
3.0
2.5
2.0
1.5
1.0
0.5
0.0
f1 (ppm)

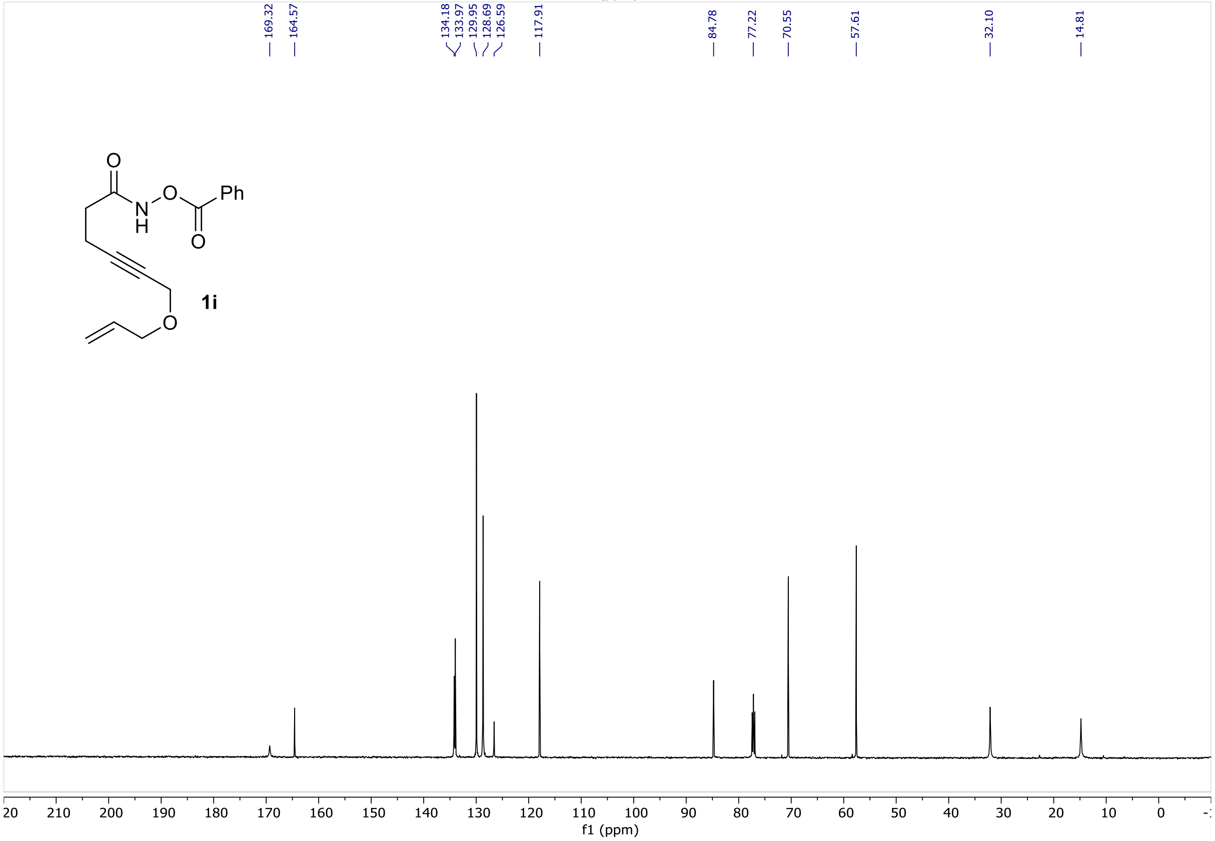
169.32
164.57
134.18
133.97
129.95
128.69
126.59
117.91
84.78
77.22
70.55
57.61
32.10
14.81
O
N
H
O
Ph
O
O
1i
210
200
190
180
170
160
150
140
130
120
110
100
90
80
70
60
50
40
30
20
10
0
f1 (ppm)

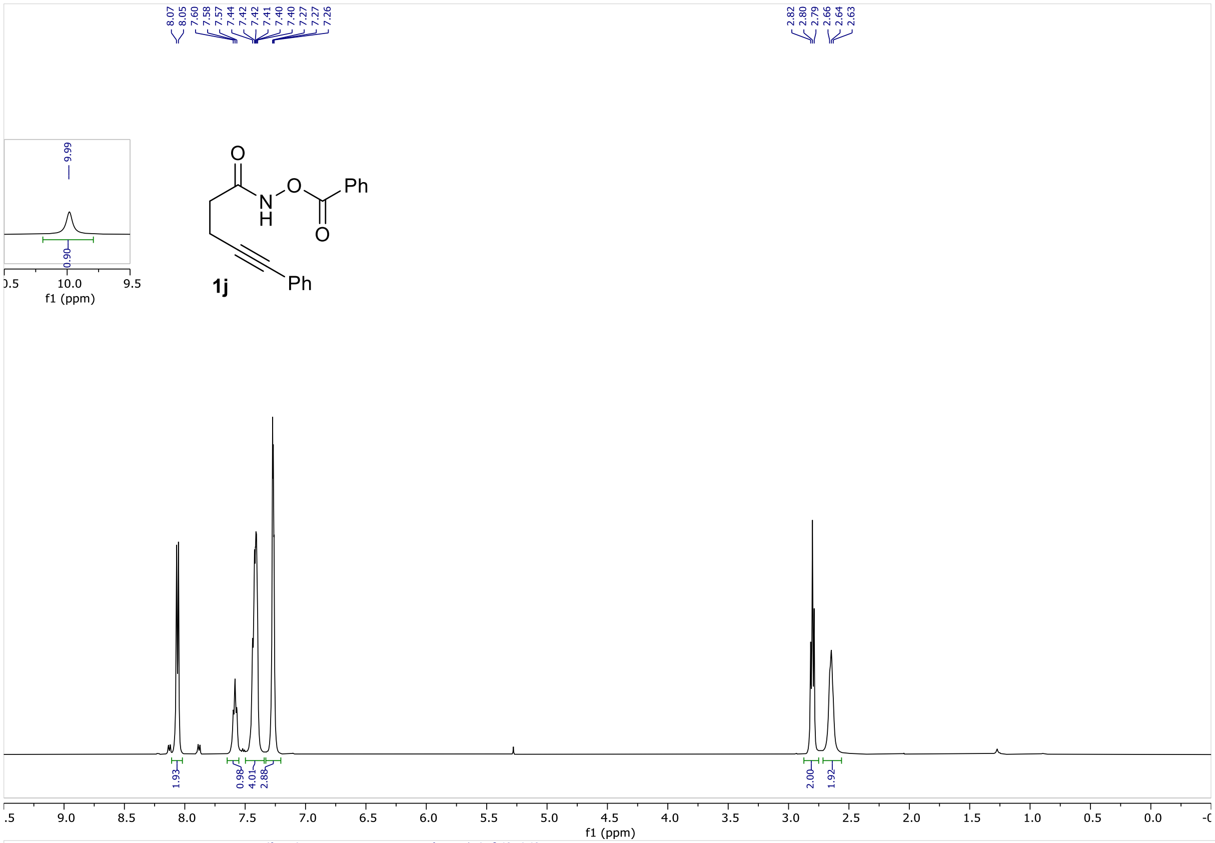
8.07
8.05
7.60
7.58
7.57
7.44
7.42
7.42
7.41
7.40
7.40
7.27
7.27
7.26
2.82
2.80
2.79
2.66
2.64
2.63
9.99
0.90
1j
Ph
1.93
0.98
4.01
2.88
2.00
1.92
f1 (ppm)

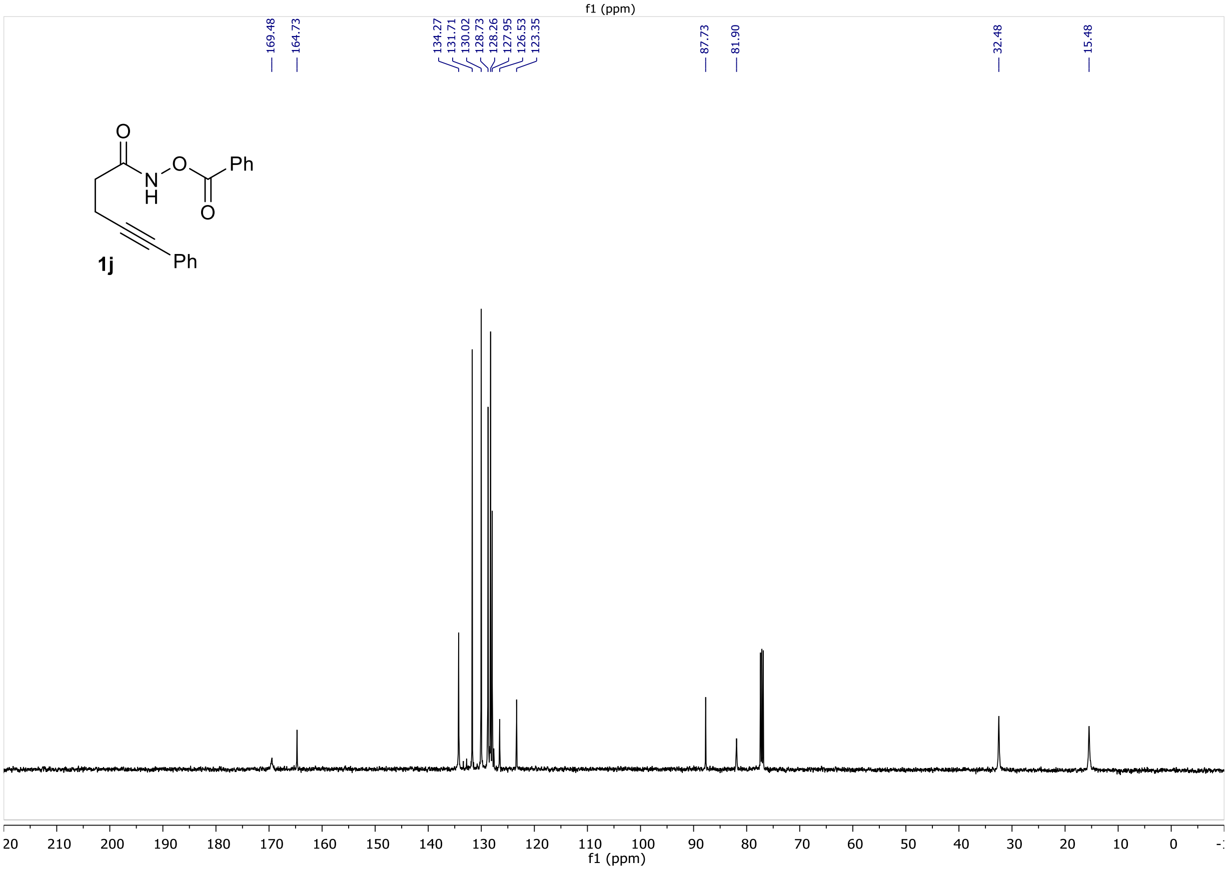
169.48
164.73
134.27
131.71
130.02
128.73
128.26
127.95
126.53
123.35
87.73
81.90
32.48
15.48
1j
Ph
f1 (ppm)

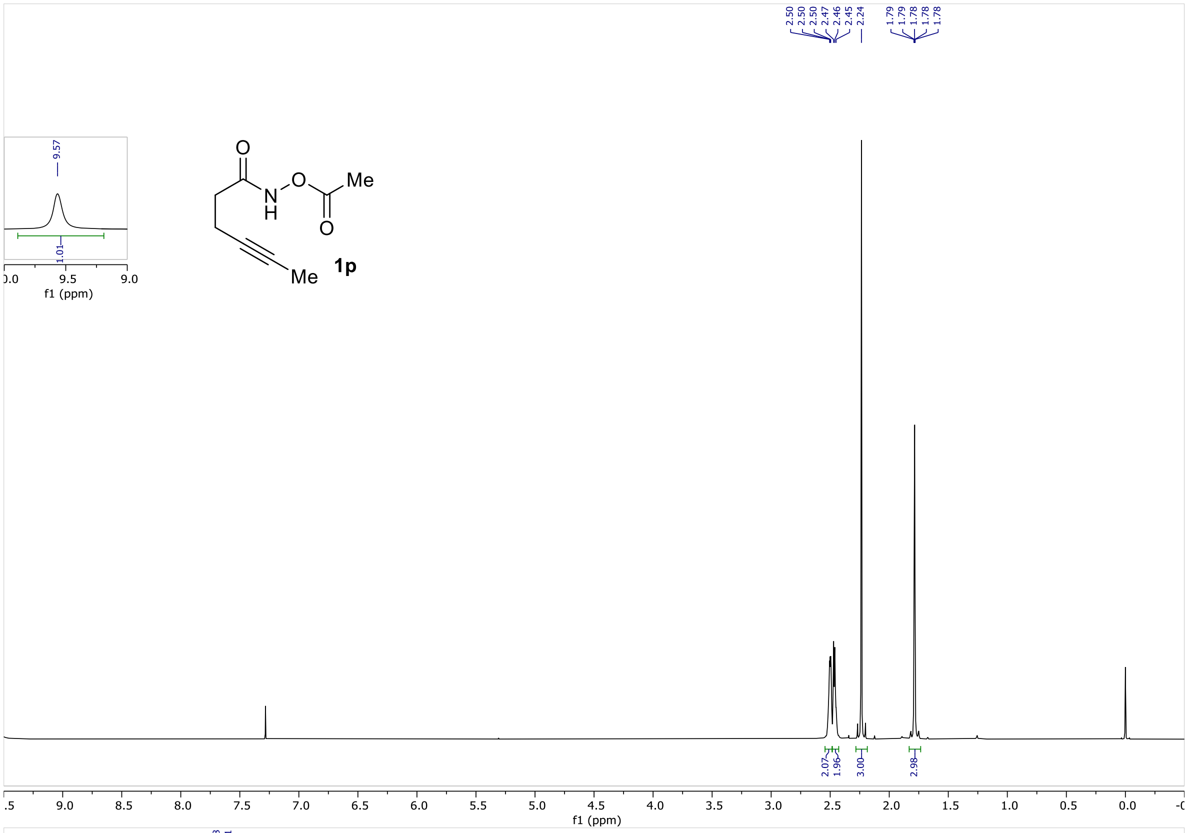

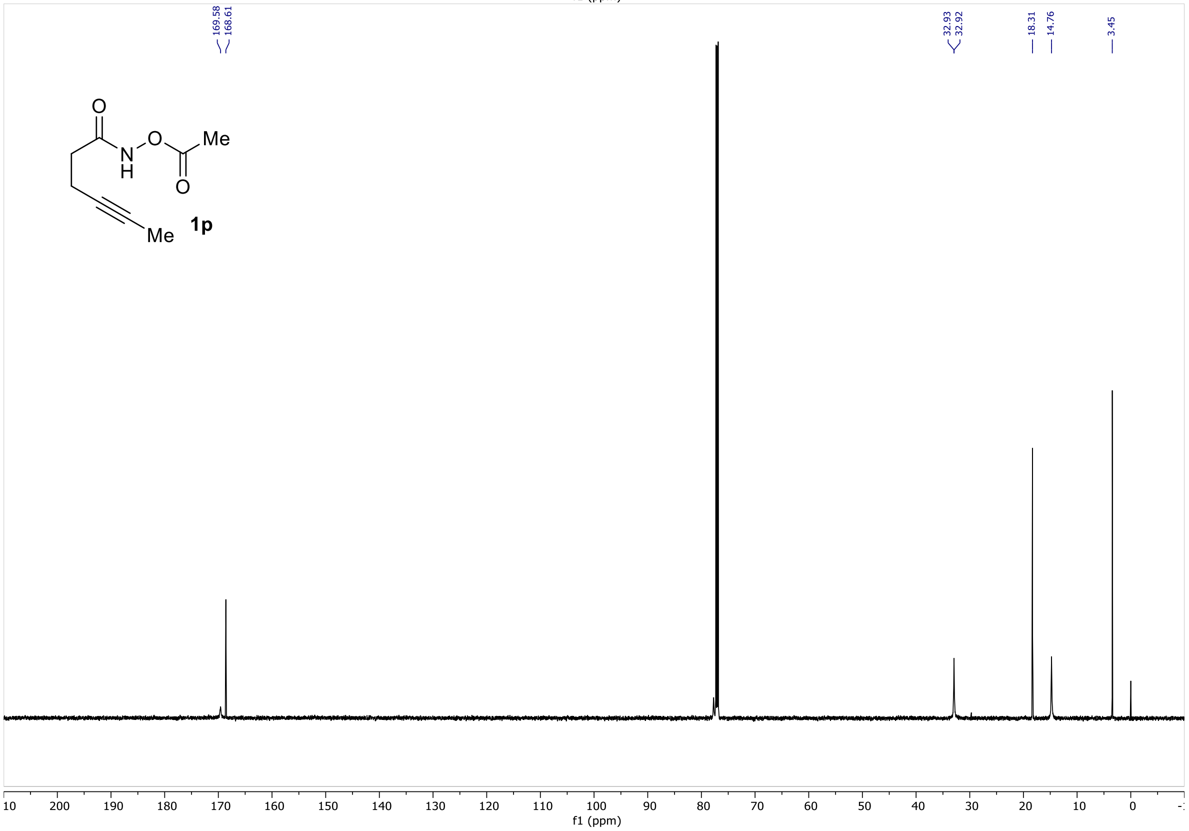

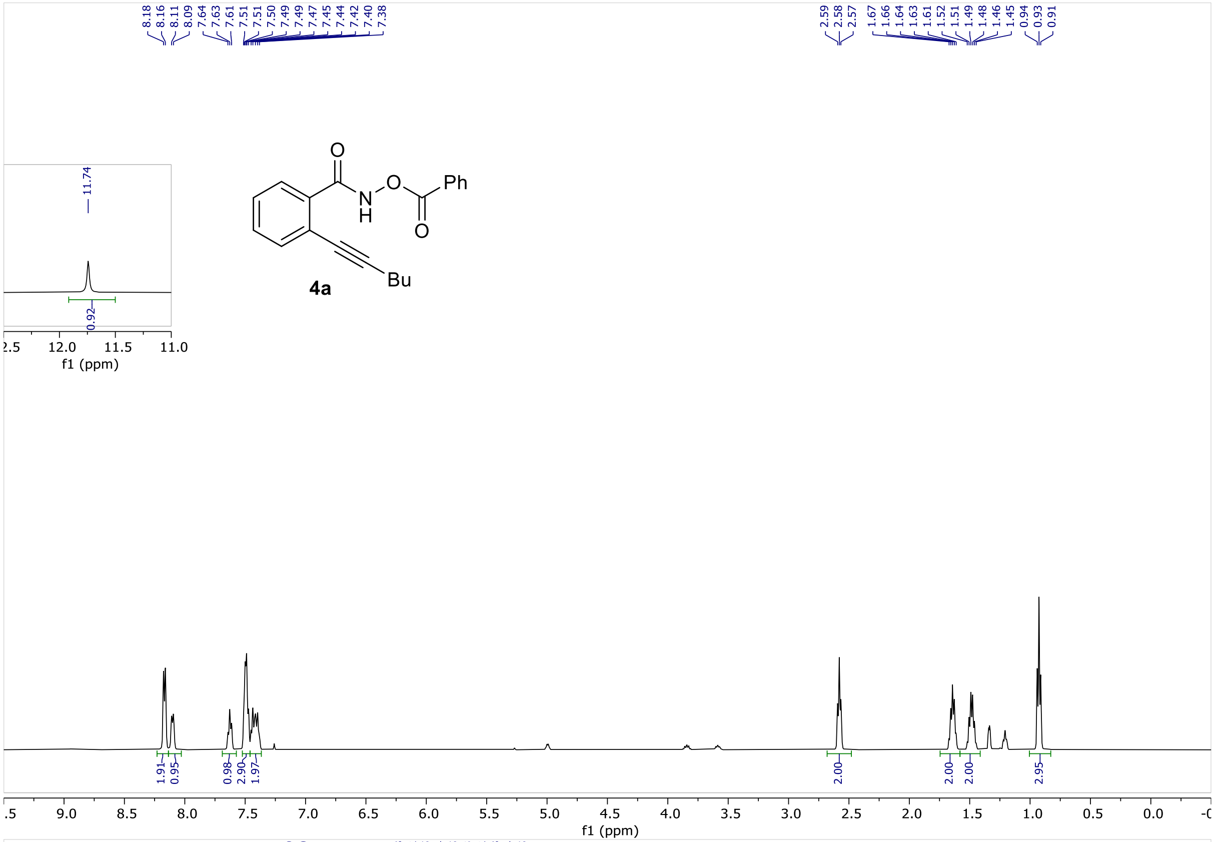
8.18
8.16
8.11
8.09
7.64
7.63
7.61
7.51
7.51
7.50
7.49
7.49
7.47
7.45
7.44
7.42
7.40
7.38
2.59
2.58
2.57
1.67
1.66
1.64
1.63
1.61
1.52
1.51
1.49
1.48
1.46
1.45
0.94
0.93
0.91
11.74
0.92
f1 (ppm)
4a
Bu
Ph
1.91
0.95
0.98
2.90
1.97
2.00
2.00
2.00
2.95
f1 (ppm)

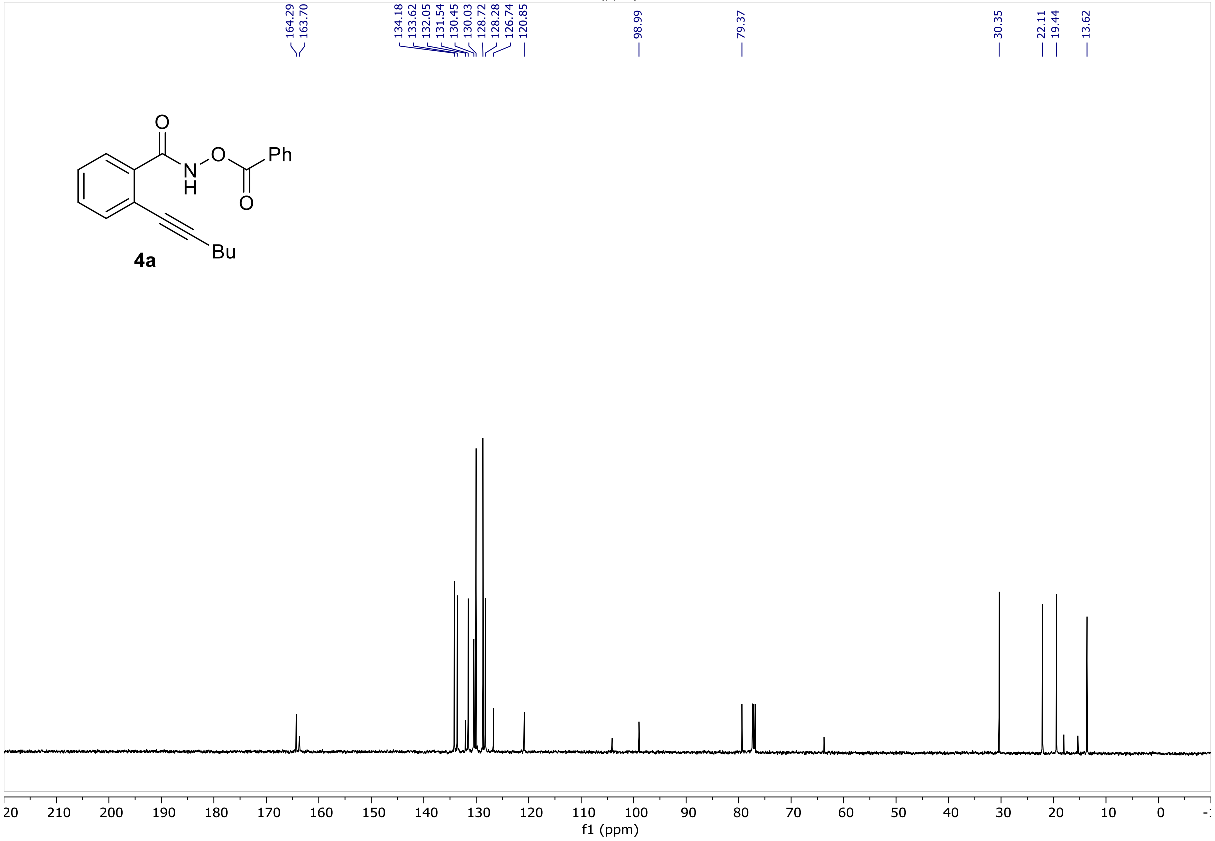
164.29
163.70
134.18
133.62
132.05
131.54
130.45
130.03
128.72
128.28
126.74
120.85
98.99
79.37
30.35
22.11
19.44
13.62
Ph
Bu
4a
f1 (ppm)

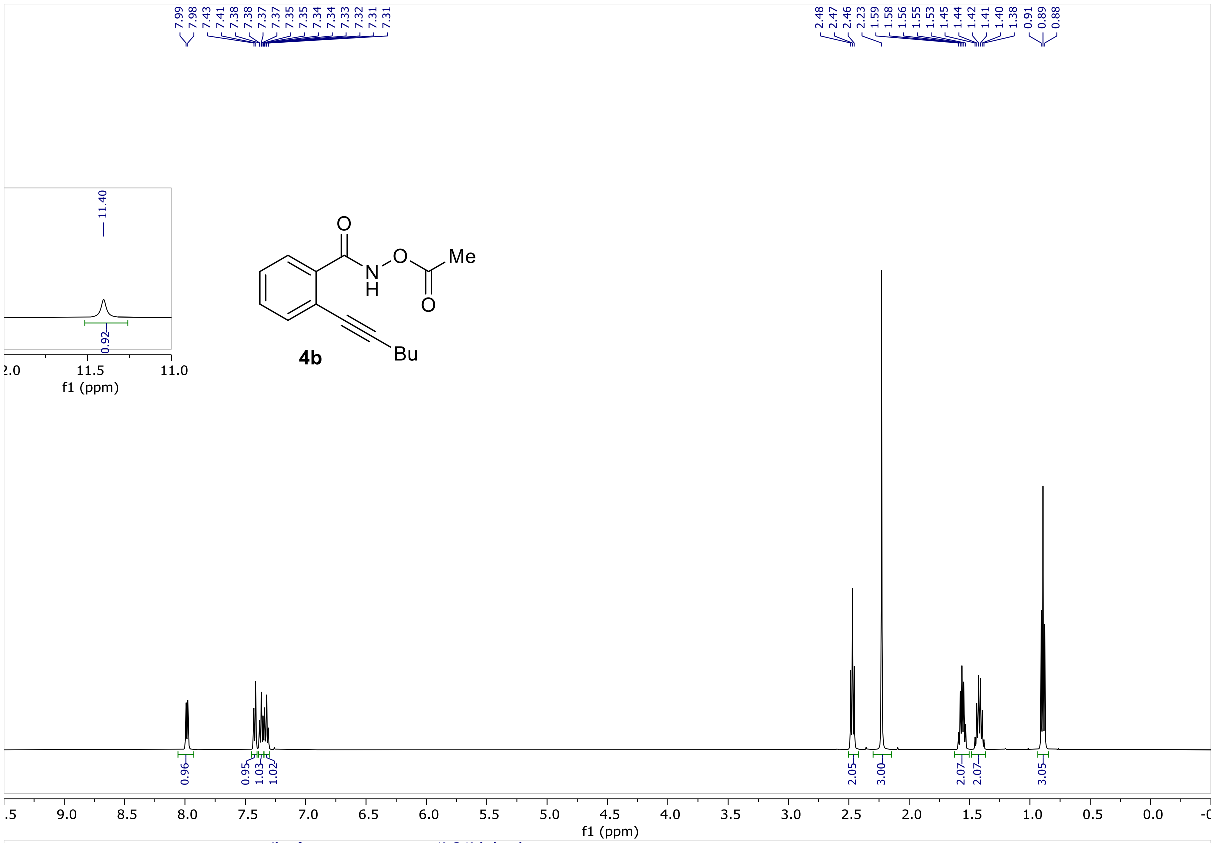

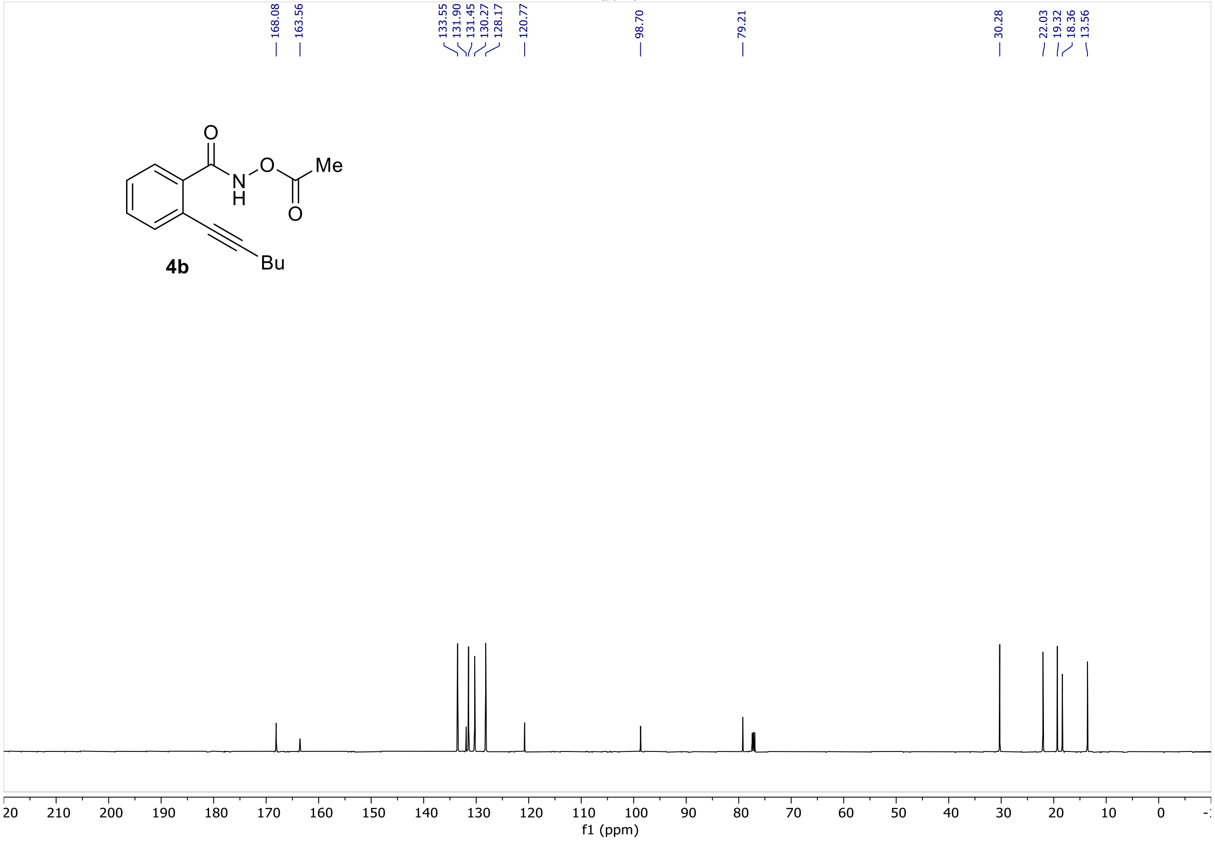

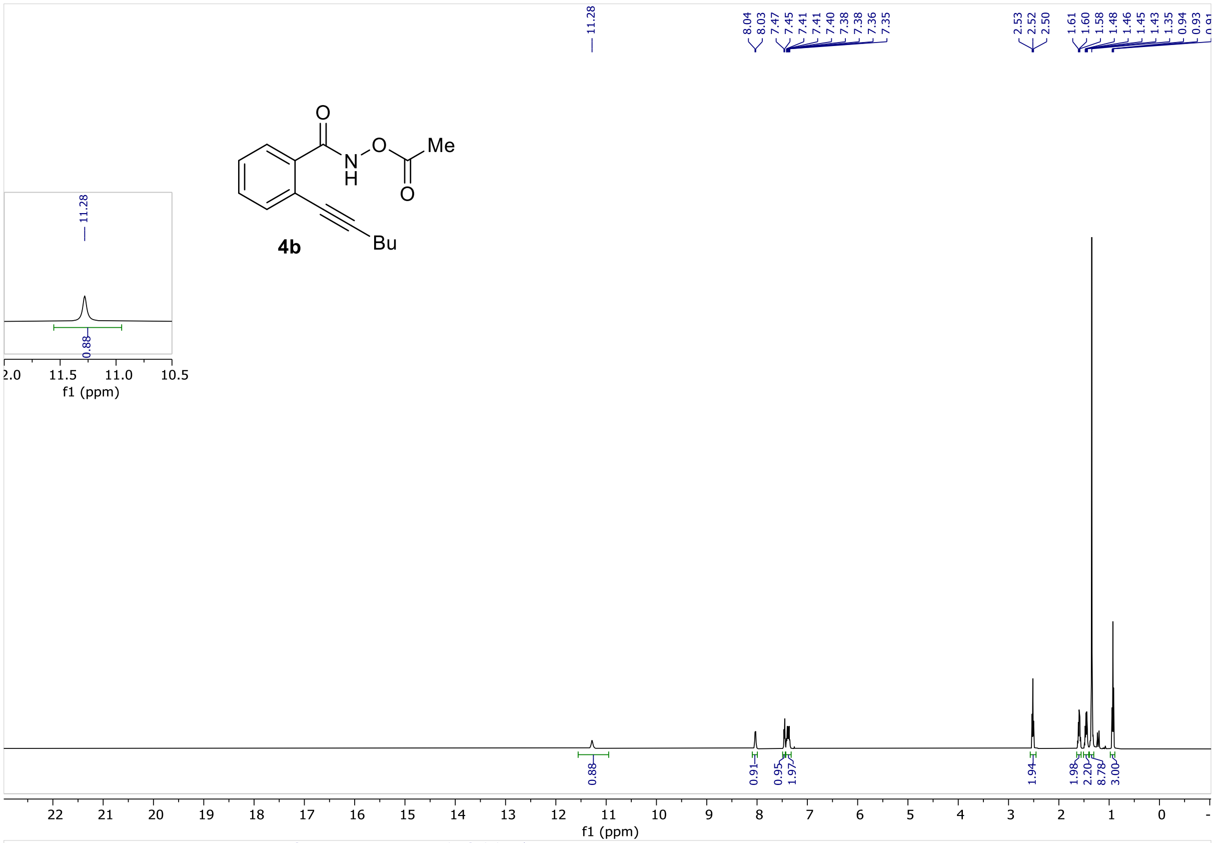
11.28
8.04
8.03
7.47
7.45
7.41
7.41
7.40
7.38
7.38
7.36
7.35
2.53
2.52
2.50
1.61
1.60
1.58
1.48
1.46
1.45
1.43
1.35
0.94
0.93
O
N
H
O
Me
O
4b
Bu
0.88
11.5
11.0
10.5
f1 (ppm)
0.91
0.95
1.97
1.94
1.98
2.20
8.78
3.00
22
21
20
19
18
17
16
15
14
13
12
11
10
9
8
7
6
5
4
3
2
1
0
f1 (ppm)

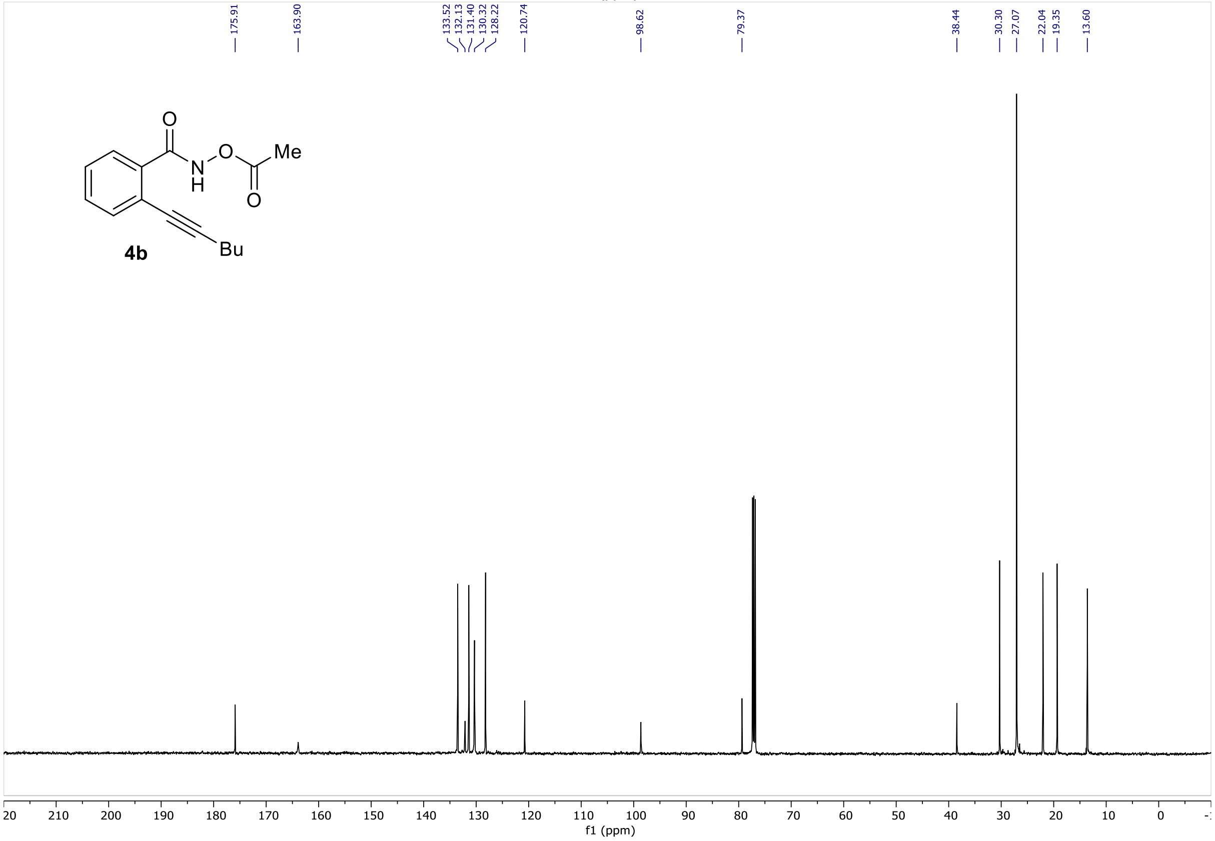
175.91
163.90
133.52
132.13
131.40
130.32
128.22
120.74
98.62
79.37
38.44
30.30
27.07
22.04
19.35
13.60
O
N
H
O
Me
O
4b
Bu
210
200
190
180
170
160
150
140
130
120
110
100
90
80
70
60
50
40
30
20
10
0
f1 (ppm)

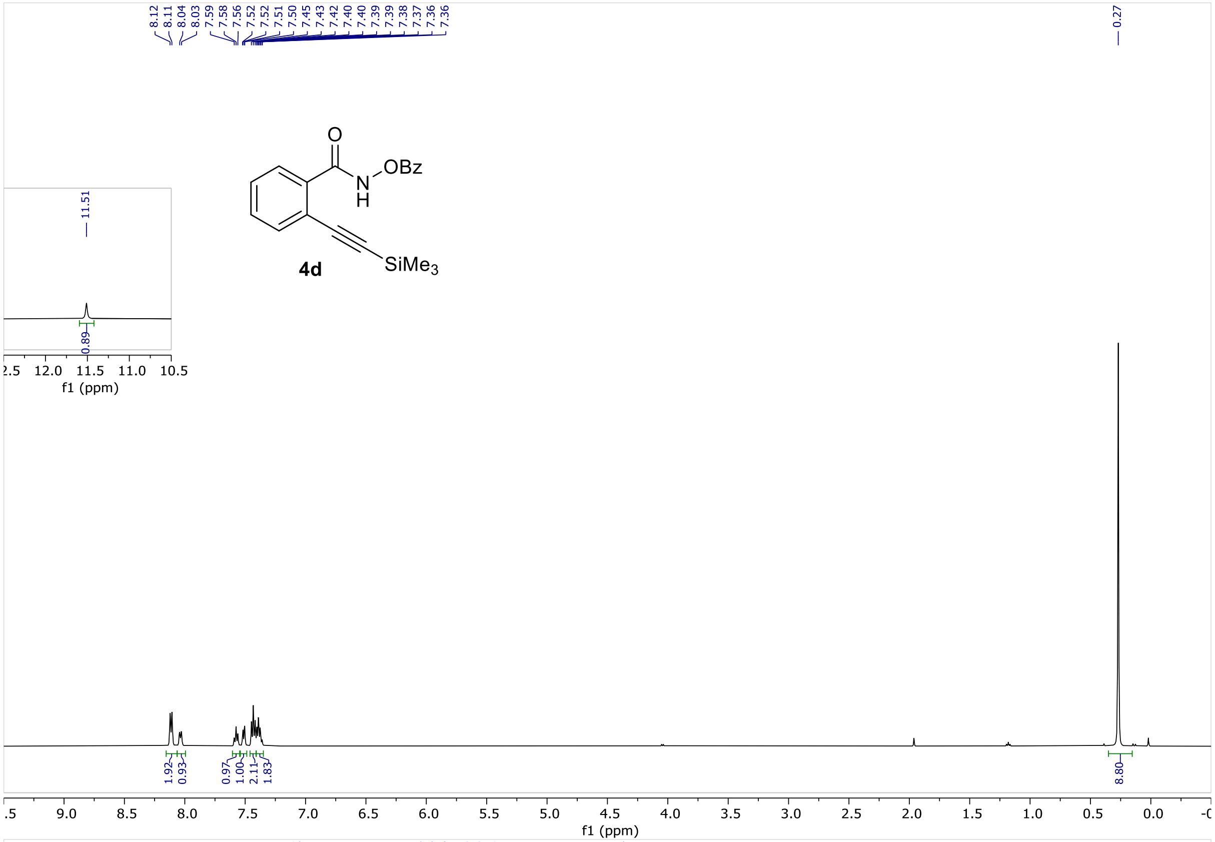
8.12
8.11
8.04
8.03
7.59
7.58
7.56
7.52
7.52
7.51
7.50
7.45
7.43
7.42
7.40
7.40
7.39
7.39
7.38
7.37
7.36
7.36
0.27
11.51
0.89
OBz
N
H
O
SiMe3
4d
1.92
0.93
0.97
1.00
2.11
1.83
8.80
f1 (ppm)

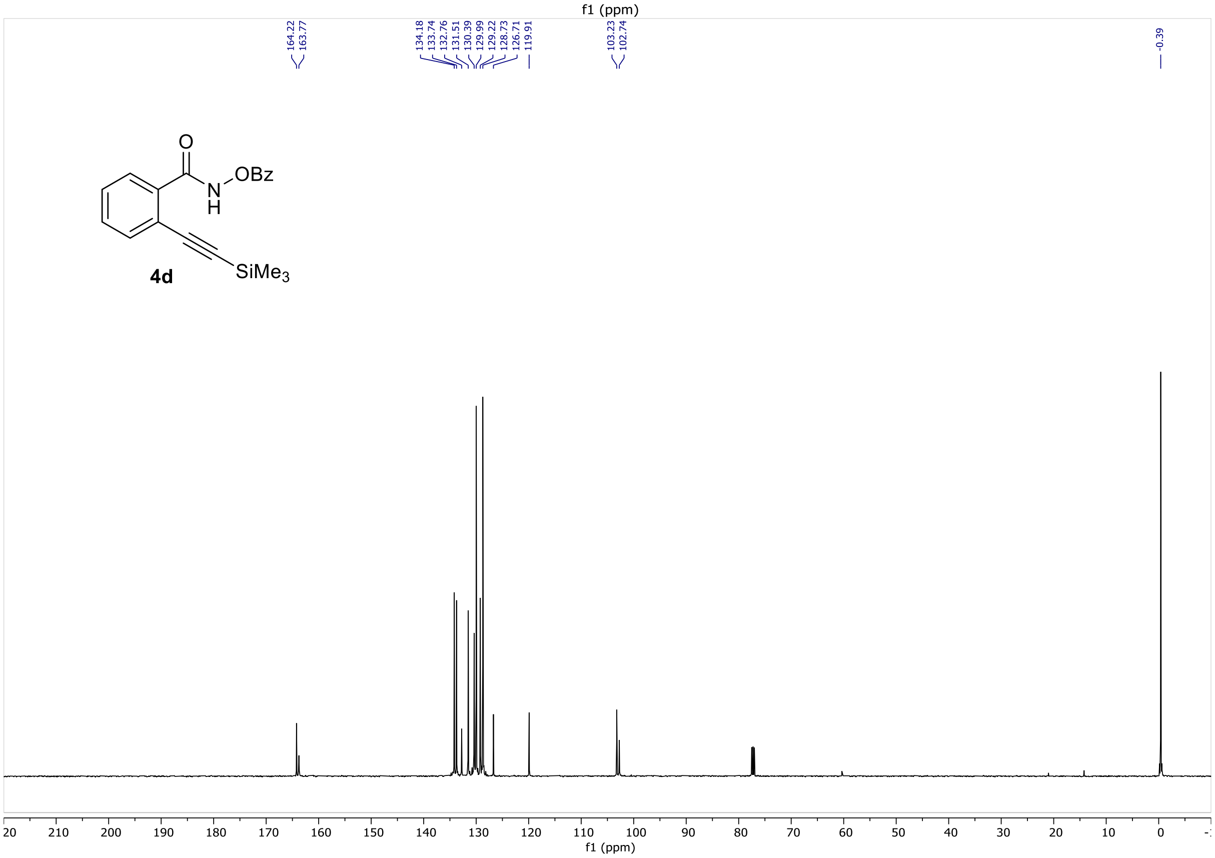
164.22
163.77
134.18
133.74
132.76
131.51
130.39
129.99
129.22
128.73
126.71
119.91
103.23
102.74
-0.39
O
N
H
OBz
SiMe3
4d
f1 (ppm)

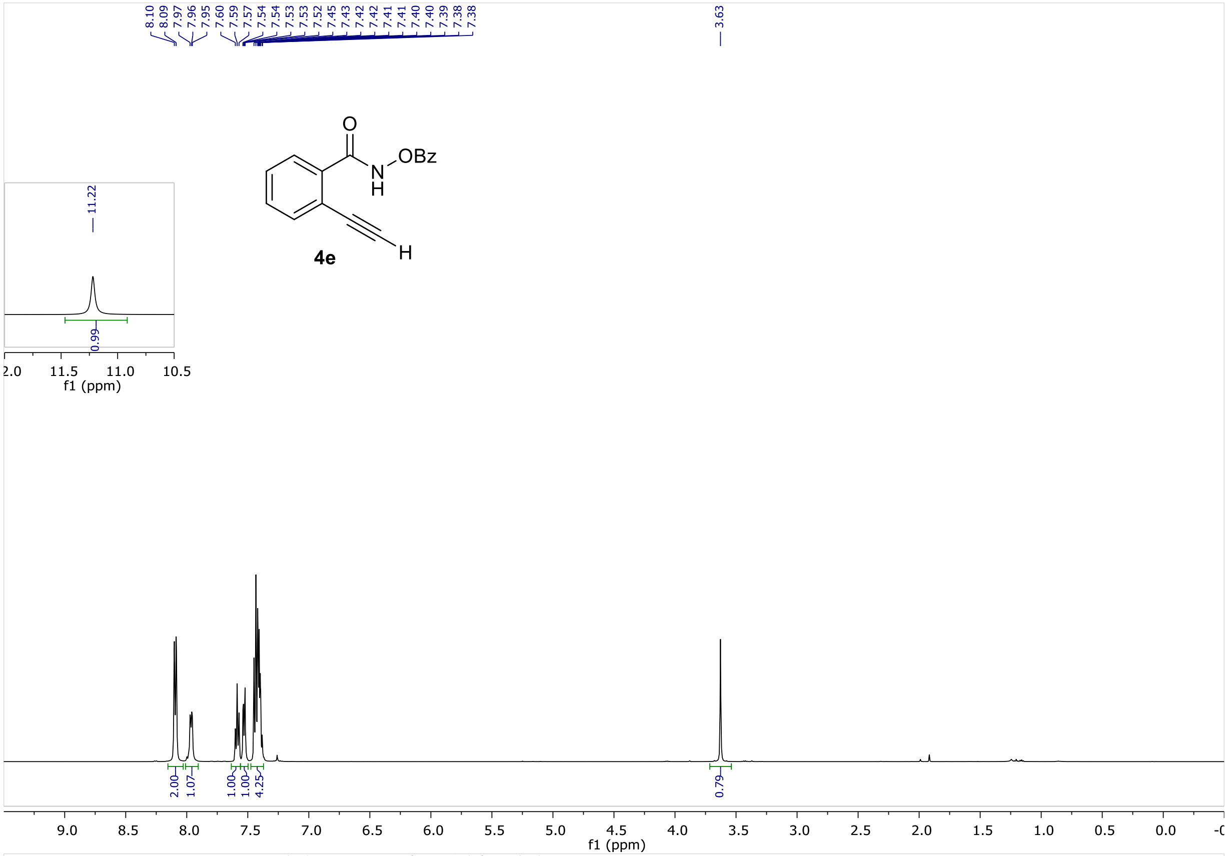

8.10
8.09
7.97
7.96
7.95
7.60
7.59
7.57
7.54
7.54
7.53
7.53
7.52
7.45
7.43
7.42
7.42
7.41
7.41
7.40
7.39
7.38
7.38
3.63
11.22
0.99
f1 (ppm)
OBz
N
H
O
H
4e
2.00
1.07
1.00
1.00
4.25
0.79
f1 (ppm)

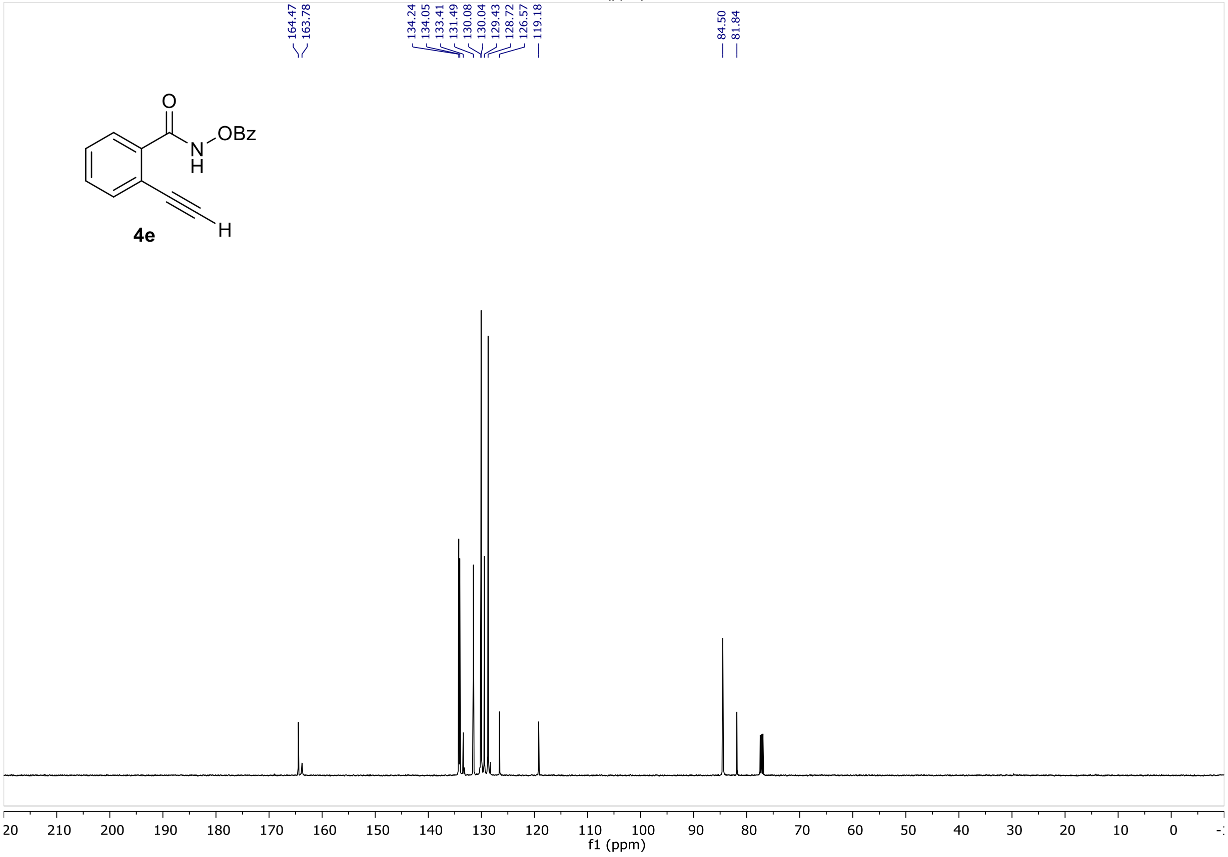

164.47
163.78
134.24
134.05
133.41
131.49
130.08
130.04
129.43
128.72
126.57
119.18
84.50
81.84
O
N
H
OBz
H
4e
f1 (ppm)

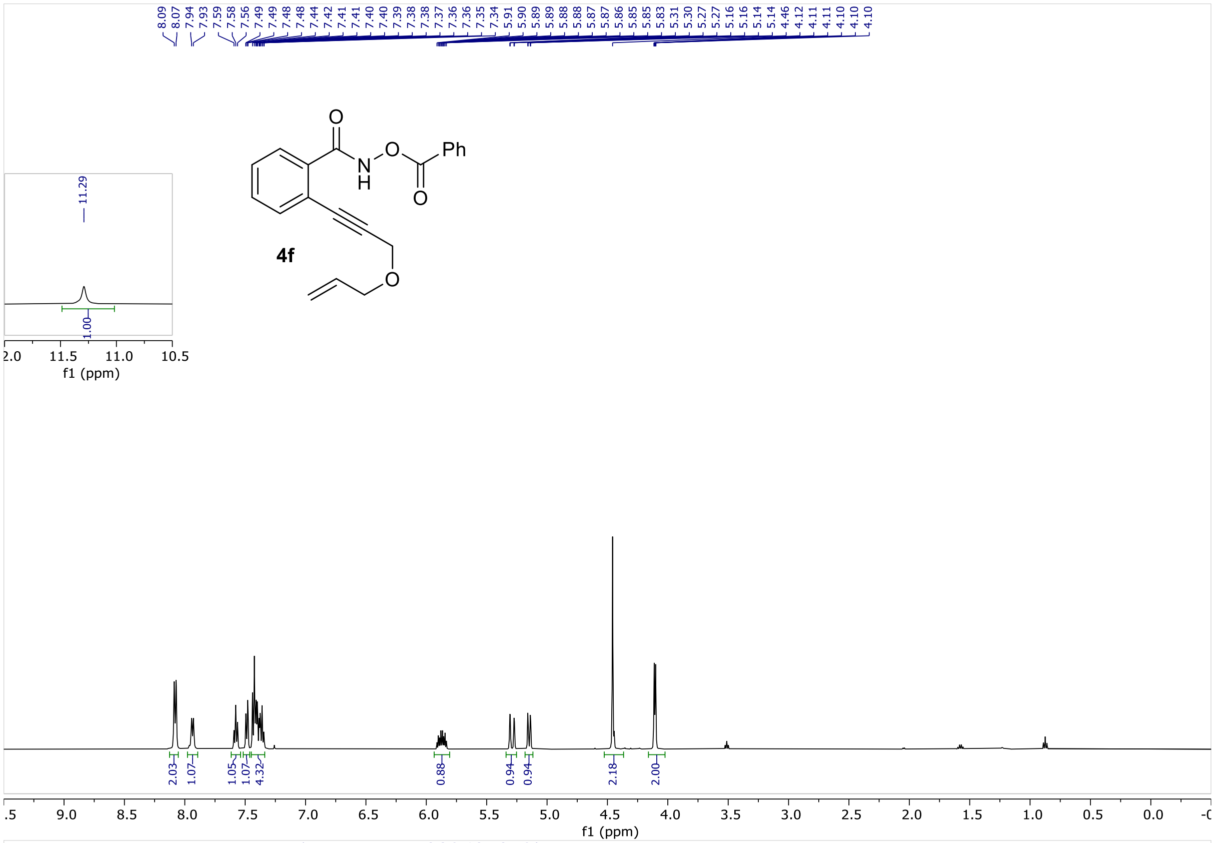

8.09
8.07
7.94
7.93
7.59
7.58
7.56
7.49
7.49
7.48
7.48
7.44
7.42
7.41
7.41
7.40
7.40
7.39
7.38
7.38
7.37
7.36
7.36
7.35
7.34
5.91
5.90
5.89
5.89
5.88
5.88
5.87
5.87
5.86
5.85
5.85
5.83
5.31
5.30
5.27
5.27
5.16
5.16
5.14
5.14
4.46
4.12
4.11
4.11
4.10
4.10
4.10
11.29
1.00
11.5
11.0
10.5
f1 (ppm)
Ph
O
N
H
4f
2.03
1.07
1.05
1.07
4.32
0.88
0.94
0.94
2.18
2.00
9.0
8.5
8.0
7.5
7.0
6.5
6.0
5.5
5.0
4.5
4.0
3.5
3.0
2.5
2.0
1.5
1.0
0.5
0.0
f1 (ppm)

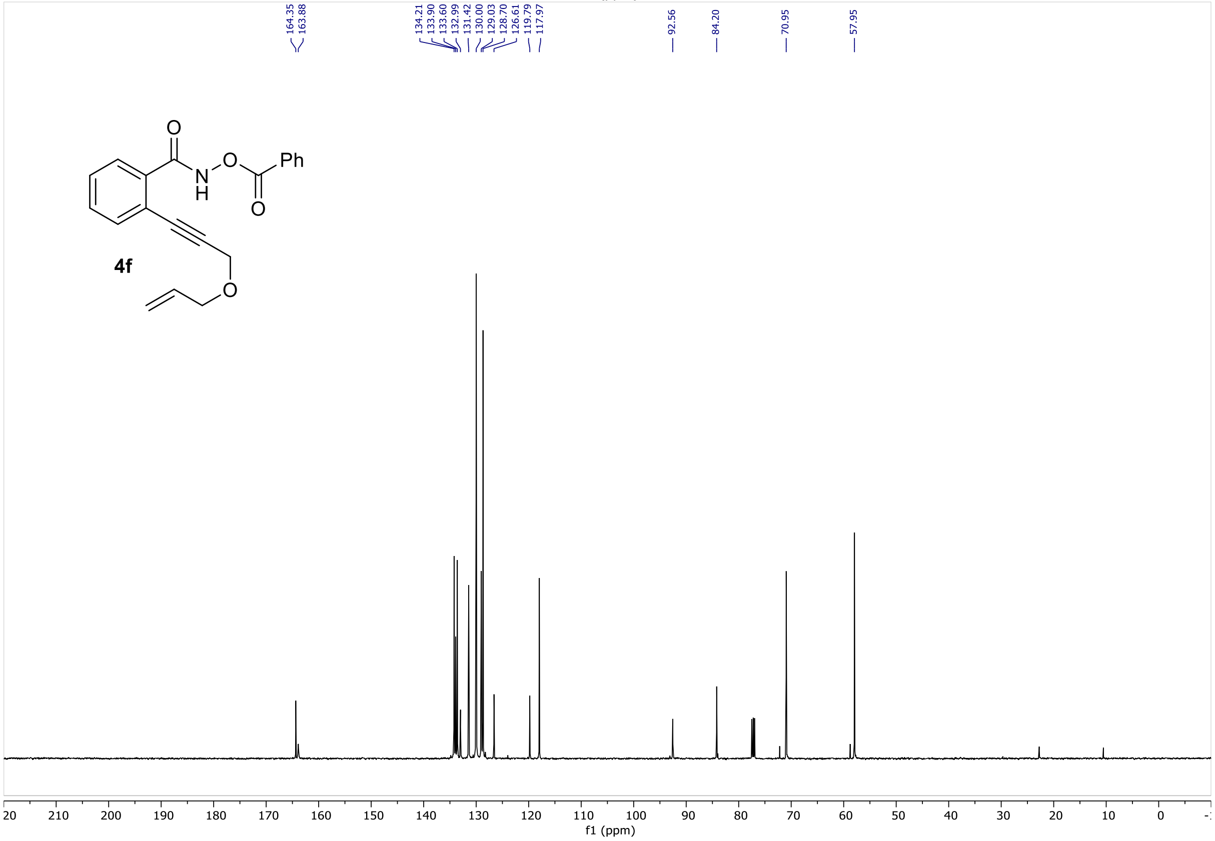

164.35
163.88
134.21
133.90
133.60
132.99
131.42
130.00
129.03
128.70
126.61
119.79
117.97
92.56
84.20
70.95
57.95
Ph
O
N
H
4f
210
200
190
180
170
160
150
140
130
120
110
100
90
80
70
60
50
40
30
20
10
0
f1 (ppm)

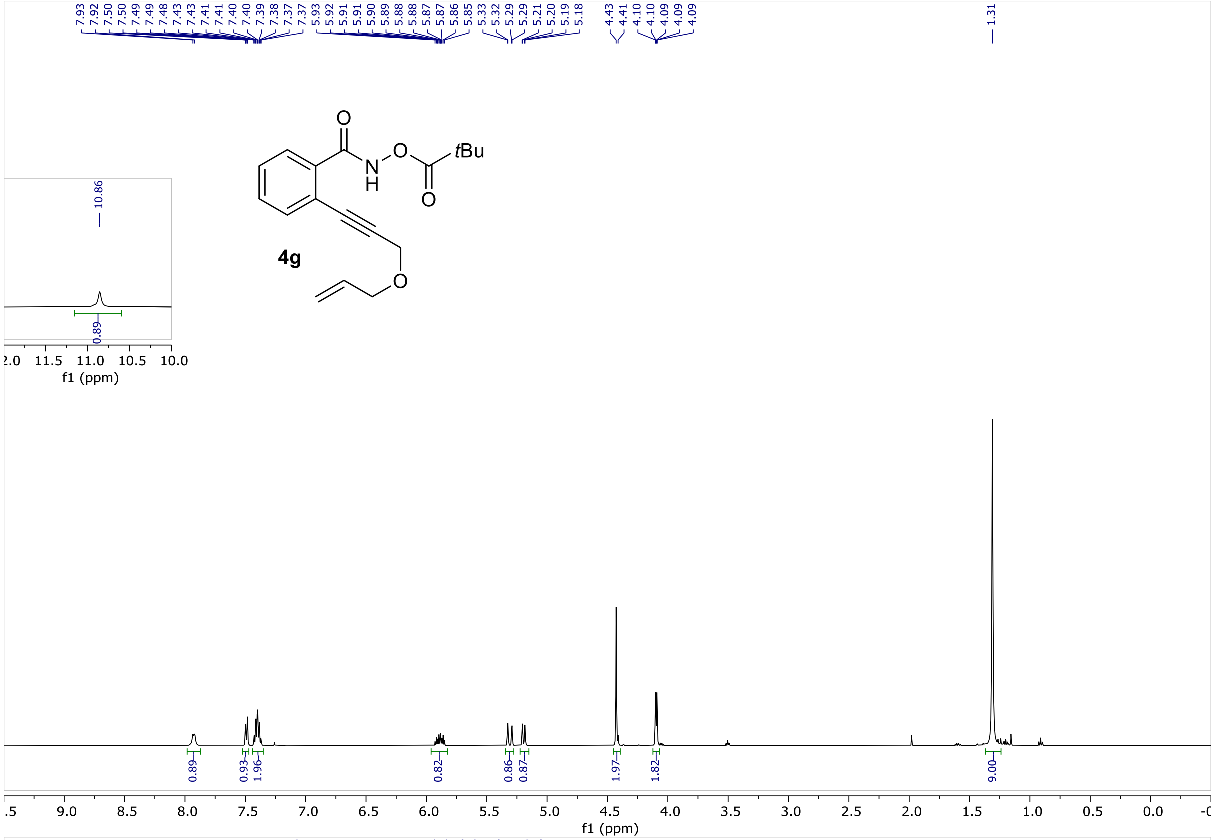
7.93
7.92
7.50
7.50
7.49
7.49
7.48
7.43
7.43
7.41
7.41
7.40
7.40
7.39
7.38
7.37
7.37
5.93
5.92
5.91
5.91
5.90
5.89
5.88
5.88
5.87
5.87
5.86
5.85
5.33
5.32
5.29
5.29
5.21
5.20
5.19
5.18
4.43
4.41
4.10
4.10
4.09
4.09
4.09
1.31
10.86
0.89
f1 (ppm)
O
N
H
O
O
tBu
O
4g
0.89
0.93
1.96
0.82
0.86
0.87
1.97
1.82
9.00
f1 (ppm)

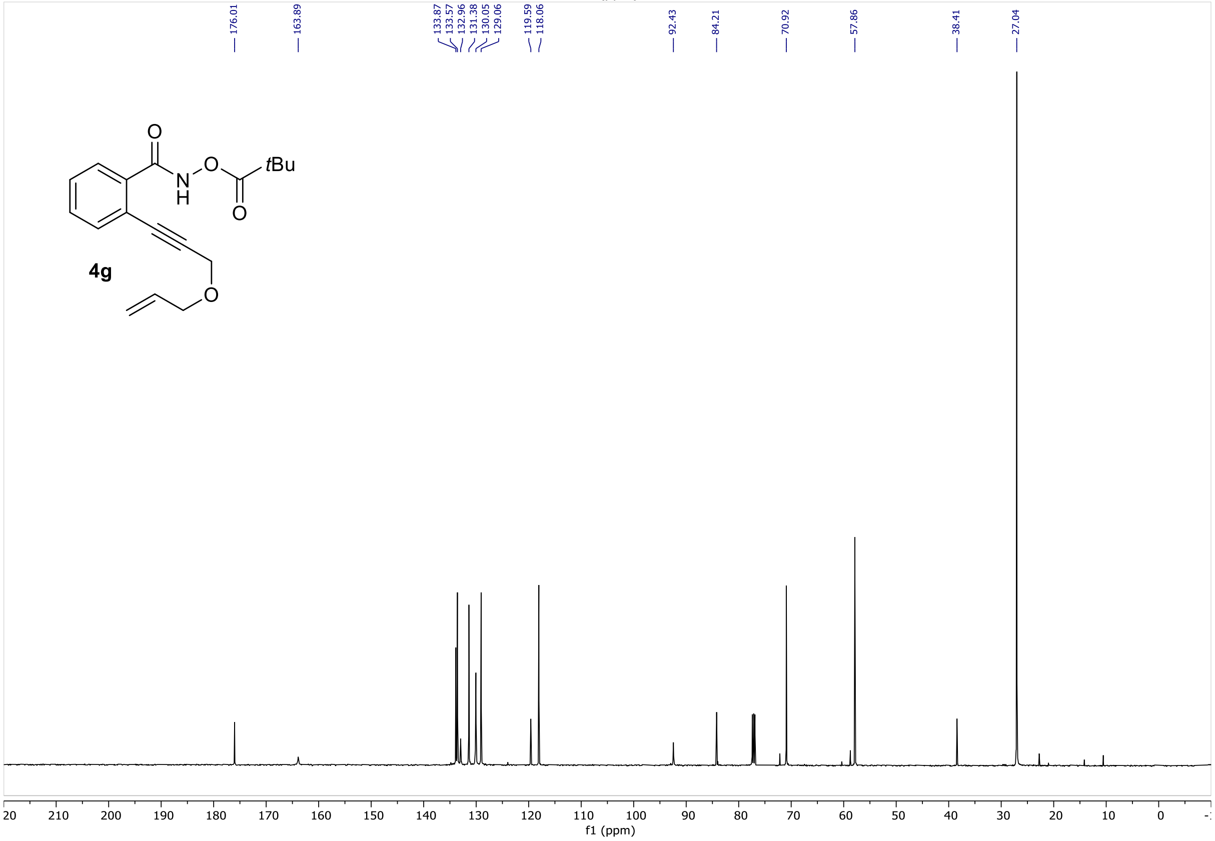
176.01
163.89
133.87
133.57
132.96
131.38
130.05
129.06
119.59
118.06
92.43
84.21
70.92
57.86
38.41
27.04
O
N
H
O
O
tBu
O
4g
f1 (ppm)

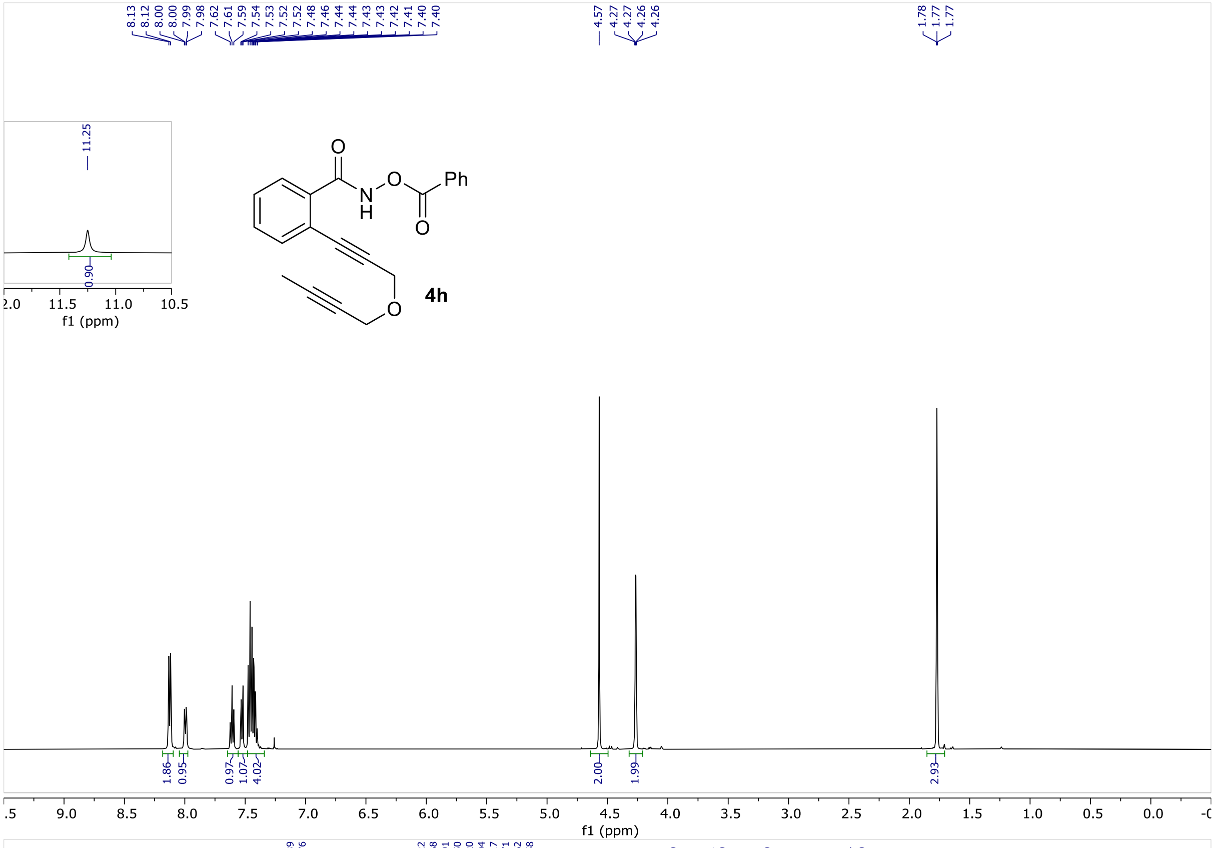
8.13 8.12 8.00 7.99 7.98 7.62 7.61 7.59 7.54 7.53 7.52 7.52 7.48 7.46 7.44 7.44 7.43 7.43 7.42 7.41 7.40 7.40
4.57 4.27 4.27 4.26 4.26
1.78 1.77 1.77
11.25
0.90
f1 (ppm)
Ph
4h
1.86 0.95 0.97 1.07 4.02 2.00 1.99 2.93
f1 (ppm)

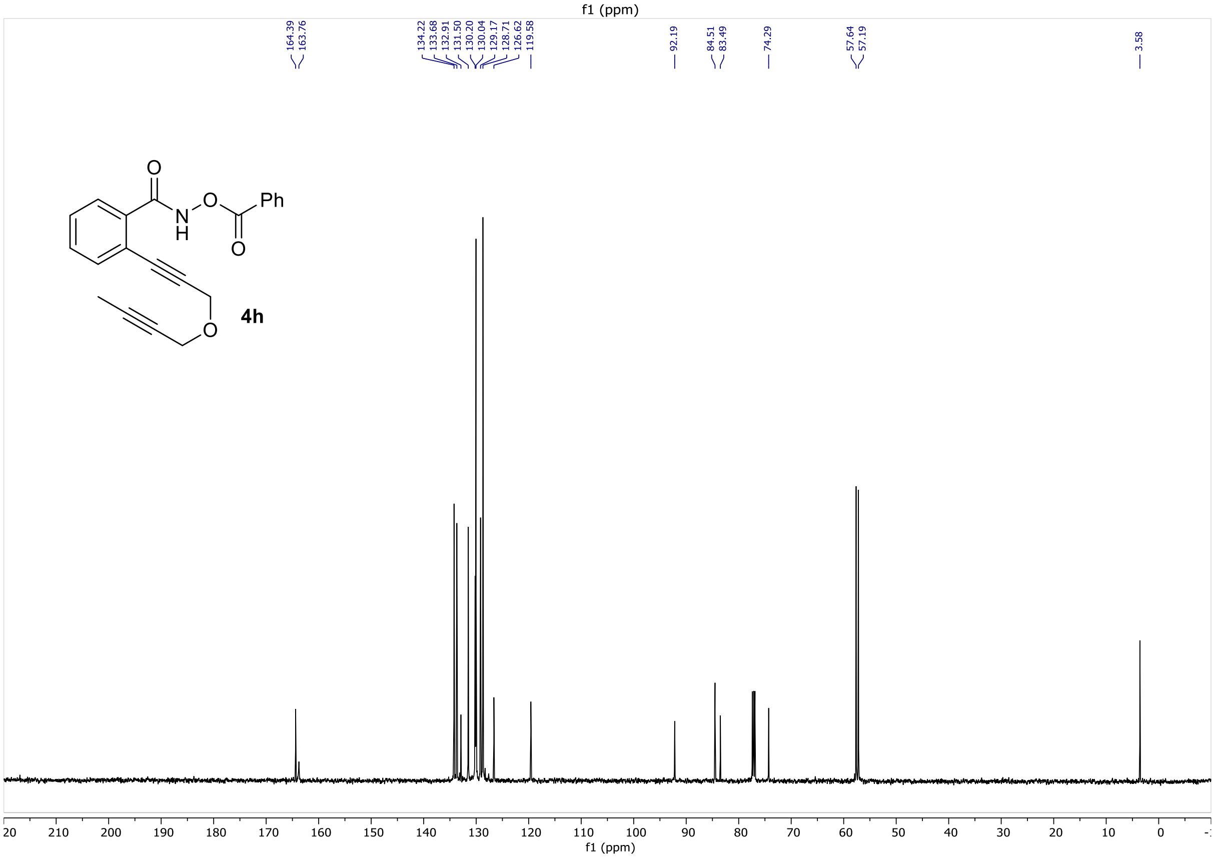
164.39 163.76
134.22 133.68 132.91 131.50 130.20 130.04 129.17 128.71 126.62 119.58
92.19
84.51 83.49
74.29
57.64 57.19
3.58
Ph
4h
f1 (ppm)

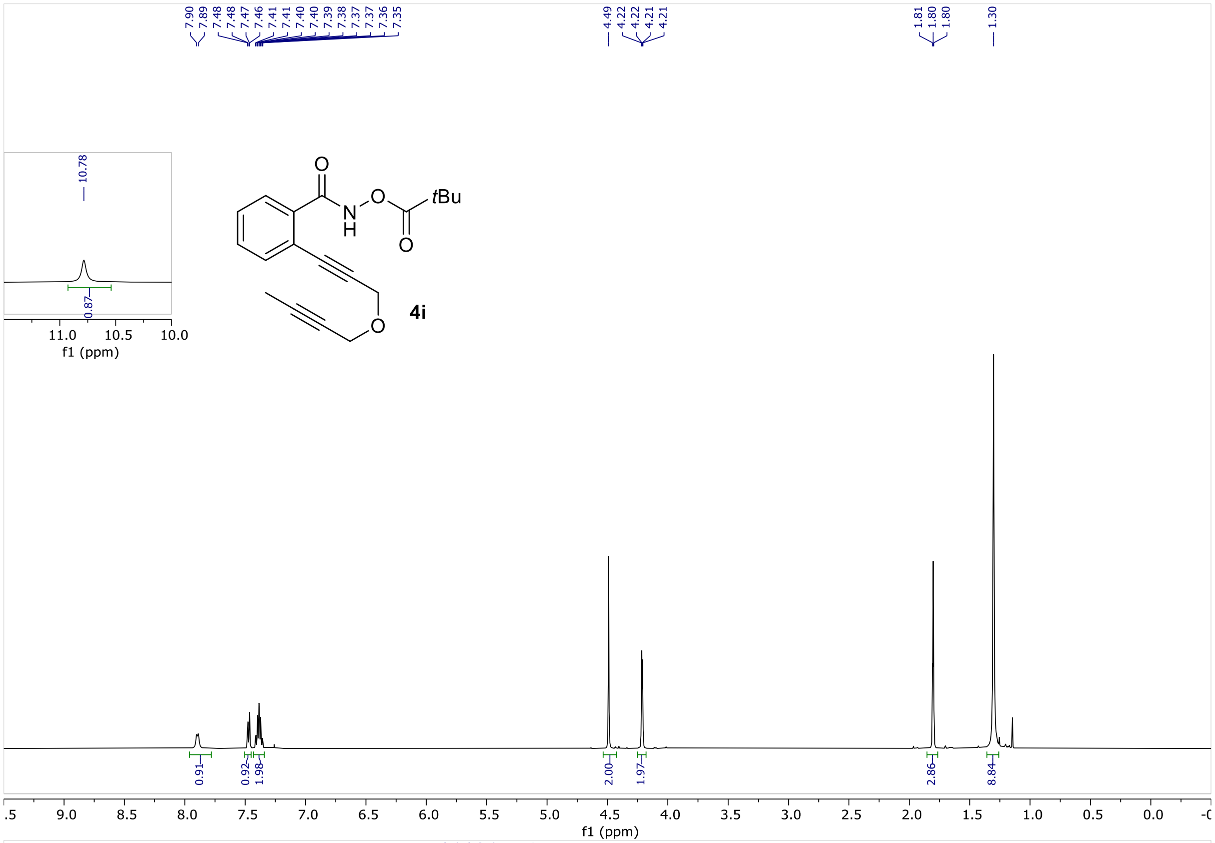

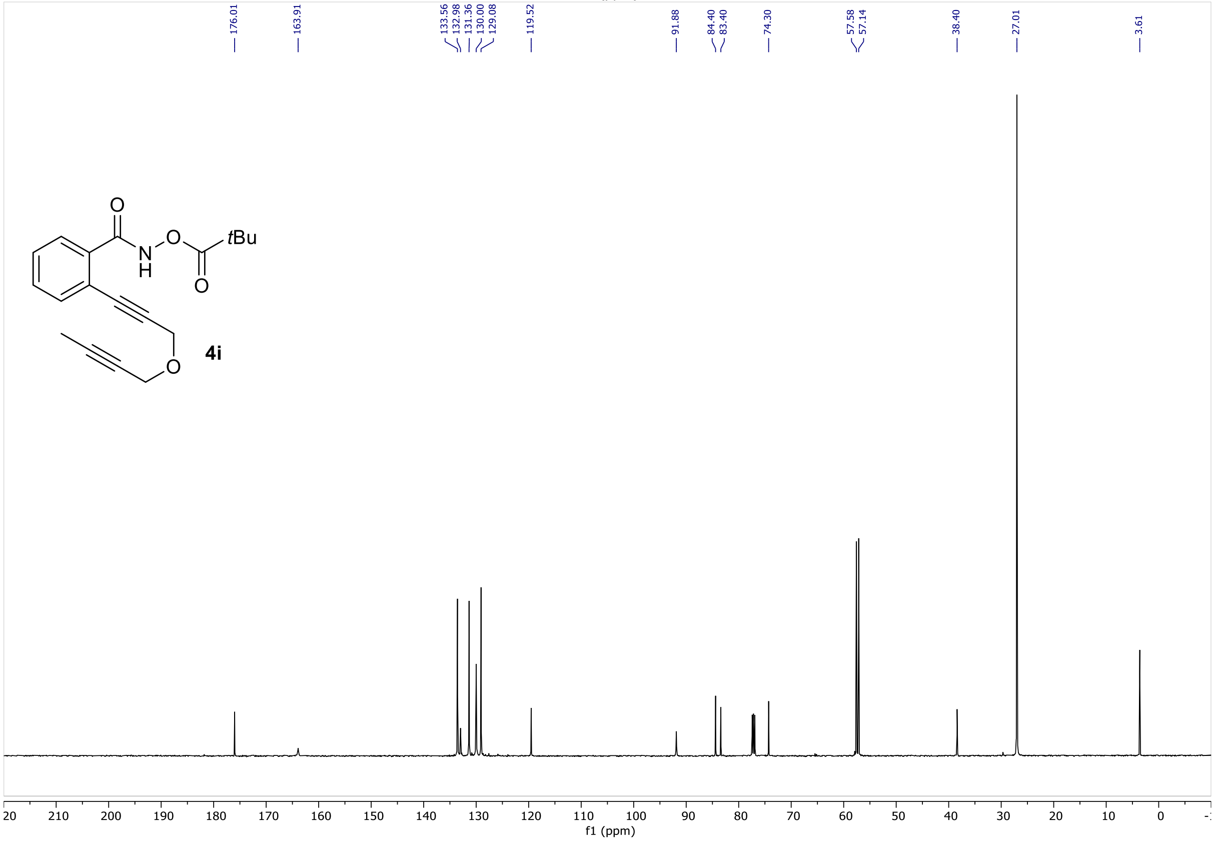

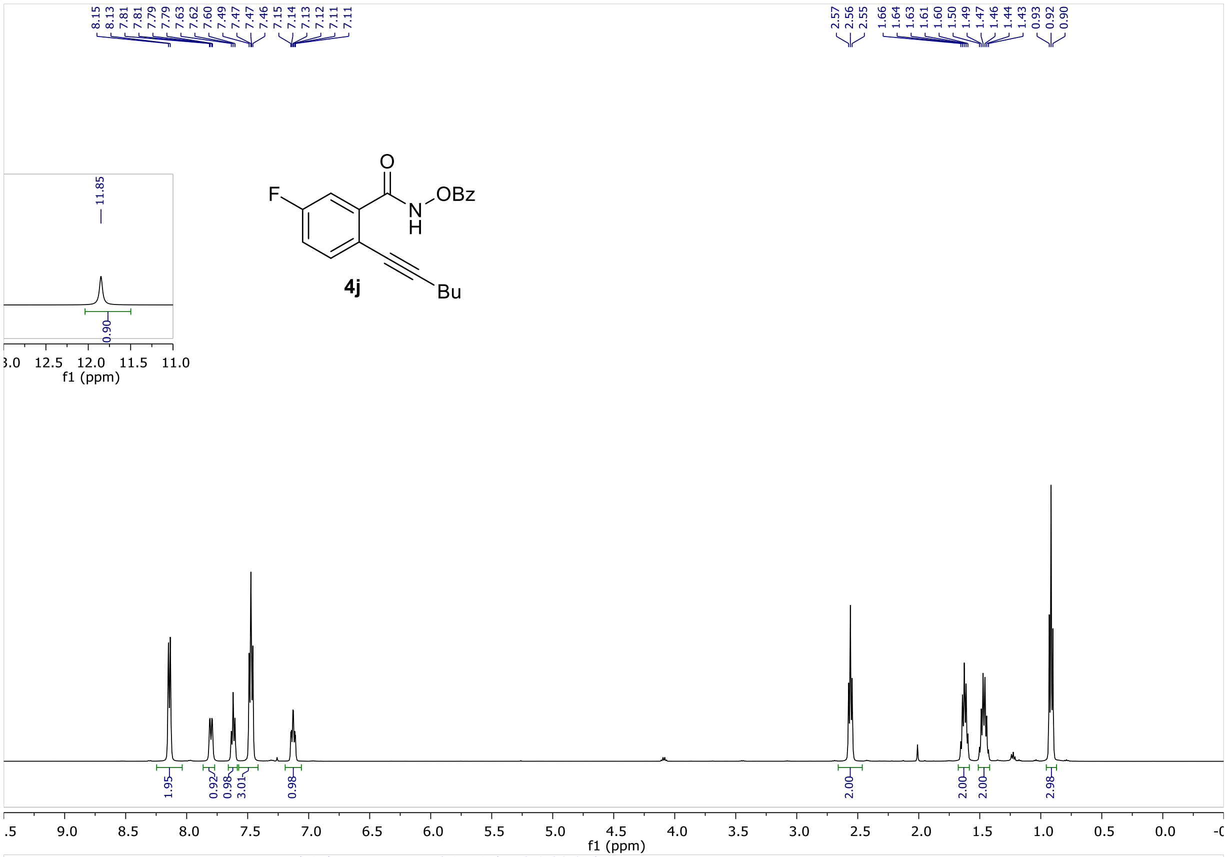

8.15
8.13
7.81
7.81
7.79
7.79
7.63
7.62
7.60
7.49
7.47
7.47
7.46
7.15
7.14
7.13
7.12
7.11
7.11
2.57
2.56
2.55
1.66
1.64
1.63
1.61
1.60
1.50
1.49
1.47
1.46
1.44
1.43
0.93
0.92
0.90
11.85
0.90
F
O
N
OBz
H
4j
Bu
1.95
0.92
0.98
3.01
0.98
2.00
2.00
2.00
2.98
f1 (ppm)

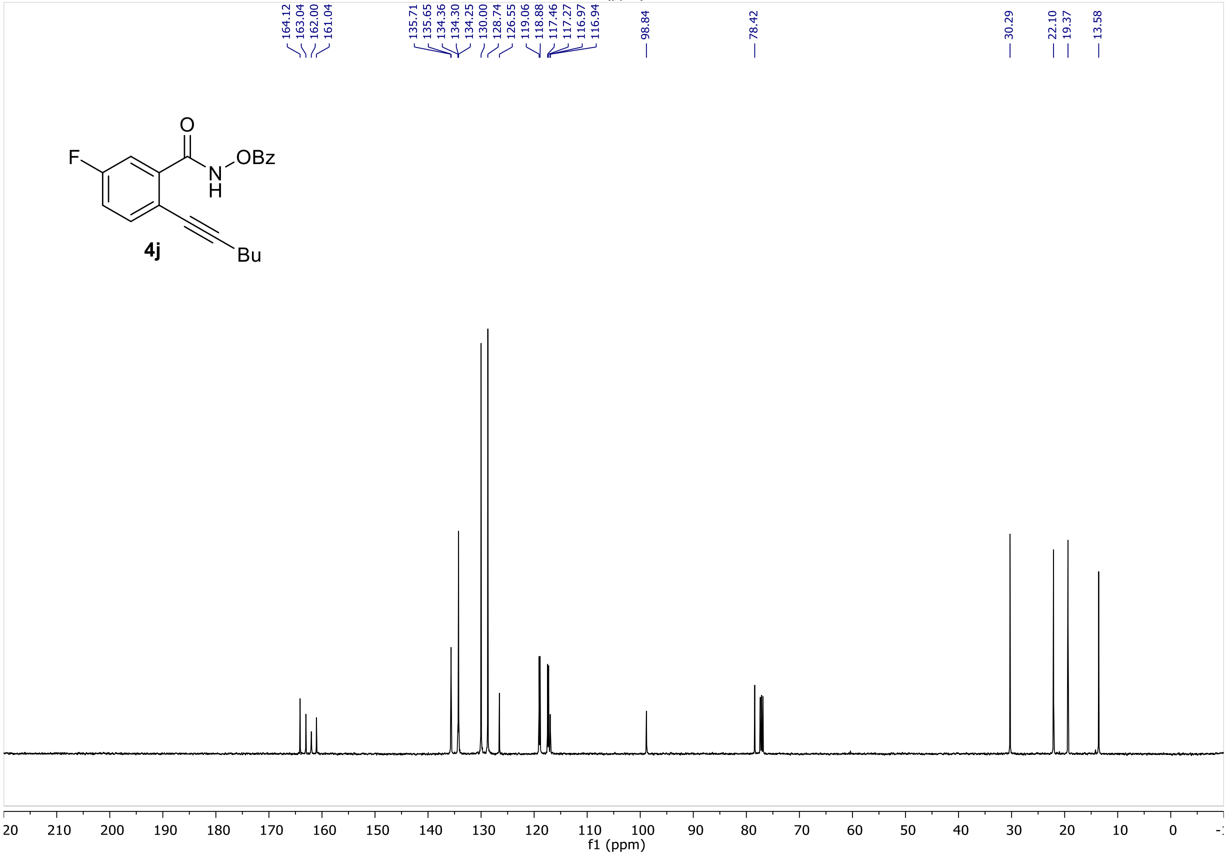

164.12
163.04
162.00
161.04
135.71
135.65
134.36
134.30
134.25
130.00
128.74
126.55
119.06
118.88
117.46
117.27
116.97
116.94
98.84
78.42
30.29
22.10
19.37
13.58
F
O
N
OBz
H
4j
Bu
f1 (ppm)

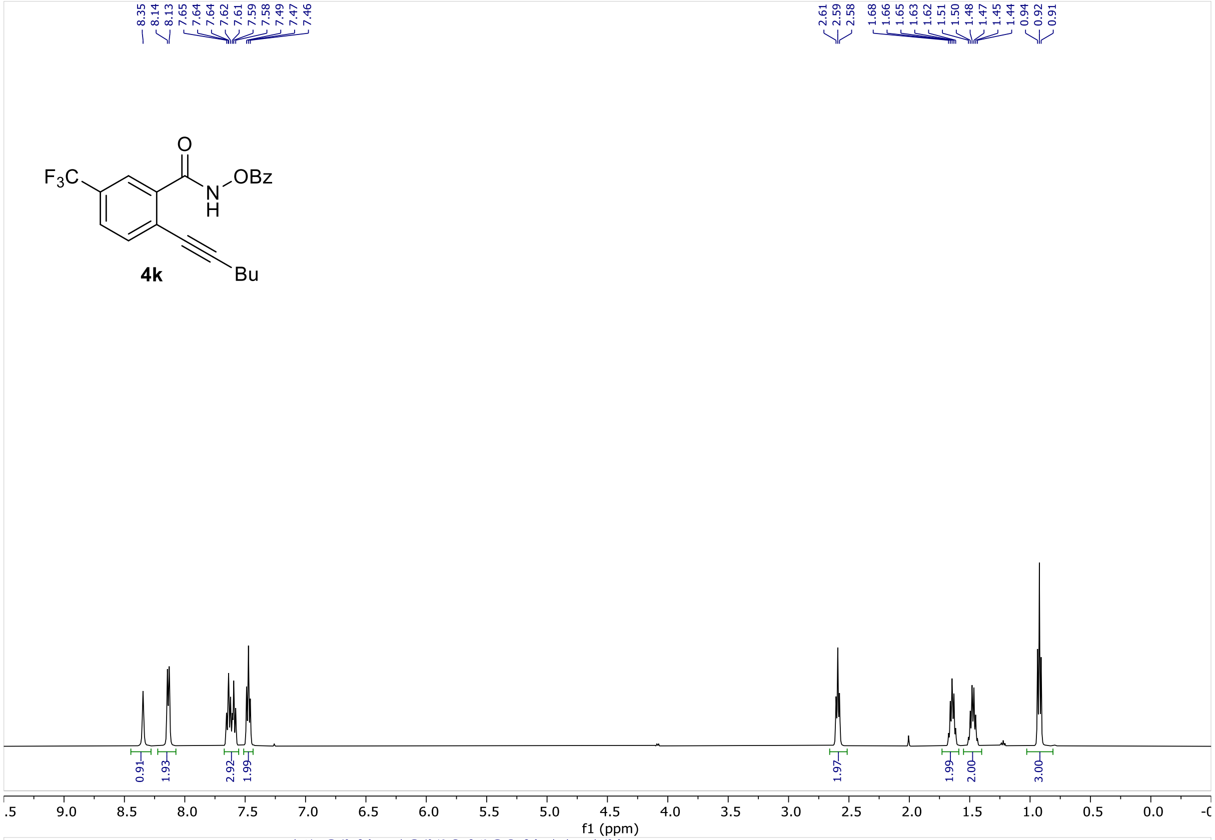

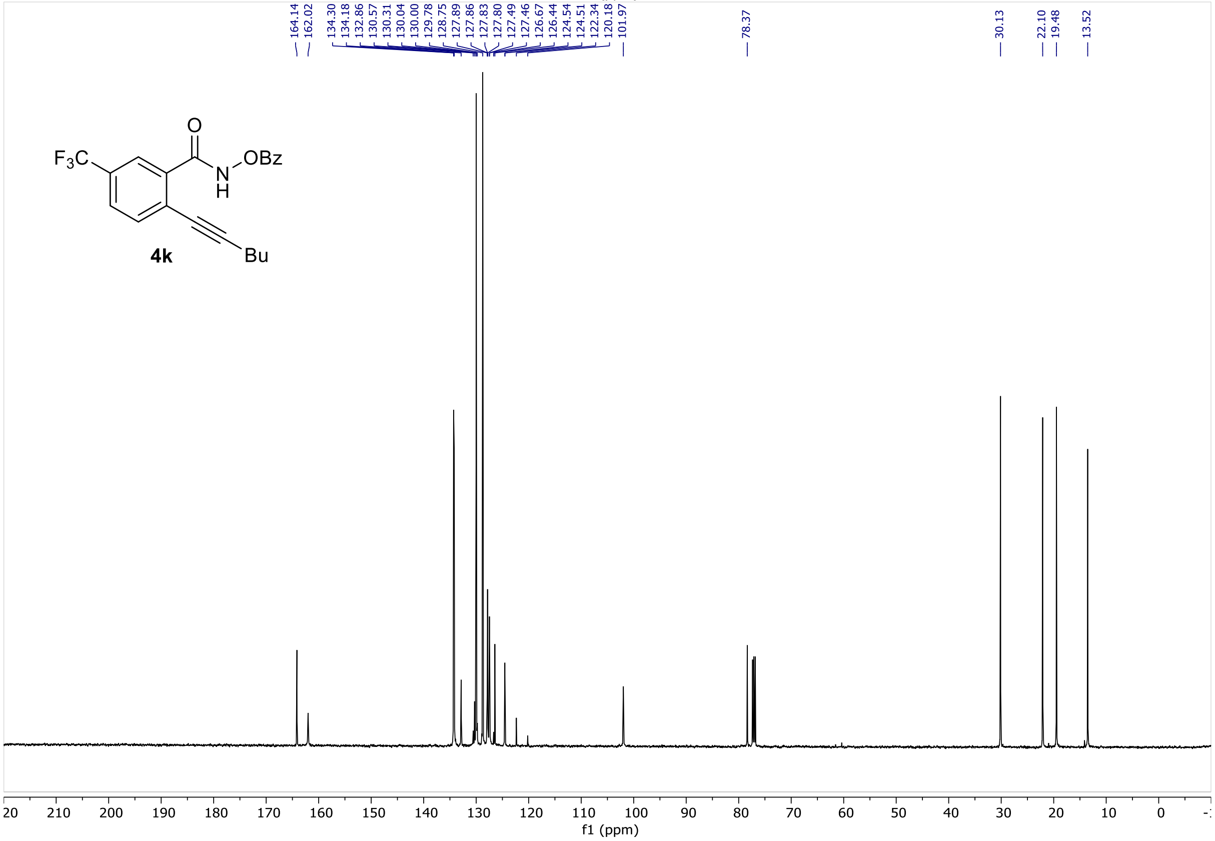

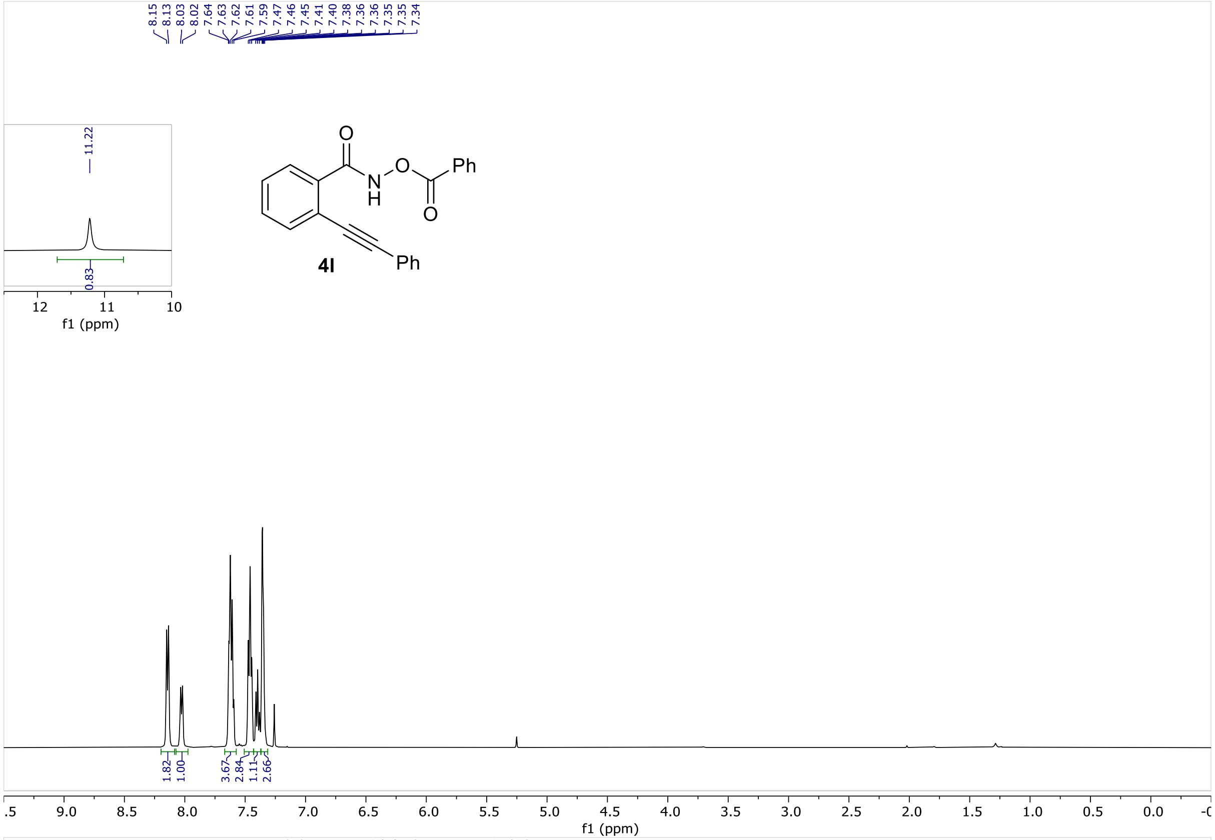
8.15
8.13
8.03
8.02
7.64
7.63
7.62
7.61
7.59
7.47
7.46
7.45
7.41
7.40
7.38
7.36
7.36
7.35
7.35
7.34
11.22
0.83
f1 (ppm)
Ph
Ph
O
O
N
H
O
4l
1.82
1.00
3.67
2.84
1.11
2.66
f1 (ppm)

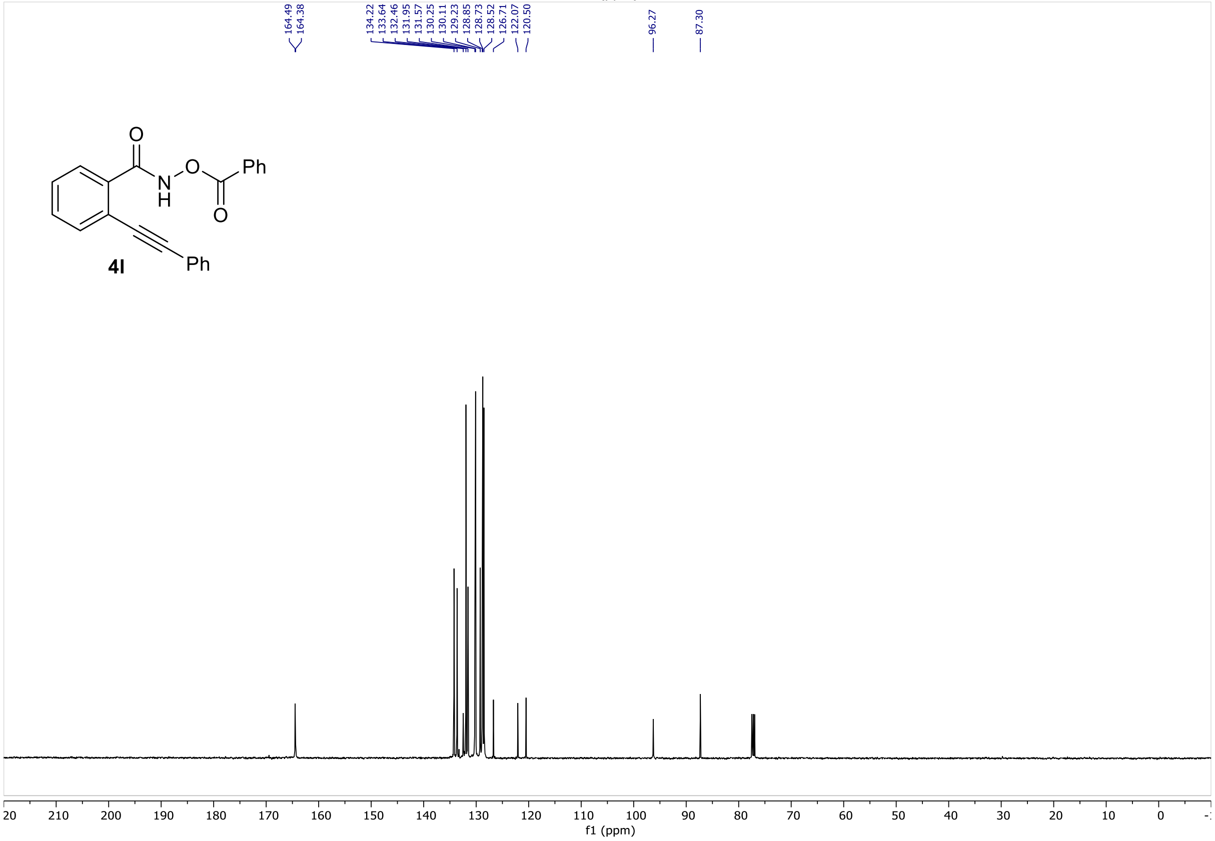
164.49
164.38
134.22
133.64
132.46
131.95
131.57
130.25
130.11
129.23
128.85
128.73
128.52
126.71
122.07
120.50
96.27
87.30
Ph
Ph
O
O
N
H
O
4l
f1 (ppm)

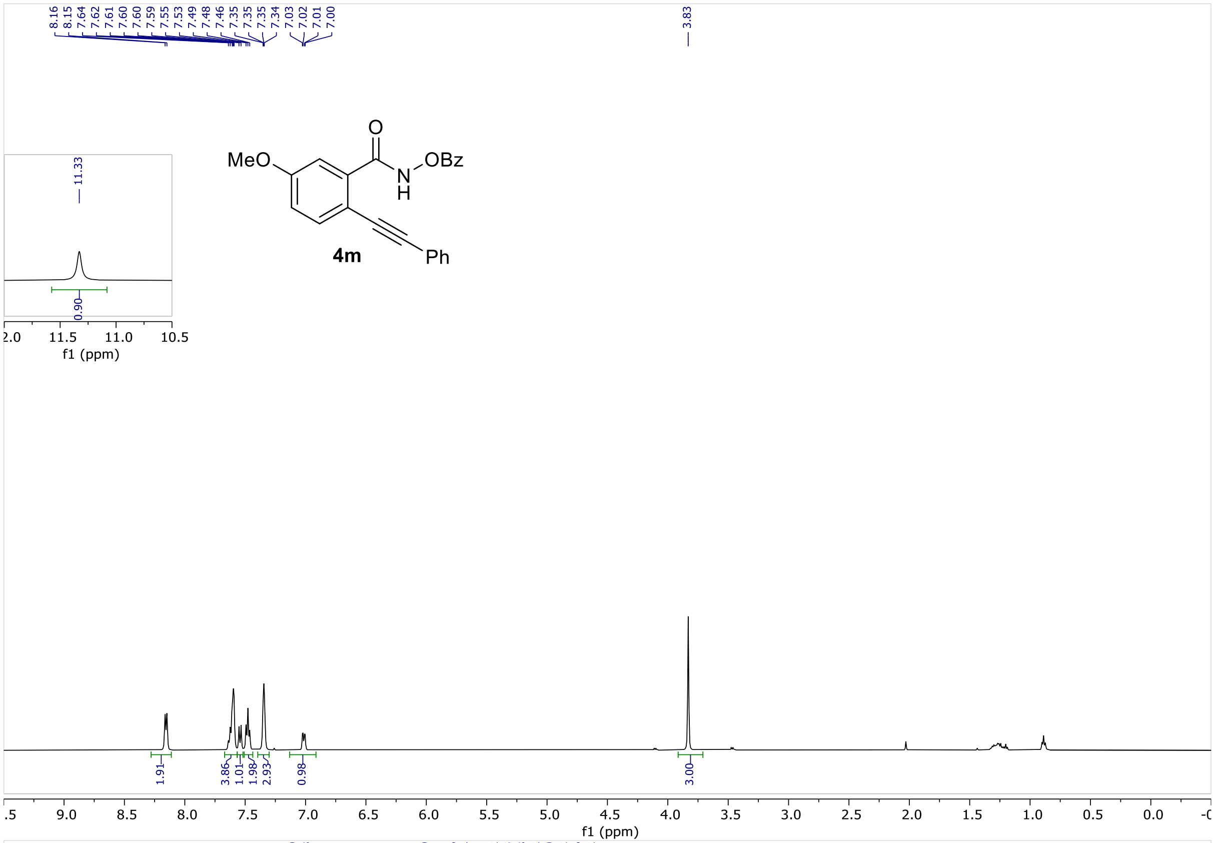
8.16
8.15
7.64
7.62
7.61
7.60
7.60
7.59
7.55
7.53
7.49
7.48
7.46
7.35
7.35
7.35
7.34
7.03
7.02
7.01
7.00
3.83
11.33
0.90
MeO
O
N
OBz
H
Ph
4m
1.91
3.86
1.01
1.98
2.93
0.98
3.00
f1 (ppm)

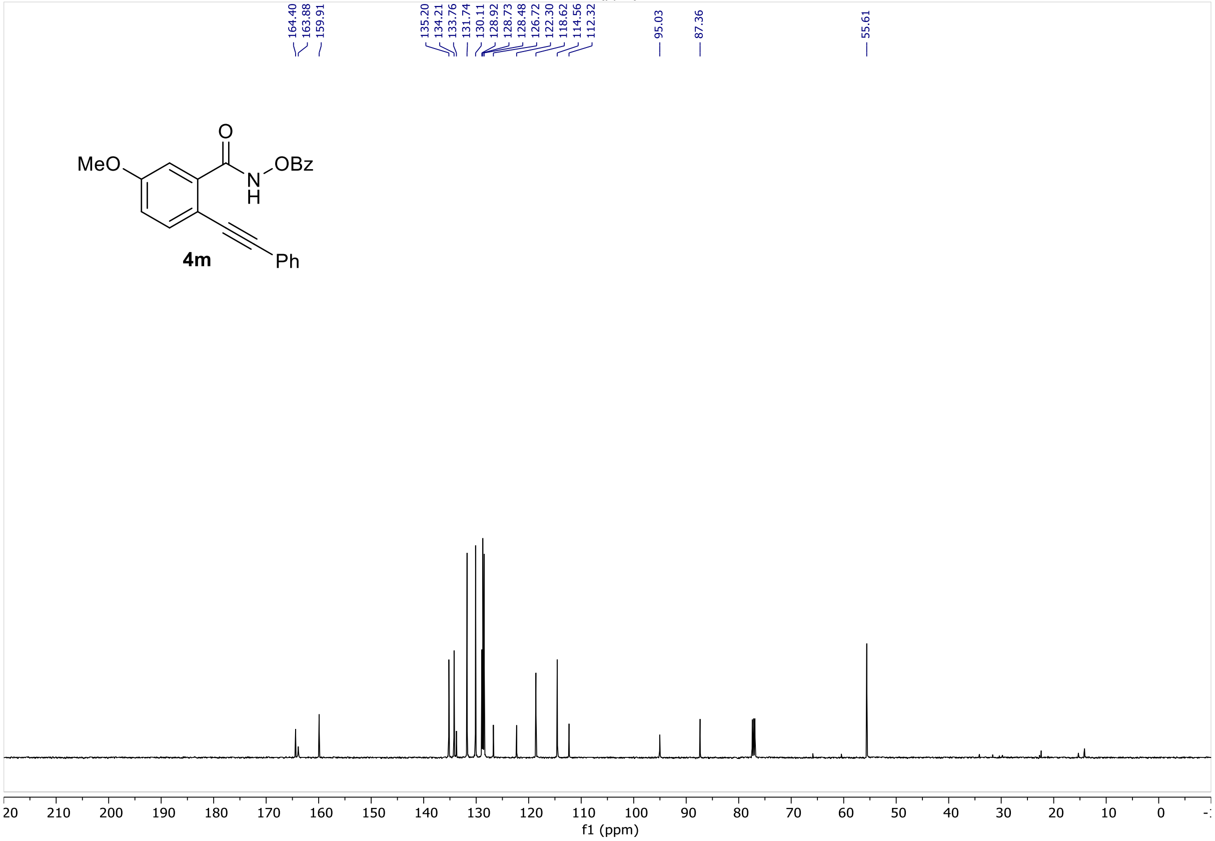
164.40
163.88
159.91
135.20
134.21
133.76
131.74
130.11
128.92
128.73
128.48
126.72
122.30
118.62
114.56
112.32
95.03
87.36
55.61
MeO
O
N
OBz
H
Ph
4m
f1 (ppm)

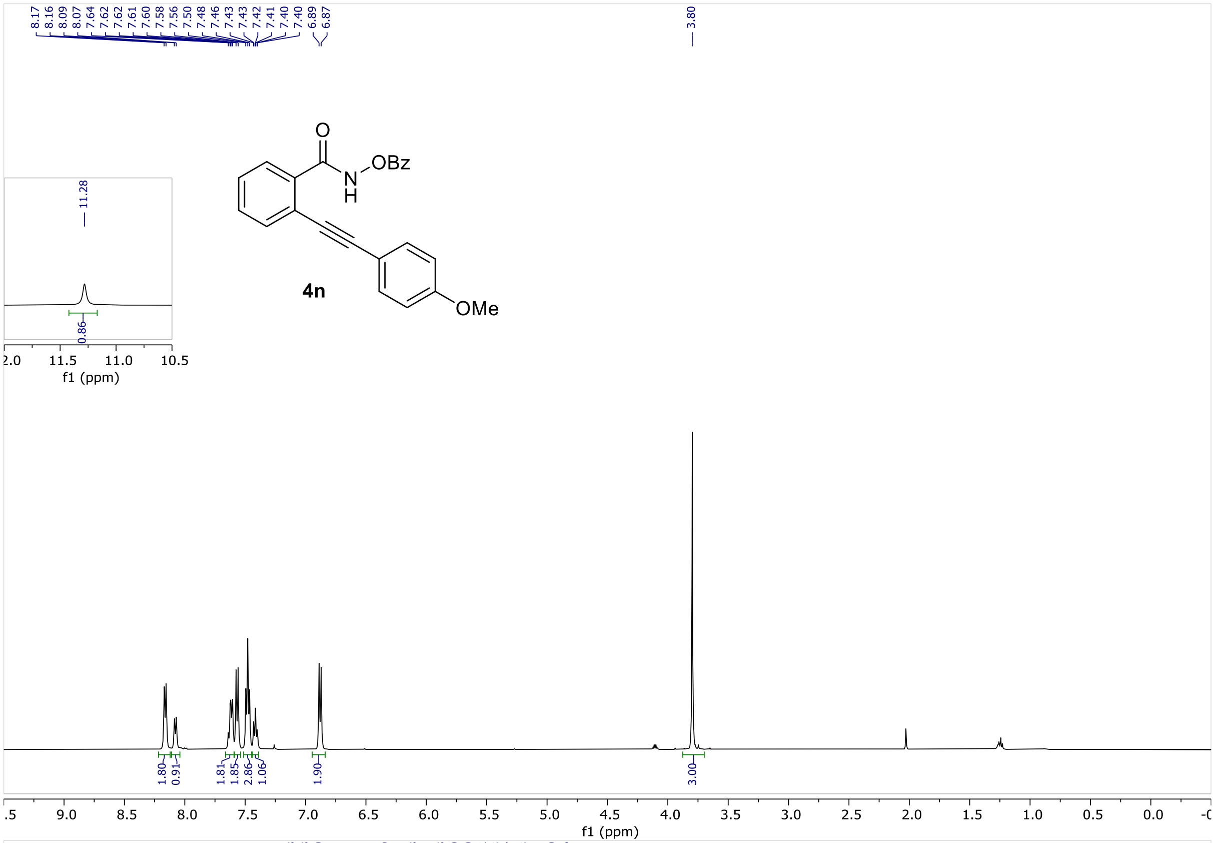

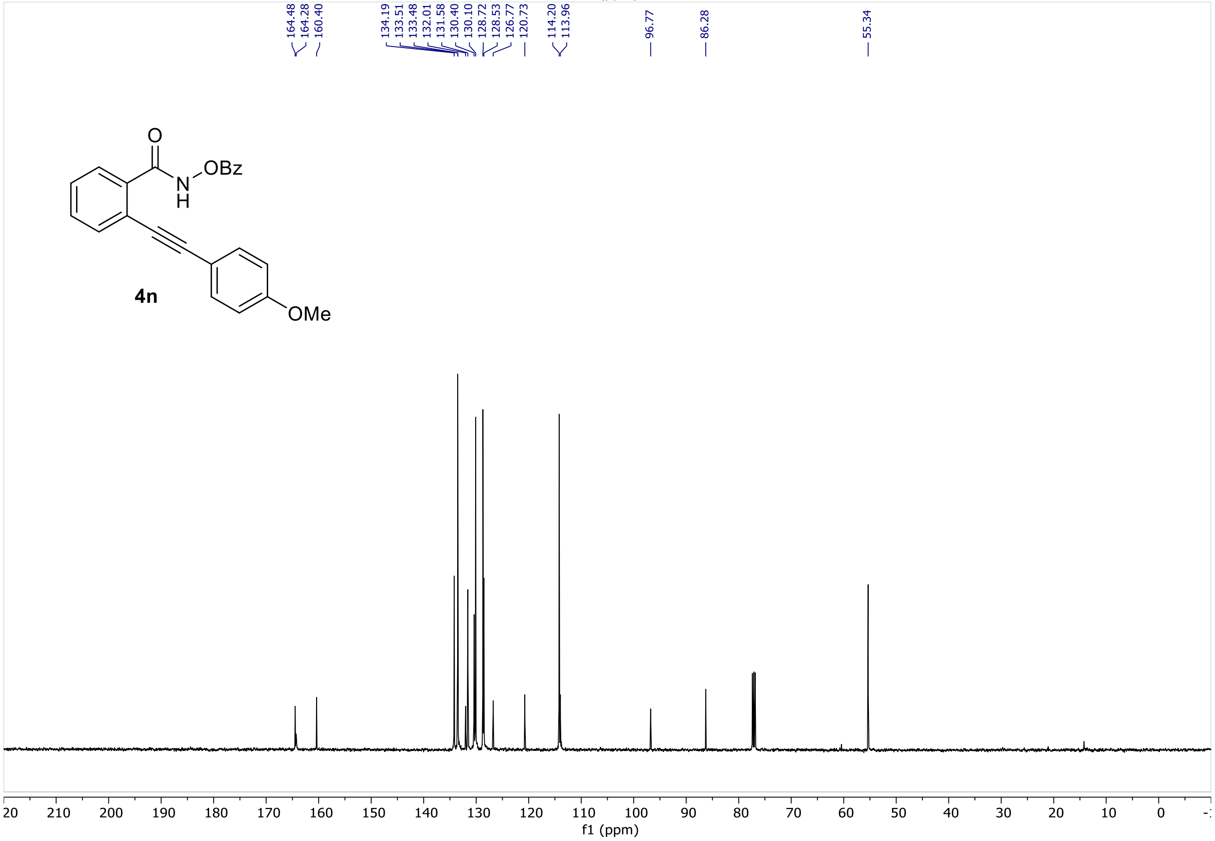

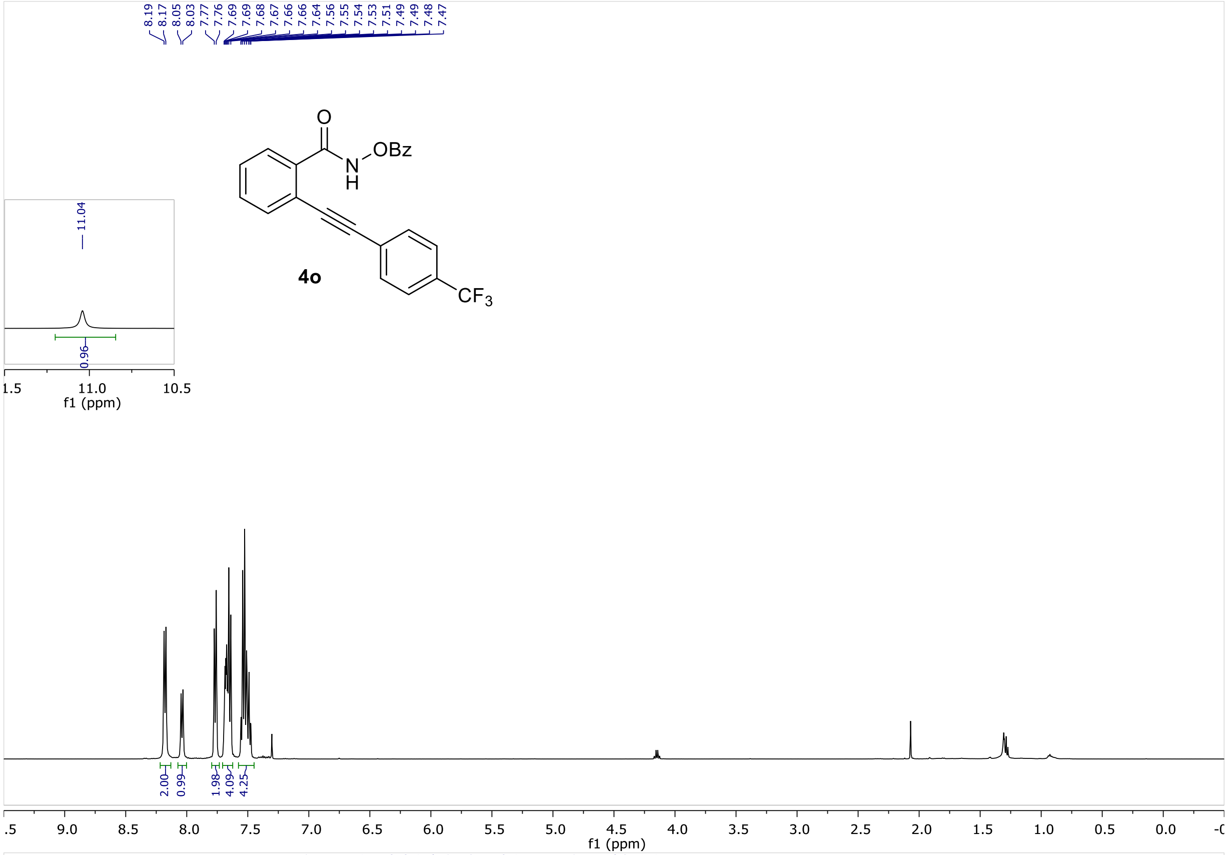

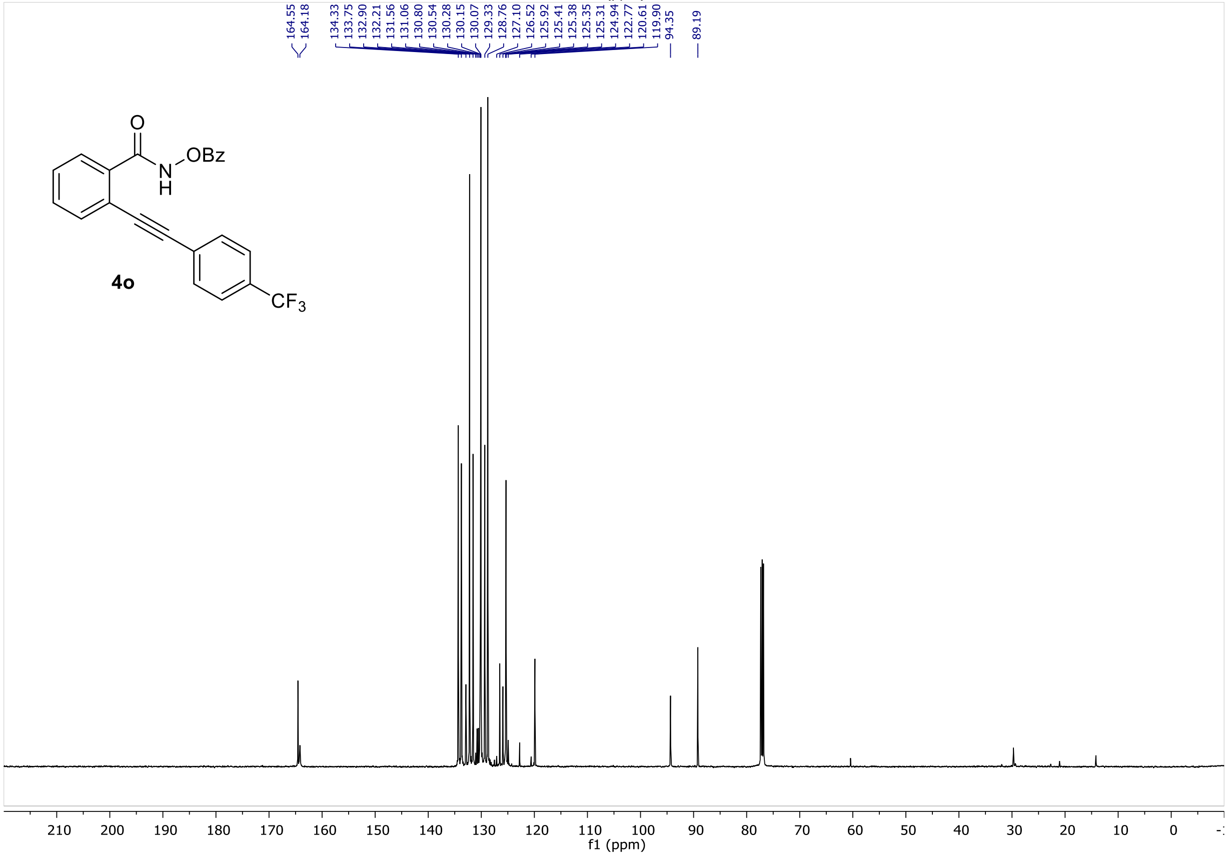

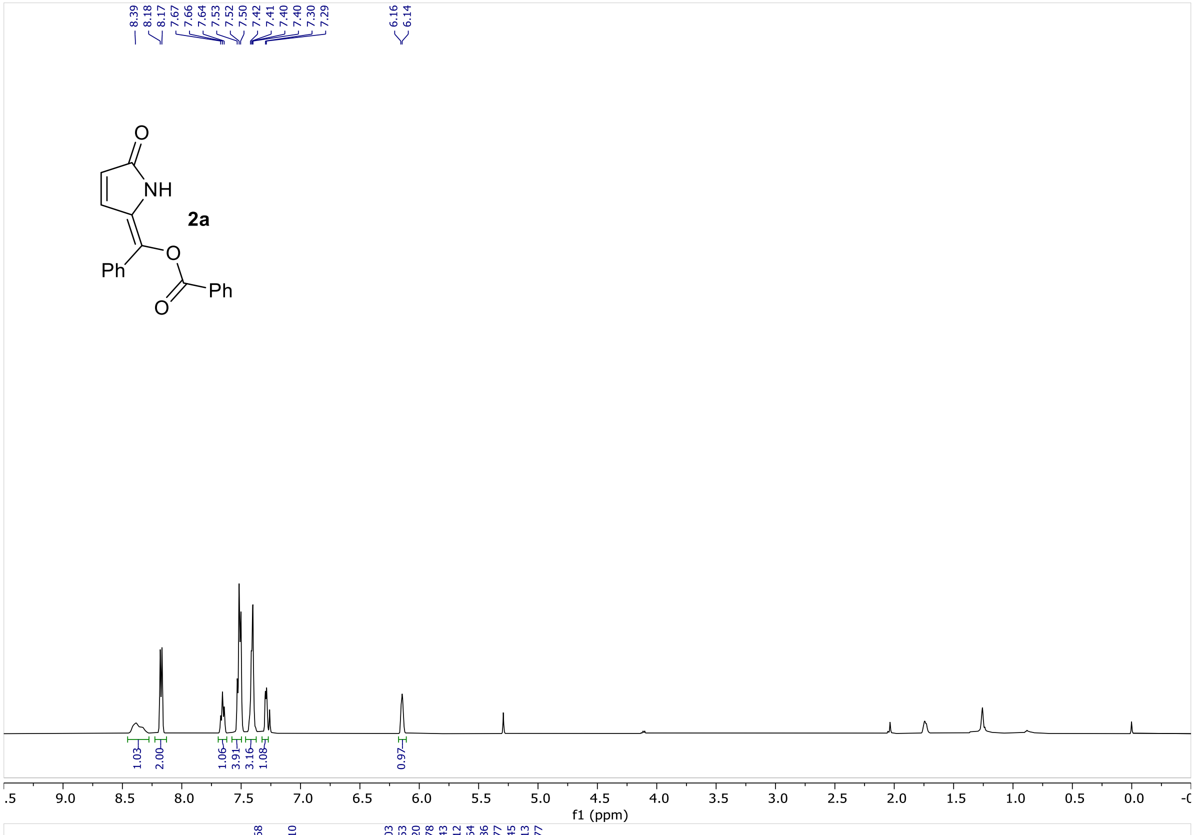
8.39
8.18
8.17
7.67
7.66
7.64
7.53
7.52
7.50
7.42
7.41
7.40
7.40
7.30
7.29
6.16
6.14
NH
2a
O
Ph
Ph
1.03
2.00
1.06
3.91
3.16
1.08
0.97
f1 (ppm)

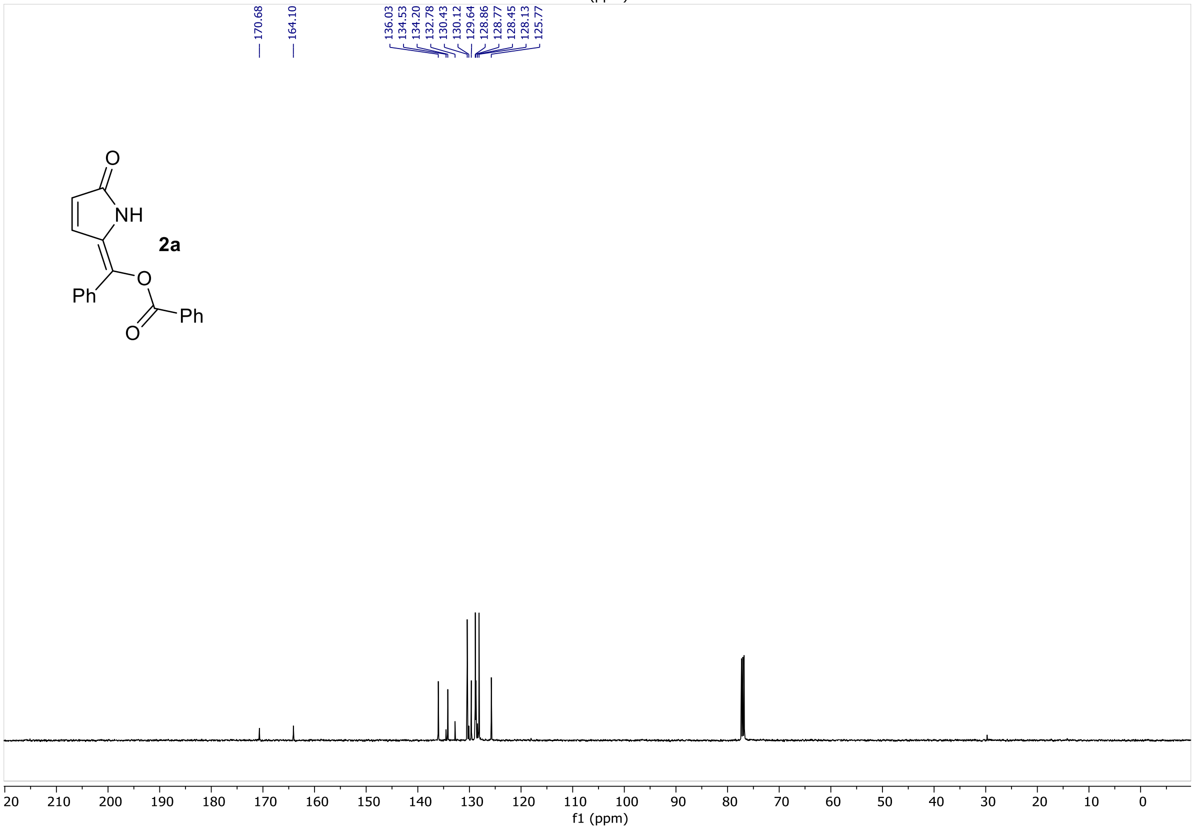
170.68
164.10
136.03
134.53
134.20
132.78
130.43
130.12
129.64
128.86
128.77
128.45
128.13
125.77
NH
2a
O
Ph
Ph
f1 (ppm)

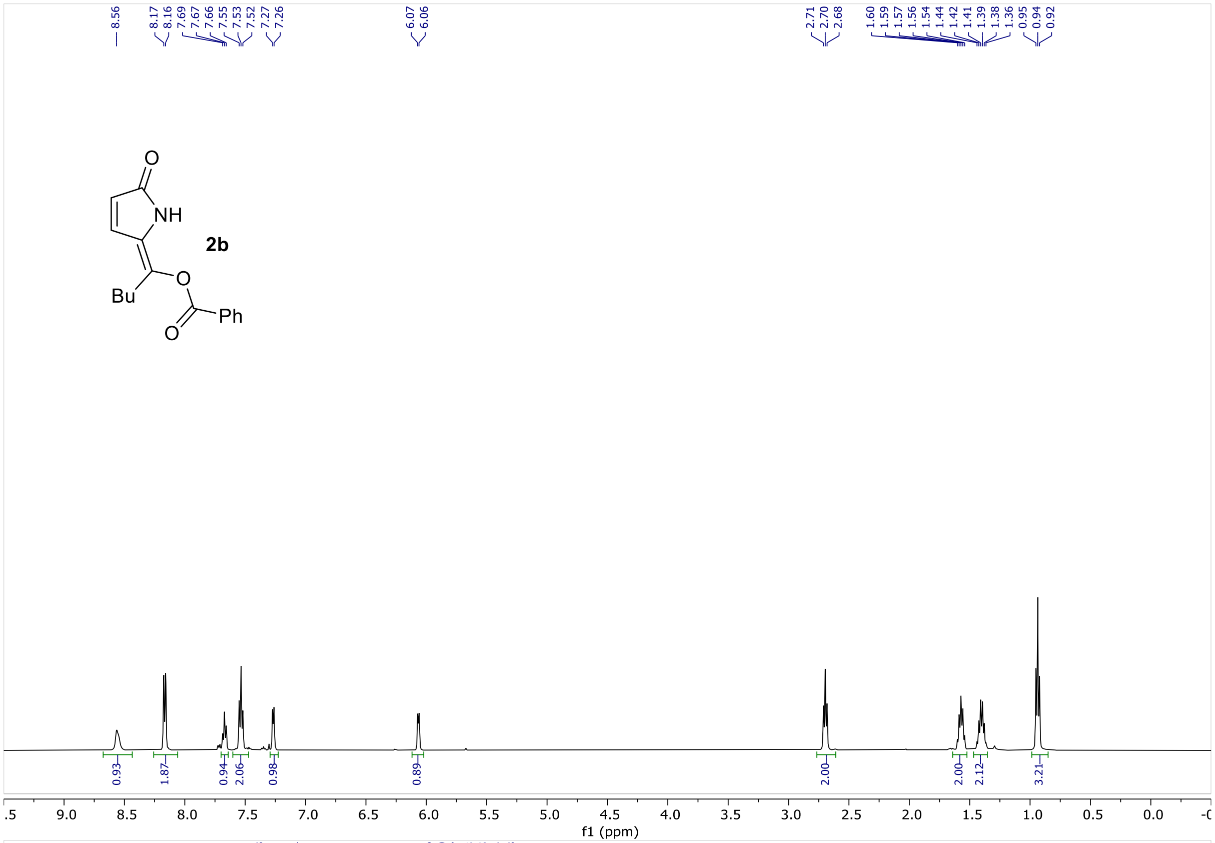

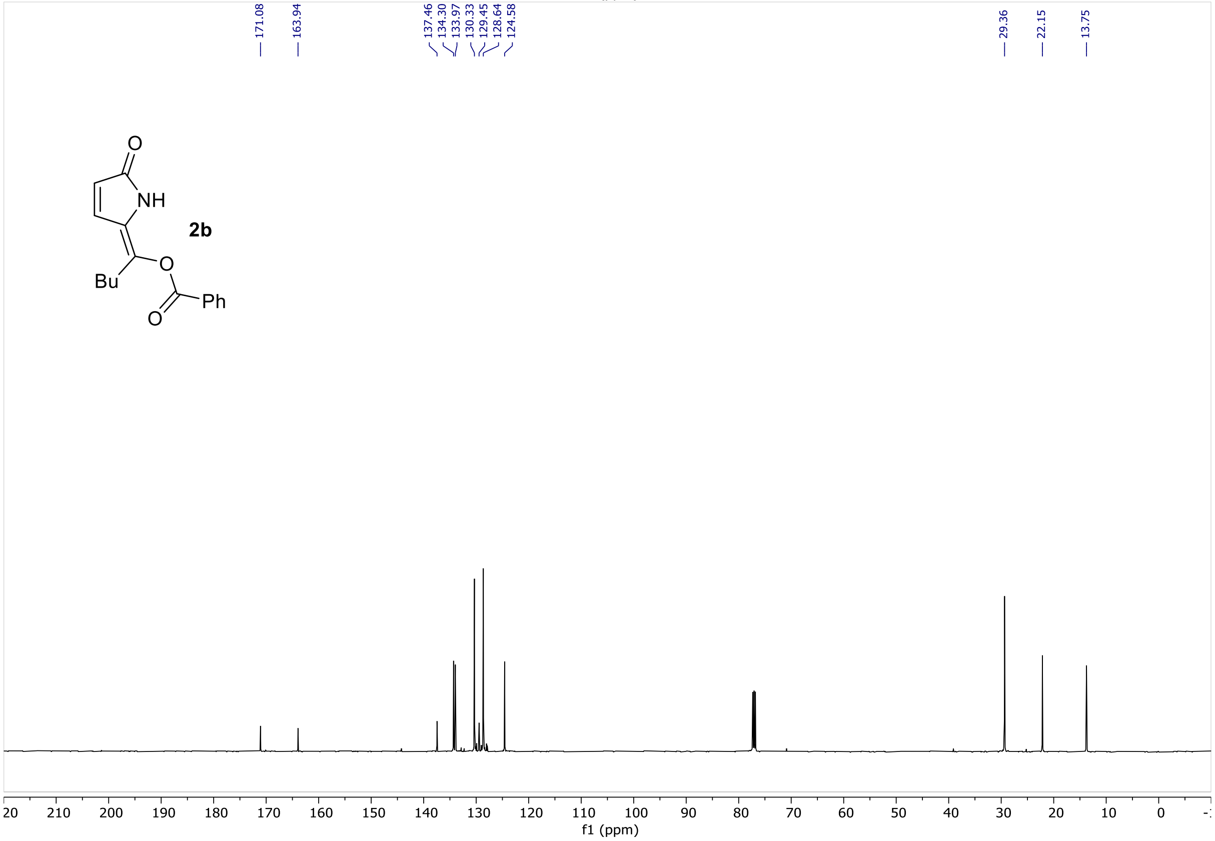

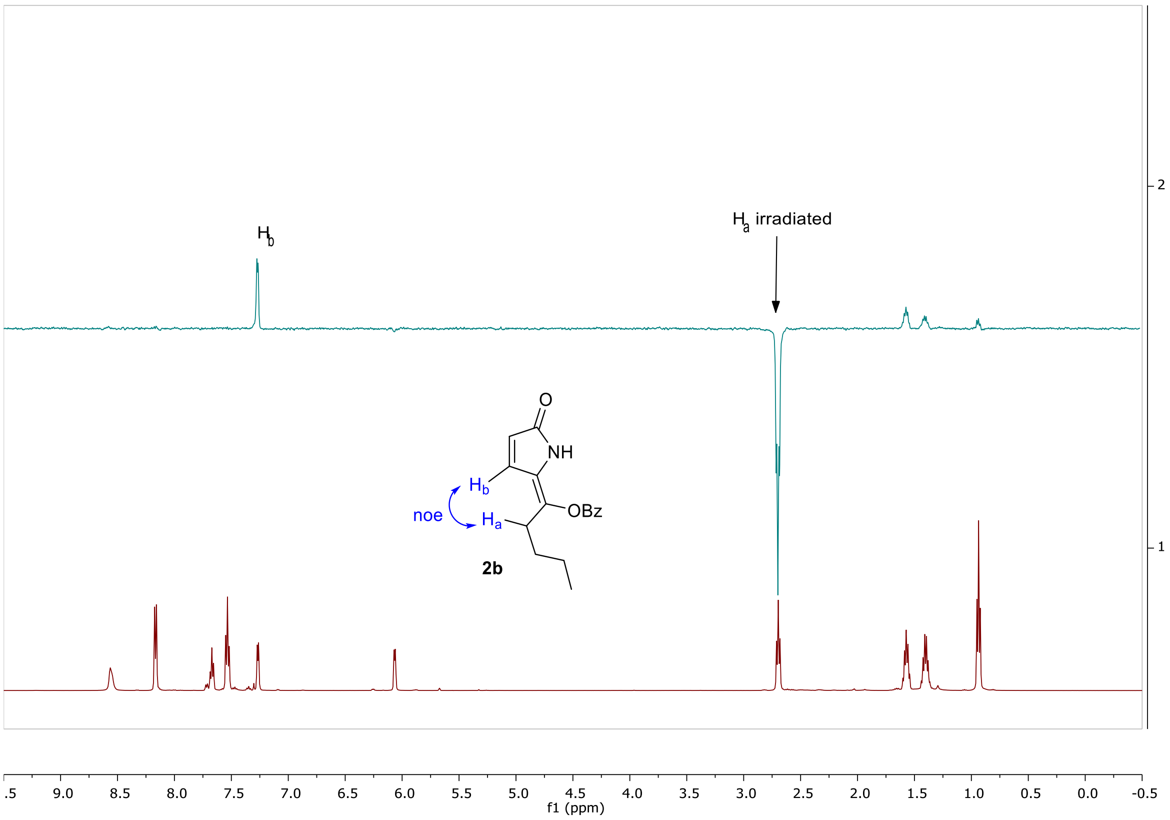

H_b
H_a irradiated
O
NH
H_b
noe
H_a
OBz
2b
2
1
.5
9.0
8.5
8.0
7.5
7.0
6.5
6.0
5.5
5.0
4.5
4.0
3.5
3.0
2.5
2.0
1.5
1.0
0.5
0.0
-0.5
f1 (ppm)

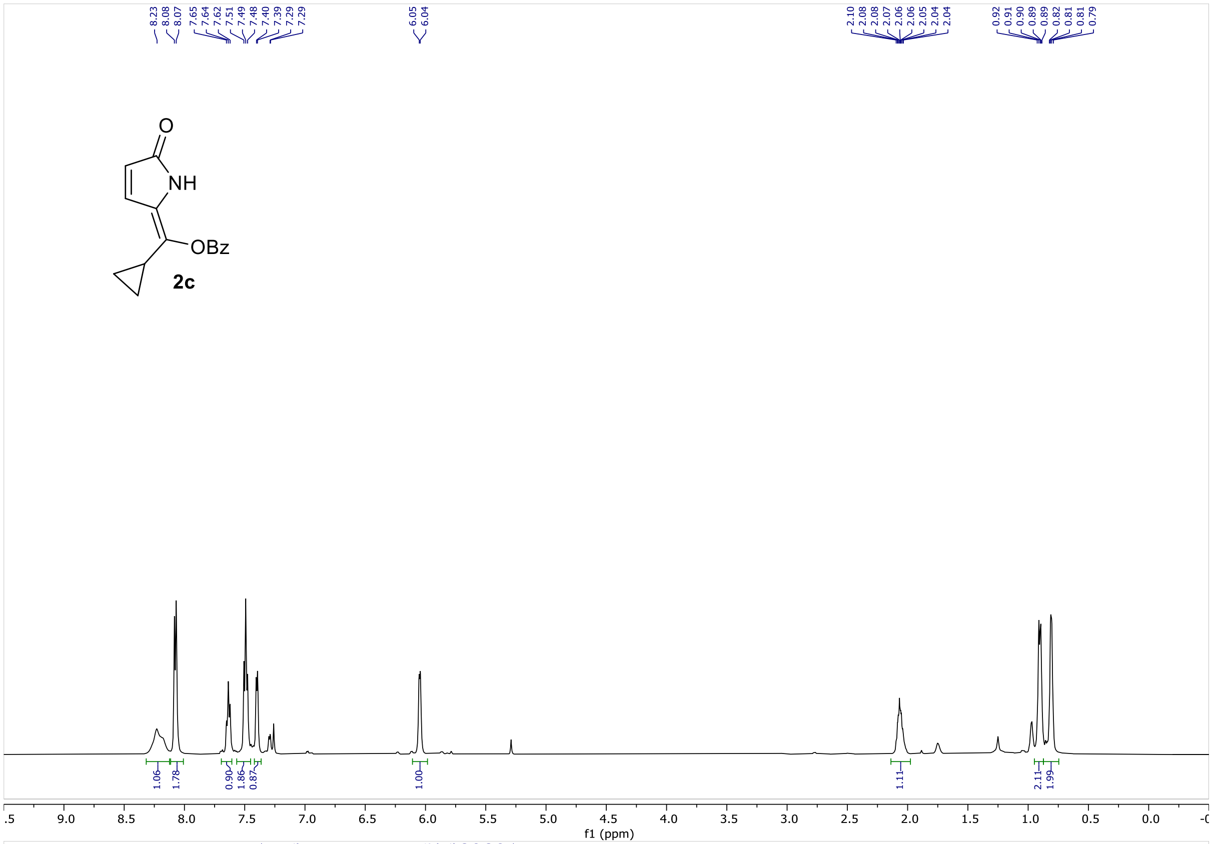
8.23
8.08
8.07
7.65
7.64
7.62
7.51
7.49
7.48
7.40
7.39
7.29
7.29
6.05
6.04
2.10
2.08
2.08
2.07
2.06
2.06
2.05
2.04
2.04
0.92
0.91
0.90
0.89
0.89
0.82
0.81
0.81
0.79
O
NH
OBz
2c
1.06
1.78
0.90
1.86
0.87
1.00
1.11
2.11
1.99
f1 (ppm)

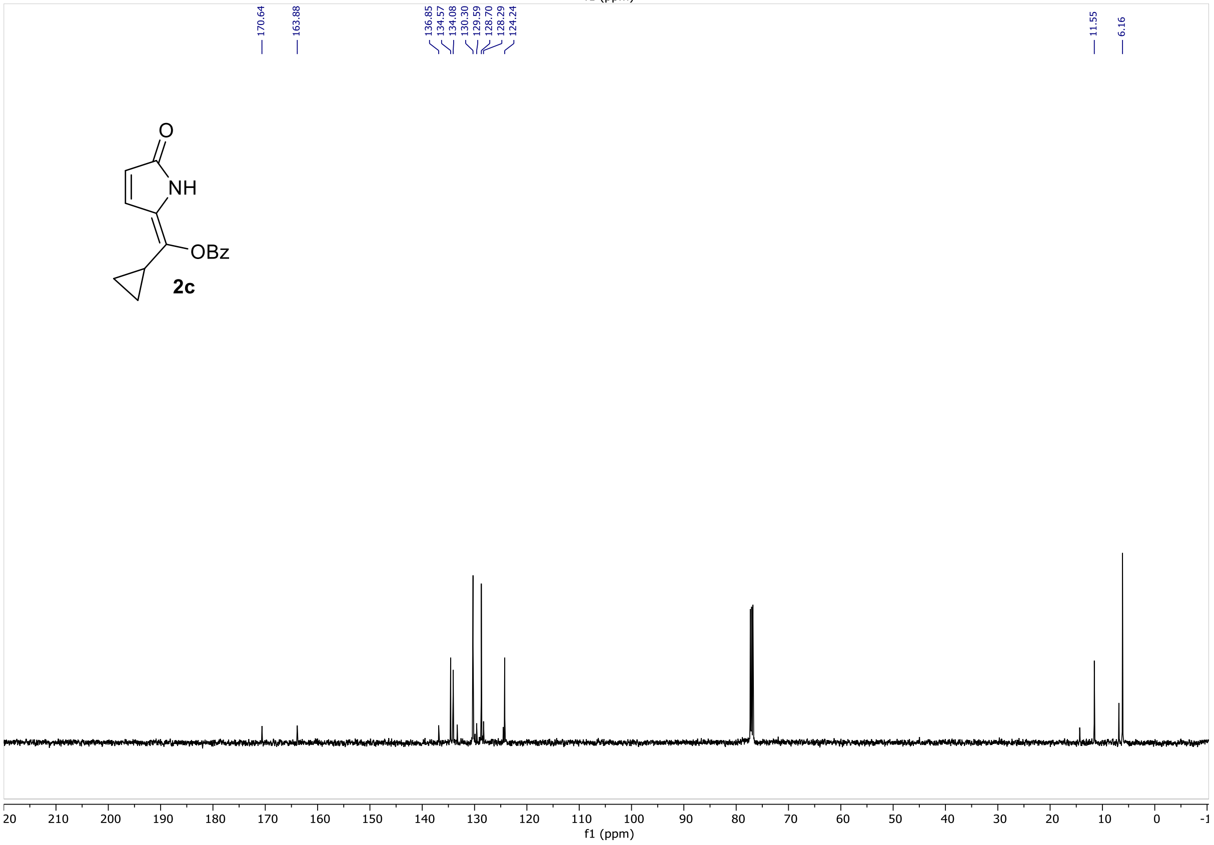
170.64
163.88
136.85
134.57
134.08
130.30
129.59
128.70
128.29
124.24
11.55
6.16
O
NH
OBz
2c
f1 (ppm)

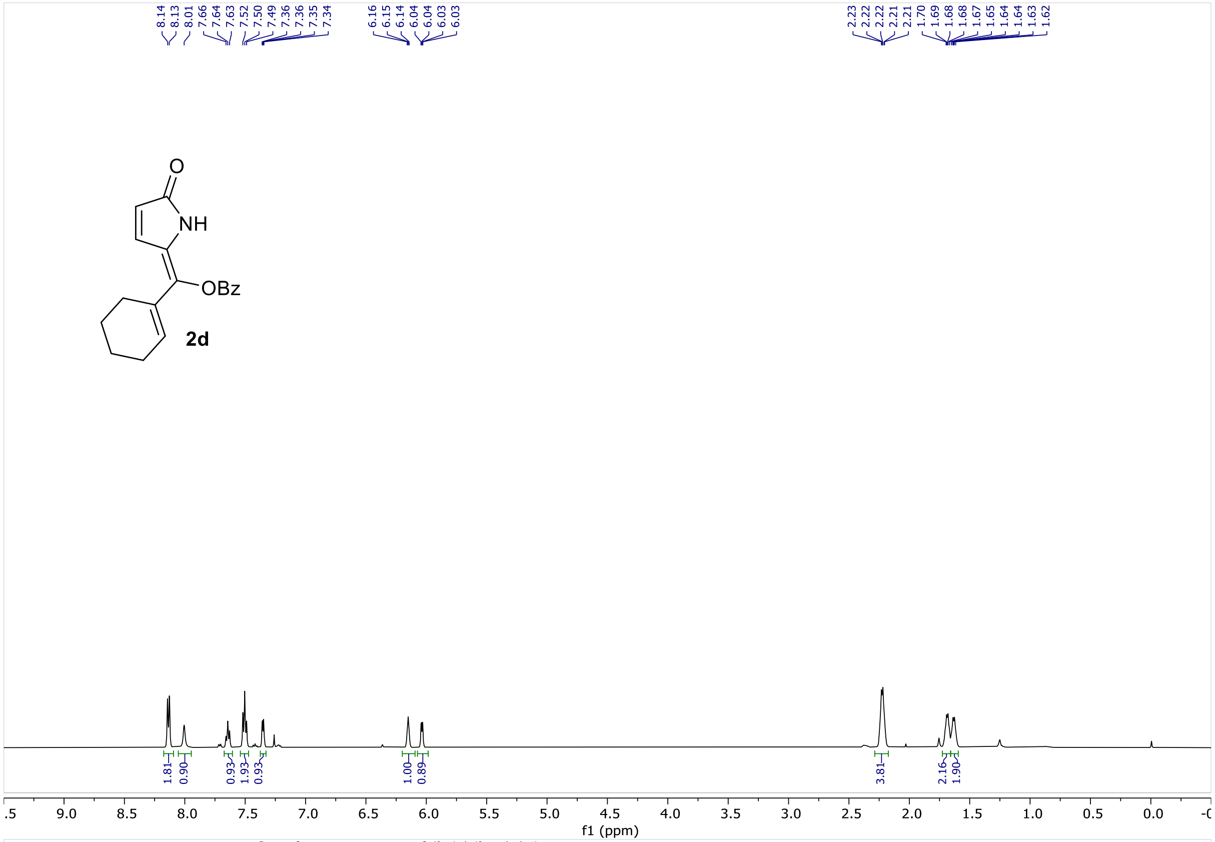
8.14
8.13
8.01
7.66
7.64
7.63
7.52
7.50
7.49
7.36
7.36
7.35
7.34
6.16
6.15
6.14
6.04
6.04
6.03
6.03
2.23
2.22
2.22
2.21
2.21
1.70
1.69
1.68
1.68
1.67
1.65
1.64
1.64
1.63
1.62
O
NH
OBz
2d
1.81
0.90
0.93
1.93
0.93
1.00
0.89
3.81
2.16
1.90
f1 (ppm)

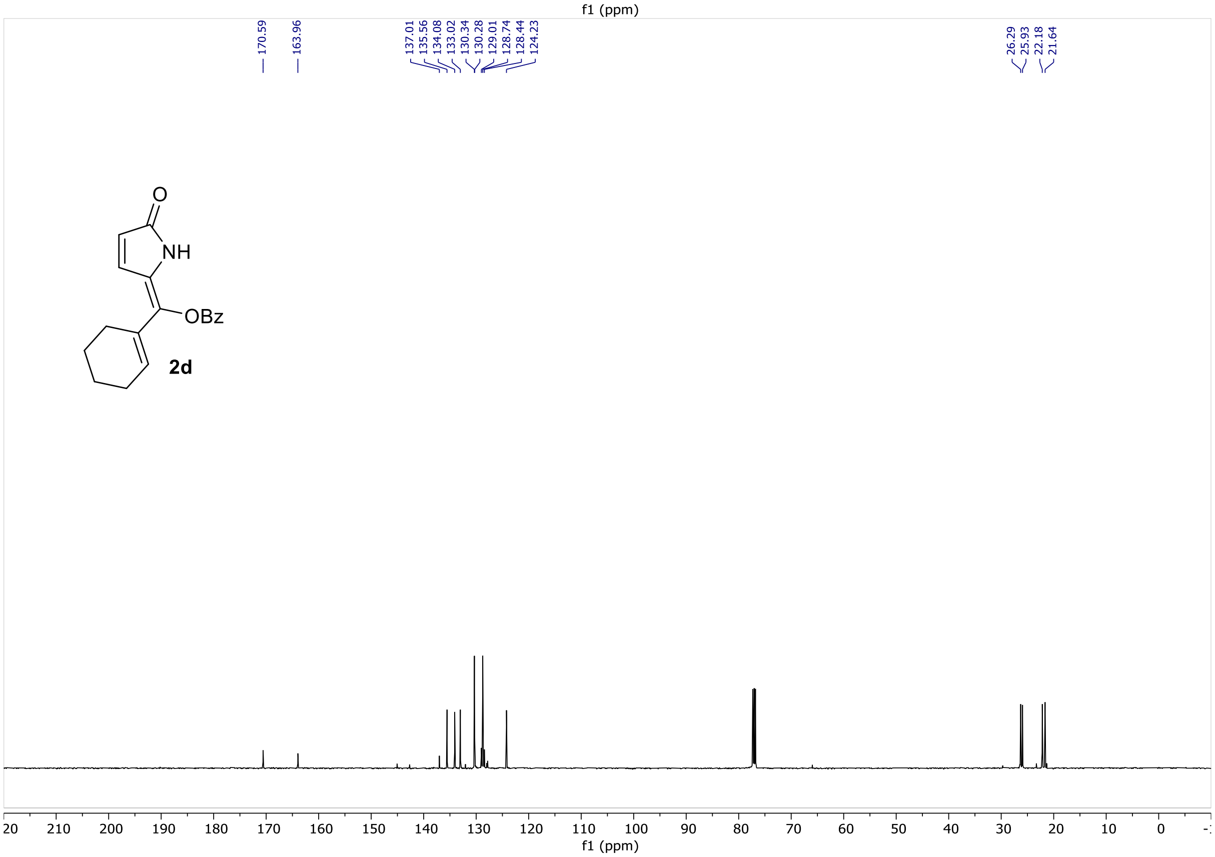
170.59
163.96
137.01
135.56
134.08
133.02
130.34
130.28
129.01
128.74
128.44
124.23
26.29
25.93
22.18
21.64
O
NH
OBz
2d
f1 (ppm)

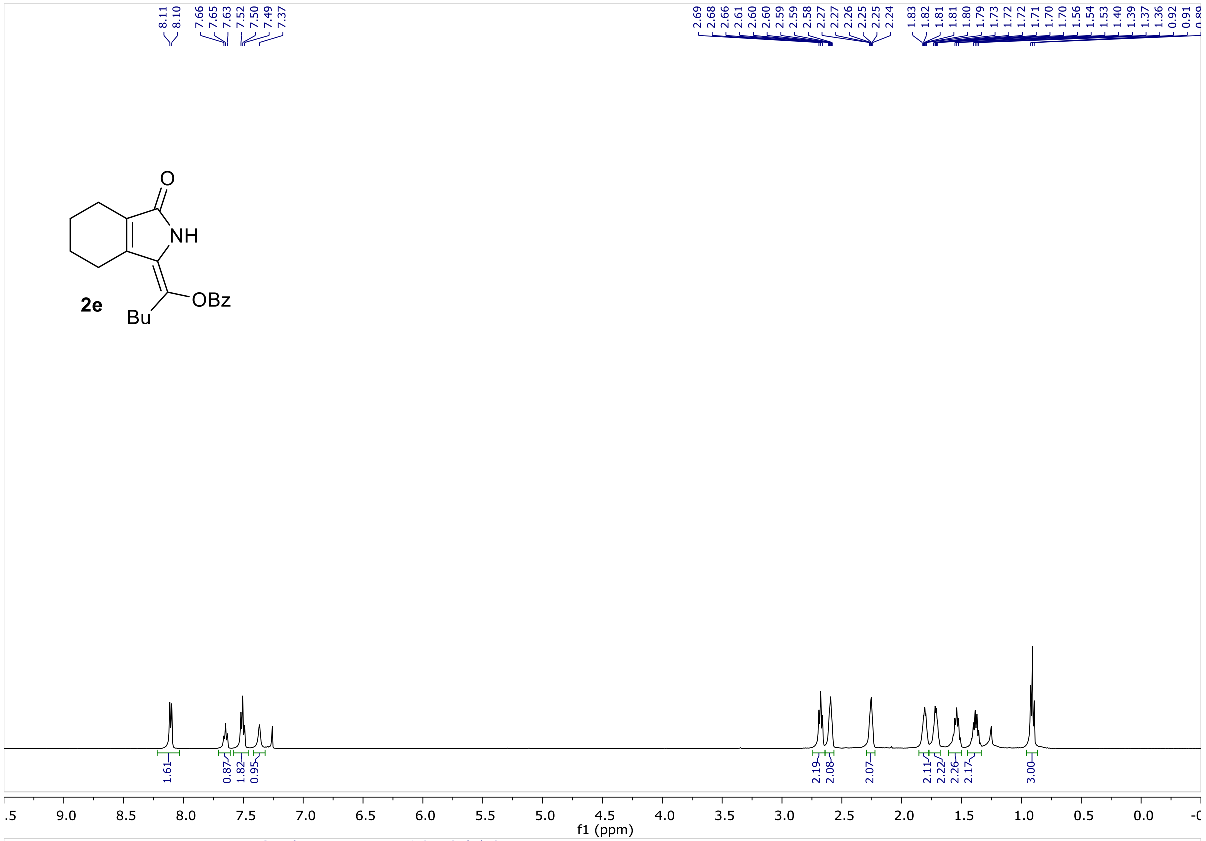

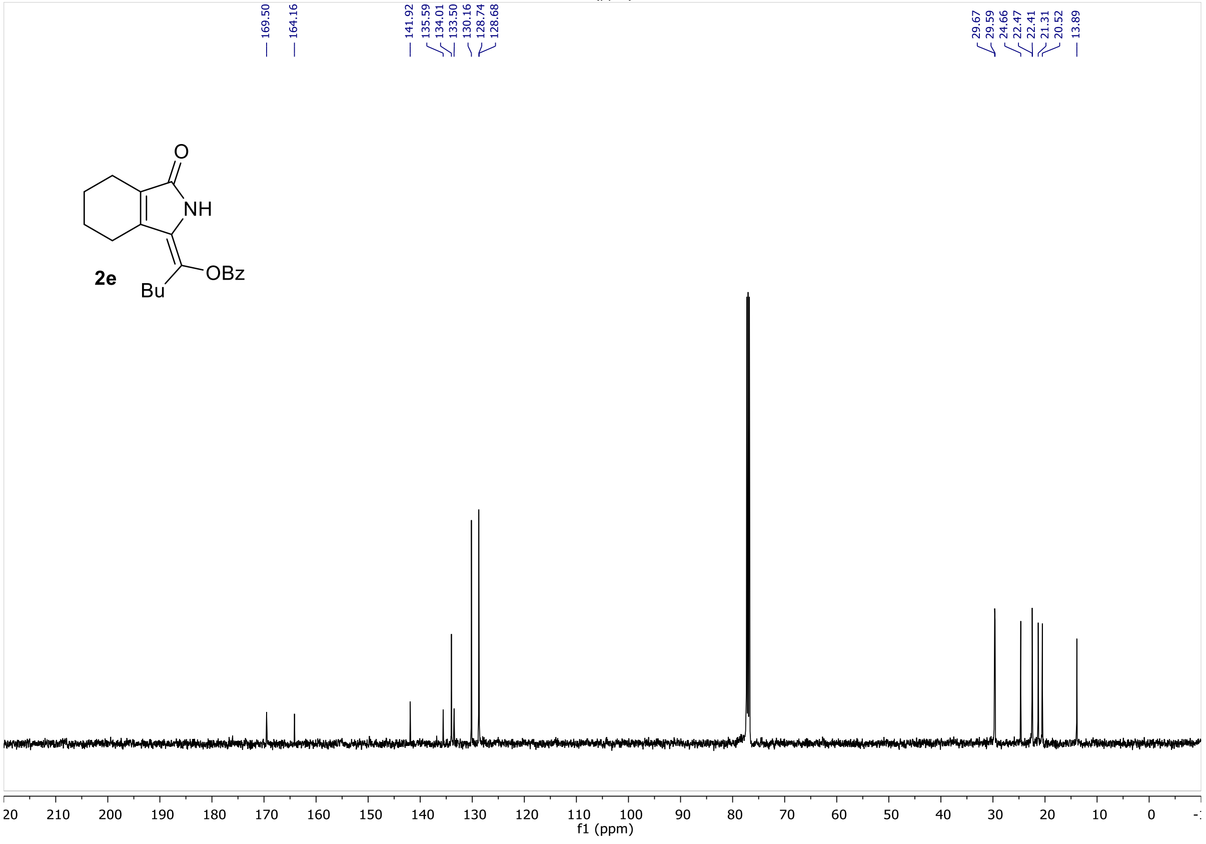

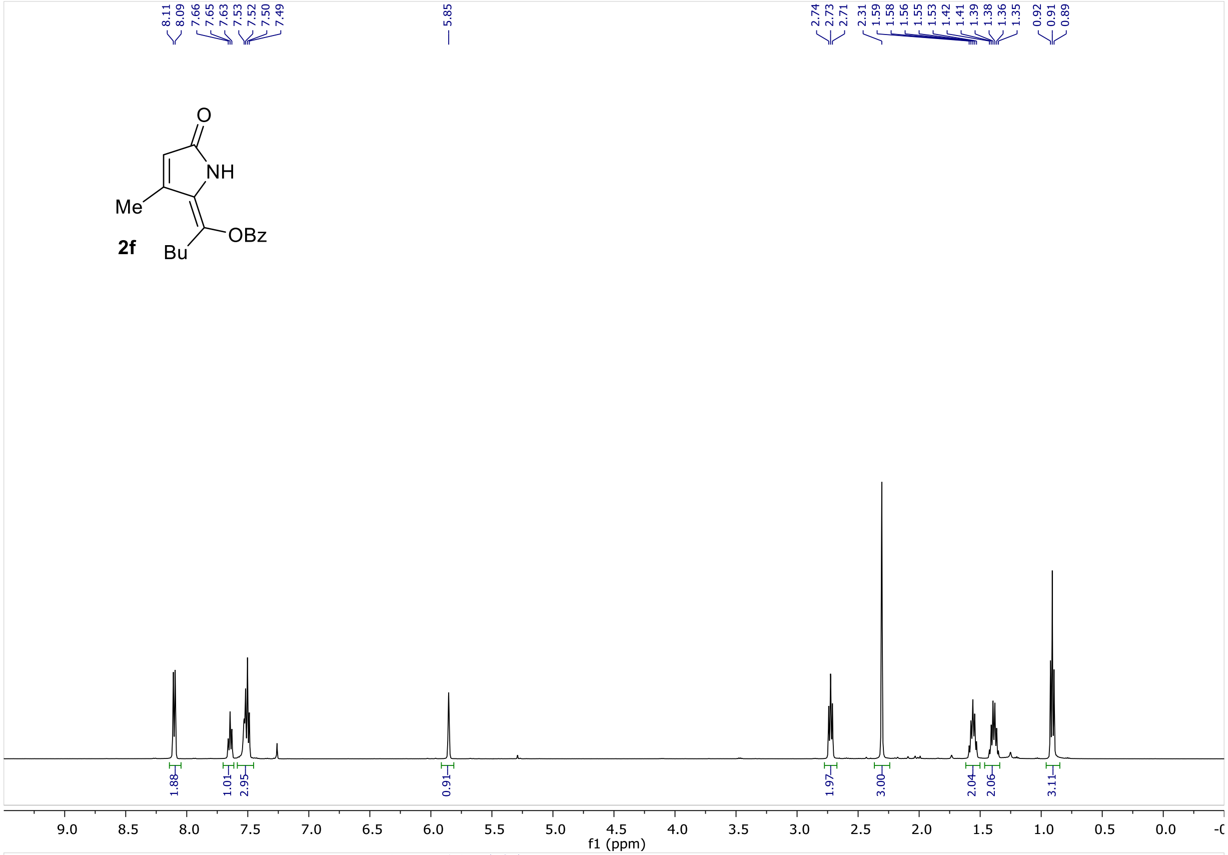
8.11
8.09
7.66
7.65
7.63
7.53
7.52
7.50
7.49
5.85
2.74
2.73
2.71
2.31
1.59
1.58
1.56
1.55
1.53
1.42
1.41
1.39
1.38
1.36
1.35
0.92
0.91
0.89
O
NH
Me
2f
Bu
OBz
1.88
1.01
2.95
0.91
1.97
3.00
2.04
2.06
3.11
f1 (ppm)

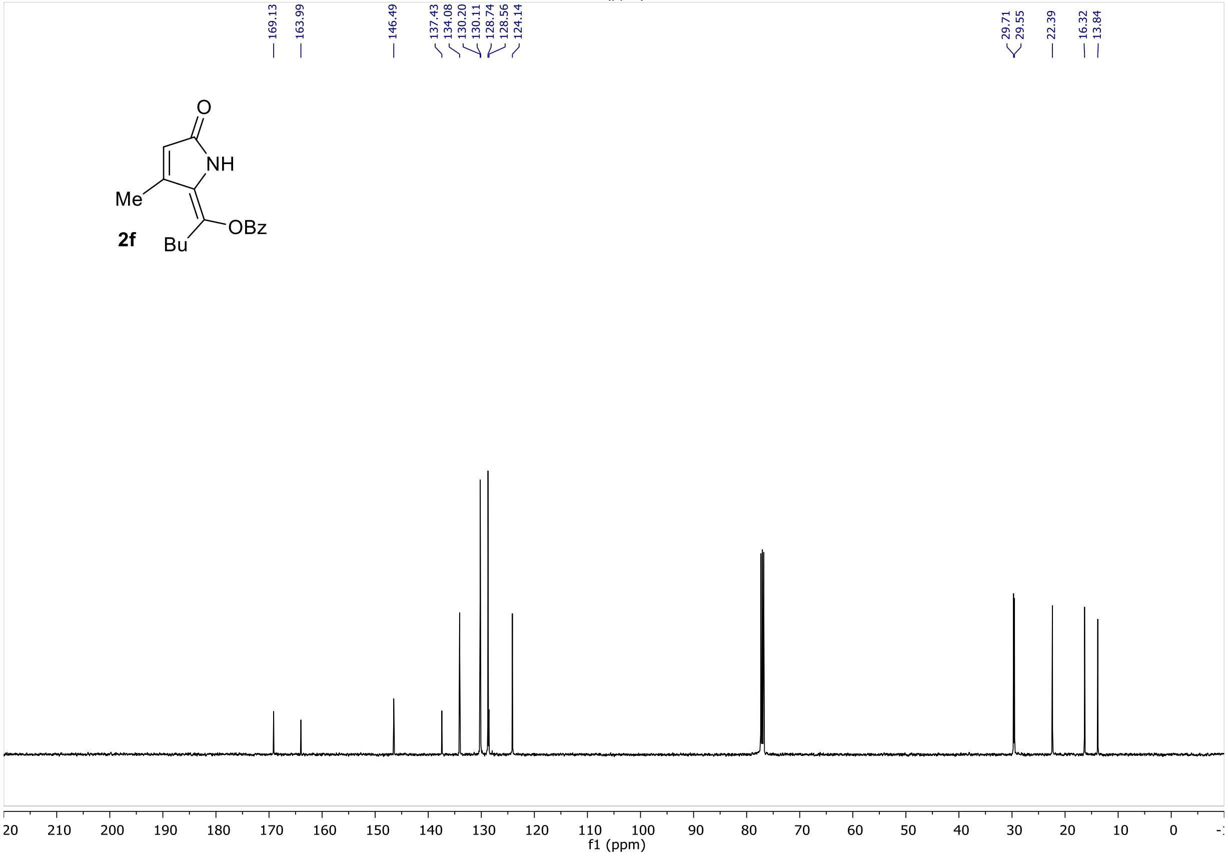
169.13
163.99
146.49
137.43
134.08
130.20
130.11
128.74
128.56
124.14
29.71
29.55
22.39
16.32
13.84
O
NH
Me
2f
Bu
OBz
f1 (ppm)

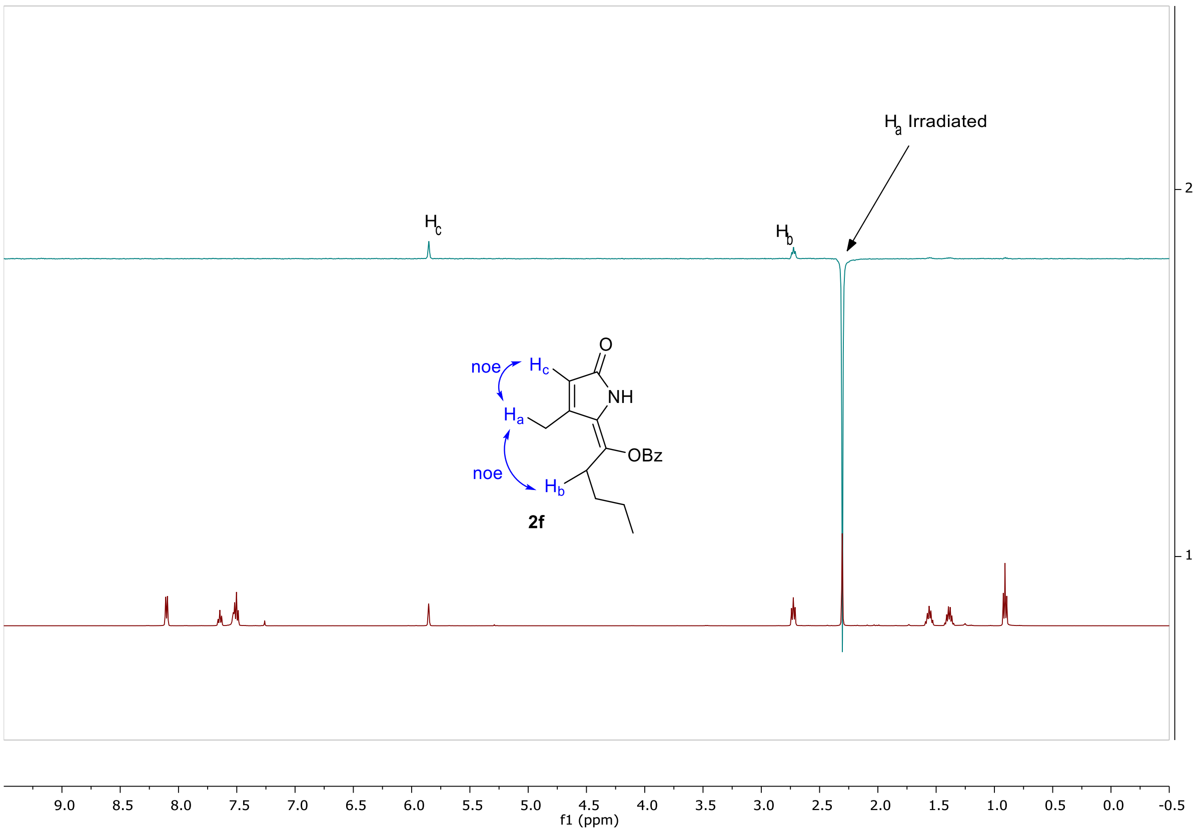

Ha Irradiated
Hc
Hb
noe
Hc
O
NH
Ha
OBz
noe
Hb
2f
2
1
9.0
8.5
8.0
7.5
7.0
6.5
6.0
5.5
5.0
4.5
4.0
3.5
3.0
2.5
2.0
1.5
1.0
0.5
0.0
-0.5
f1 (ppm)

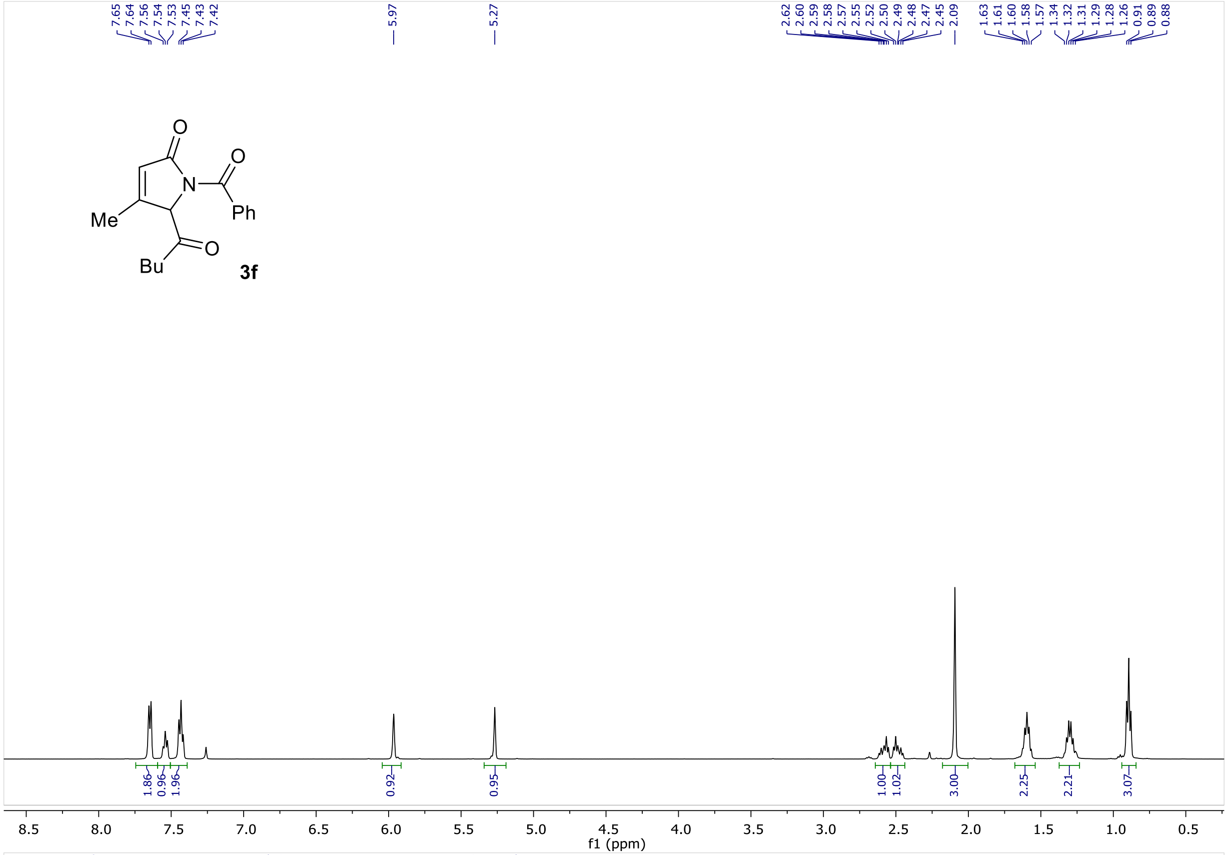
7.65
7.64
7.56
7.54
7.53
7.45
7.43
7.42
5.97
5.27
2.62
2.60
2.59
2.58
2.57
2.55
2.52
2.50
2.49
2.48
2.47
2.45
2.09
1.63
1.61
1.60
1.58
1.57
1.34
1.32
1.31
1.29
1.28
1.26
0.91
0.89
0.88
O
O
N
Ph
Me
Bu
O
3f
1.86
0.96
1.96
0.92
0.95
1.00
1.02
3.00
2.25
2.21
3.07
8.5
8.0
7.5
7.0
6.5
6.0
5.5
5.0
4.5
4.0
3.5
3.0
2.5
2.0
1.5
1.0
0.5
f1 (ppm)

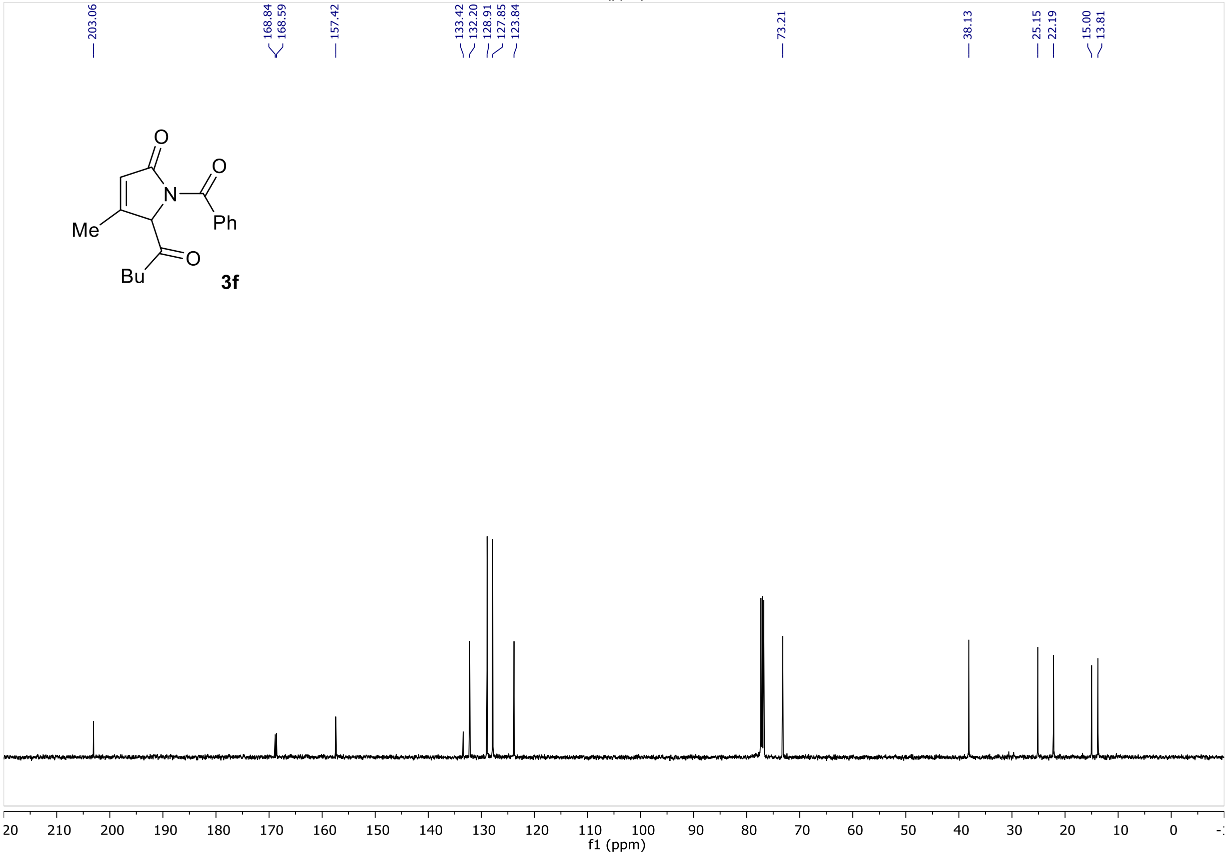
203.06
168.84
168.59
157.42
133.42
132.20
128.91
127.85
123.84
73.21
38.13
25.15
22.19
15.00
13.81
O
O
N
Ph
Me
Bu
O
3f
210
200
190
180
170
160
150
140
130
120
110
100
90
80
70
60
50
40
30
20
10
0
f1 (ppm)

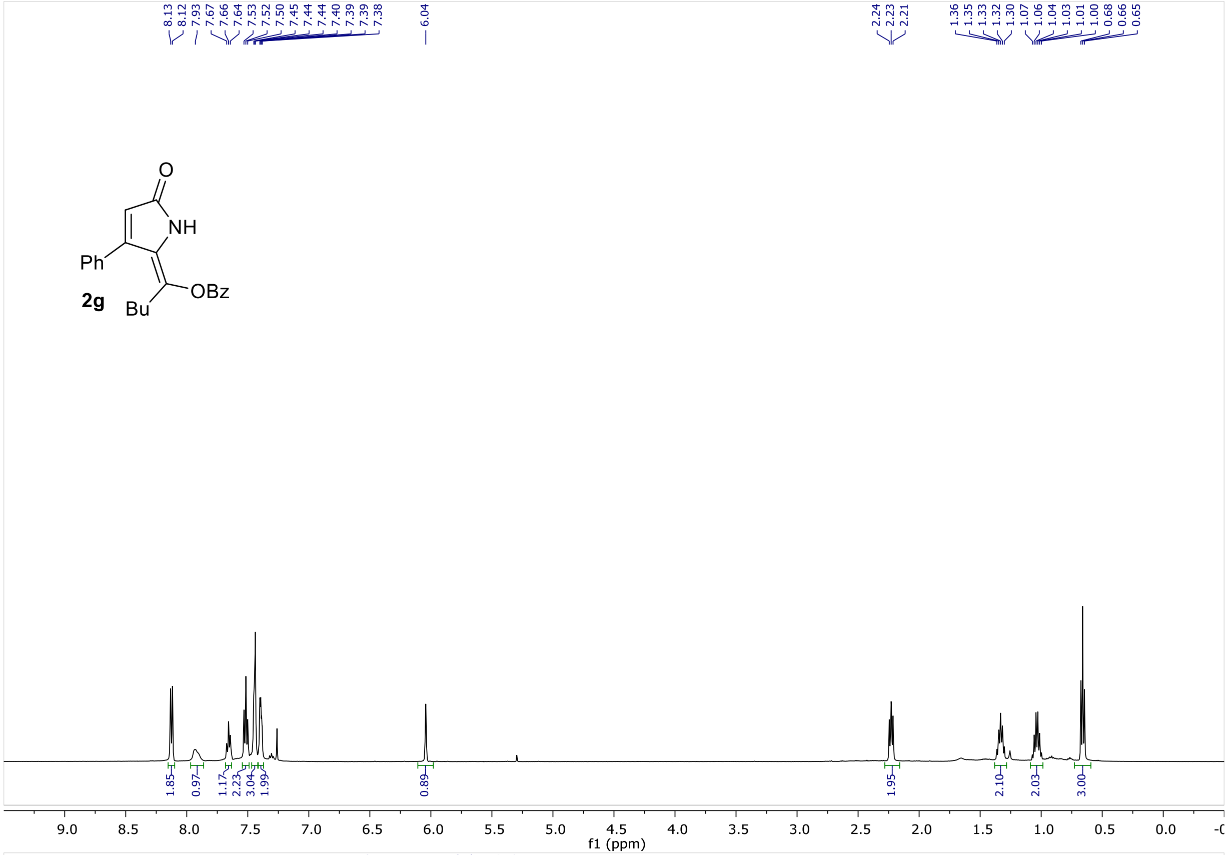
8.13
8.12
7.93
7.67
7.66
7.64
7.53
7.52
7.50
7.45
7.44
7.44
7.40
7.39
7.39
7.38
6.04
2.24
2.23
2.21
1.36
1.35
1.33
1.32
1.30
1.07
1.06
1.04
1.03
1.01
1.00
0.68
0.66
0.65
O
NH
Ph
OBz
2g
Bu
1.85
0.97
1.17
2.23
3.04
1.99
0.89
1.95
2.10
2.03
3.00
f1 (ppm)

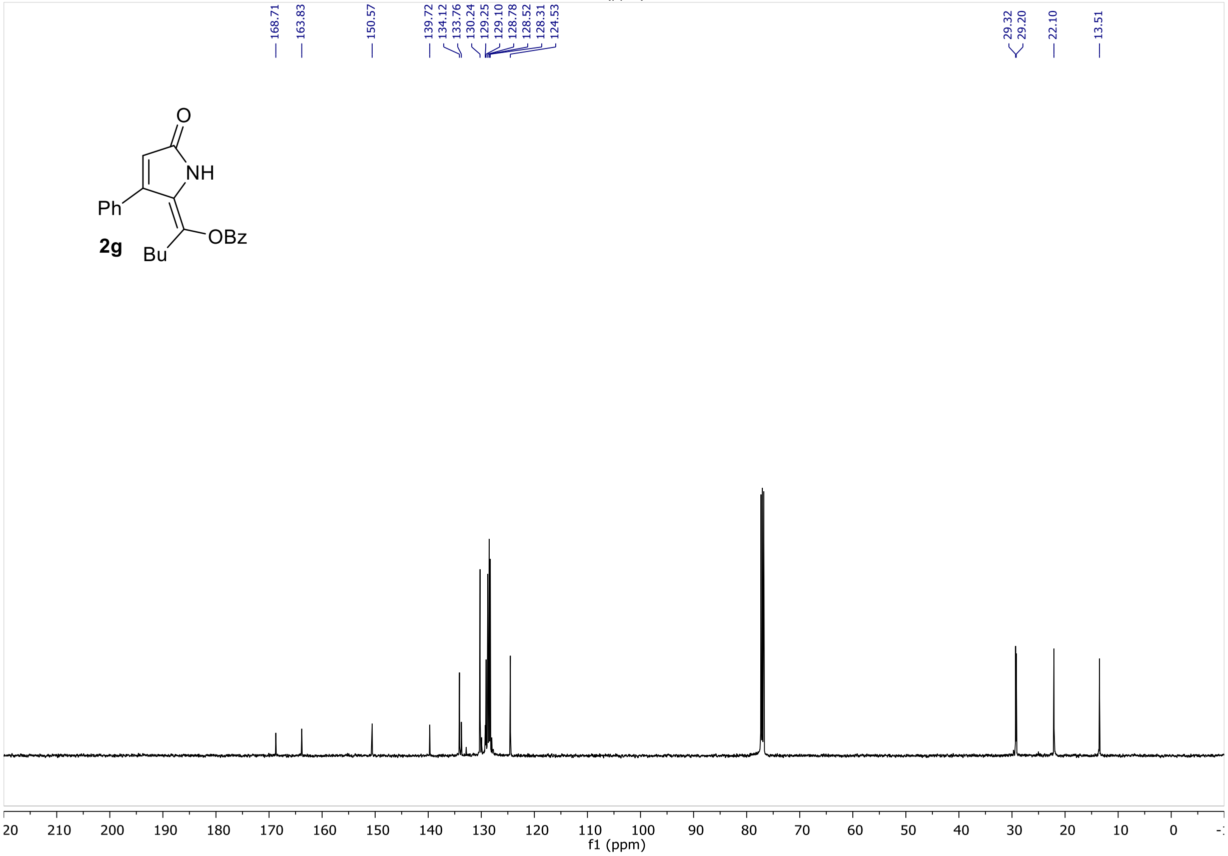
168.71
163.83
150.57
139.72
134.12
133.76
130.24
129.25
129.10
128.78
128.52
128.31
124.53
29.32
29.20
22.10
13.51
O
NH
Ph
OBz
2g
Bu
f1 (ppm)

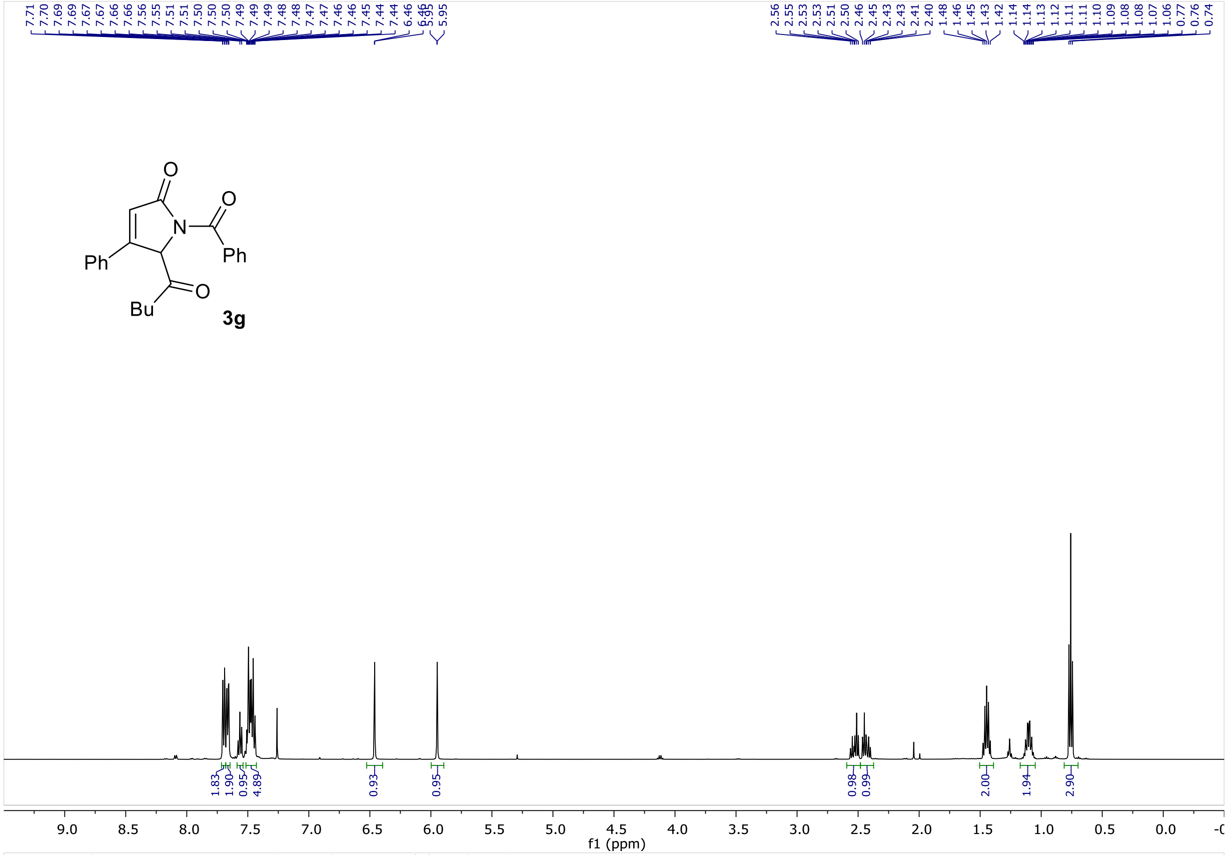

Ph
Ph
Bu
3g
f1 (ppm)

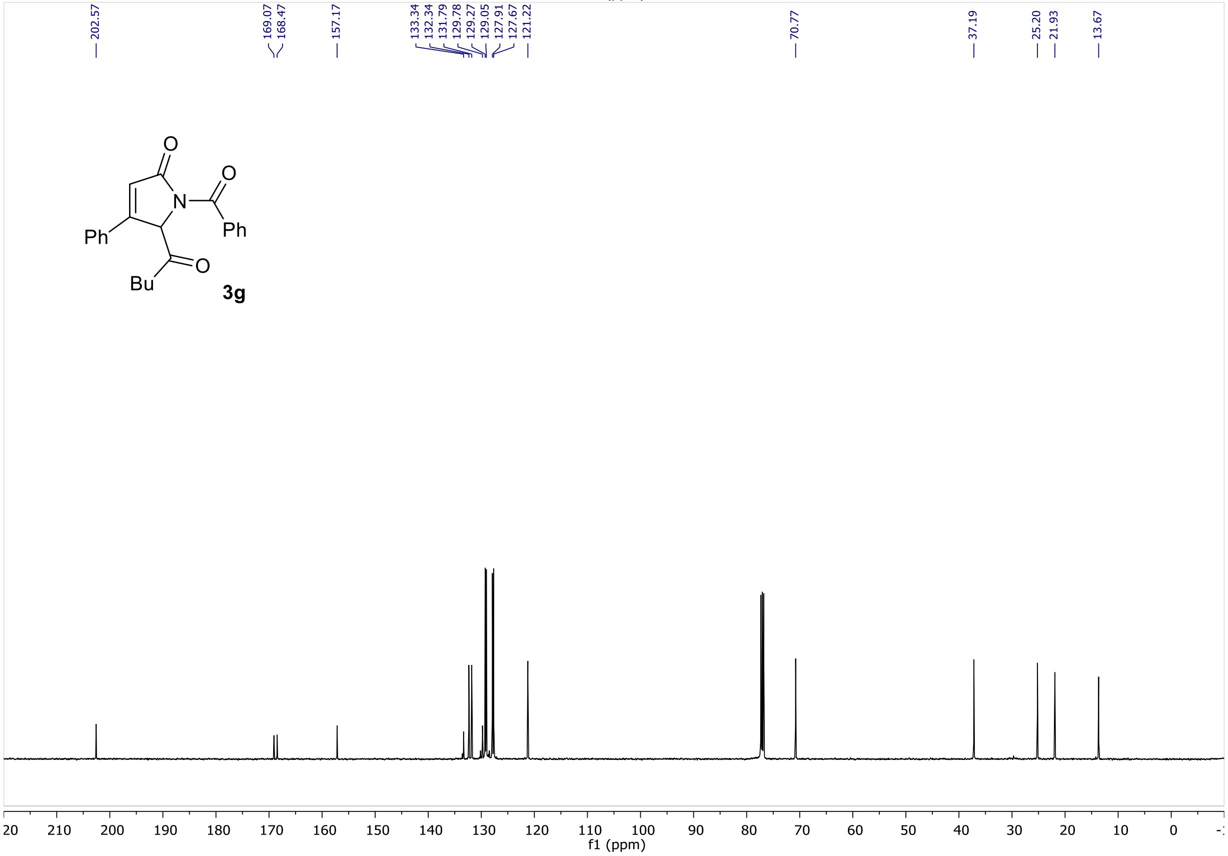

202.57
169.07
168.47
157.17
133.34
132.34
131.79
129.78
129.27
129.05
127.91
127.67
121.22
70.77
37.19
25.20
21.93
13.67
Ph
Ph
Bu
3g
f1 (ppm)

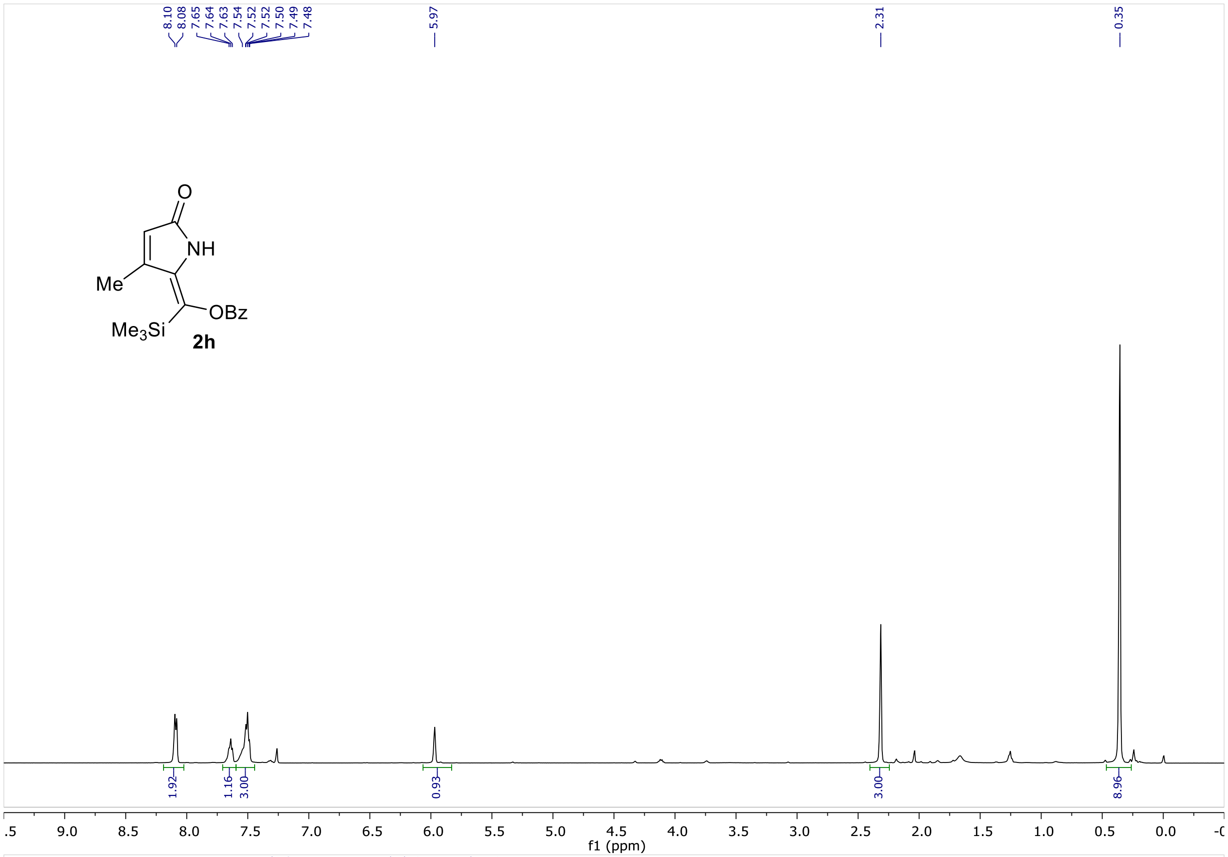
8.10
8.08
7.65
7.64
7.63
7.54
7.52
7.52
7.50
7.49
7.48
5.97
2.31
0.35
O
NH
Me
OBz
Me3Si
2h
1.92
1.16
3.00
0.93
3.00
8.96
f1 (ppm)

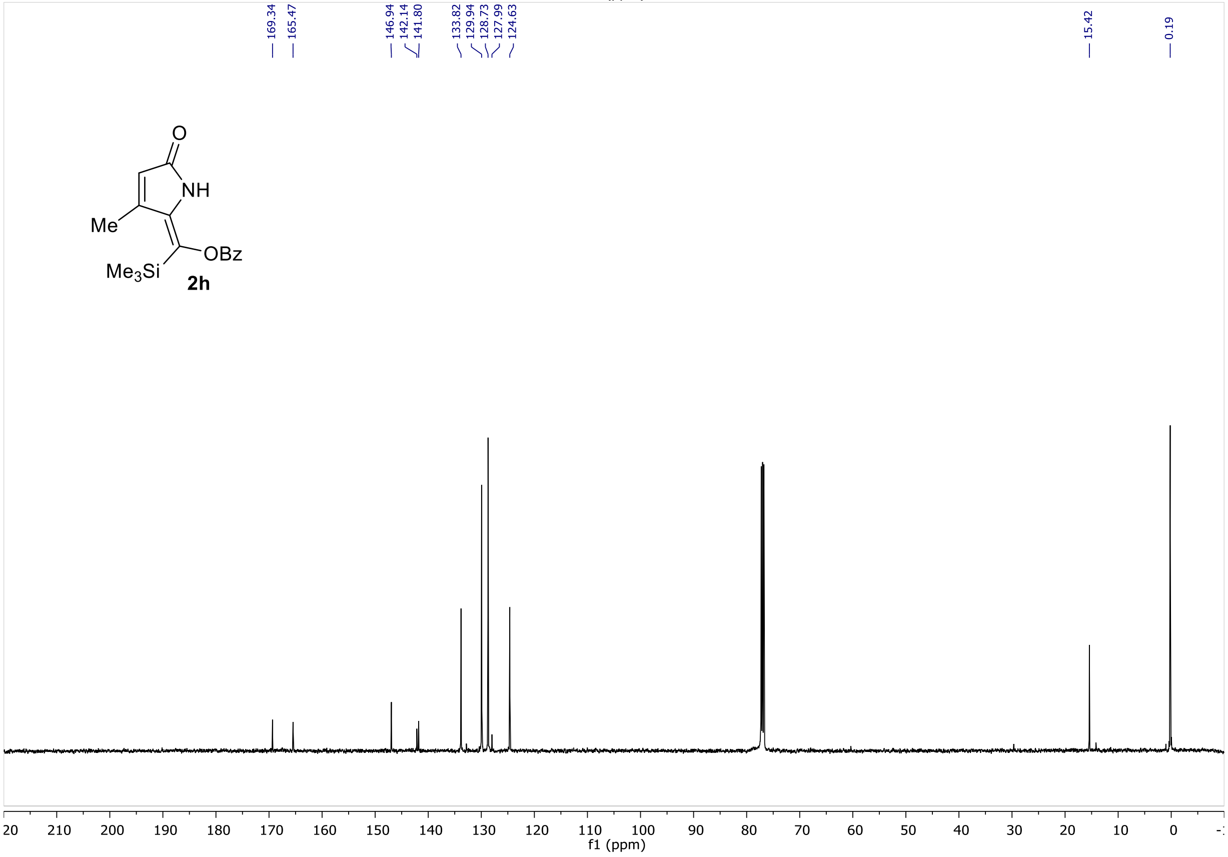
169.34
165.47
146.94
142.14
141.80
133.82
129.94
128.73
127.99
124.63
15.42
0.19
O
NH
Me
OBz
Me3Si
2h
f1 (ppm)

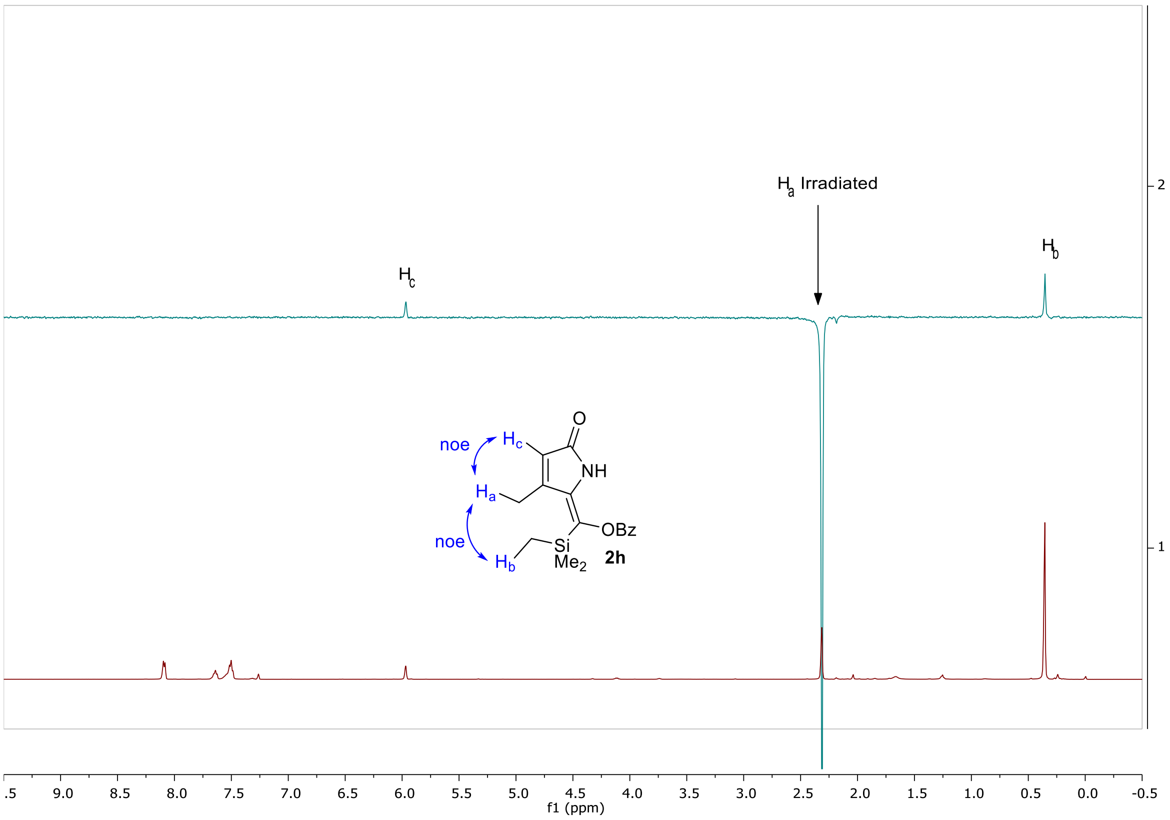

Ha Irradiated
Hc
Hb
noe
Hc
O
NH
Ha
OBz
noe
Hb
Si
Me2
2h
2
1
.5
9.0
8.5
8.0
7.5
7.0
6.5
6.0
5.5
5.0
4.5
4.0
3.5
3.0
2.5
2.0
1.5
1.0
0.5
0.0
-0.5
f1 (ppm)

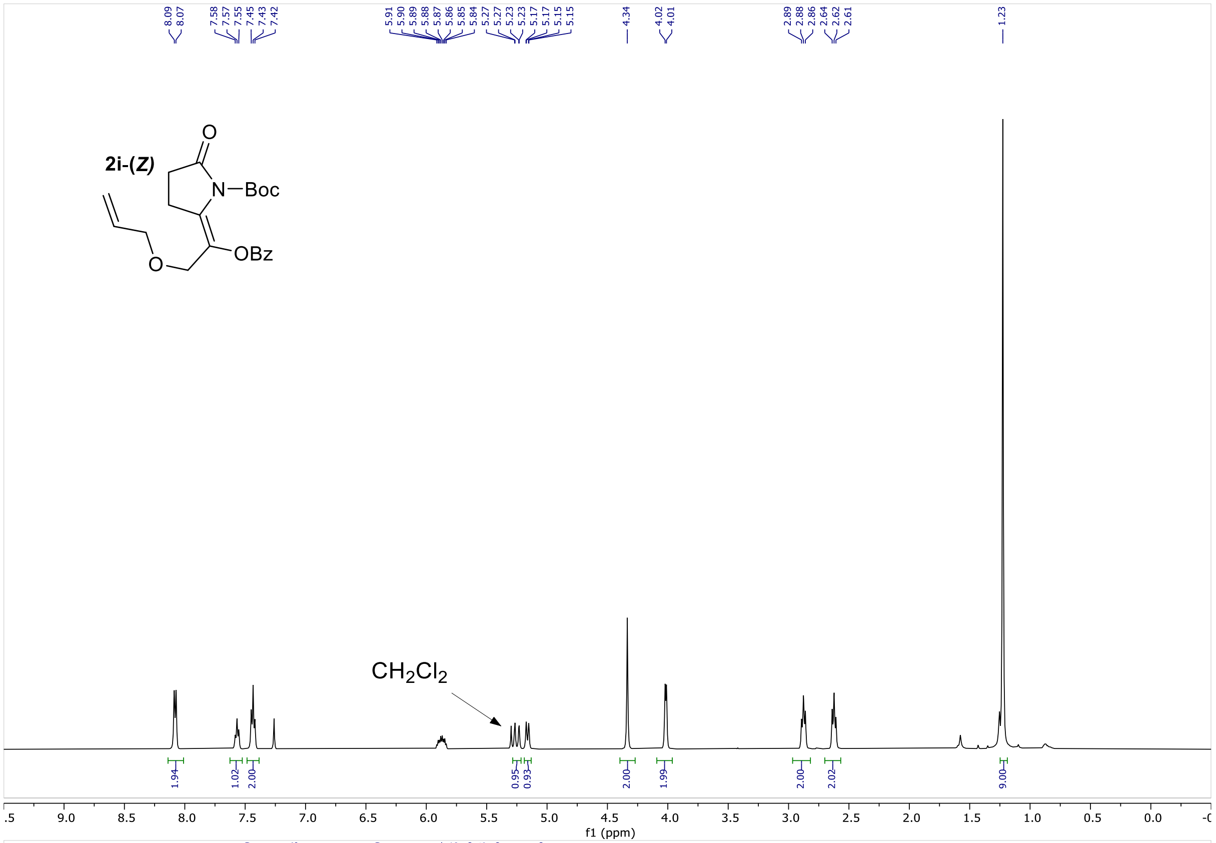
2i-(Z)
O
N−Boc
OBz
O
CH2Cl2
8.09
8.07
7.58
7.57
7.55
7.45
7.43
7.42
5.91
5.90
5.89
5.88
5.87
5.86
5.85
5.84
5.27
5.27
5.23
5.23
5.17
5.17
5.15
5.15
4.34
4.02
4.01
2.89
2.88
2.86
2.64
2.62
2.61
1.23
1.94
1.02
2.00
0.95
0.93
2.00
1.99
2.00
2.02
9.00
f1 (ppm)

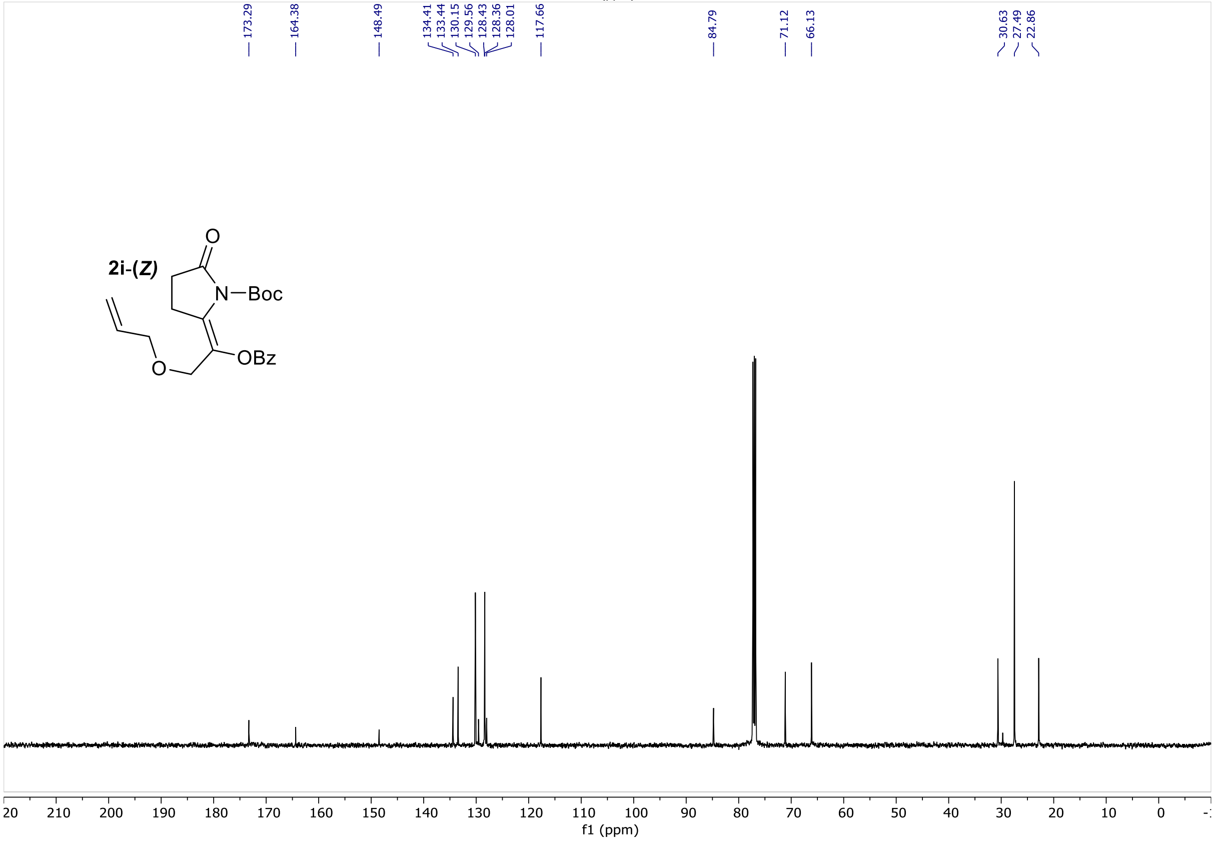
2i-(Z)
O
N−Boc
OBz
O
173.29
164.38
148.49
134.41
133.44
130.15
129.56
128.43
128.36
128.01
117.66
84.79
71.12
66.13
30.63
27.49
22.86
f1 (ppm)

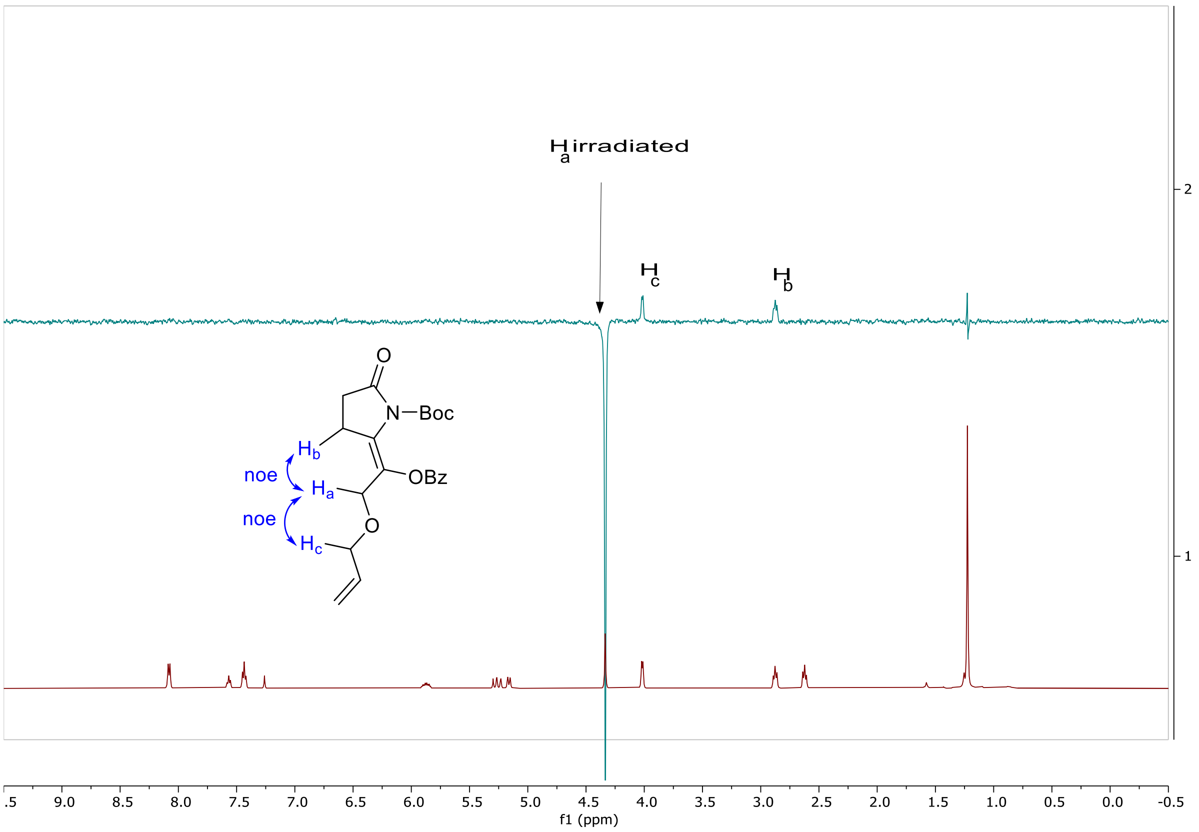

H a irradiated
H c
H b
O
N—Boc
OBz
O
noe
noe
H b
H a
H c
2
1
.5 9.0 8.5 8.0 7.5 7.0 6.5 6.0 5.5 5.0 4.5 4.0 3.5 3.0 2.5 2.0 1.5 1.0 0.5 0.0 -0.5
f1 (ppm)

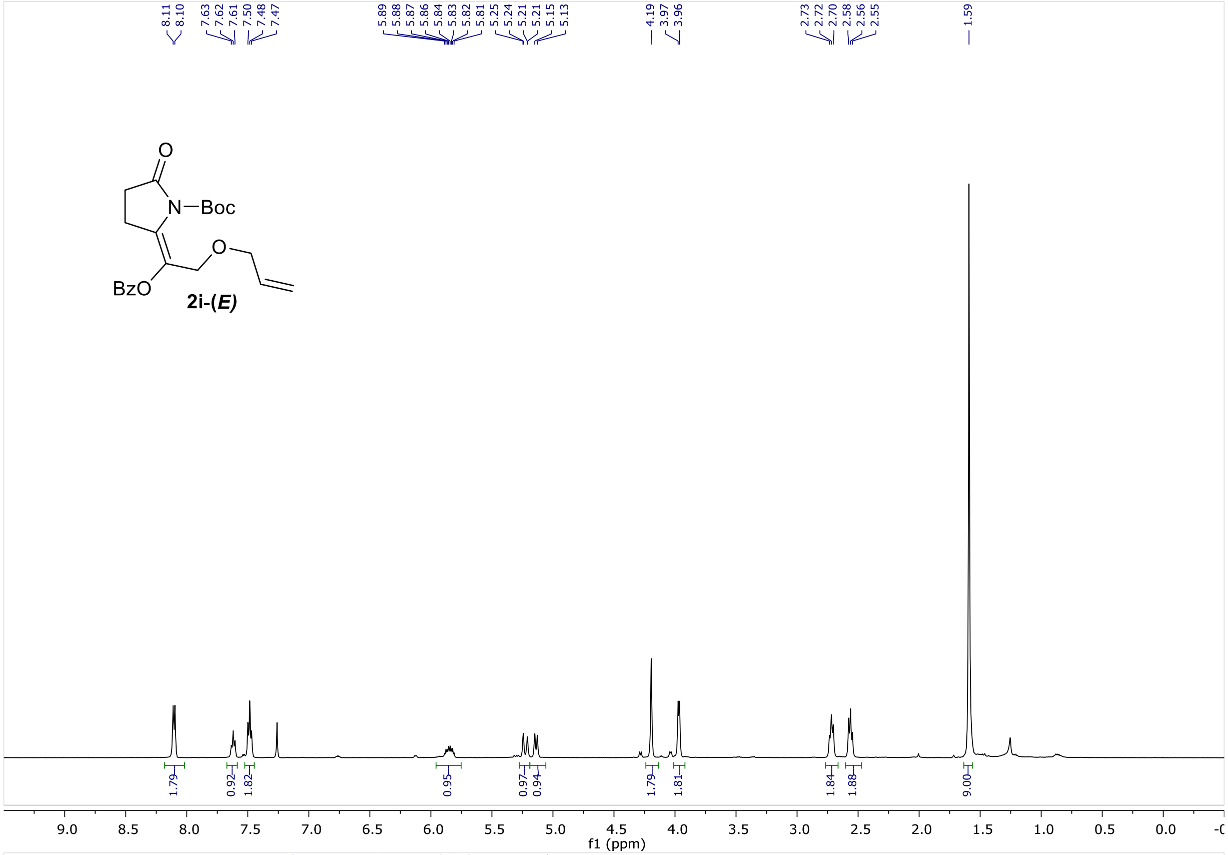
8.11
8.10
7.63
7.62
7.61
7.50
7.48
7.47
5.89
5.88
5.87
5.86
5.84
5.83
5.82
5.81
5.25
5.24
5.21
5.21
5.15
5.13
4.19
3.97
3.96
2.73
2.72
2.70
2.58
2.56
2.55
1.59
O
N−Boc
O
BzO
2i-(E)
1.79
0.92
1.82
0.95
0.97
0.94
1.79
1.81
1.84
1.88
9.00
f1 (ppm)

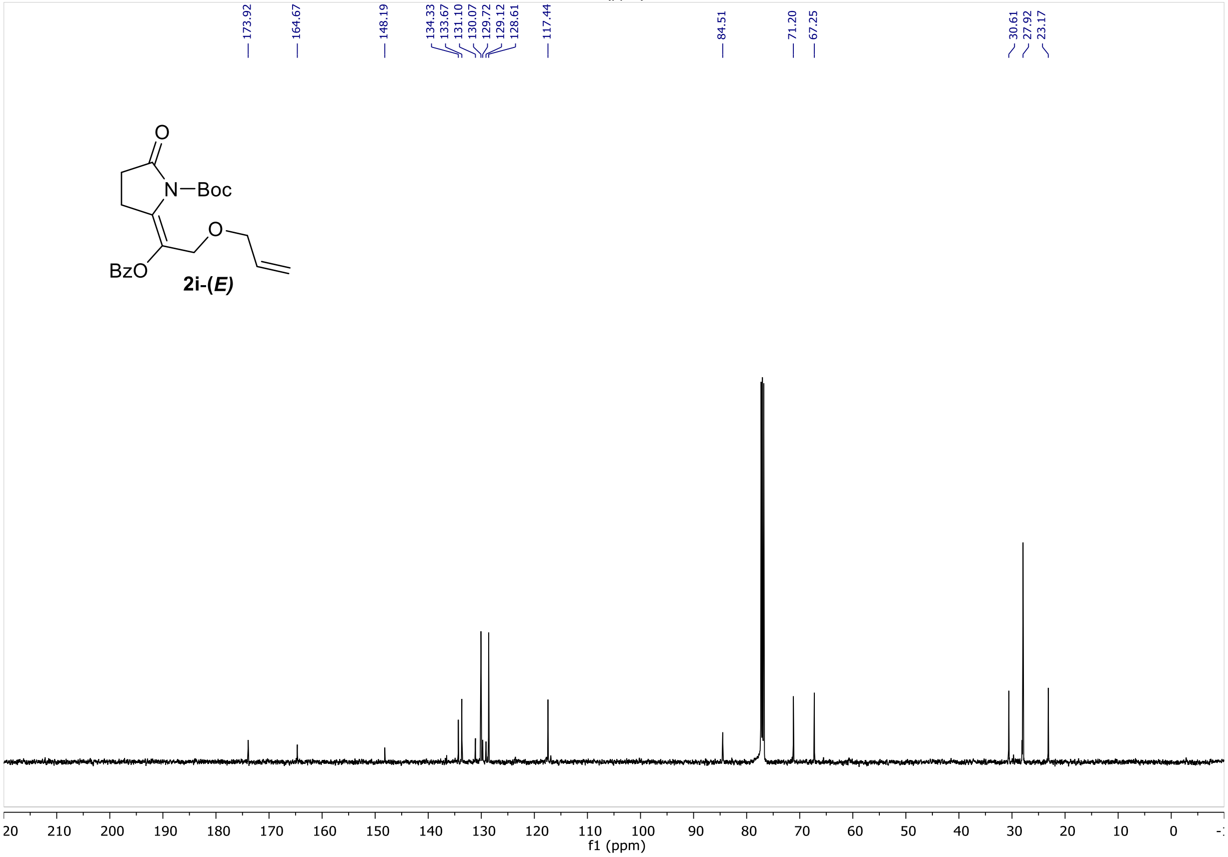
173.92
164.67
148.19
134.33
133.67
131.10
130.07
129.72
129.12
128.61
117.44
84.51
71.20
67.25
30.61
27.92
23.17
O
N−Boc
O
BzO
2i-(E)
f1 (ppm)

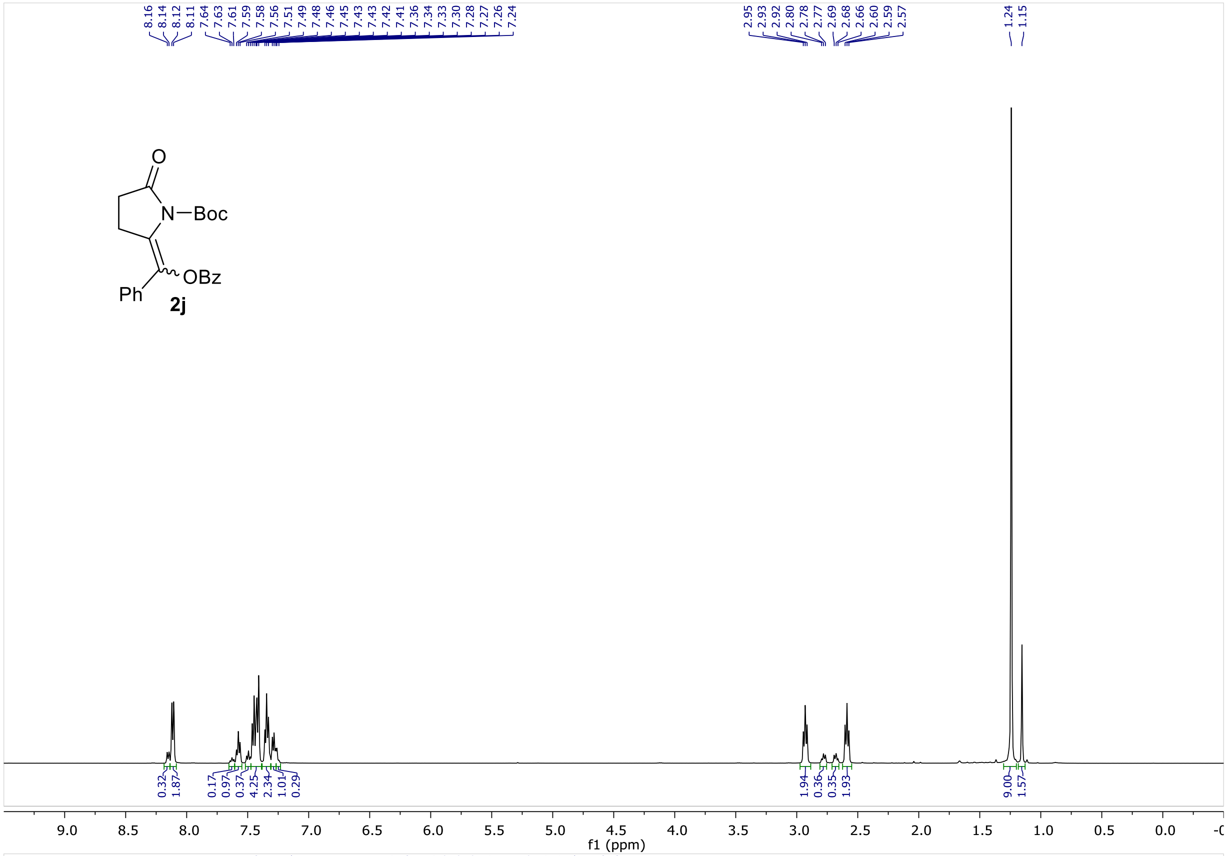

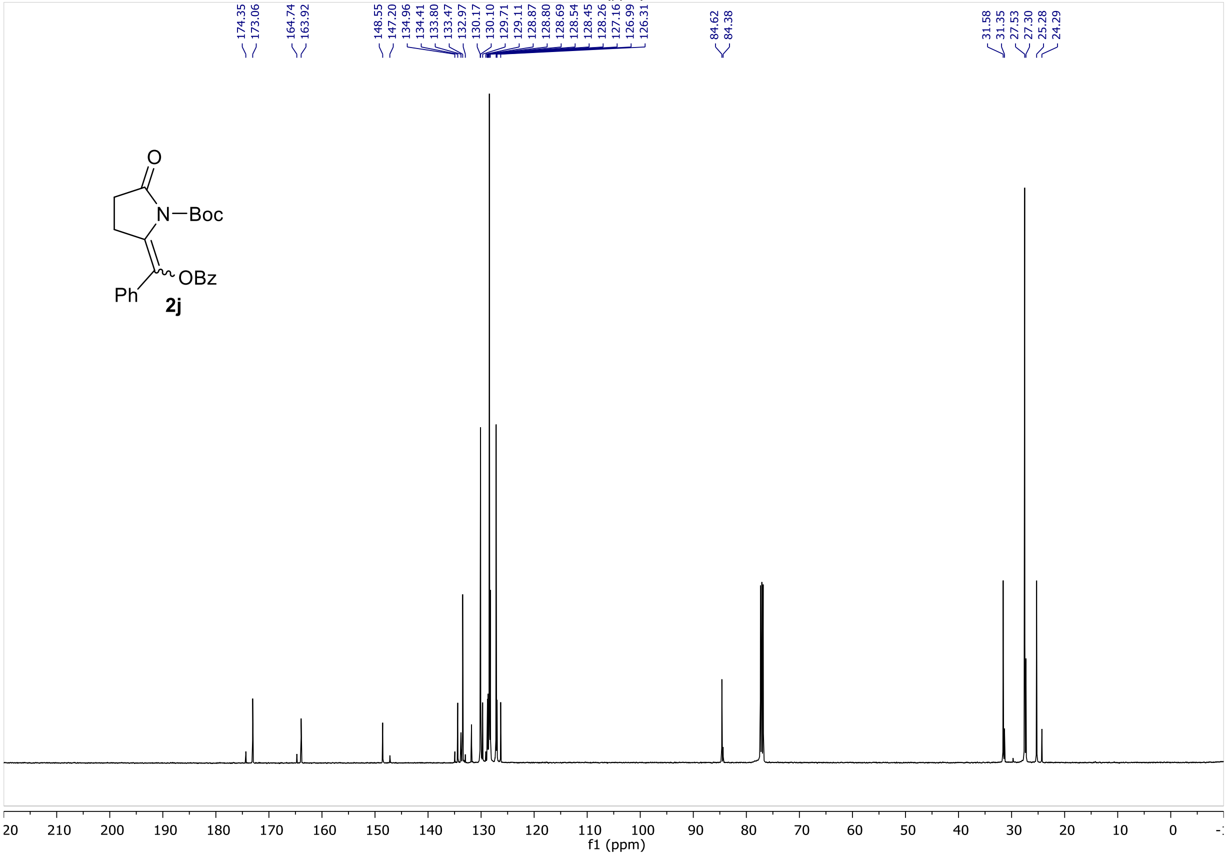

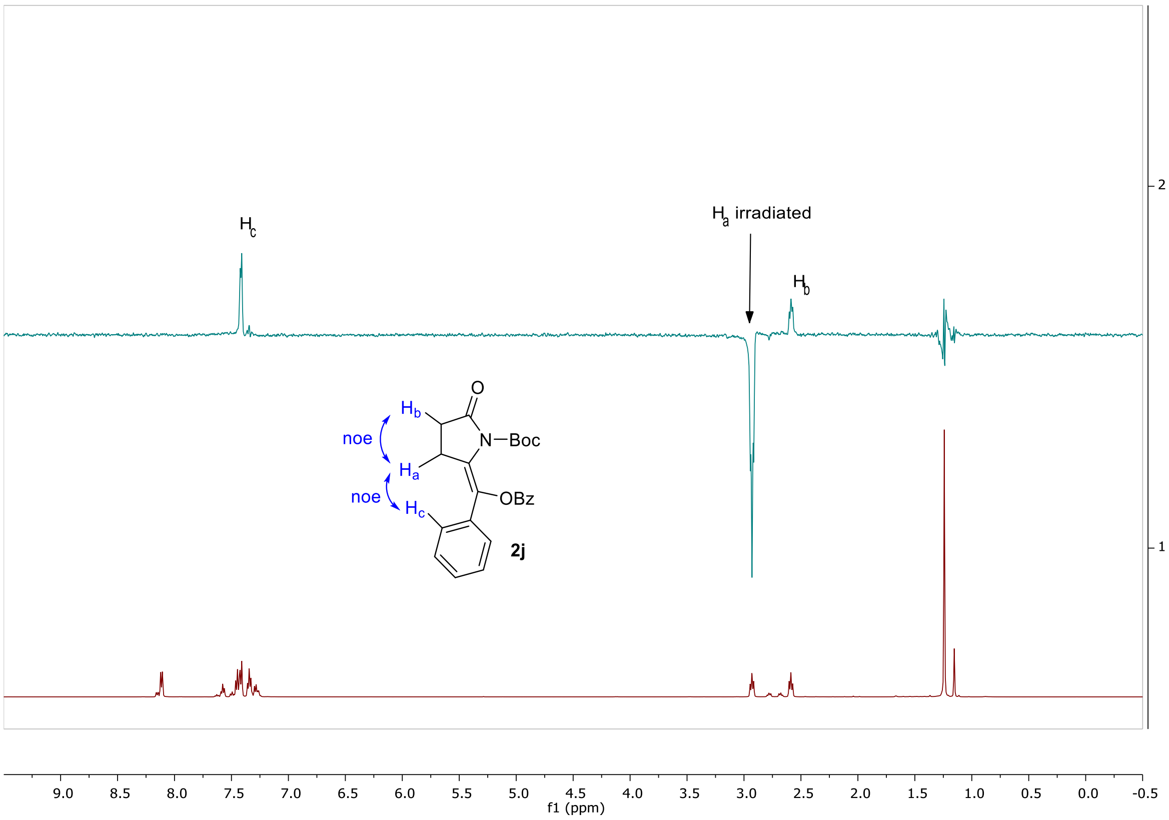

H$_c$
H$_a$ irradiated
H$_b$
noe
noe
H$_b$
H$_a$
H$_c$
O
N—Boc
OBz
2j
2
1
9.0
8.5
8.0
7.5
7.0
6.5
6.0
5.5
5.0
4.5
4.0
3.5
3.0
2.5
2.0
1.5
1.0
0.5
0.0
-0.5
f1 (ppm)

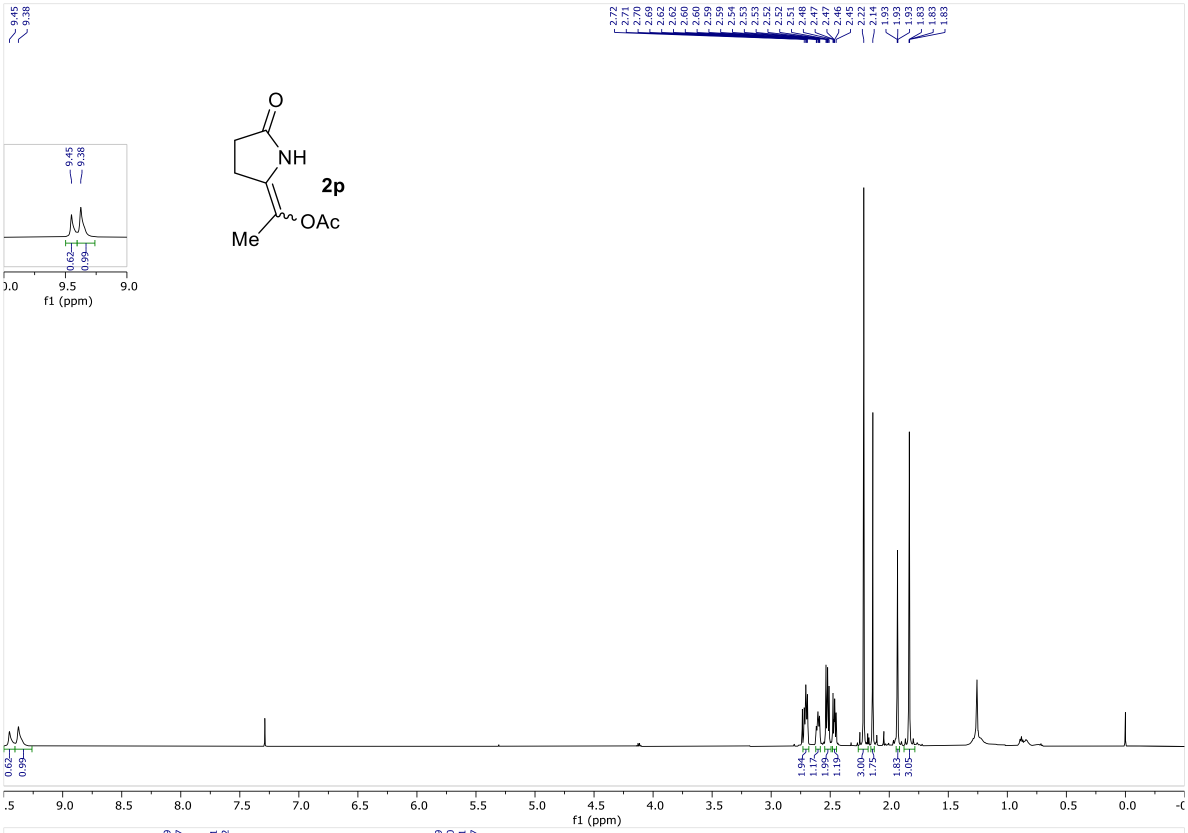
9.45
9.38
2.72
2.71
2.70
2.69
2.62
2.62
2.60
2.60
2.59
2.59
2.54
2.53
2.53
2.52
2.52
2.51
2.48
2.47
2.47
2.46
2.45
2.22
2.14
1.93
1.93
1.93
1.83
1.83
1.83
O
NH
2p
Me
OAc
0.62
0.99
1.94
1.17
1.99
1.19
3.00
1.75
1.83
3.05
f1 (ppm)

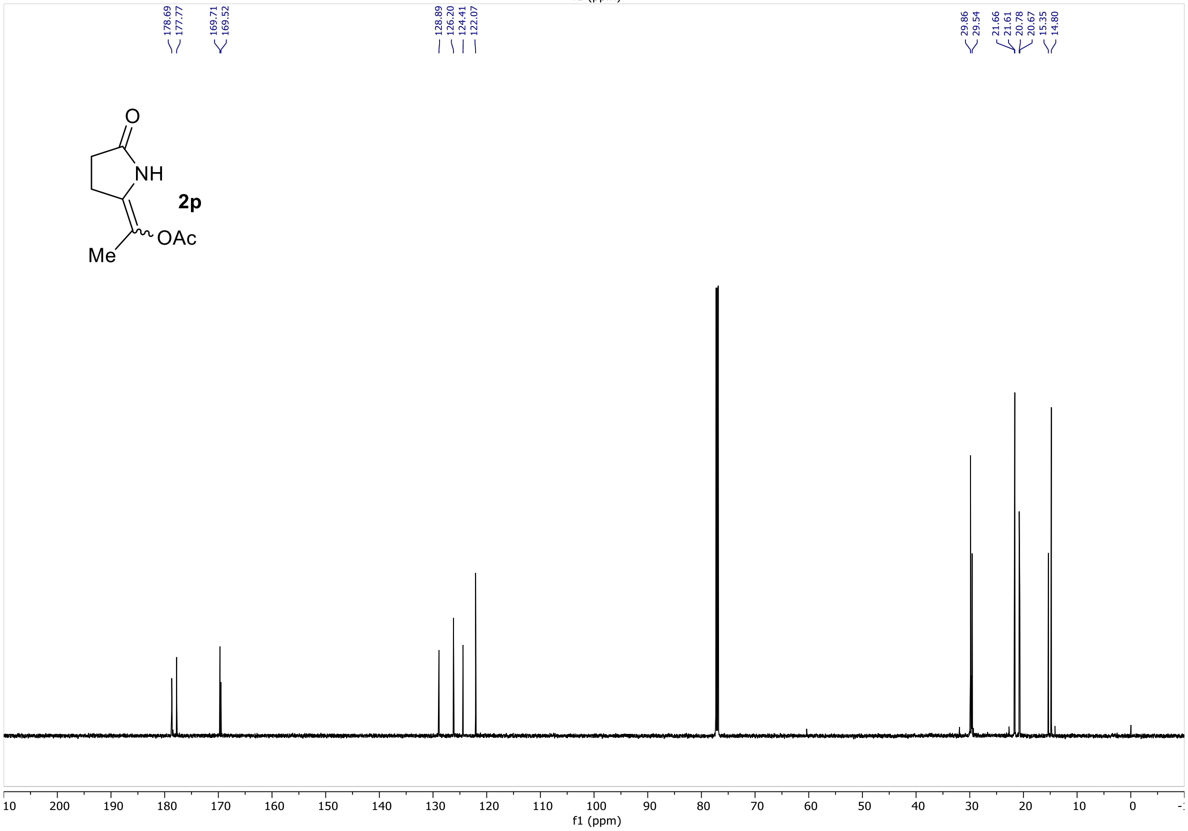
178.69
177.77
169.71
169.52
128.89
126.20
124.41
122.07
29.86
29.54
21.66
21.61
20.78
20.67
15.35
14.80
O
NH
2p
Me
OAc
f1 (ppm)

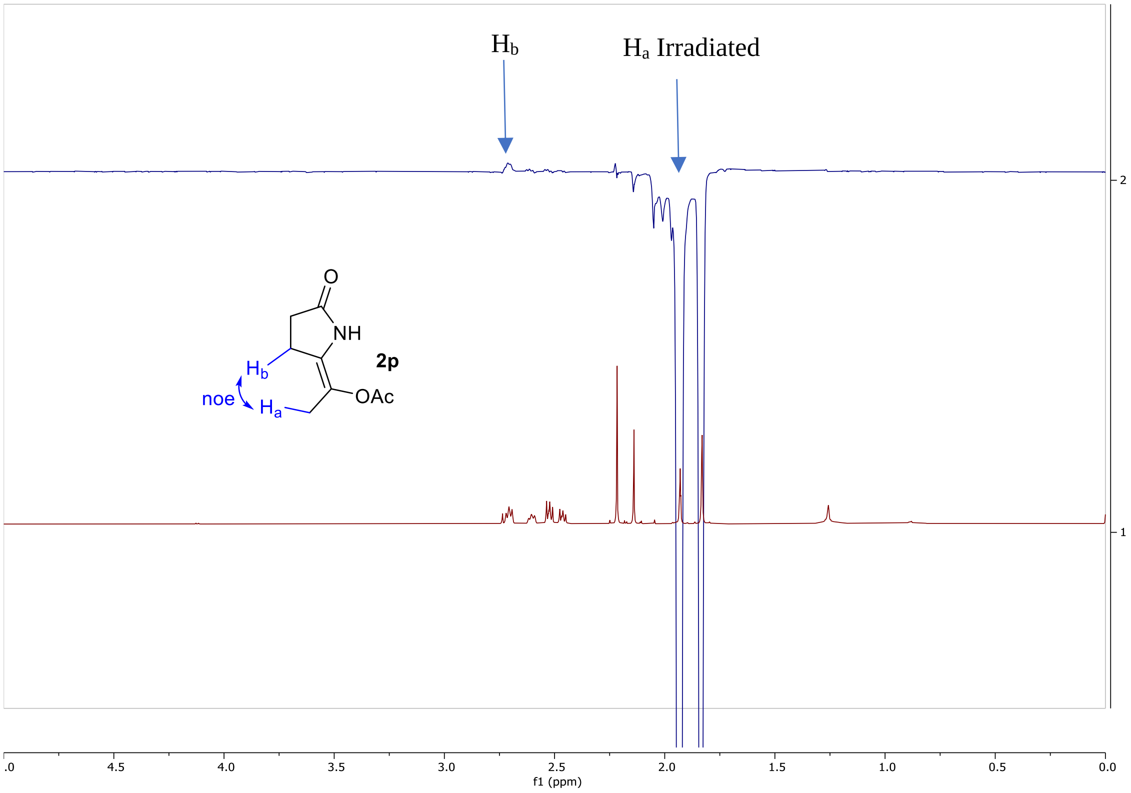
H_b
H_a Irradiated
O
NH
2p
H_b
noe
H_a
OAc
.0
4.5
4.0
3.5
3.0
2.5
2.0
1.5
1.0
0.5
0.0
f1 (ppm)
2
1

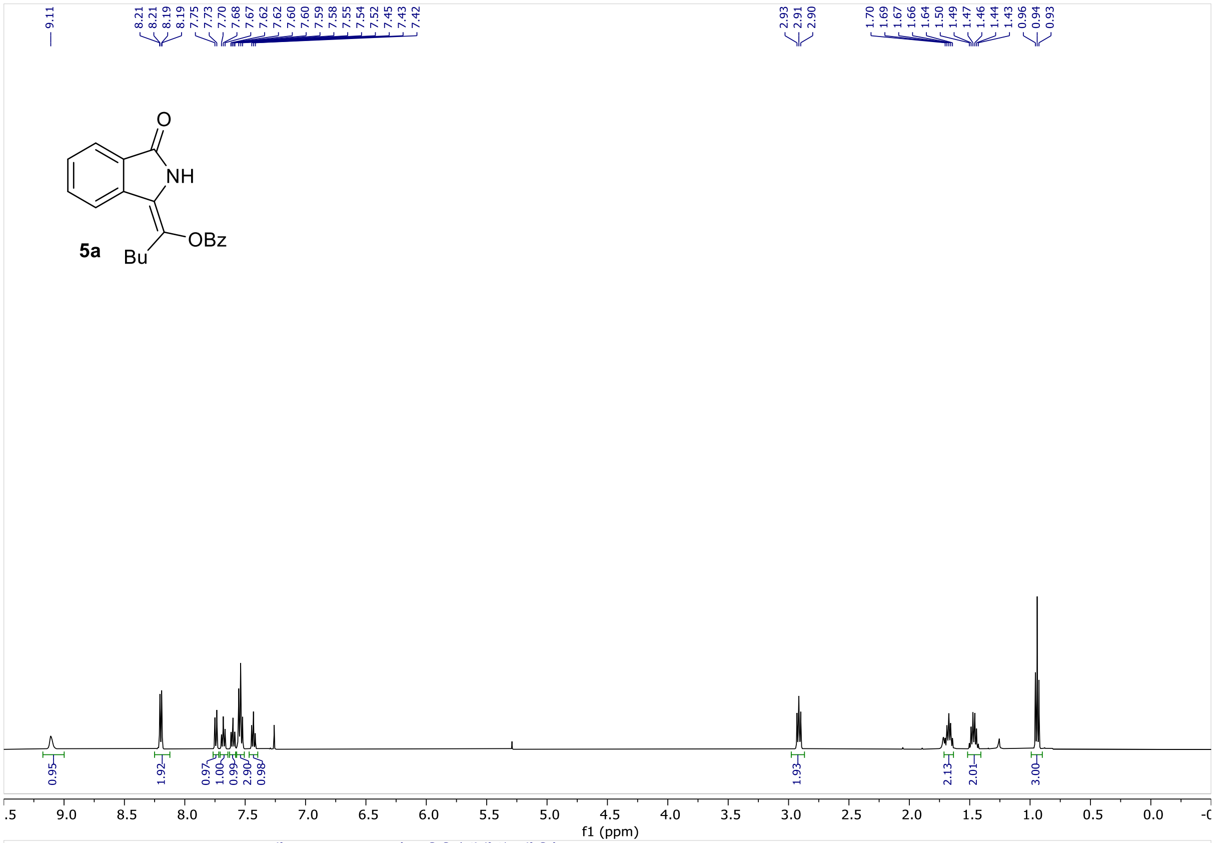

5a
9.11
8.21
8.21
8.19
8.19
7.75
7.73
7.70
7.68
7.67
7.62
7.62
7.60
7.60
7.59
7.58
7.55
7.54
7.52
7.45
7.43
7.42
2.93
2.91
2.90
1.70
1.69
1.67
1.66
1.64
1.50
1.49
1.47
1.46
1.44
1.43
0.96
0.94
0.93
0.95
1.92
0.97
1.00
0.99
2.90
0.98
1.93
2.13
2.01
3.00
f1 (ppm)

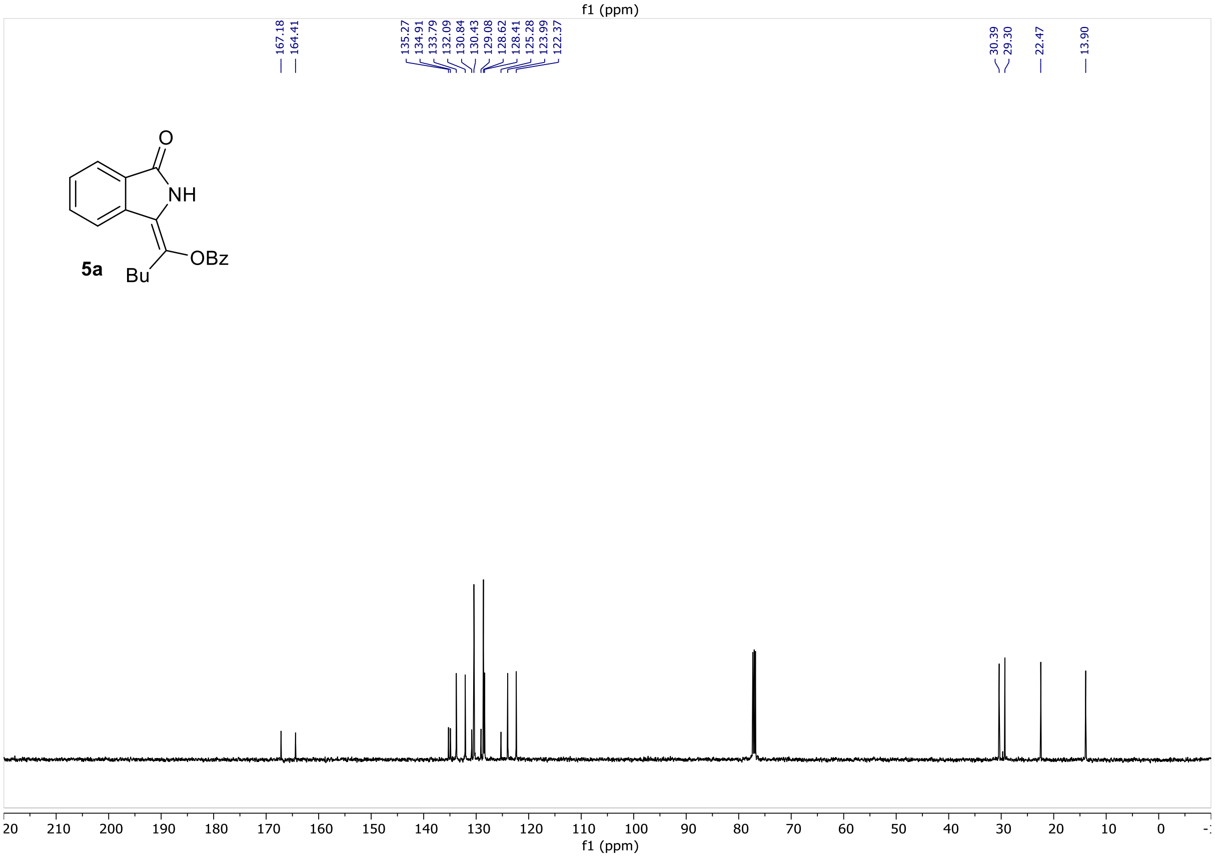

5a
167.18
164.41
135.27
134.91
133.79
132.09
130.84
130.43
129.08
128.62
128.41
125.28
123.99
122.37
30.39
29.30
22.47
13.90
f1 (ppm)

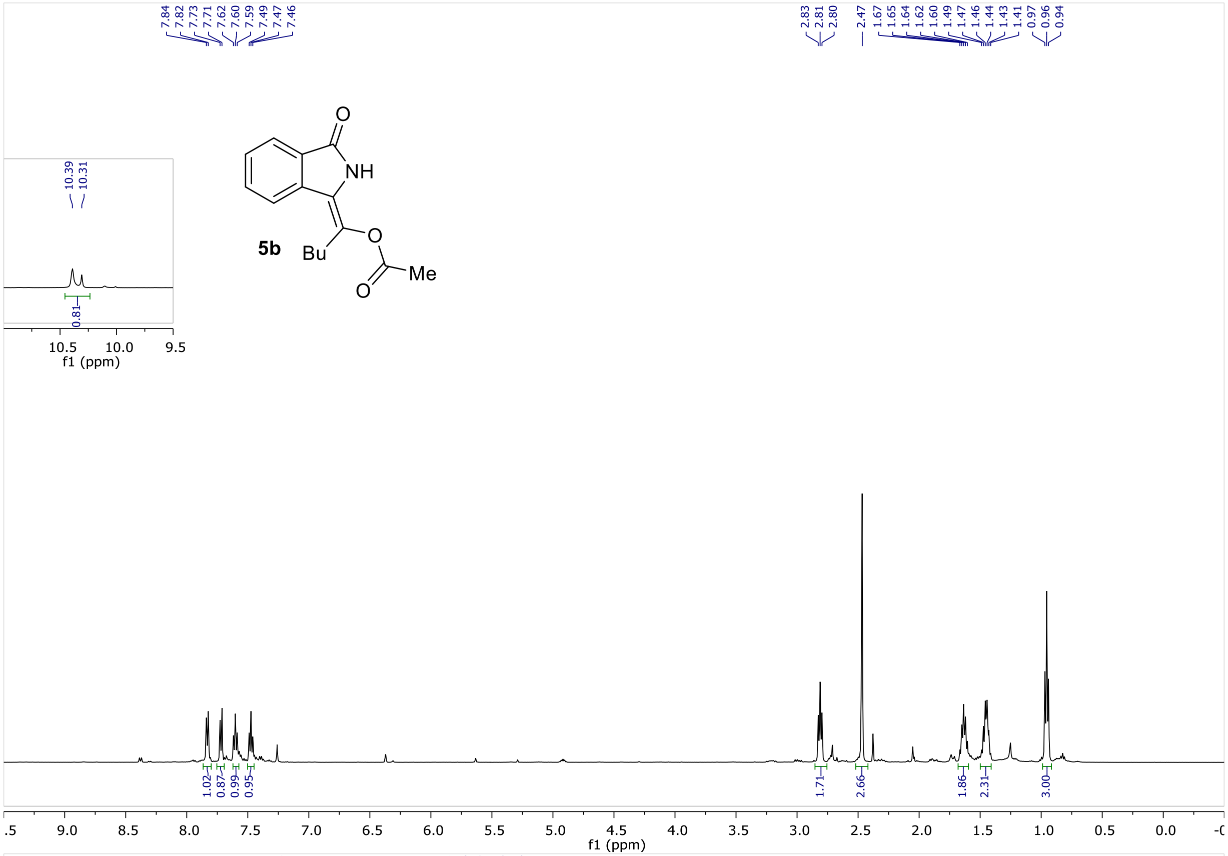

7.84 7.82 7.73 7.71 7.62 7.60 7.59 7.49 7.47 7.46
2.83 2.81 2.80 2.47 1.67 1.65 1.64 1.62 1.60 1.49 1.47 1.46 1.44 1.43 1.41 0.97 0.96 0.94
10.39 10.31
0.81
O
NH
5b
Bu
O
O
Me
1.02 0.87 0.99 0.95
1.71 2.66 1.86 2.31 3.00
f1 (ppm)

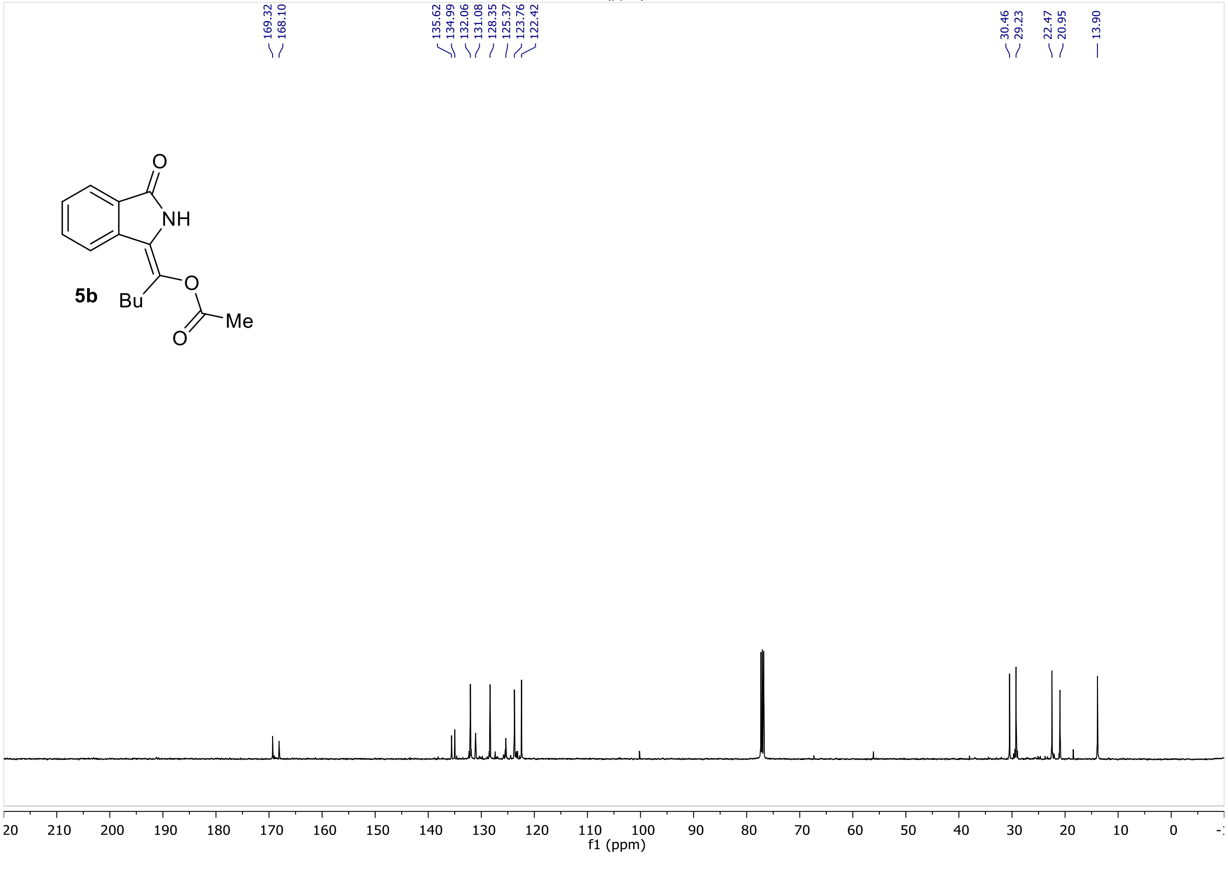

169.32 168.10
135.62 134.99 132.06 131.08 128.35 125.37 123.76 122.42
30.46 29.23 22.47 20.95 13.90
O
NH
5b
Bu
O
O
Me
f1 (ppm)

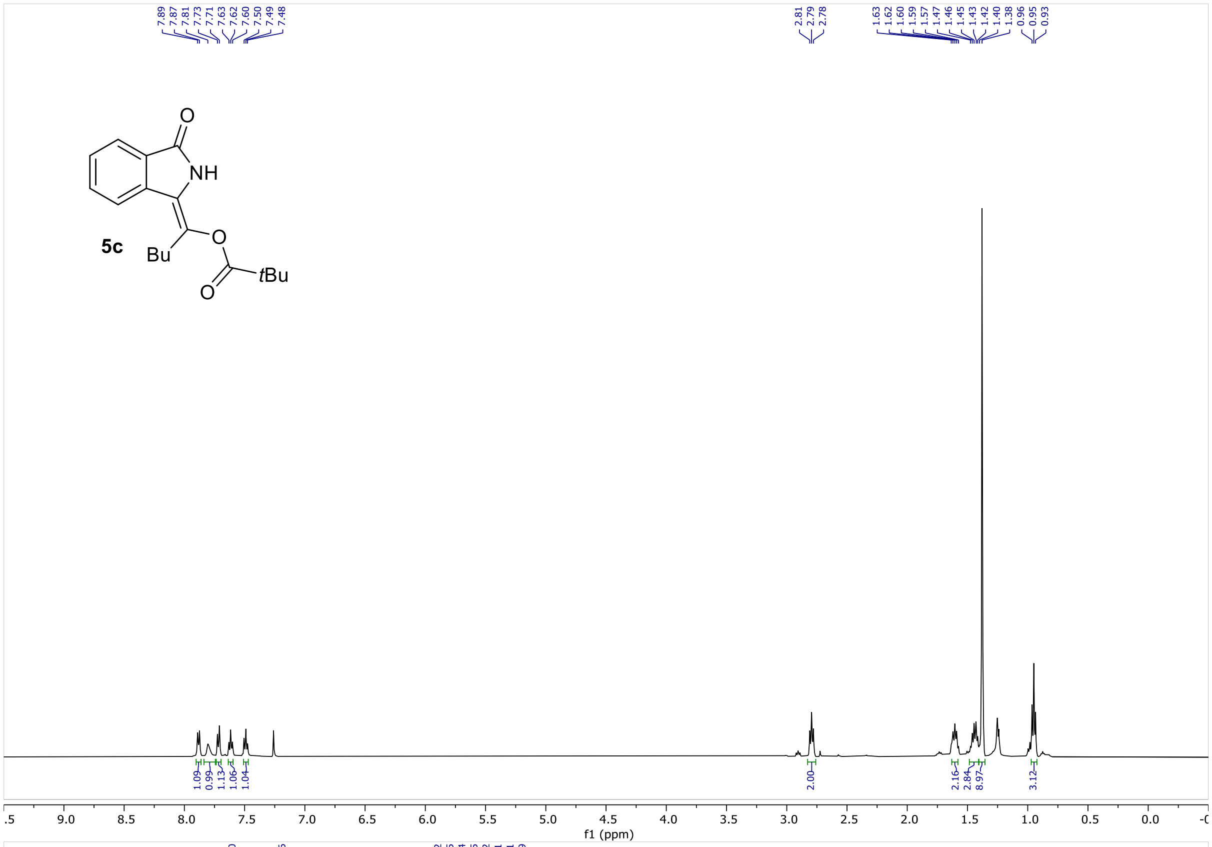

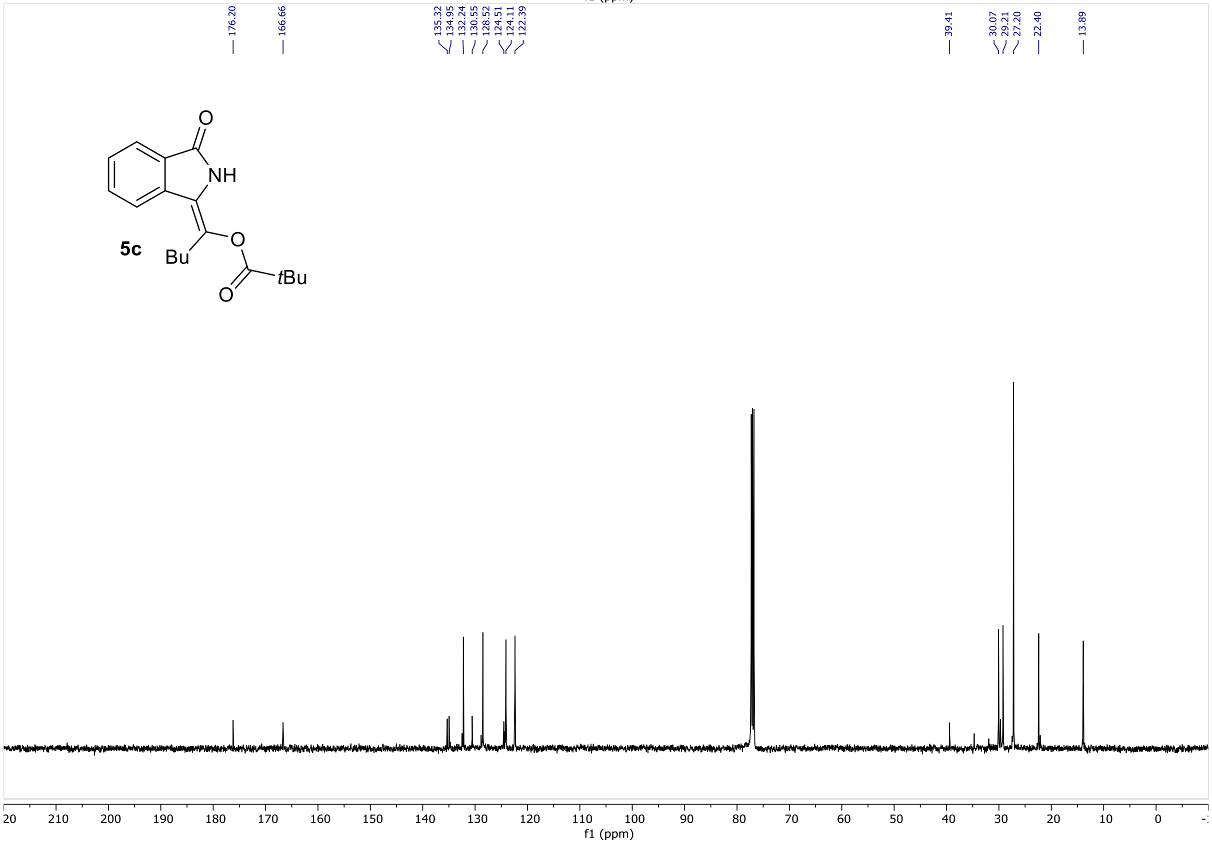

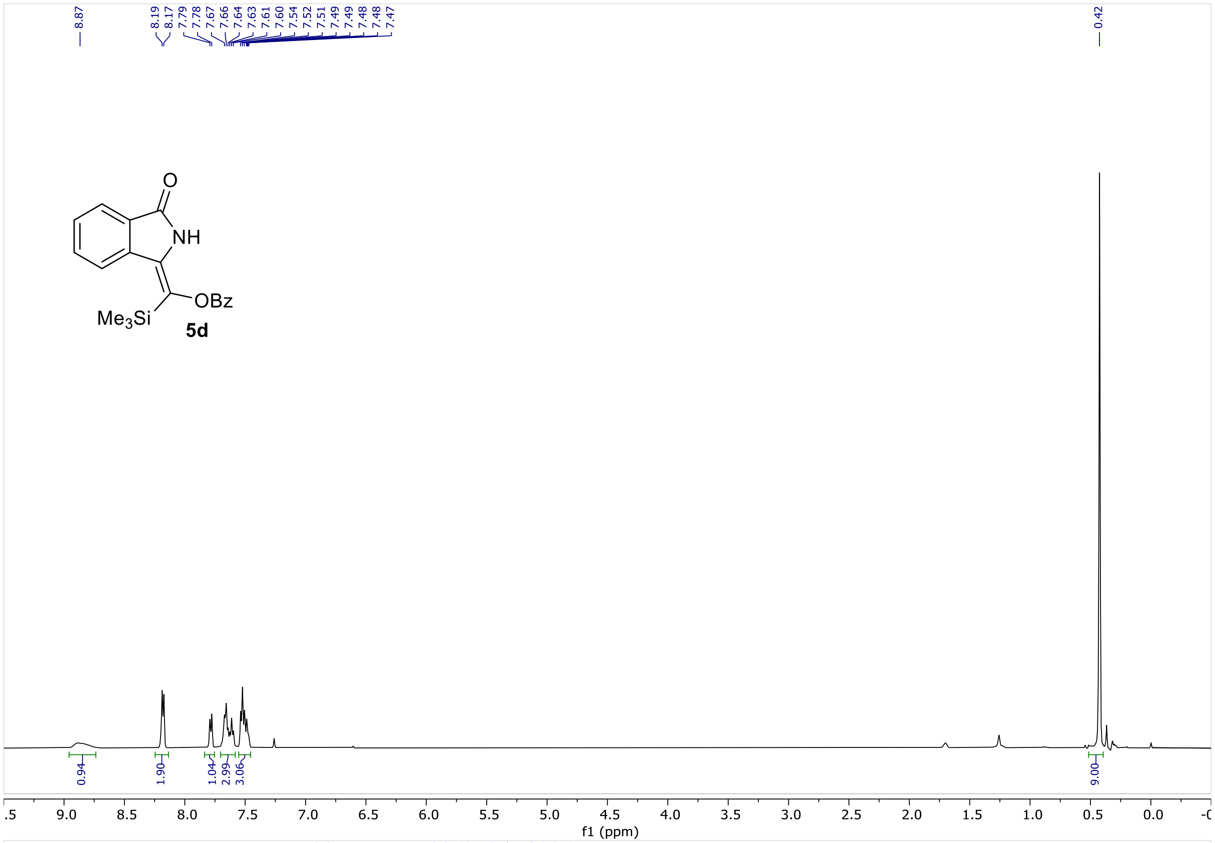

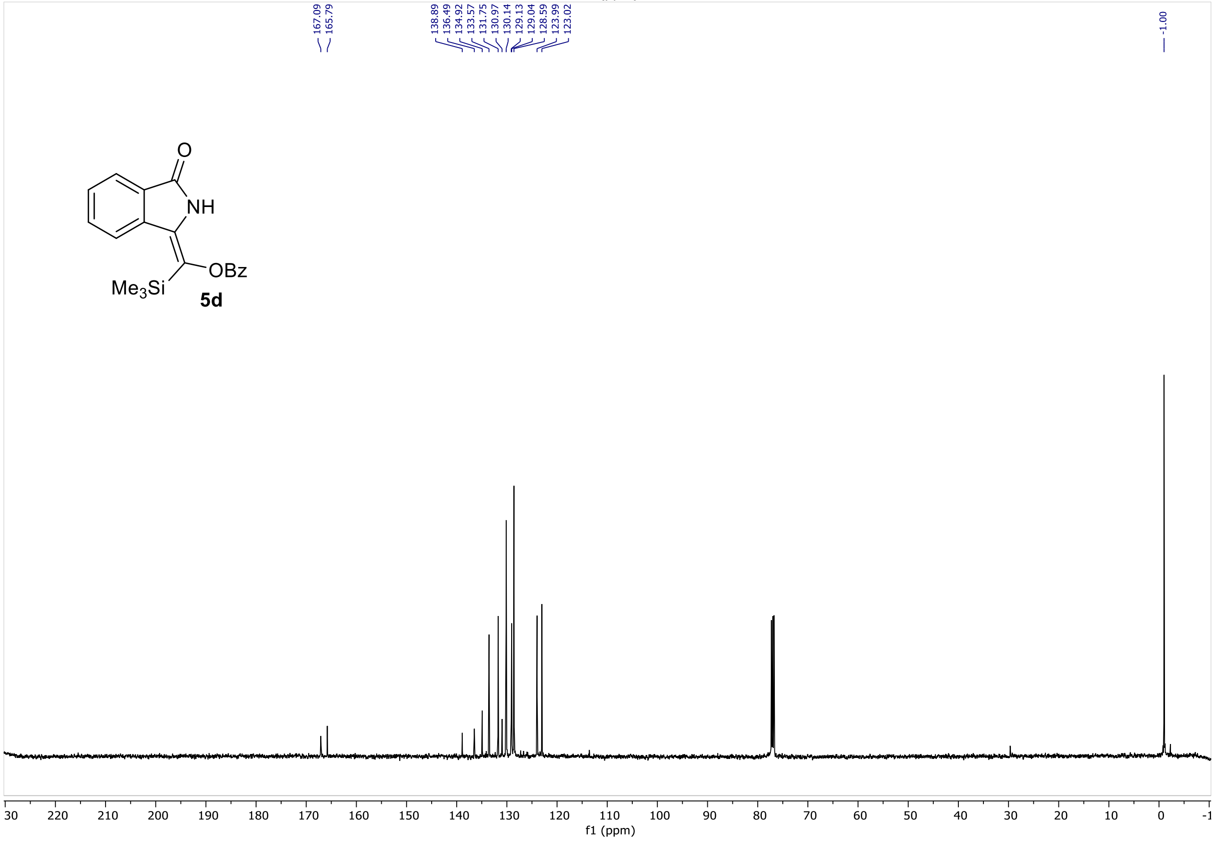

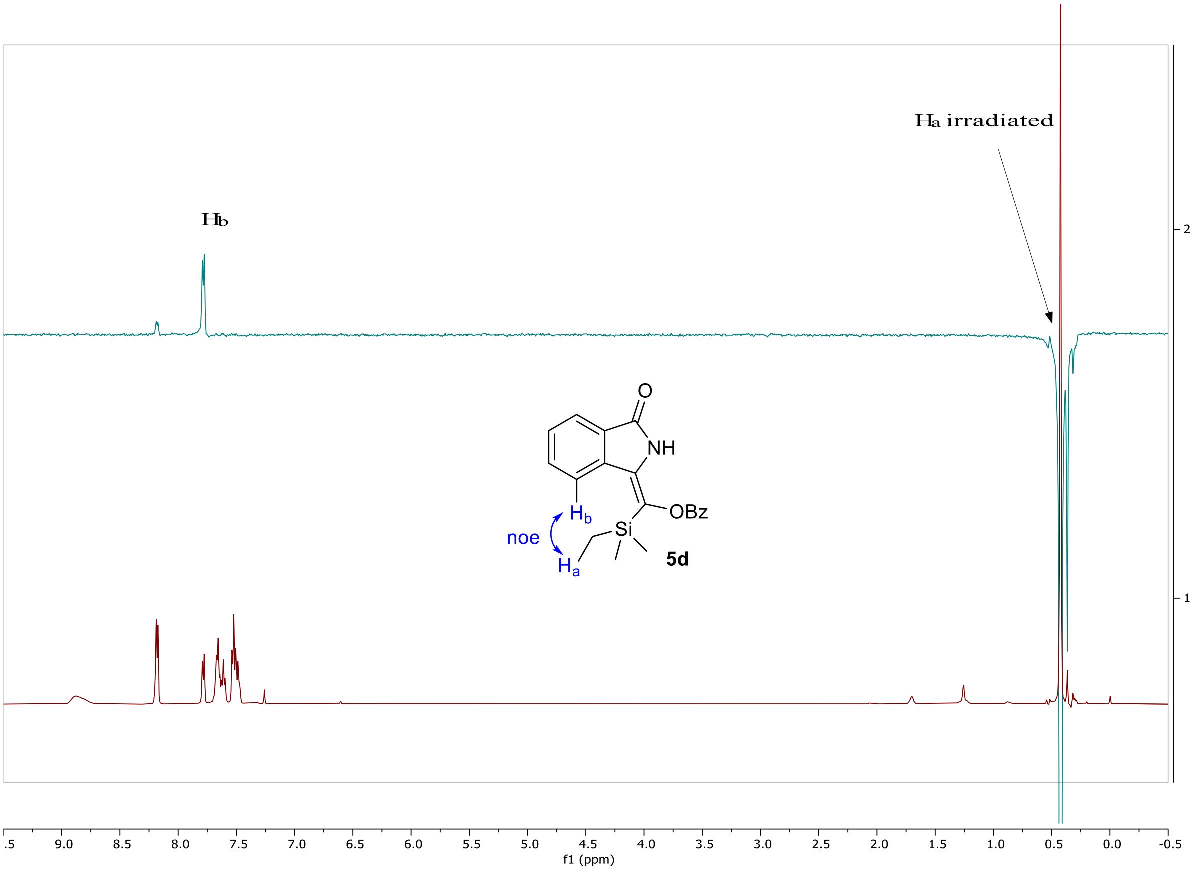

Ha irradiated
Hb
O
NH
Hb
OBz
noe
Si
Ha
5d
2
1
.5
9.0
8.5
8.0
7.5
7.0
6.5
6.0
5.5
5.0
4.5
4.0
3.5
3.0
2.5
2.0
1.5
1.0
0.5
0.0
-0.5
f1 (ppm)

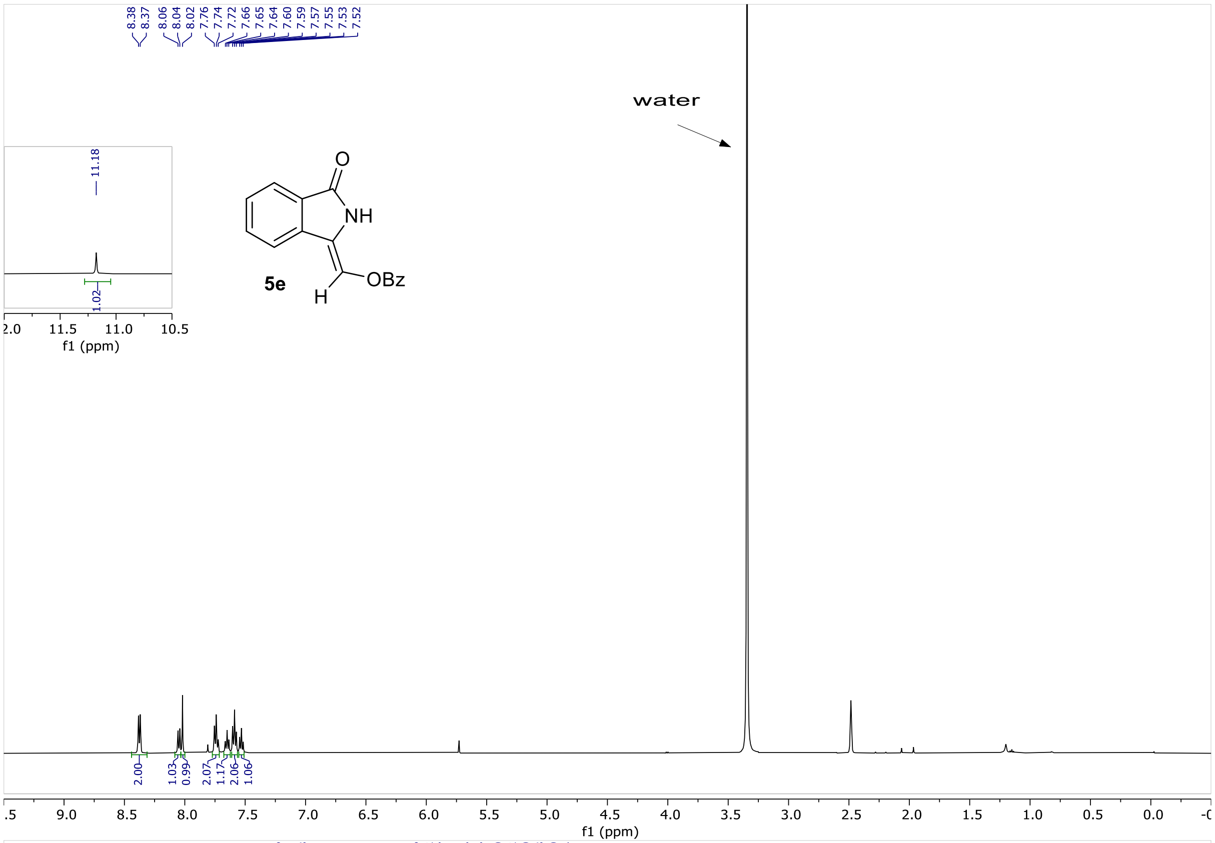
8.38
8.37
8.06
8.04
8.02
7.76
7.74
7.72
7.66
7.65
7.64
7.60
7.59
7.57
7.55
7.53
7.52
water
11.18
1.02
f1 (ppm)
O
NH
5e
H
OBz
2.00
1.03
0.99
2.07
1.17
2.06
1.06
f1 (ppm)

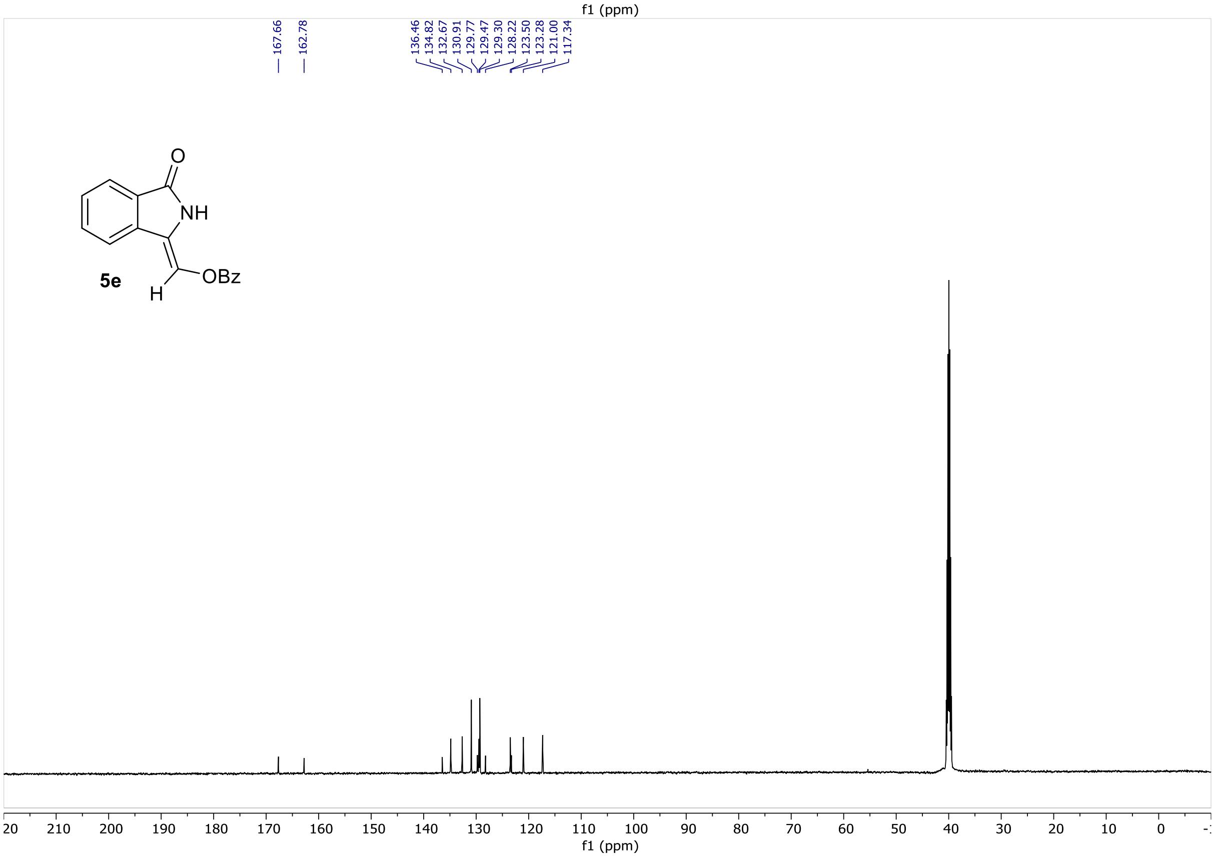
167.66
162.78
136.46
134.82
132.67
130.91
129.77
129.47
129.30
128.22
123.50
123.28
121.00
117.34
O
NH
5e
H
OBz
f1 (ppm)

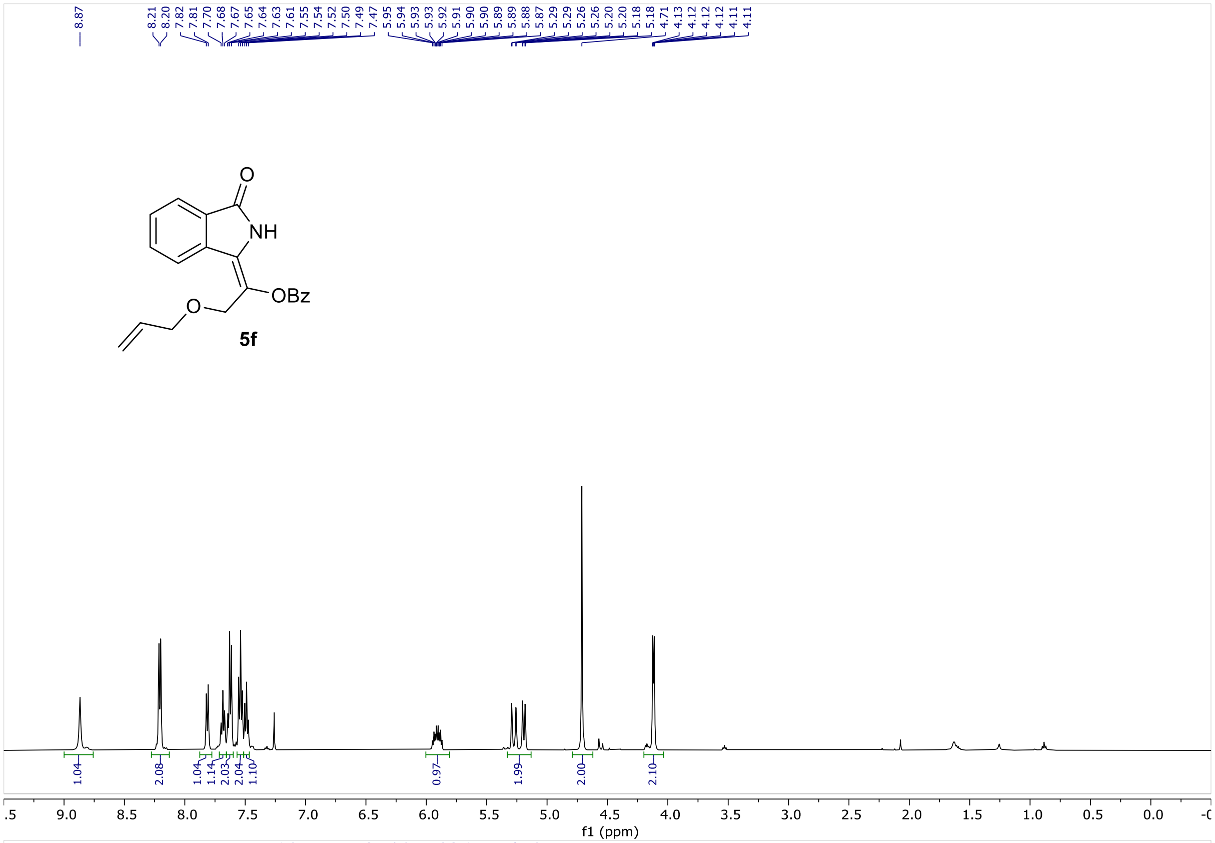

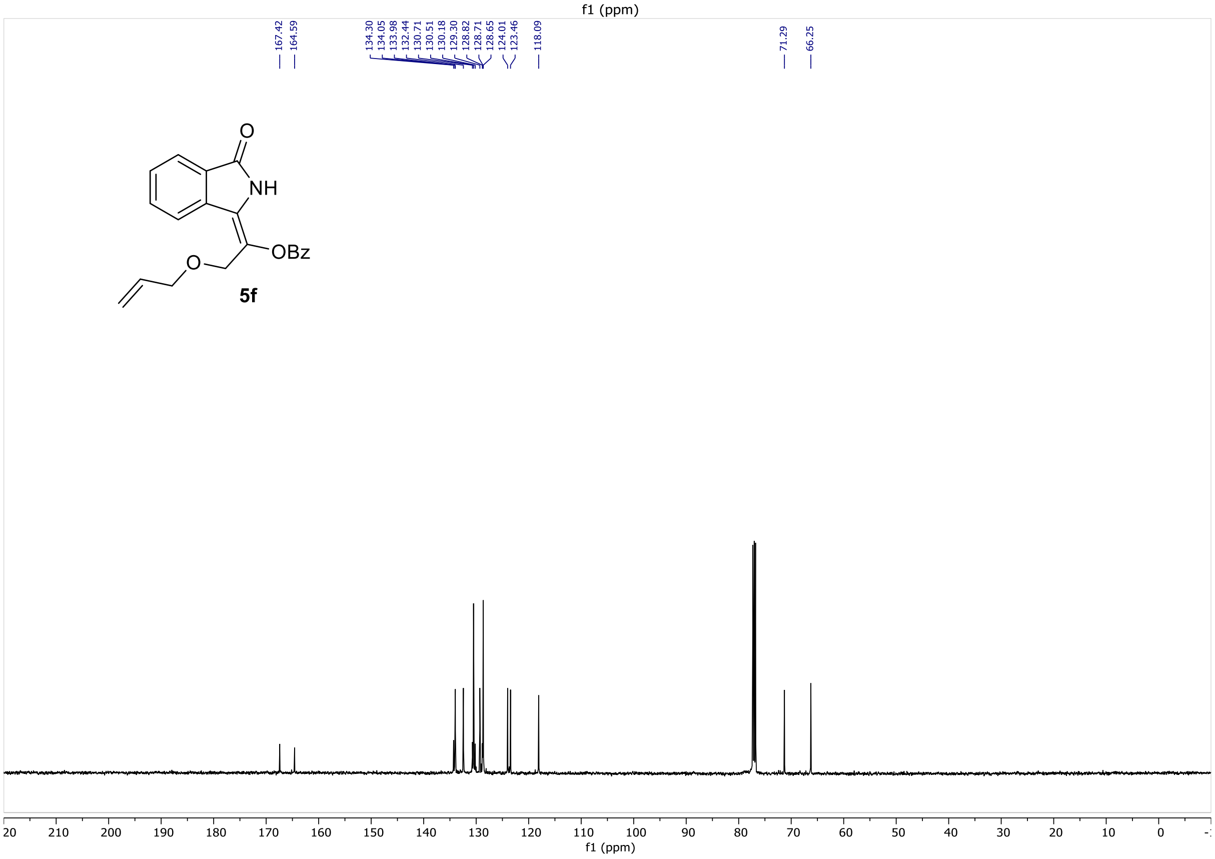

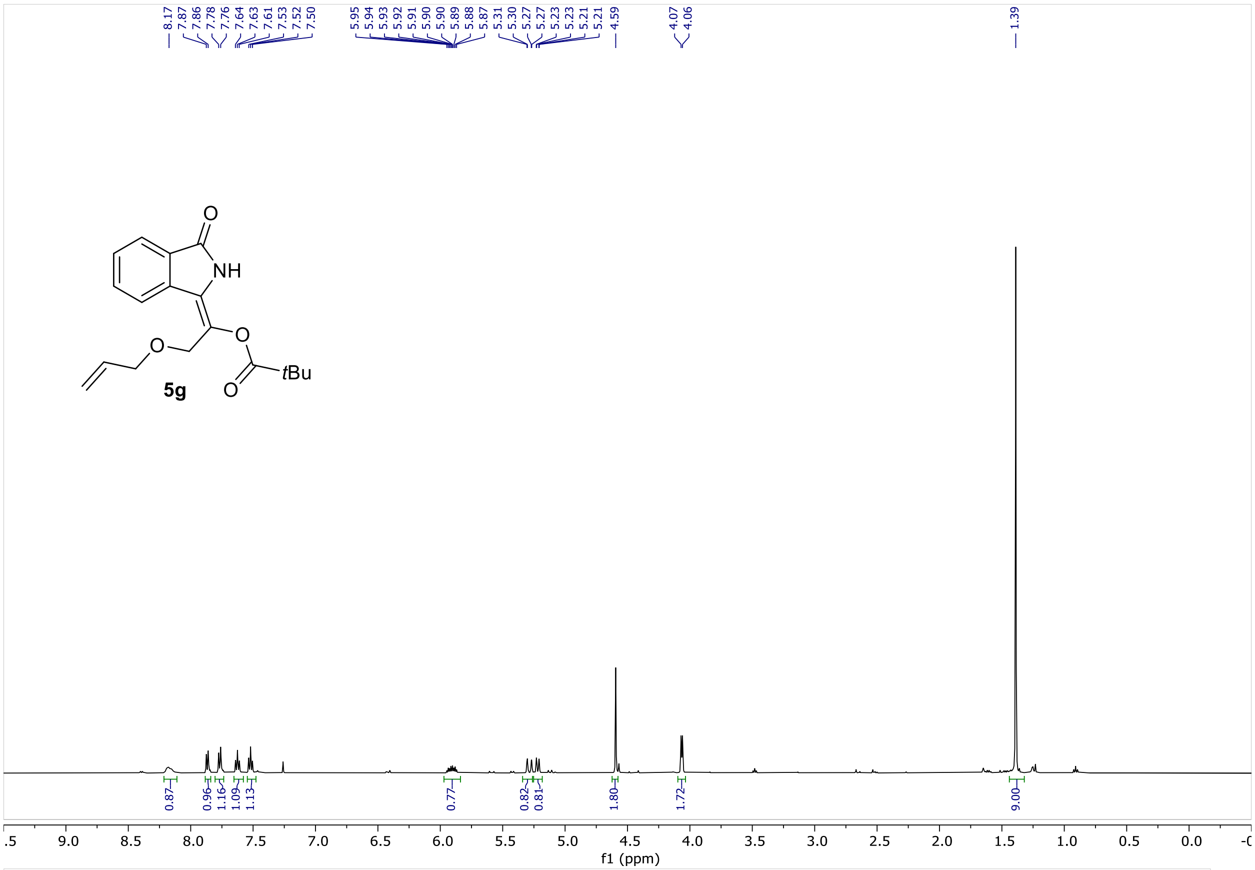

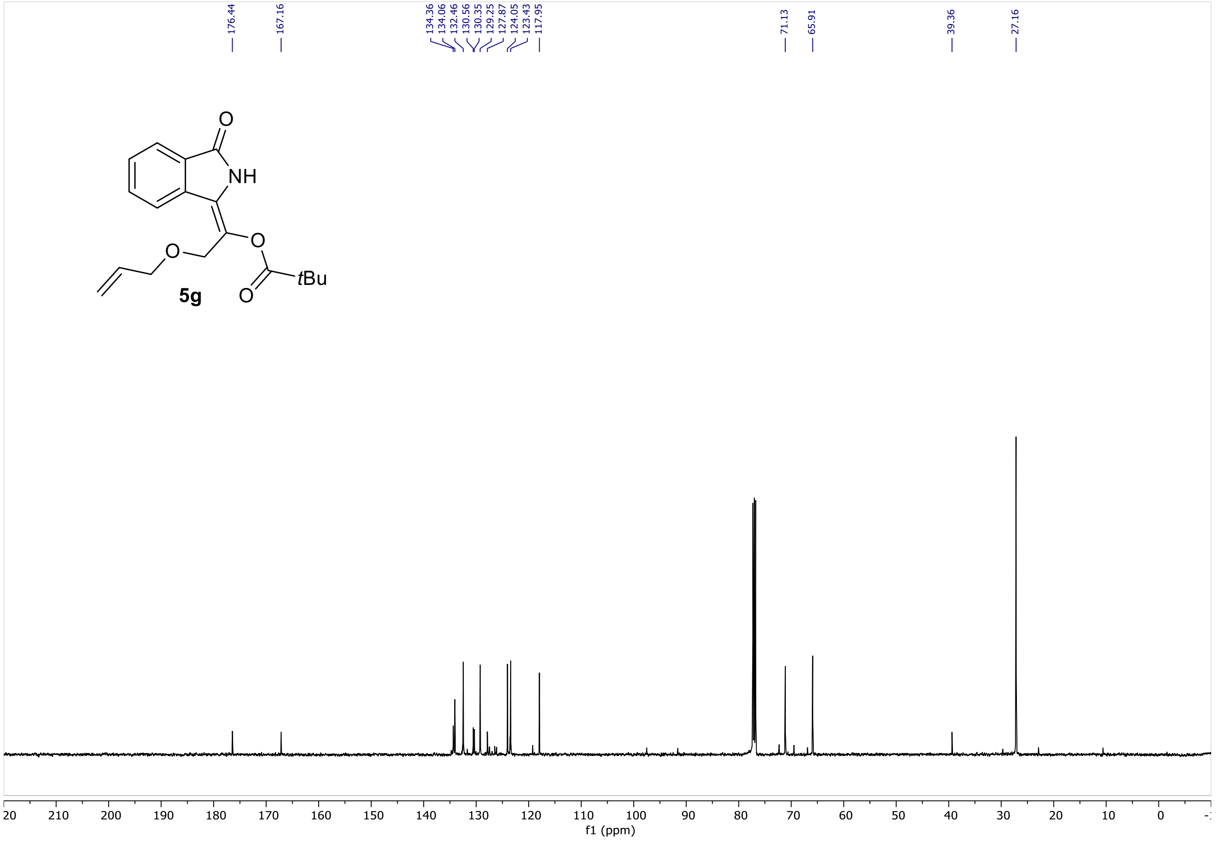

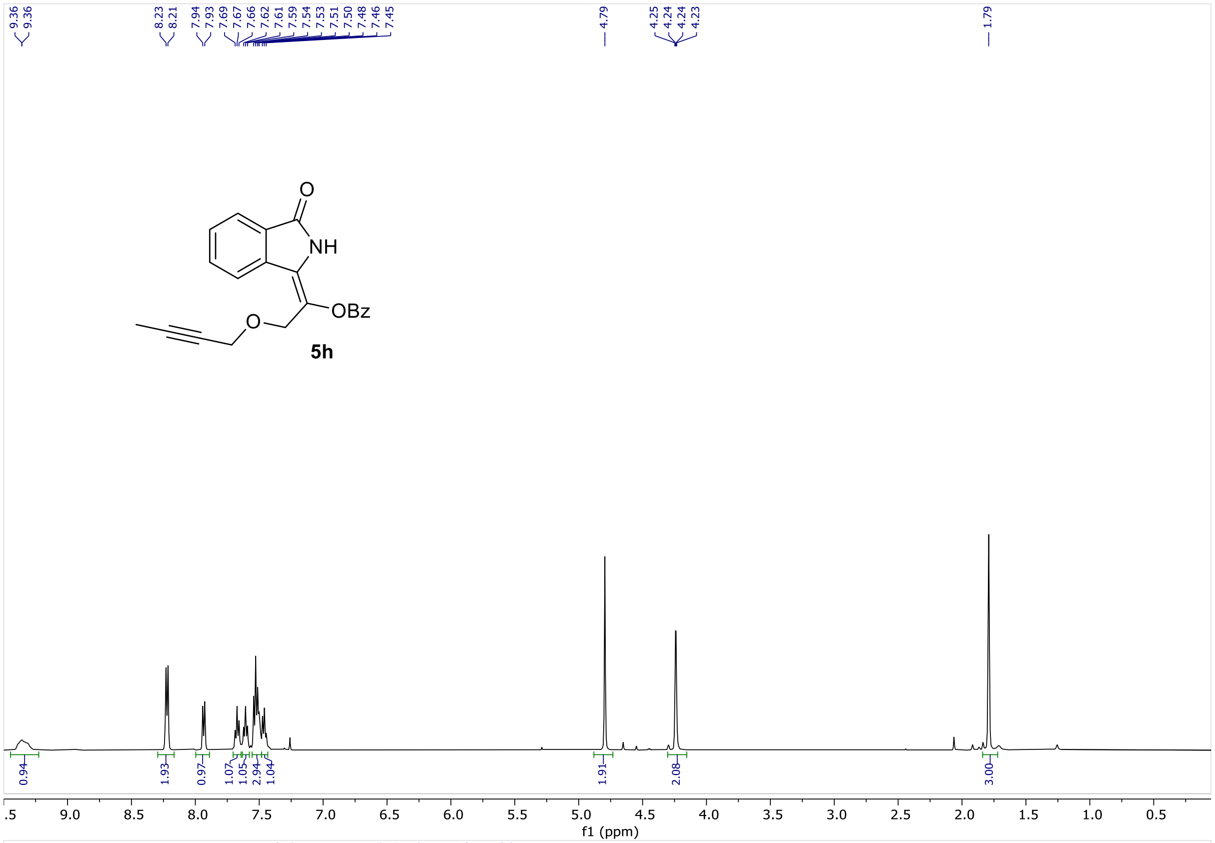
9.36
9.36
8.23
8.21
7.94
7.93
7.69
7.67
7.66
7.62
7.61
7.59
7.54
7.53
7.51
7.50
7.48
7.46
7.45
4.79
4.25
4.24
4.24
4.23
1.79
O
NH
OBz
O
5h
0.94
1.93
0.97
1.07
1.05
2.94
1.04
1.91
2.08
3.00
f1 (ppm)

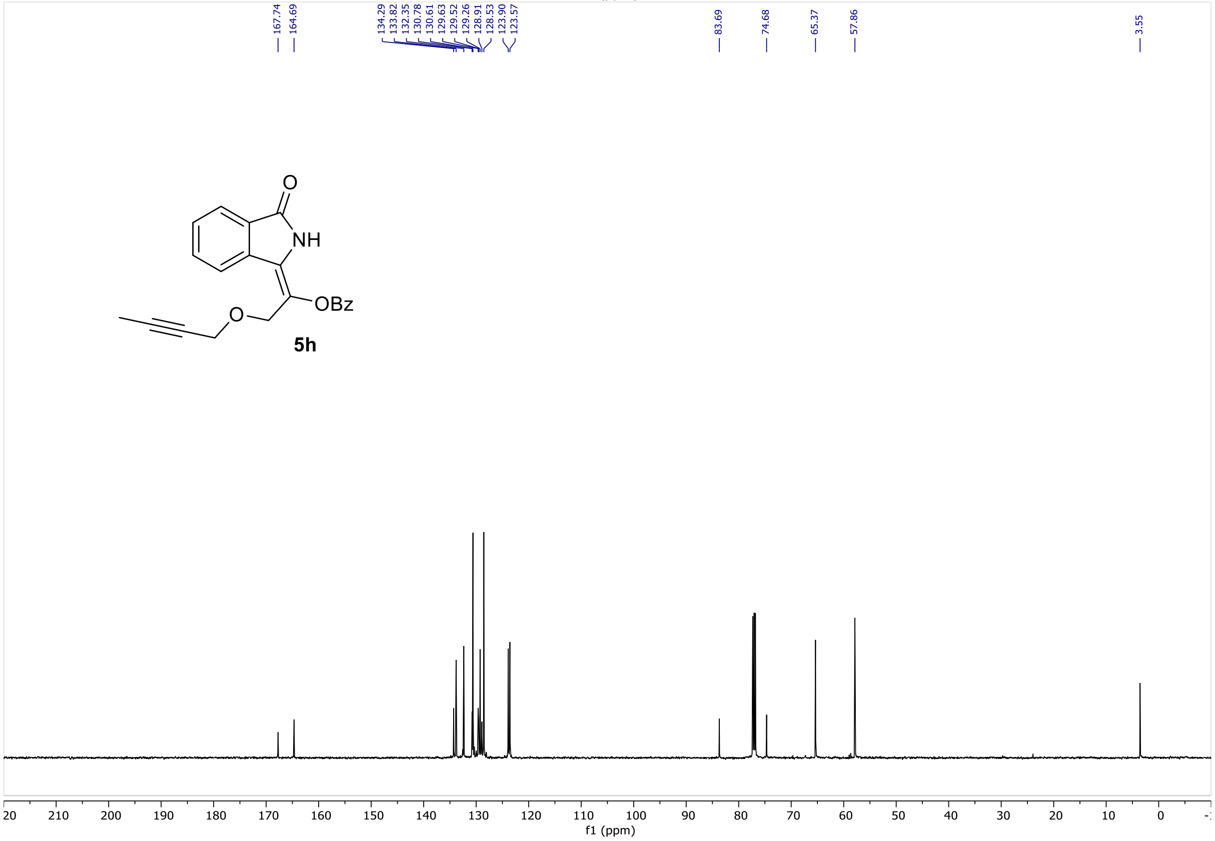
167.74
164.69
134.29
133.82
132.35
130.78
130.61
129.63
129.52
129.26
128.91
128.53
123.90
123.57
83.69
74.68
65.37
57.86
3.55
O
NH
OBz
O
5h
f1 (ppm)

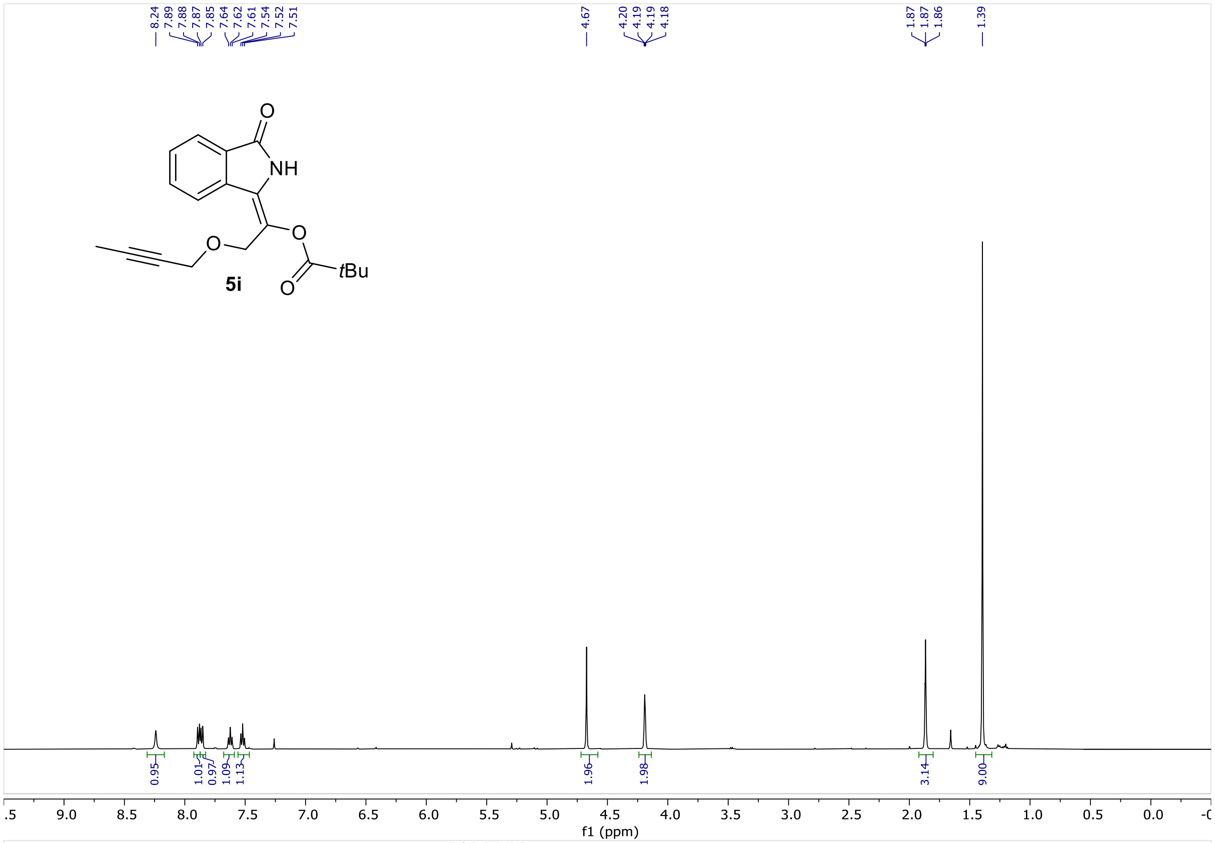

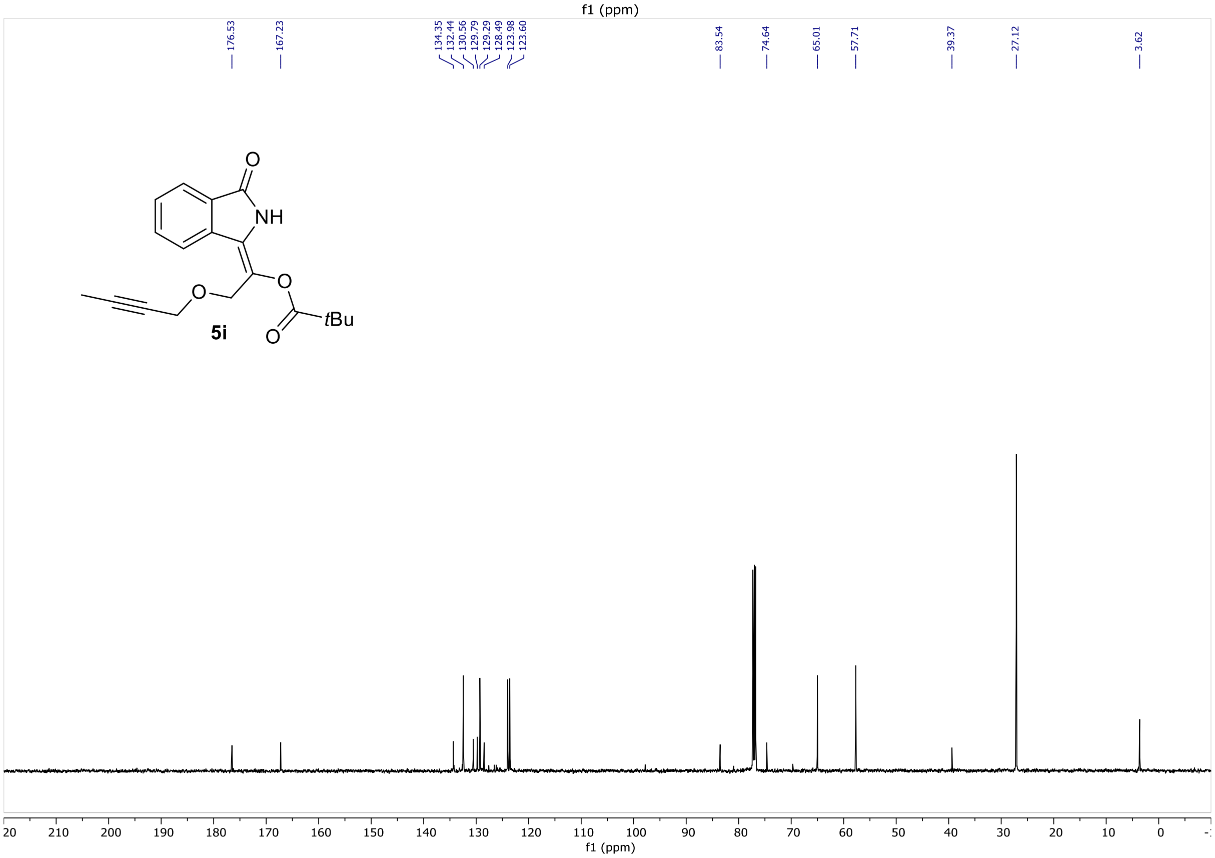

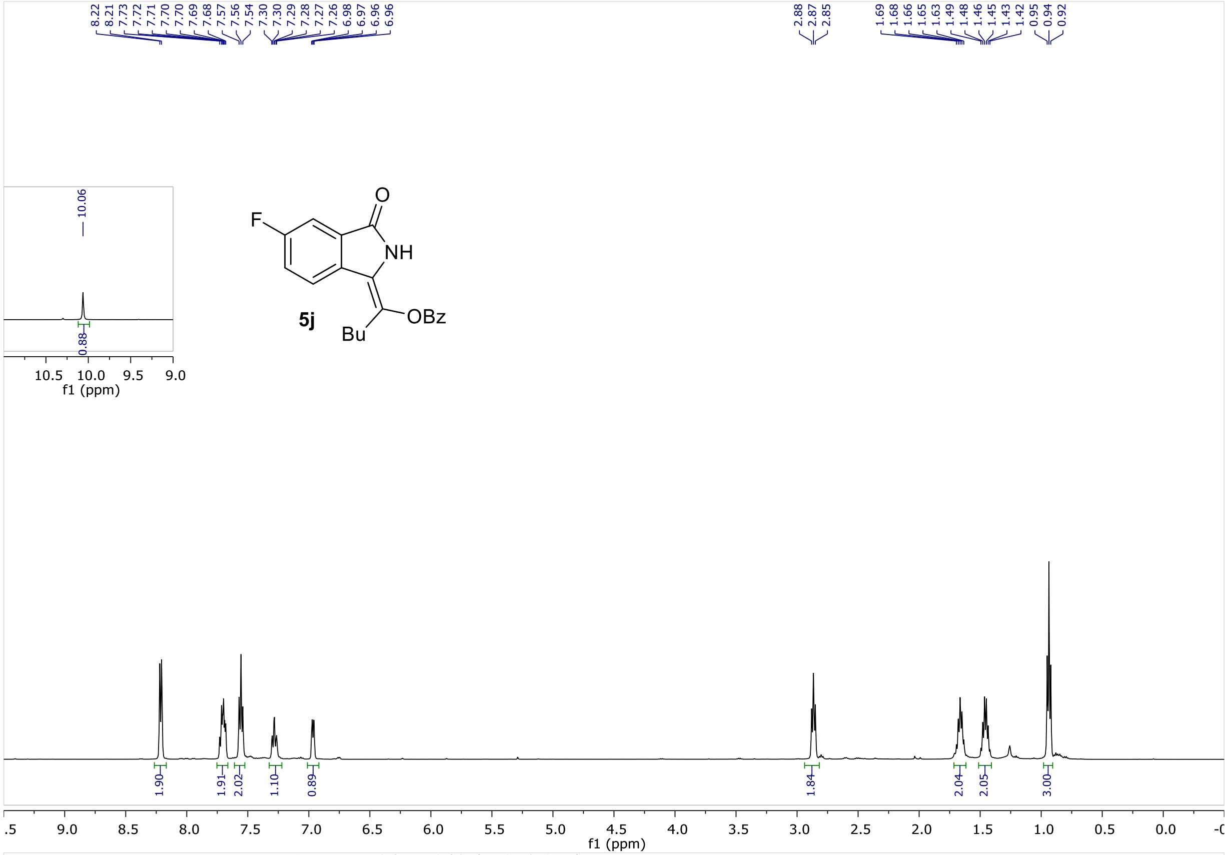

5j
f1 (ppm)

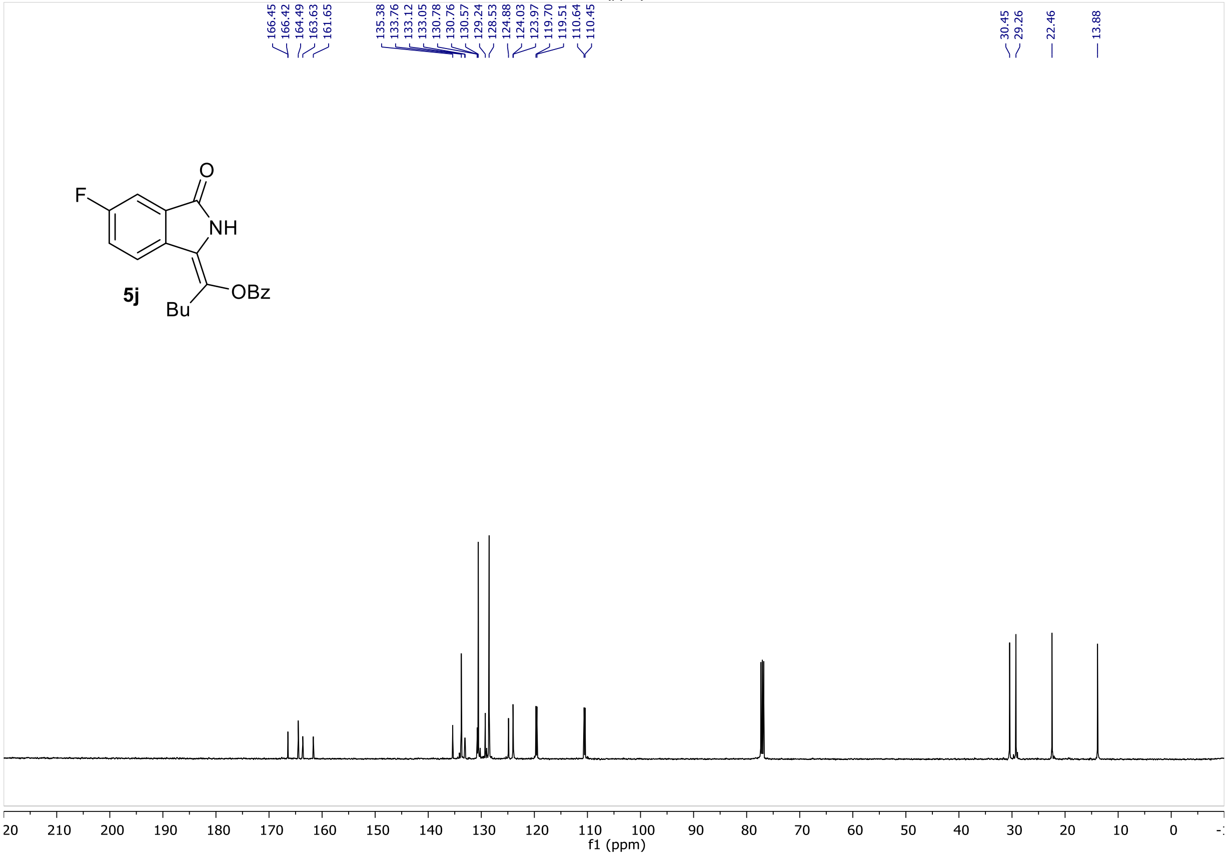

5j
f1 (ppm)

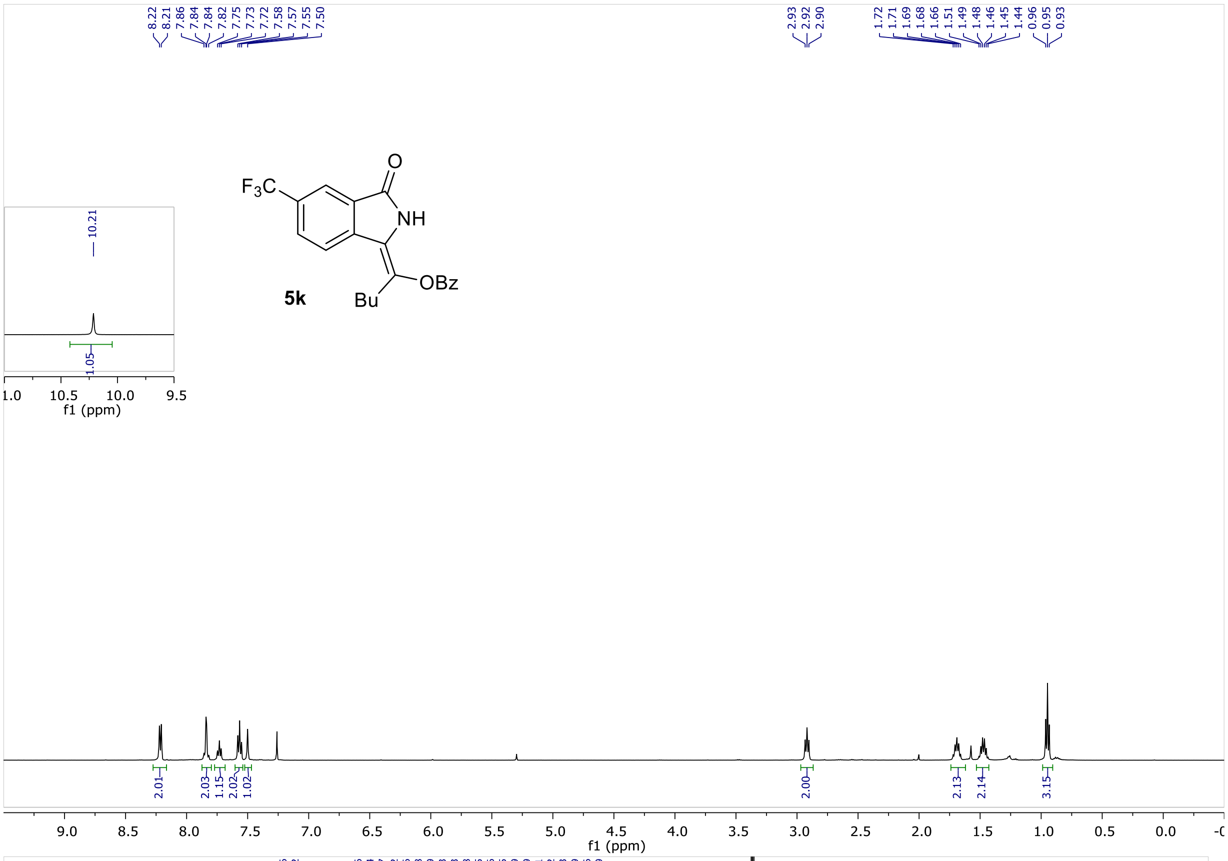
5k
f1 (ppm)

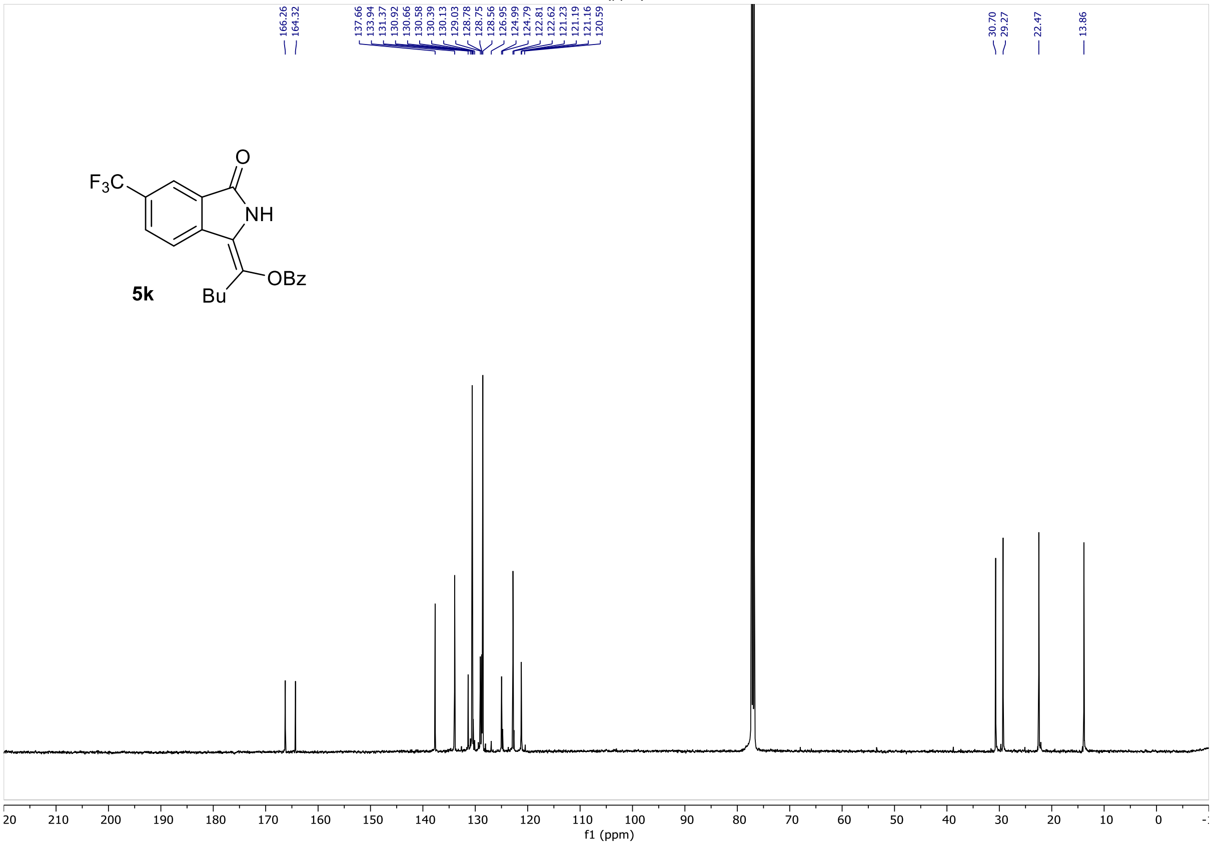
5k
f1 (ppm)

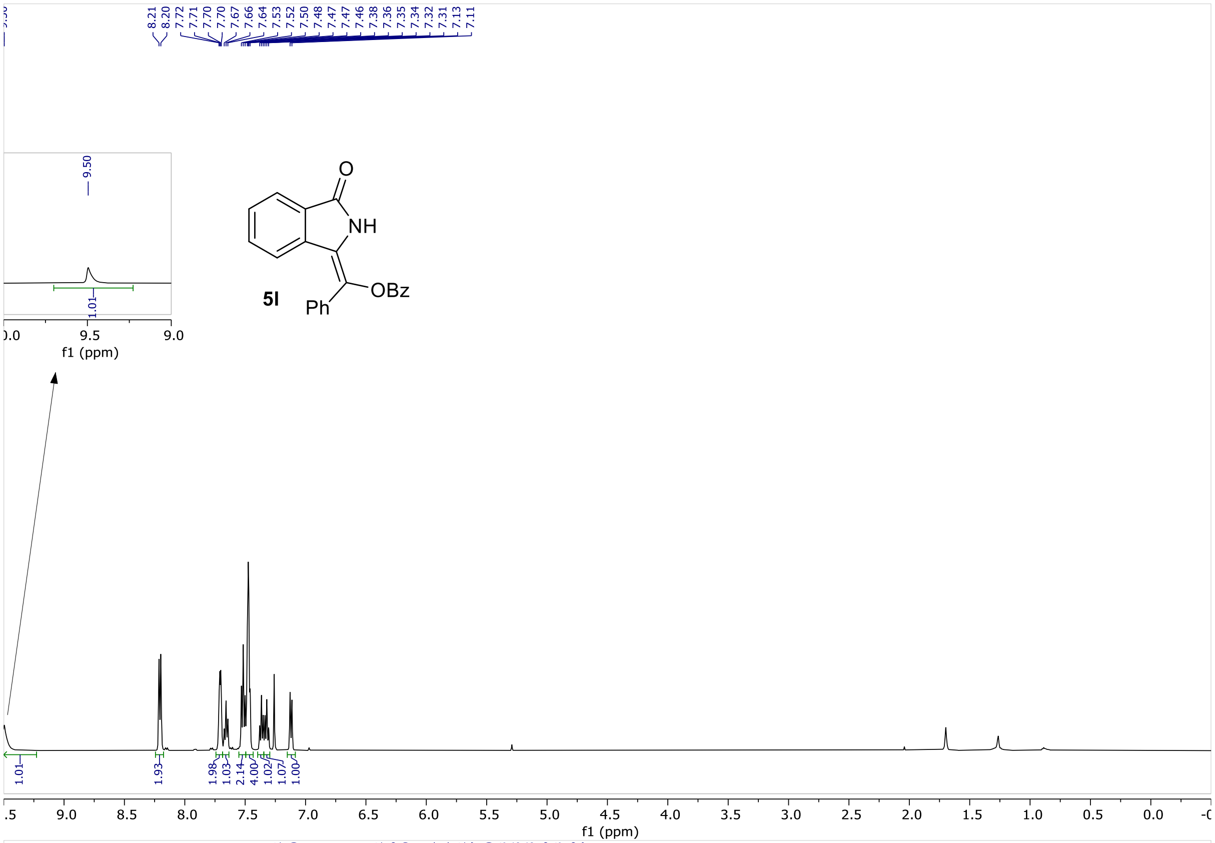
8.21
8.20
7.72
7.71
7.70
7.70
7.67
7.66
7.64
7.53
7.52
7.50
7.48
7.47
7.47
7.46
7.38
7.36
7.35
7.34
7.32
7.31
7.13
7.11
9.50
1.01
9.5
9.0
f1 (ppm)
O
NH
OBz
5l
Ph
1.01
1.93
1.98
1.03
2.14
4.00
1.02
1.07
1.00
9.0
8.5
8.0
7.5
7.0
6.5
6.0
5.5
5.0
4.5
4.0
3.5
3.0
2.5
2.0
1.5
1.0
0.5
0.0
f1 (ppm)

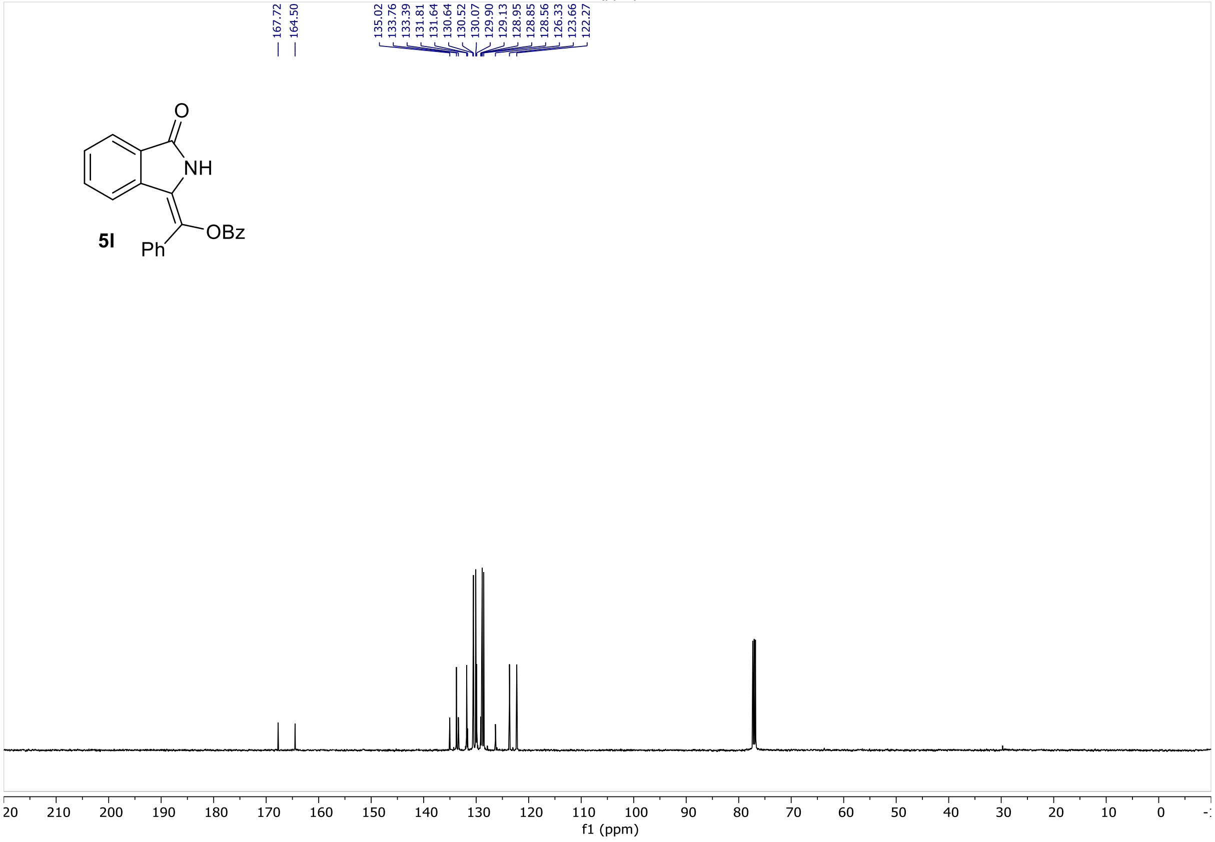
167.72
164.50
135.02
133.76
133.39
131.81
131.64
130.64
130.52
130.07
129.90
129.13
128.95
128.85
128.56
126.33
123.66
122.27
O
NH
OBz
5l
Ph
210
200
190
180
170
160
150
140
130
120
110
100
90
80
70
60
50
40
30
20
10
0
f1 (ppm)

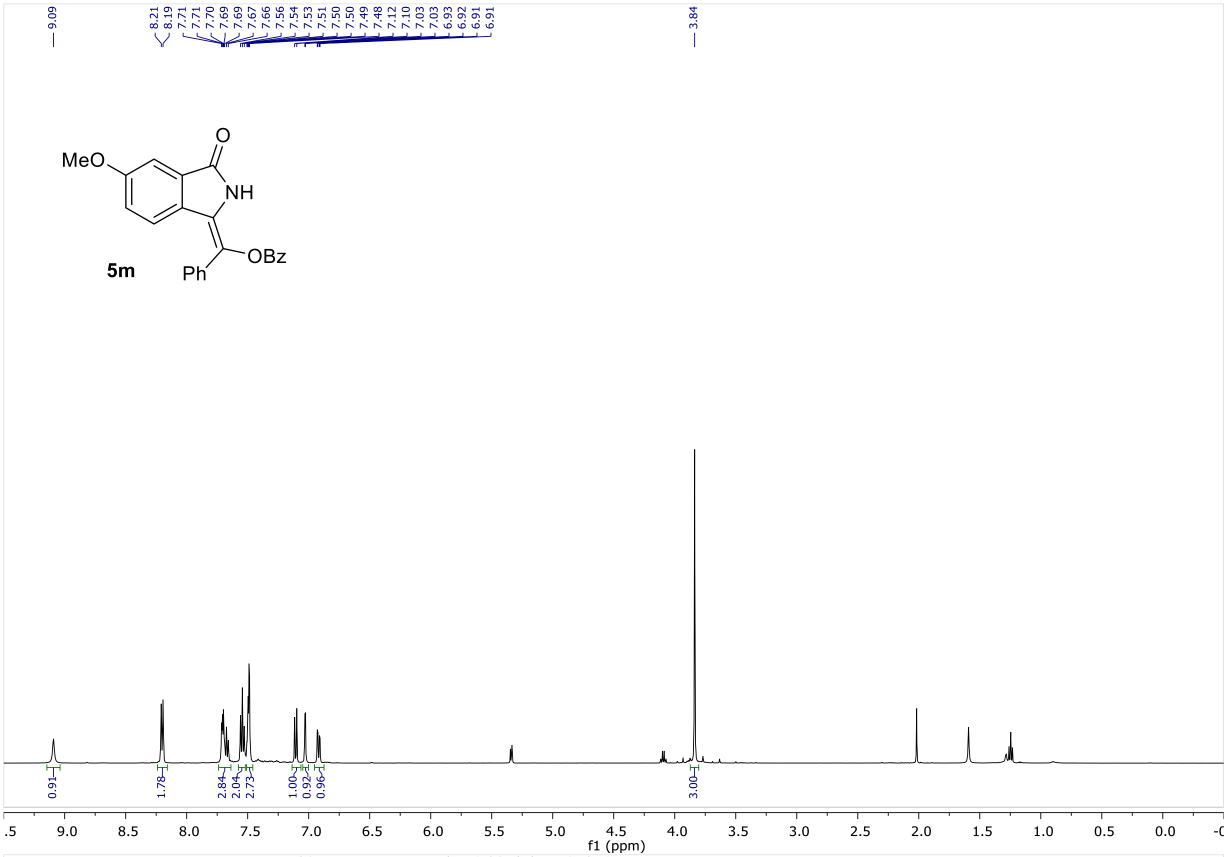
9.09
8.21
8.19
7.71
7.71
7.70
7.69
7.69
7.67
7.66
7.56
7.54
7.53
7.51
7.50
7.50
7.49
7.48
7.12
7.10
7.03
7.03
6.93
6.92
6.91
6.91
3.84
MeO
O
NH
OBz
Ph
5m
0.91
1.78
2.84
2.04
2.73
1.00
0.92
0.96
3.00
f1 (ppm)

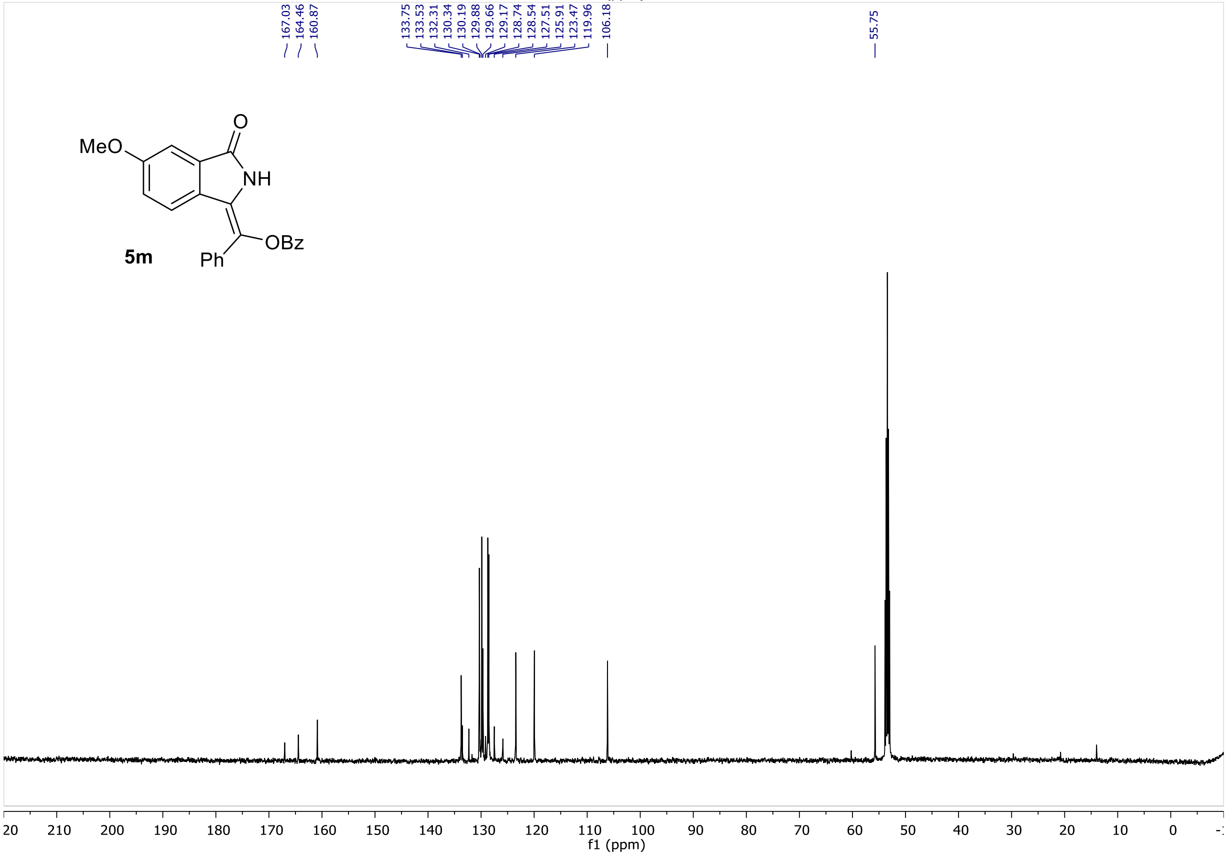
167.03
164.46
160.87
133.75
133.53
132.31
130.34
130.19
129.88
129.66
129.17
128.74
128.54
127.51
125.91
123.47
119.96
106.18
55.75
MeO
O
NH
OBz
Ph
5m
f1 (ppm)

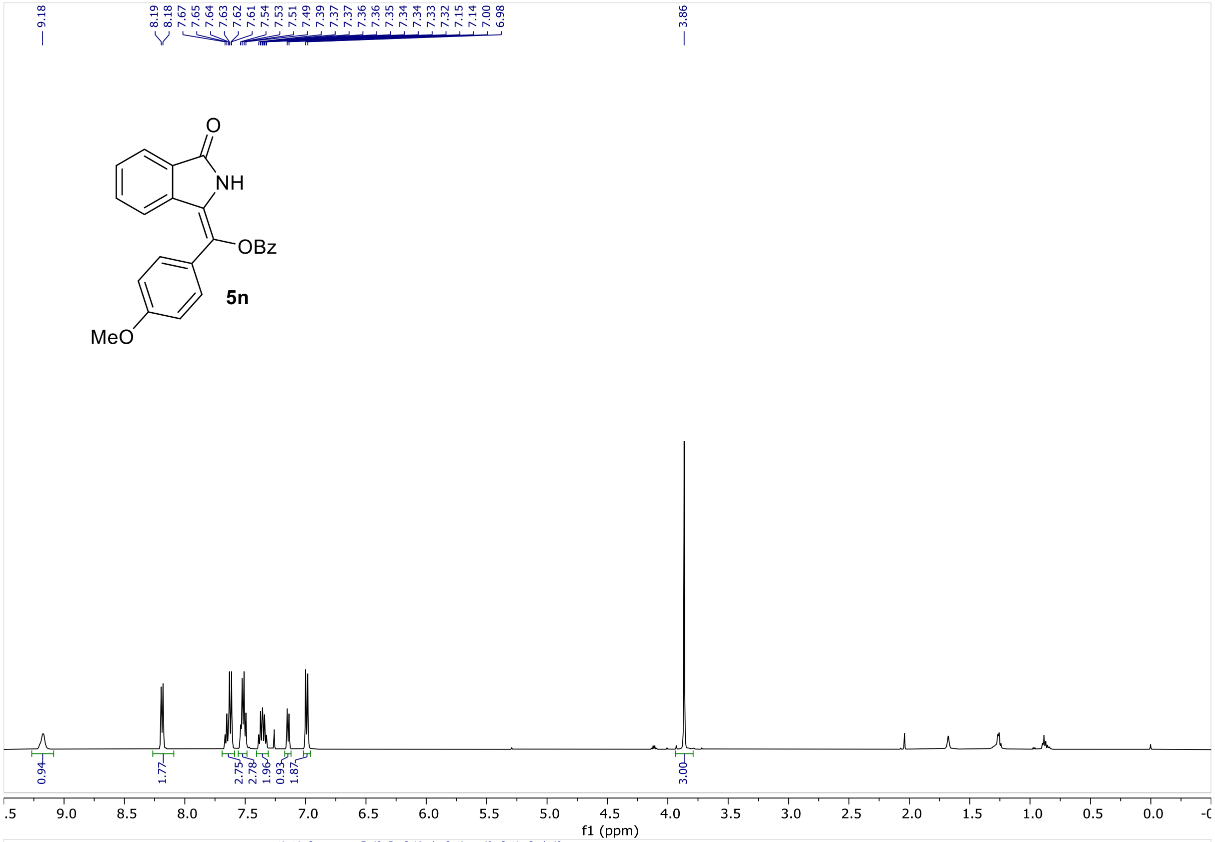

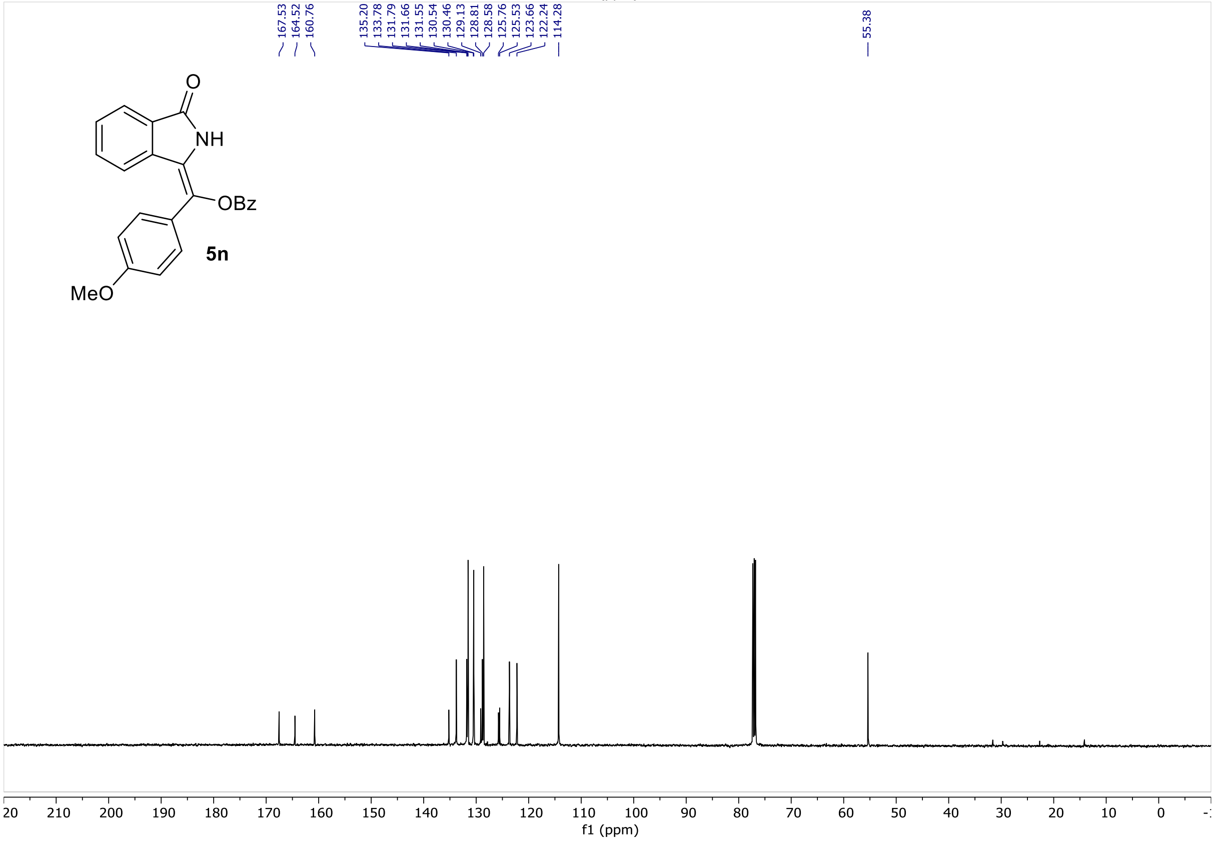

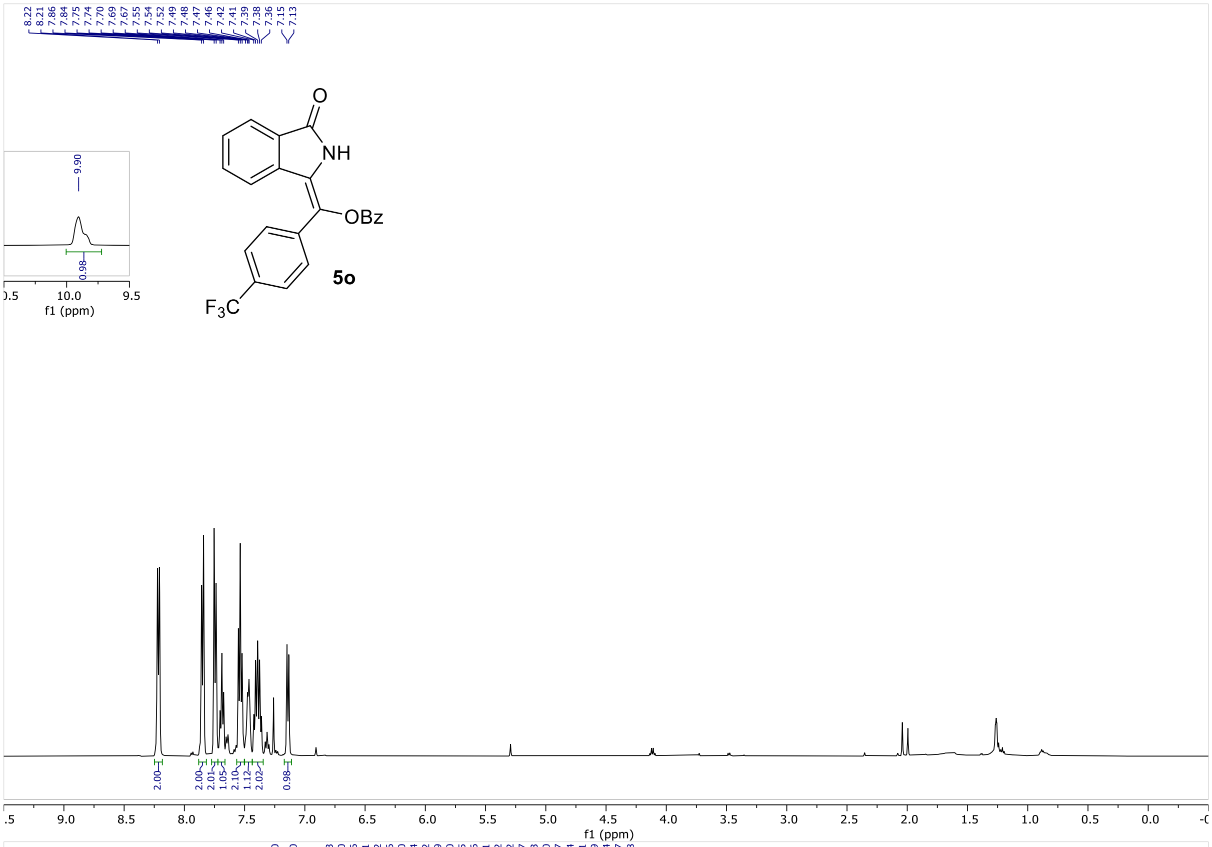
8.22
8.21
7.86
7.84
7.75
7.74
7.70
7.69
7.67
7.55
7.54
7.52
7.49
7.48
7.47
7.46
7.42
7.41
7.39
7.38
7.36
7.15
7.13
9.90
0.98
10.0
9.5
f1 (ppm)
NH
O
OBz
F3C
5o
2.00
2.00
2.01
1.05
2.10
1.12
2.02
0.98
9.0
8.5
8.0
7.5
7.0
6.5
6.0
5.5
5.0
4.5
4.0
3.5
3.0
2.5
2.0
1.5
1.0
0.5
0.0
f1 (ppm)

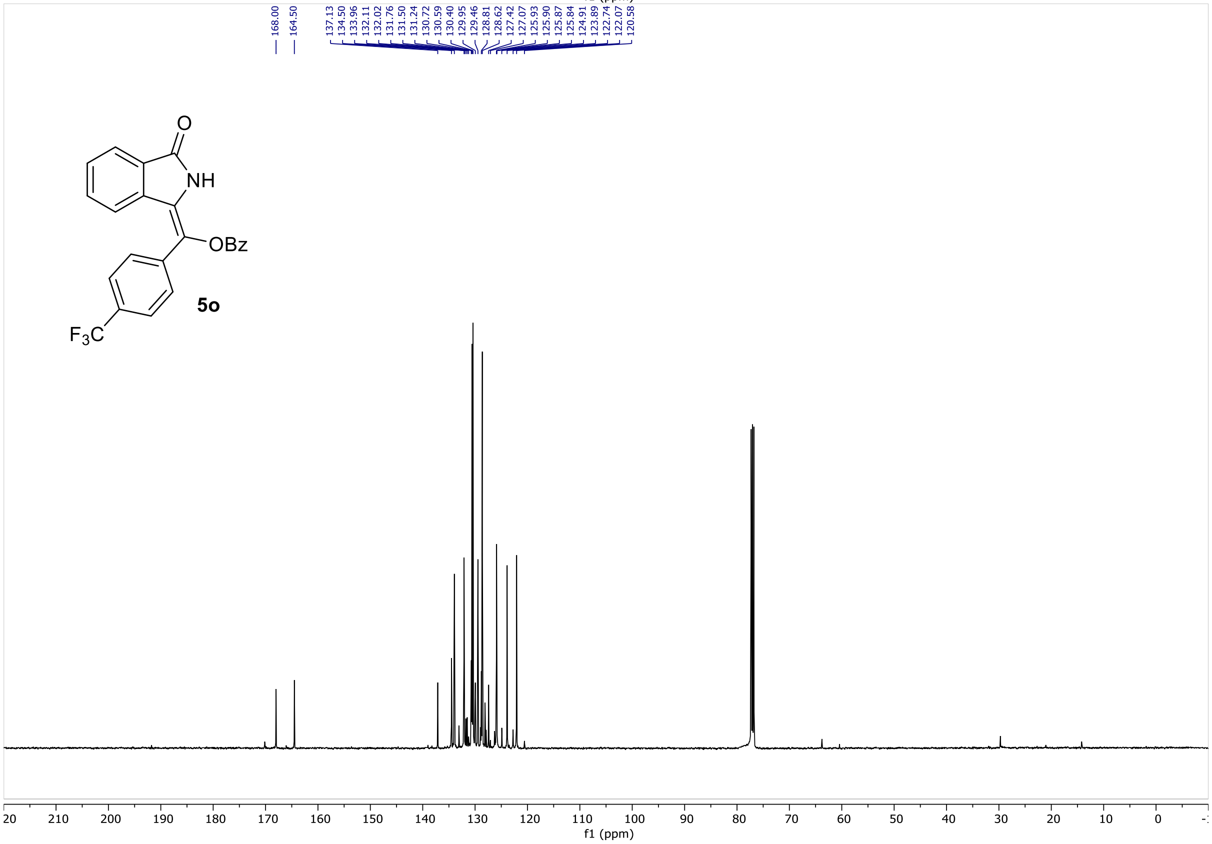
168.00
164.50
137.13
134.50
133.96
132.11
132.02
131.76
131.50
131.24
130.72
130.59
130.40
129.95
129.46
128.81
128.62
127.42
127.07
125.93
125.90
125.87
125.84
124.91
123.89
122.74
122.07
120.58
O
NH
OBz
F3C
5o
210
200
190
180
170
160
150
140
130
120
110
100
90
80
70
60
50
40
30
20
10
0
f1 (ppm)

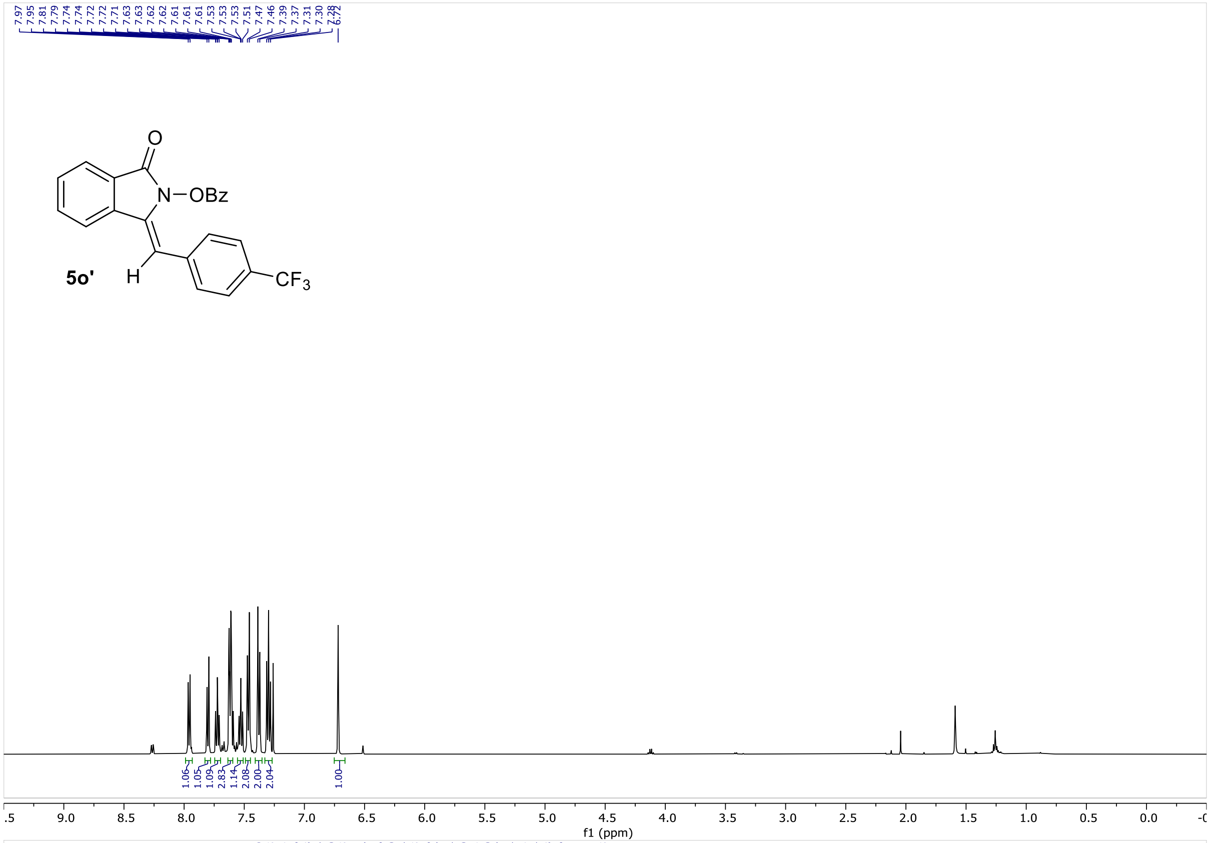

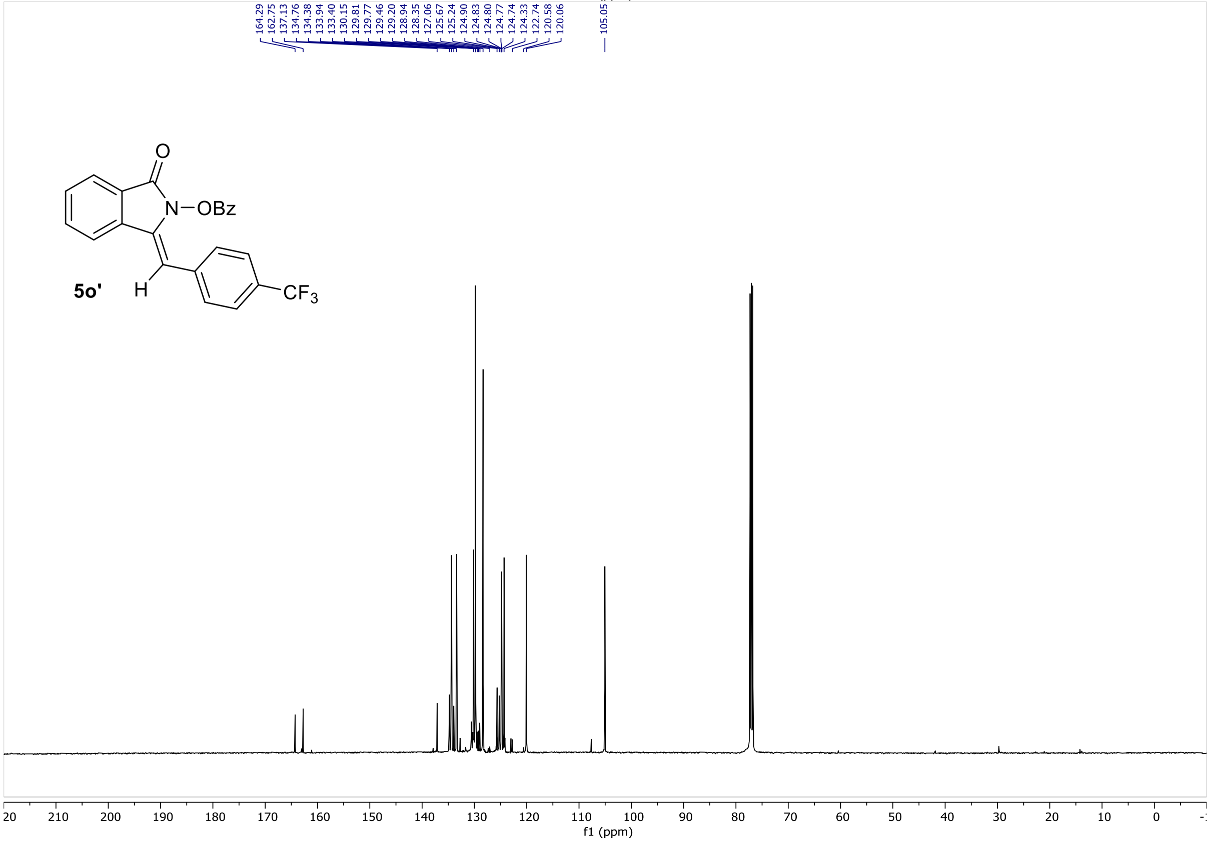

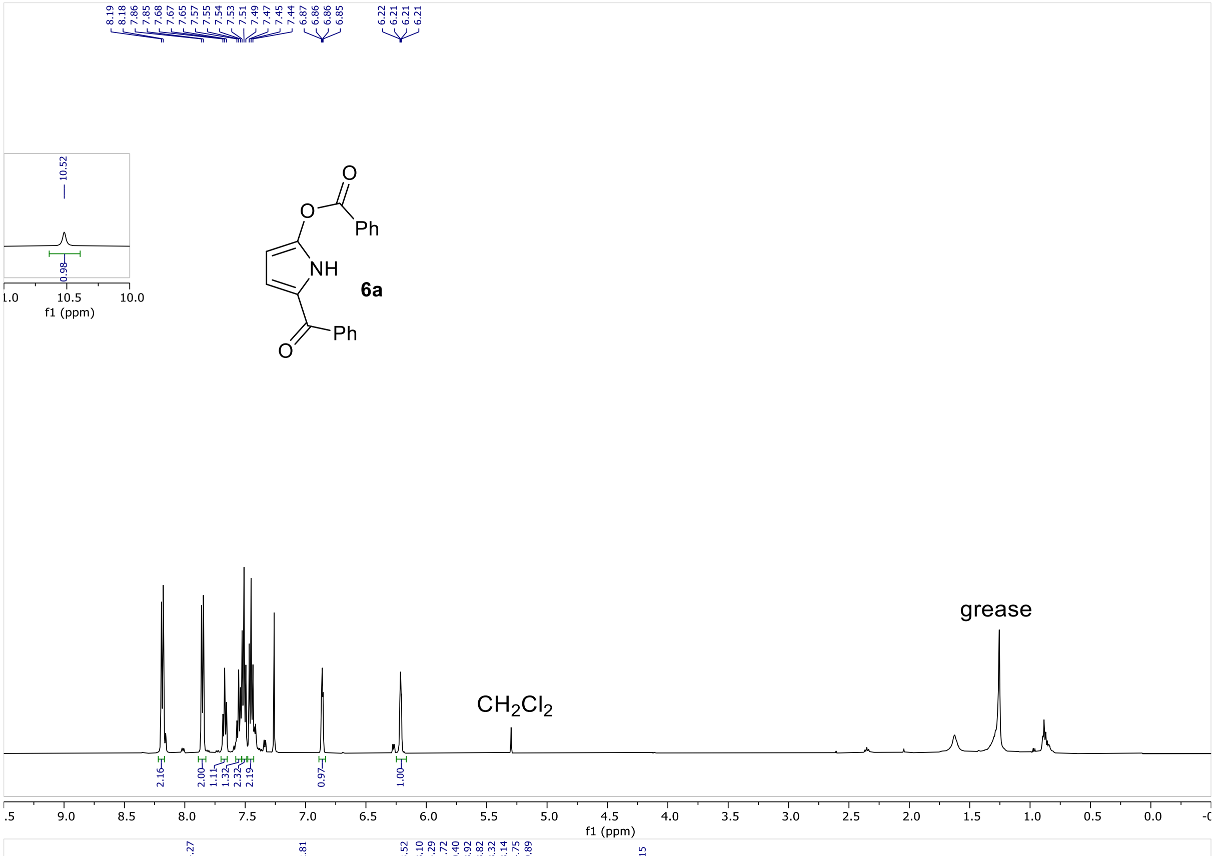
8.19
8.18
7.86
7.85
7.68
7.67
7.65
7.57
7.55
7.54
7.53
7.51
7.49
7.47
7.45
7.44
6.87
6.86
6.86
6.85
6.22
6.21
6.21
6.21
10.52
0.98
f1 (ppm)
O
Ph
NH
6a
Ph
O
grease
CH_2Cl_2
2.16
2.00
1.11
1.32
2.32
2.19
0.97
1.00
f1 (ppm)

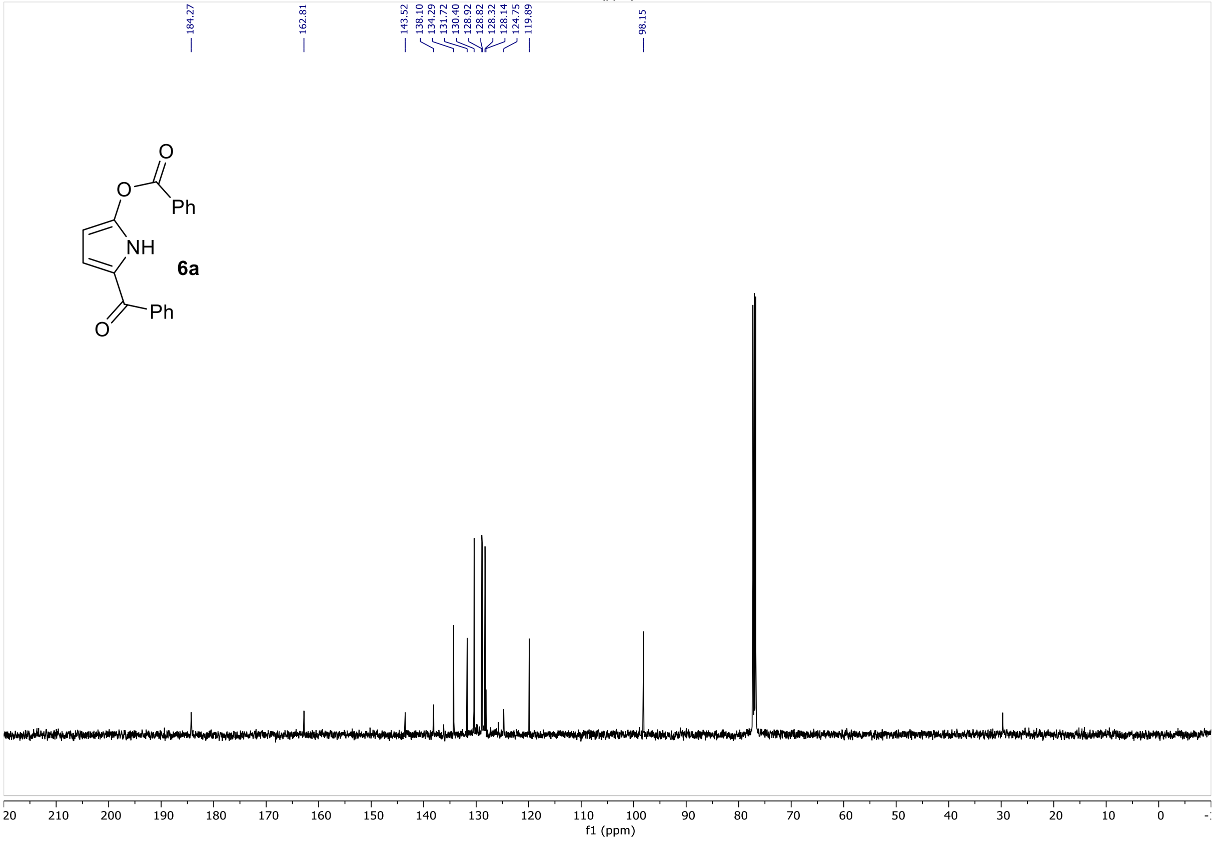
184.27
162.81
143.52
138.10
134.29
131.72
130.40
128.92
128.82
128.32
128.14
124.75
119.89
98.15
O
Ph
NH
6a
Ph
O
f1 (ppm)

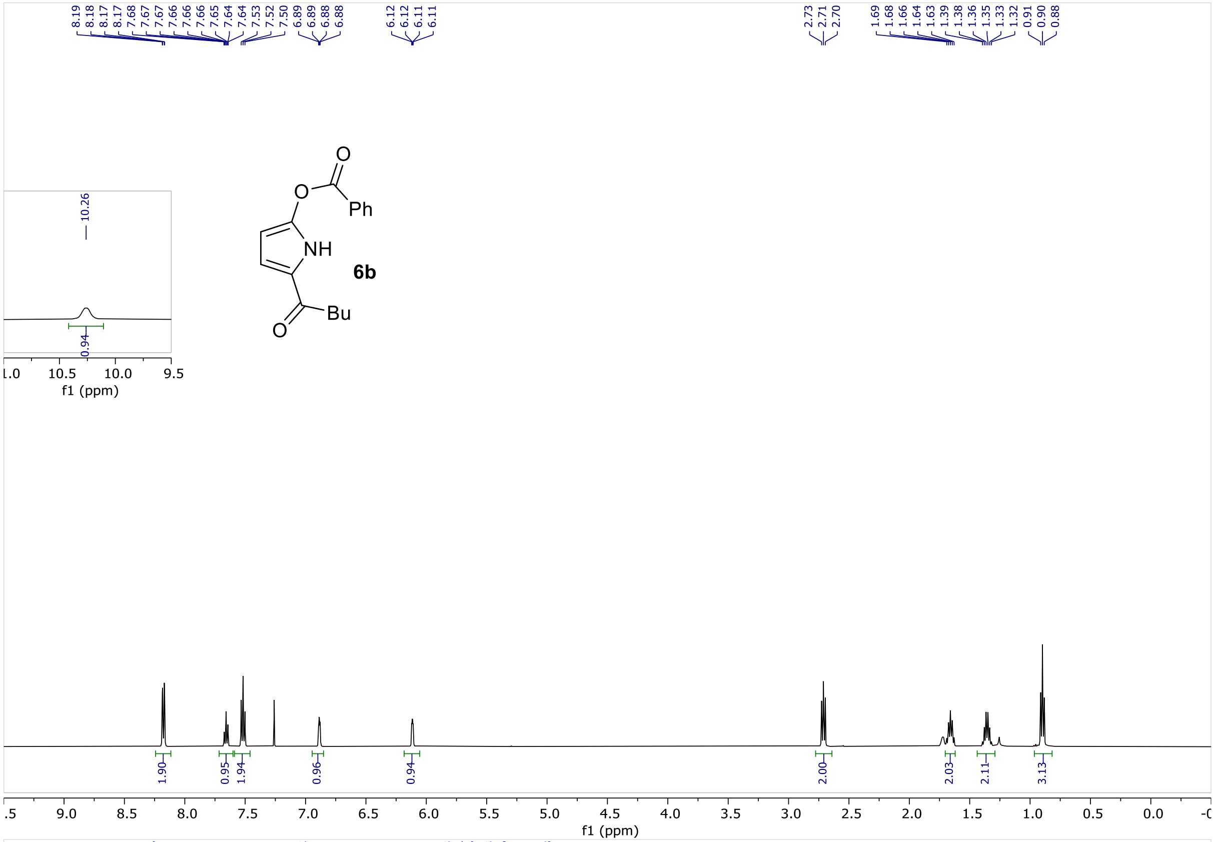
8.19
8.18
8.17
8.17
7.68
7.67
7.67
7.66
7.66
7.66
7.65
7.64
7.64
7.53
7.52
7.50
6.89
6.89
6.88
6.88
6.12
6.12
6.11
6.11
2.73
2.71
2.70
1.69
1.68
1.66
1.64
1.63
1.39
1.38
1.36
1.35
1.33
1.32
0.91
0.90
0.88
10.26
0.94
O
O
Ph
NH
6b
O
Bu
1.90
0.95
1.94
0.96
0.94
2.00
2.03
2.11
3.13
f1 (ppm)

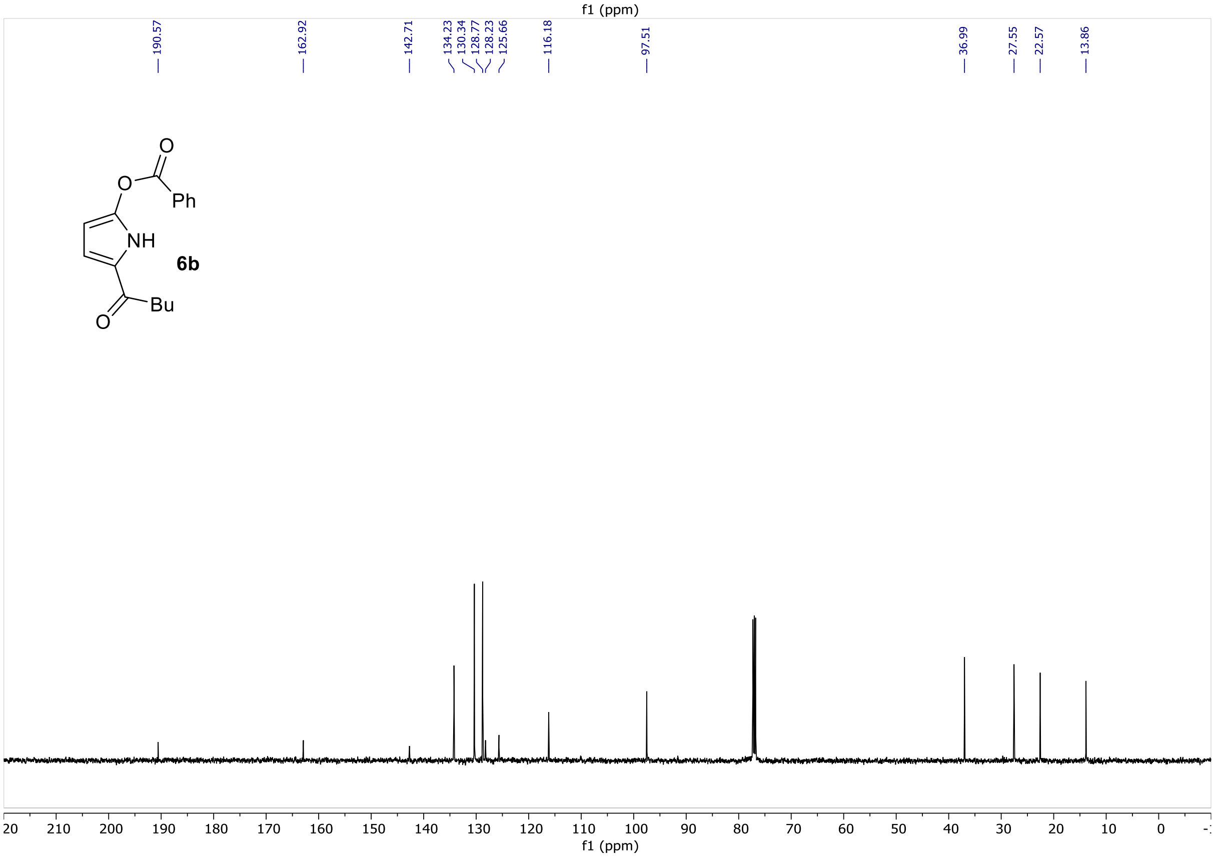
190.57
162.92
142.71
134.23
130.34
128.77
128.23
125.66
116.18
97.51
36.99
27.55
22.57
13.86
O
O
Ph
NH
6b
O
Bu
f1 (ppm)

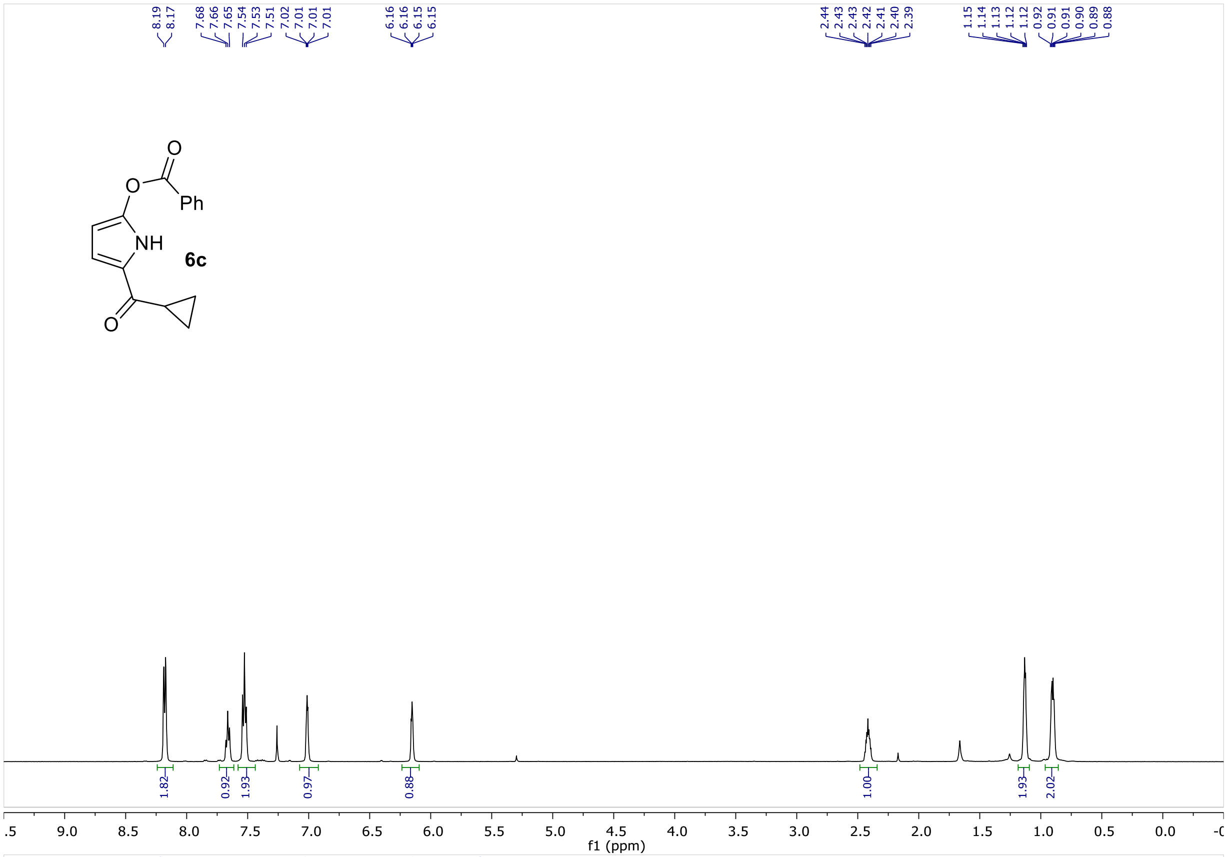
8.19
8.17
7.68
7.66
7.65
7.54
7.53
7.51
7.02
7.01
7.01
7.01
6.16
6.16
6.15
6.15
2.44
2.43
2.43
2.42
2.41
2.40
2.39
1.15
1.14
1.13
1.12
1.12
0.92
0.91
0.91
0.90
0.89
0.88
O
O
Ph
NH
6c
O
1.82
0.92
1.93
0.97
0.88
1.00
1.93
2.02
f1 (ppm)

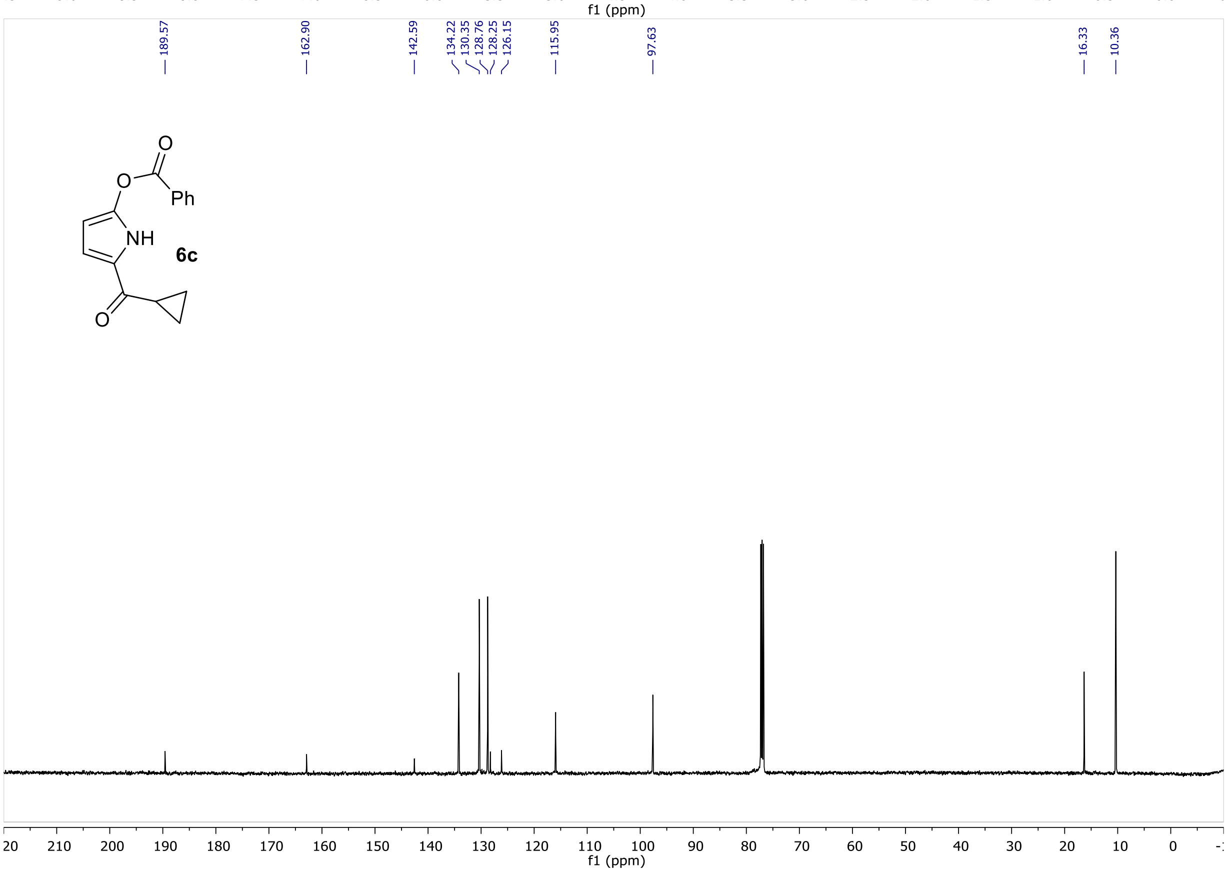
189.57
162.90
142.59
134.22
130.35
128.76
128.25
126.15
115.95
97.63
16.33
10.36
O
O
Ph
NH
6c
O
f1 (ppm)

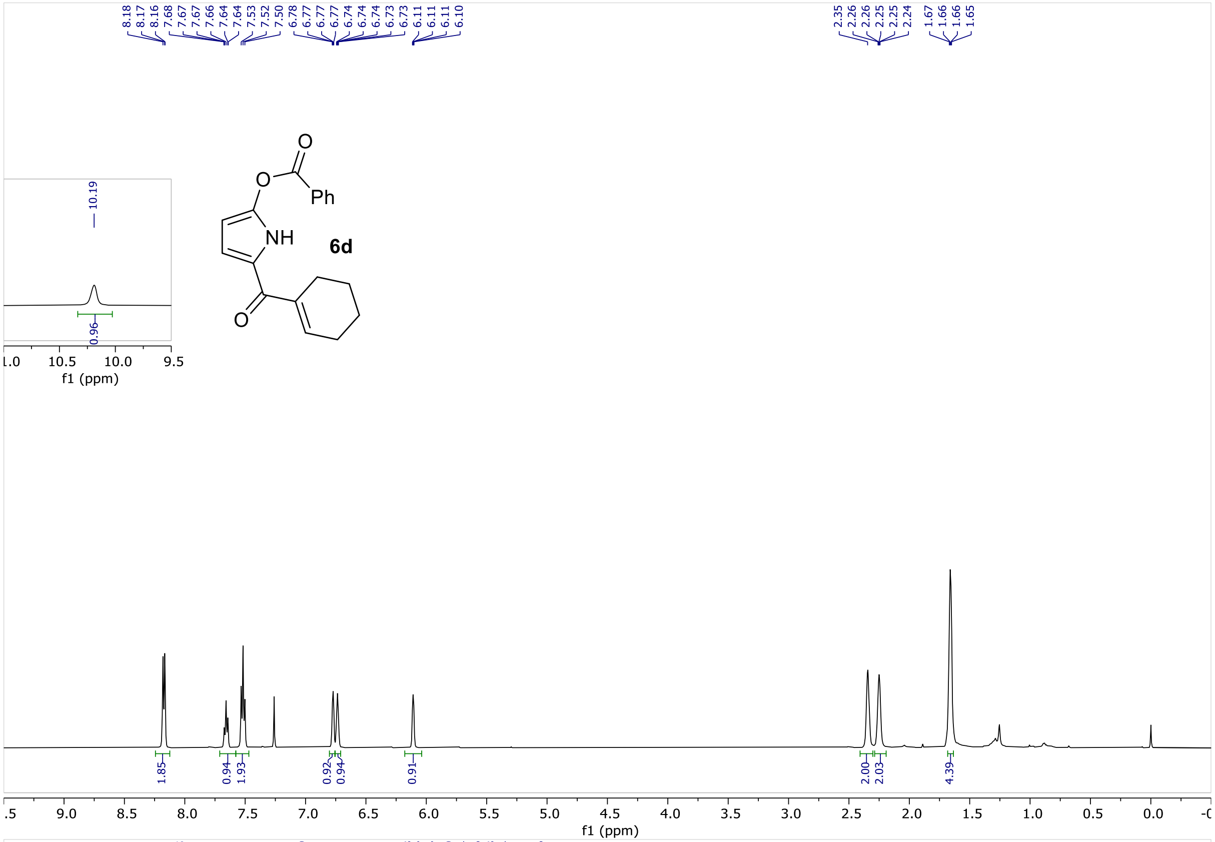

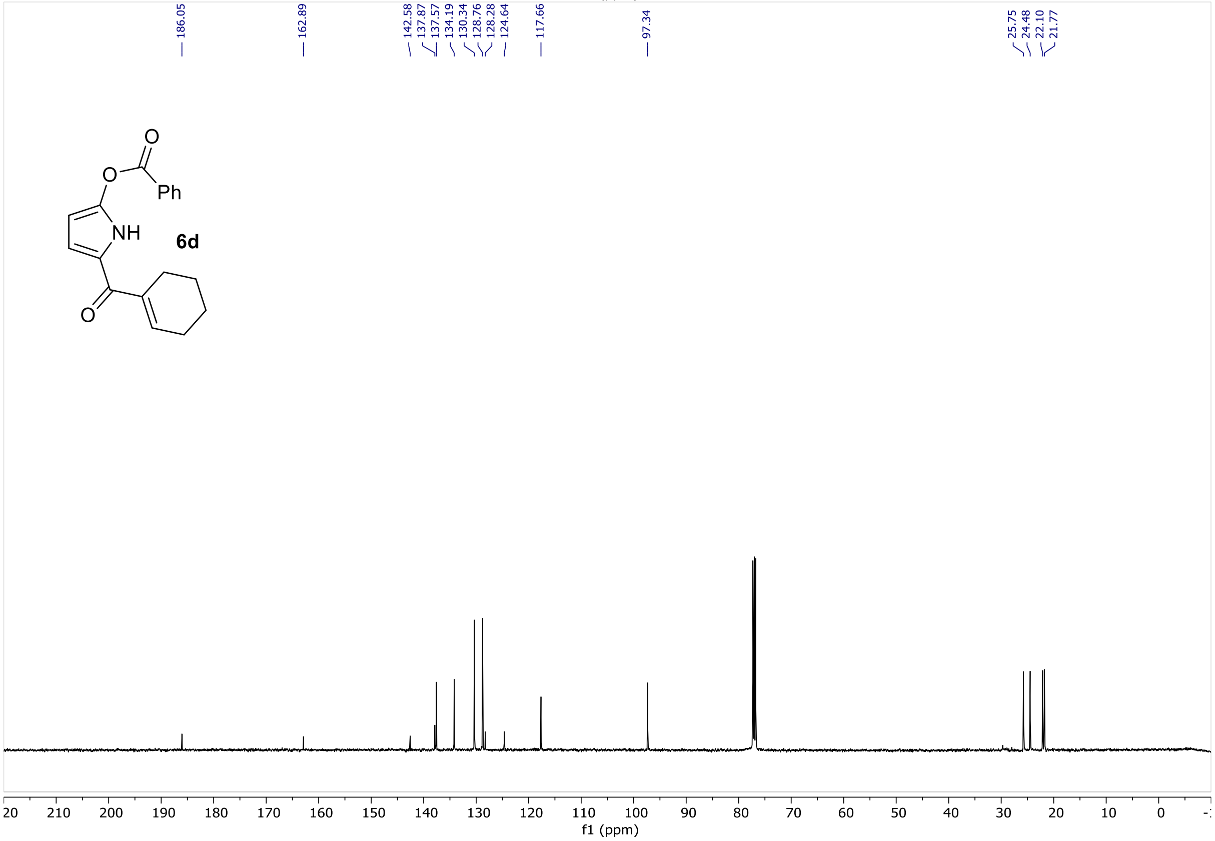

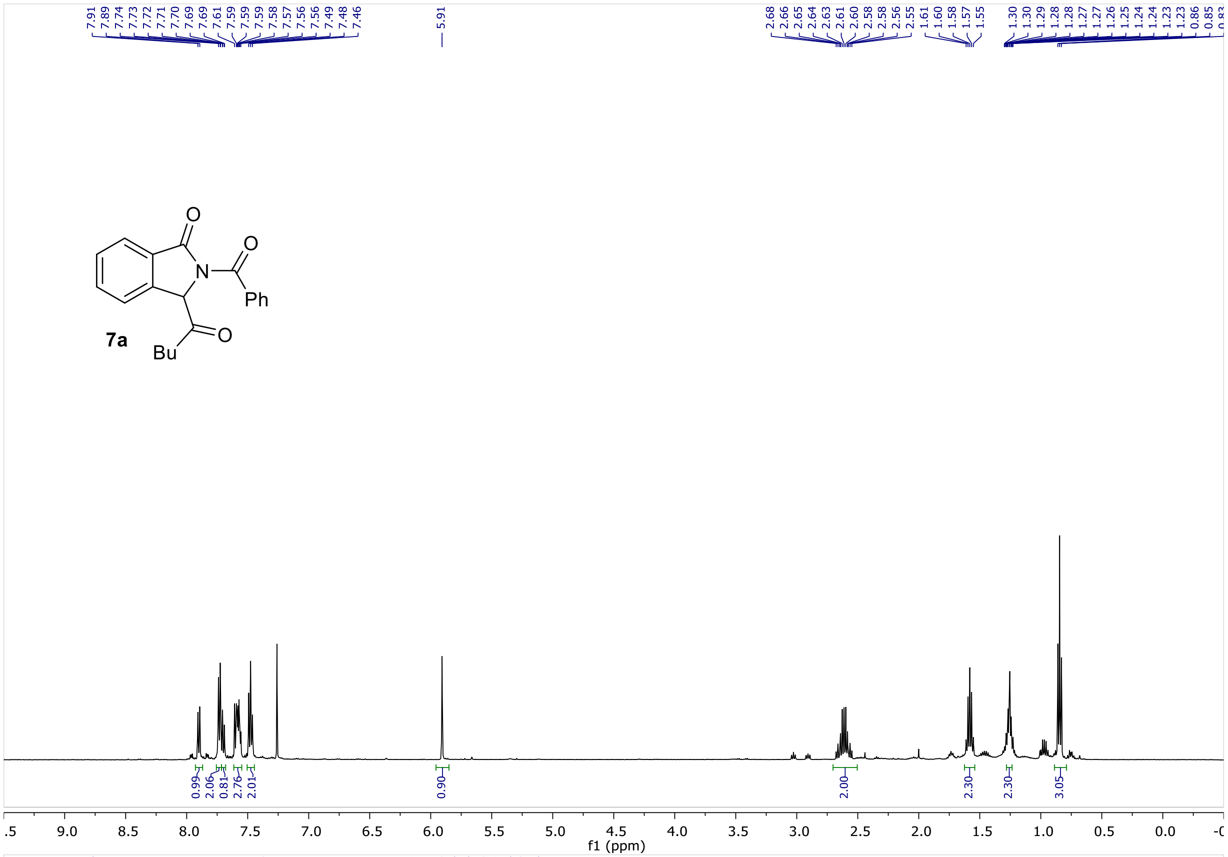

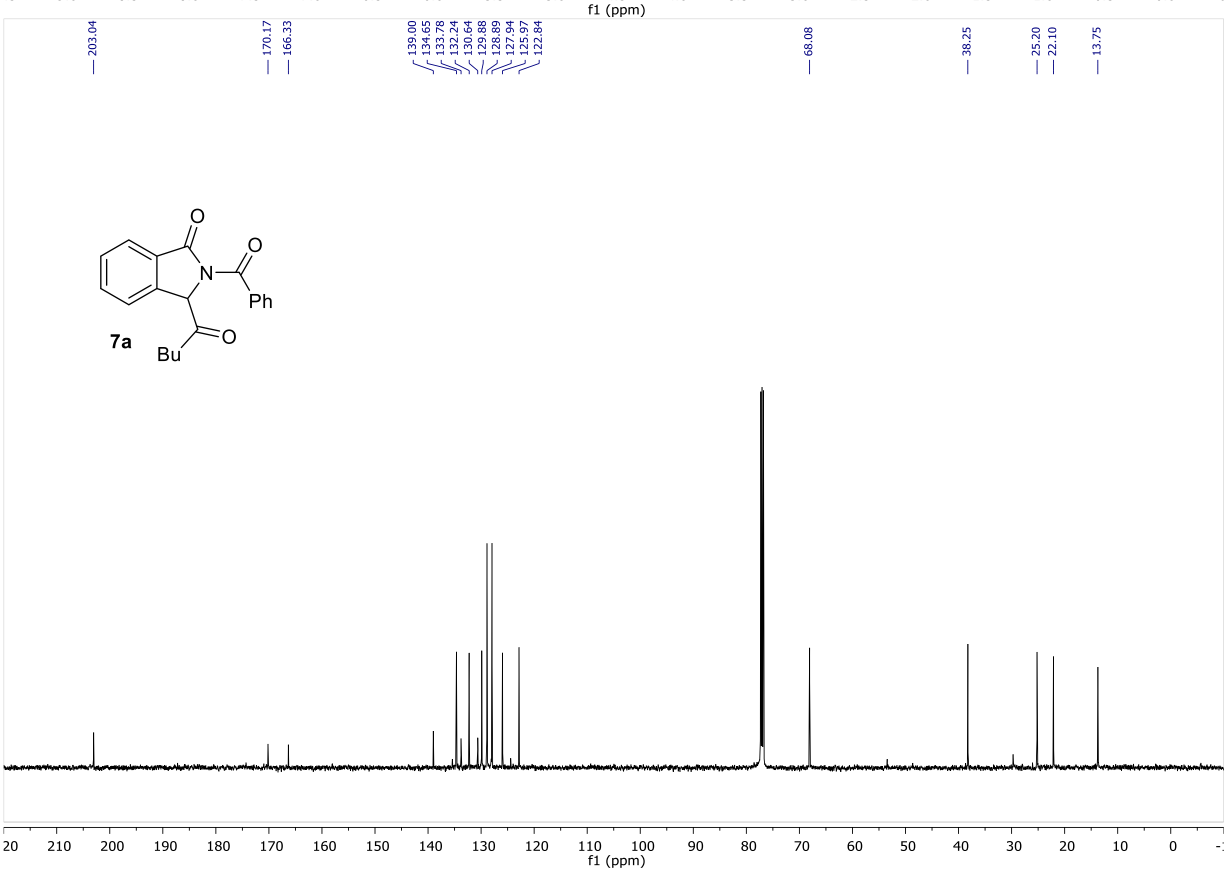

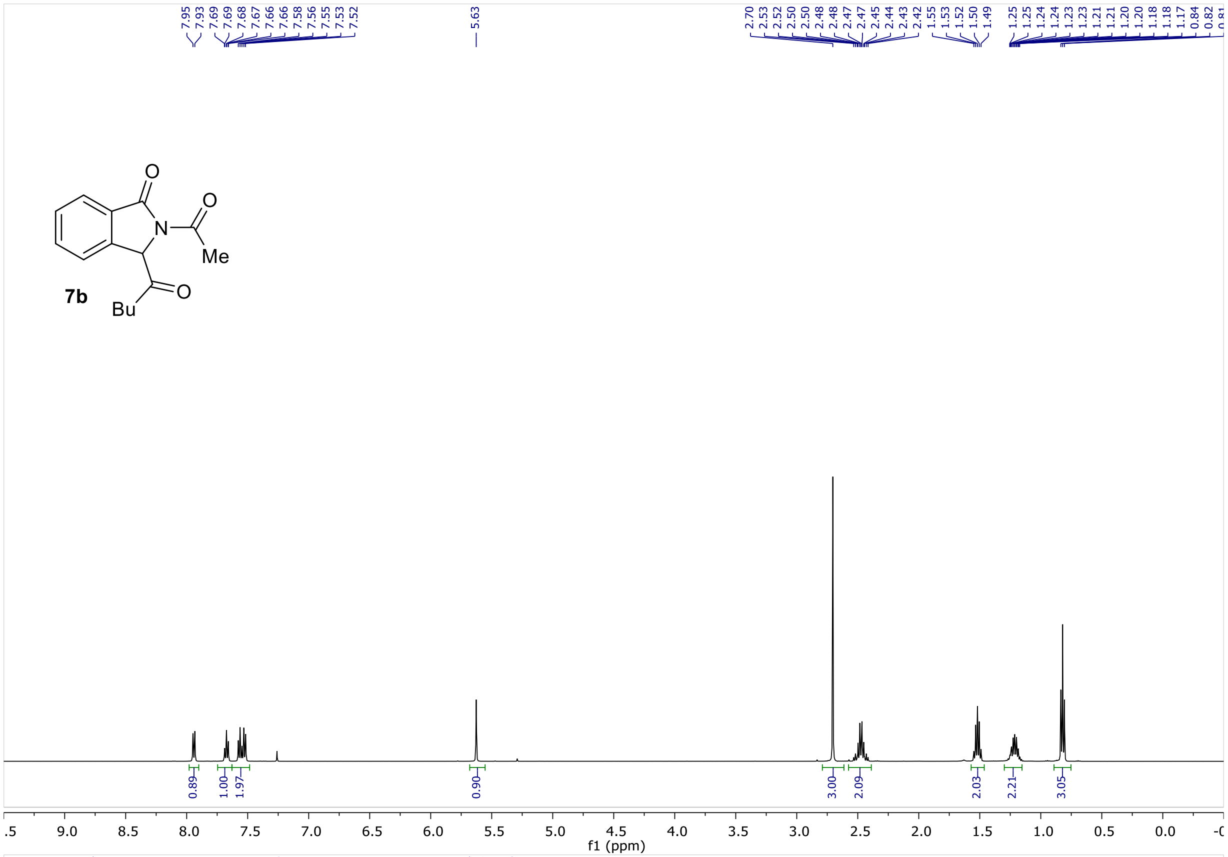

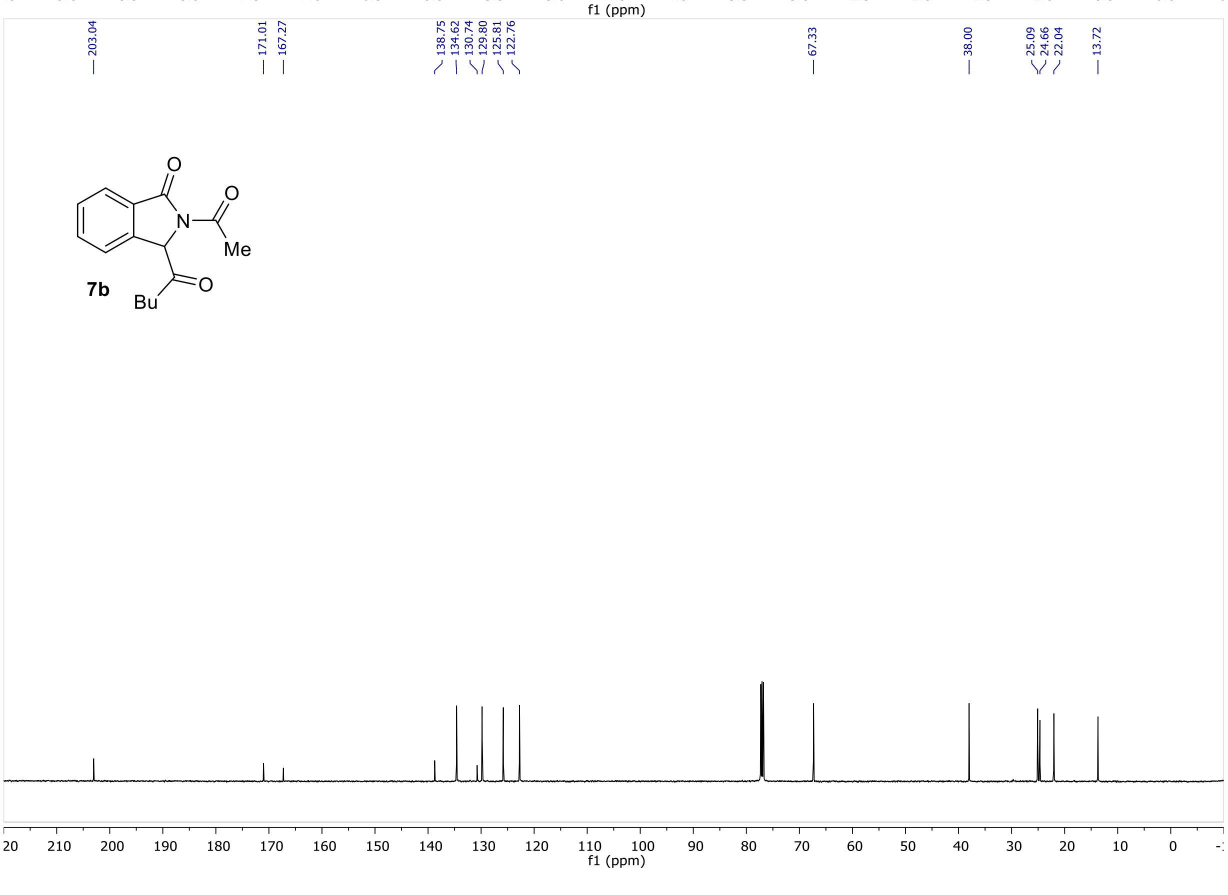

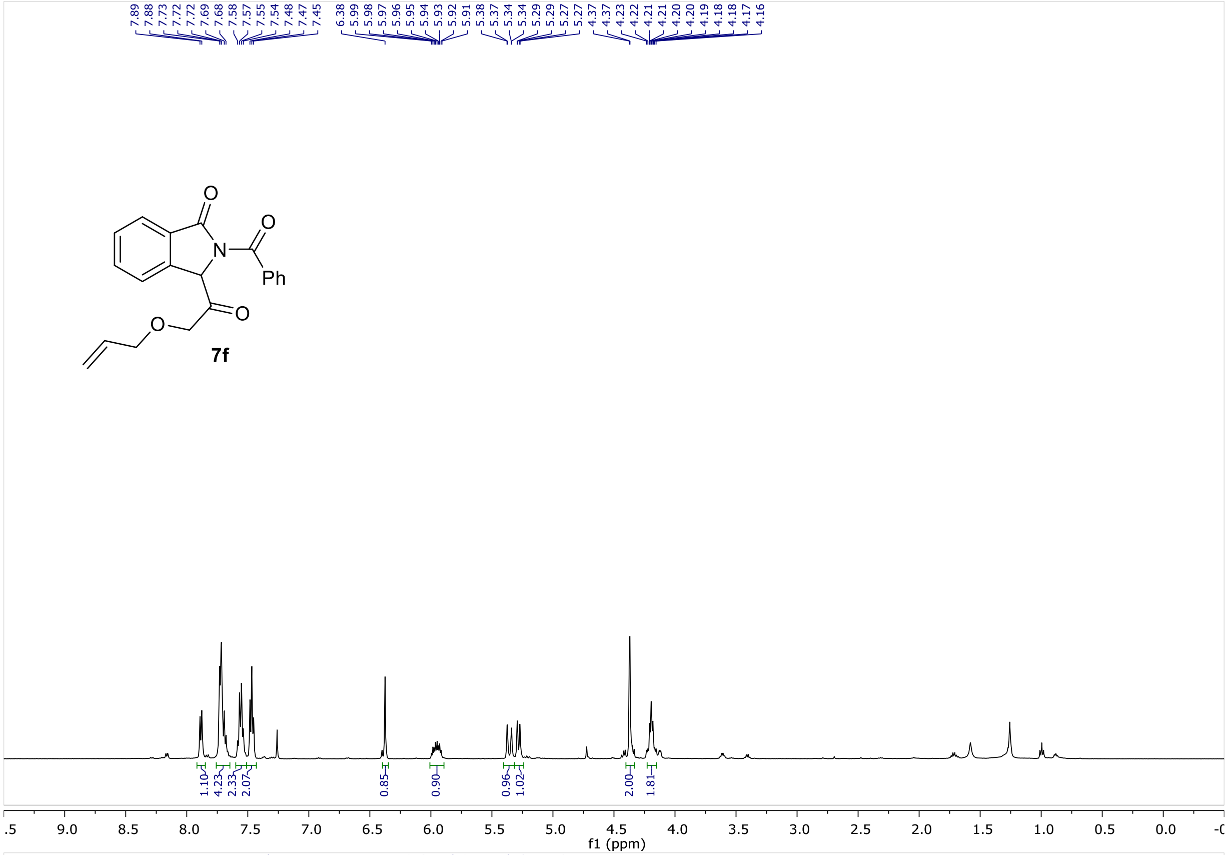

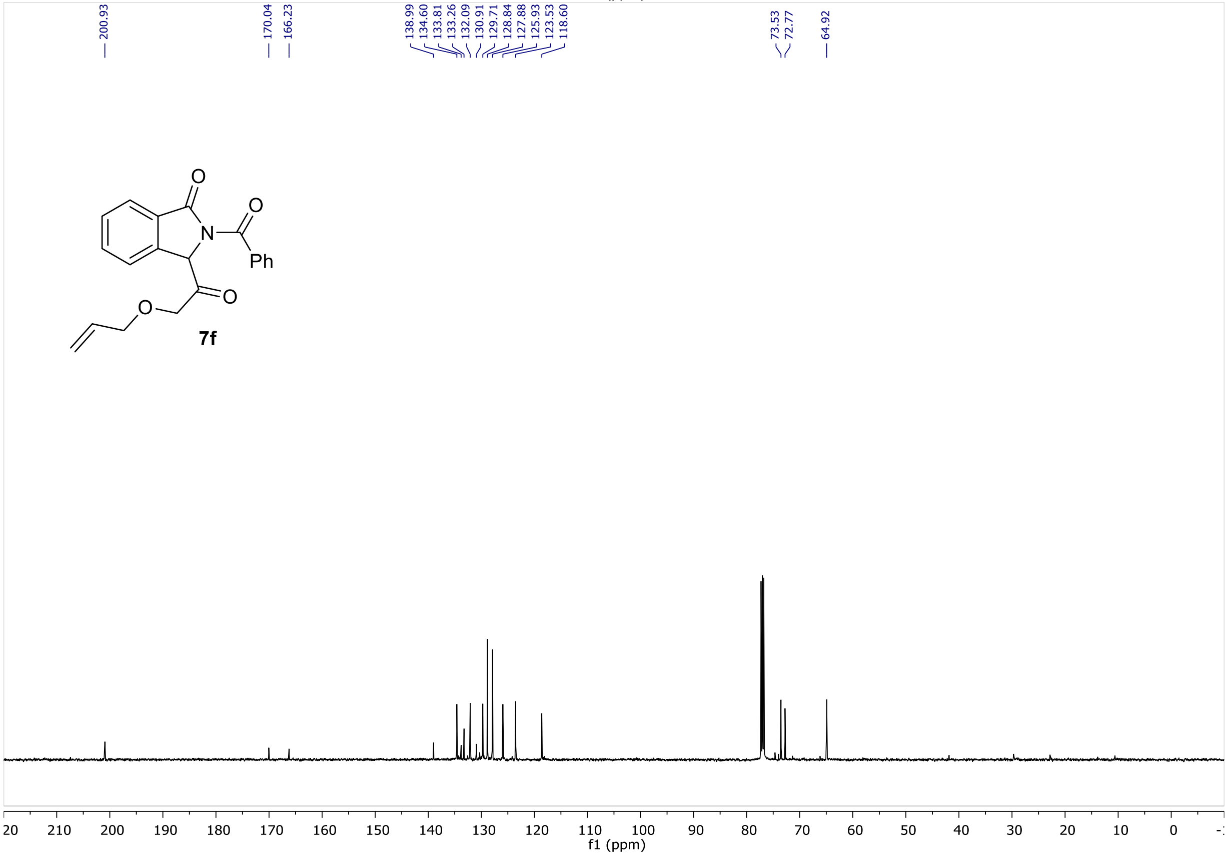

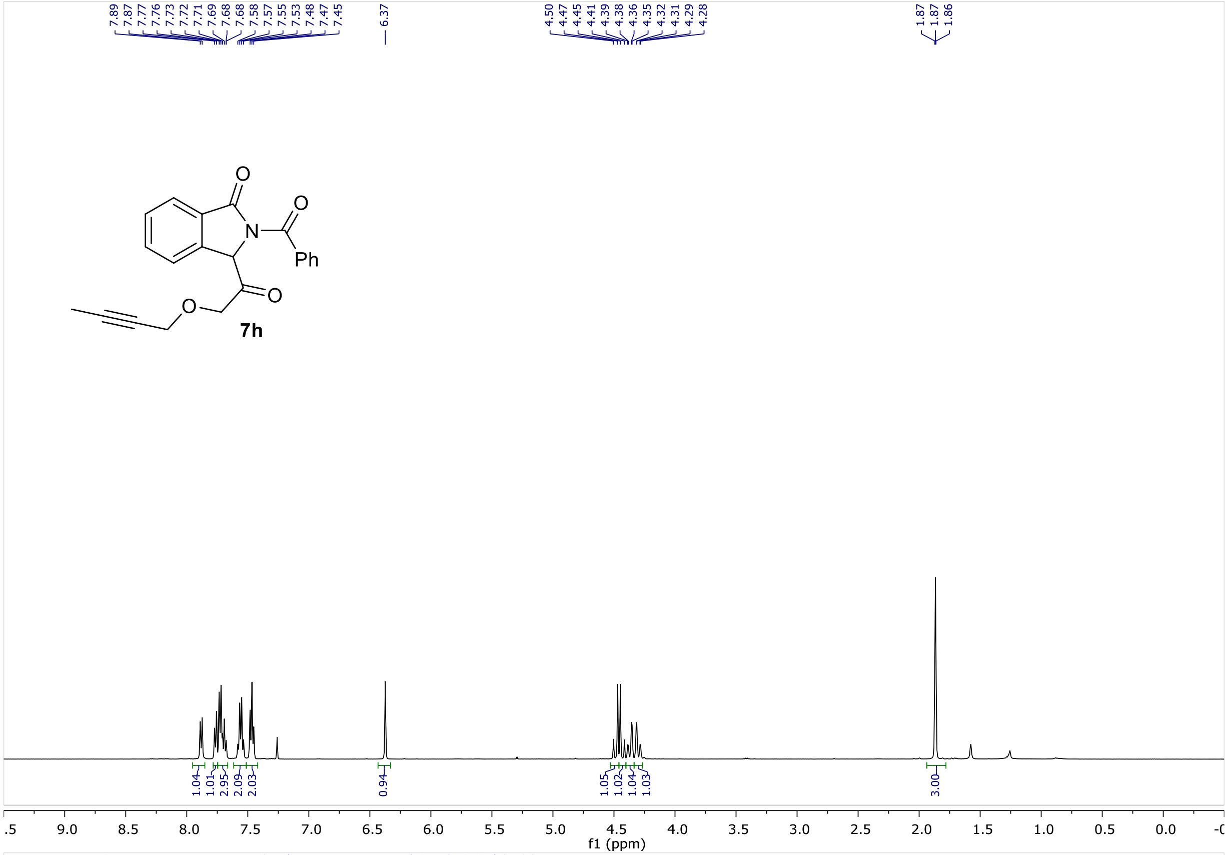
7.89
7.87
7.77
7.76
7.73
7.72
7.71
7.69
7.68
7.68
7.58
7.57
7.55
7.53
7.48
7.47
7.45
6.37
4.50
4.47
4.45
4.41
4.39
4.38
4.36
4.35
4.32
4.31
4.29
4.28
1.87
1.87
1.86
O
N
O
Ph
O
O
7h
1.04
1.01
2.95
2.09
2.03
0.94
1.05
1.02
1.04
1.03
3.00
f1 (ppm)

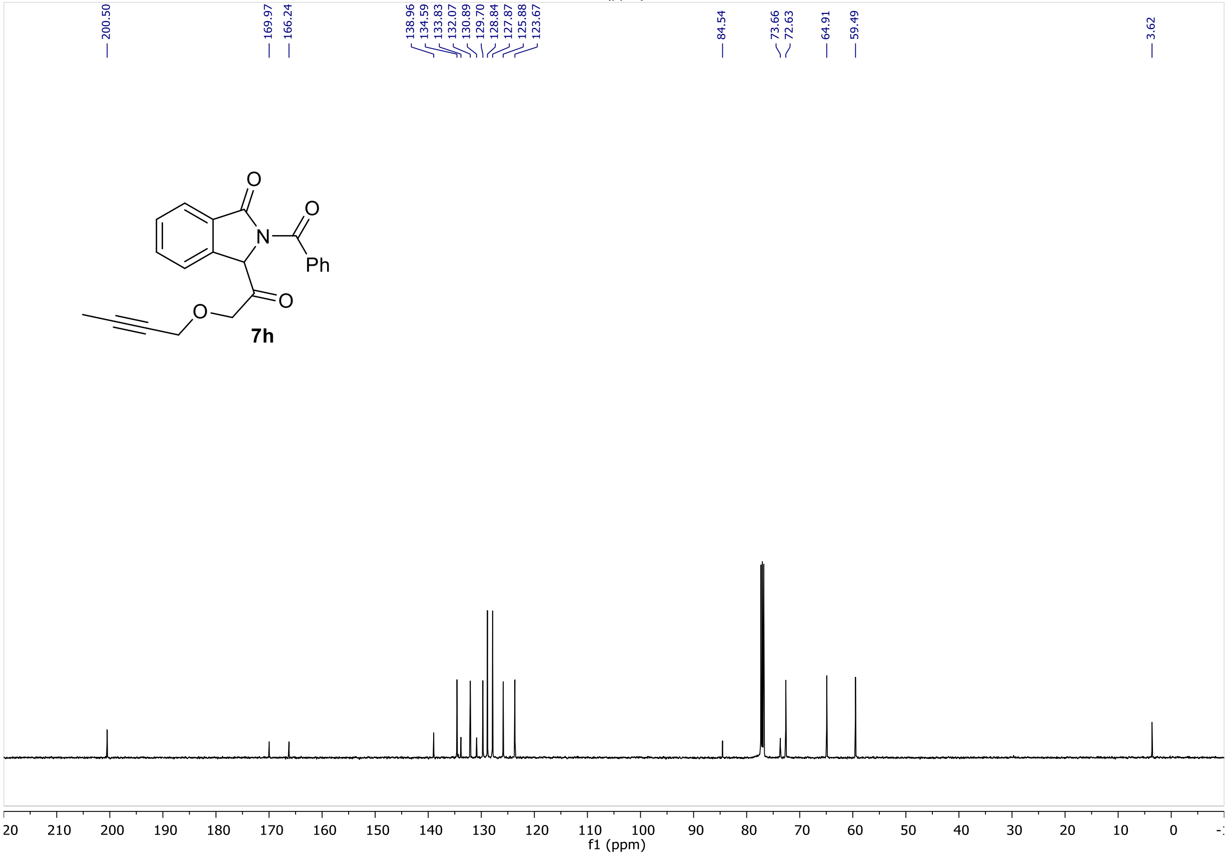
200.50
169.97
166.24
138.96
134.59
133.83
132.07
130.89
129.70
128.84
127.87
125.88
123.67
84.54
73.66
72.63
64.91
59.49
3.62
O
N
O
Ph
O
O
7h
f1 (ppm)

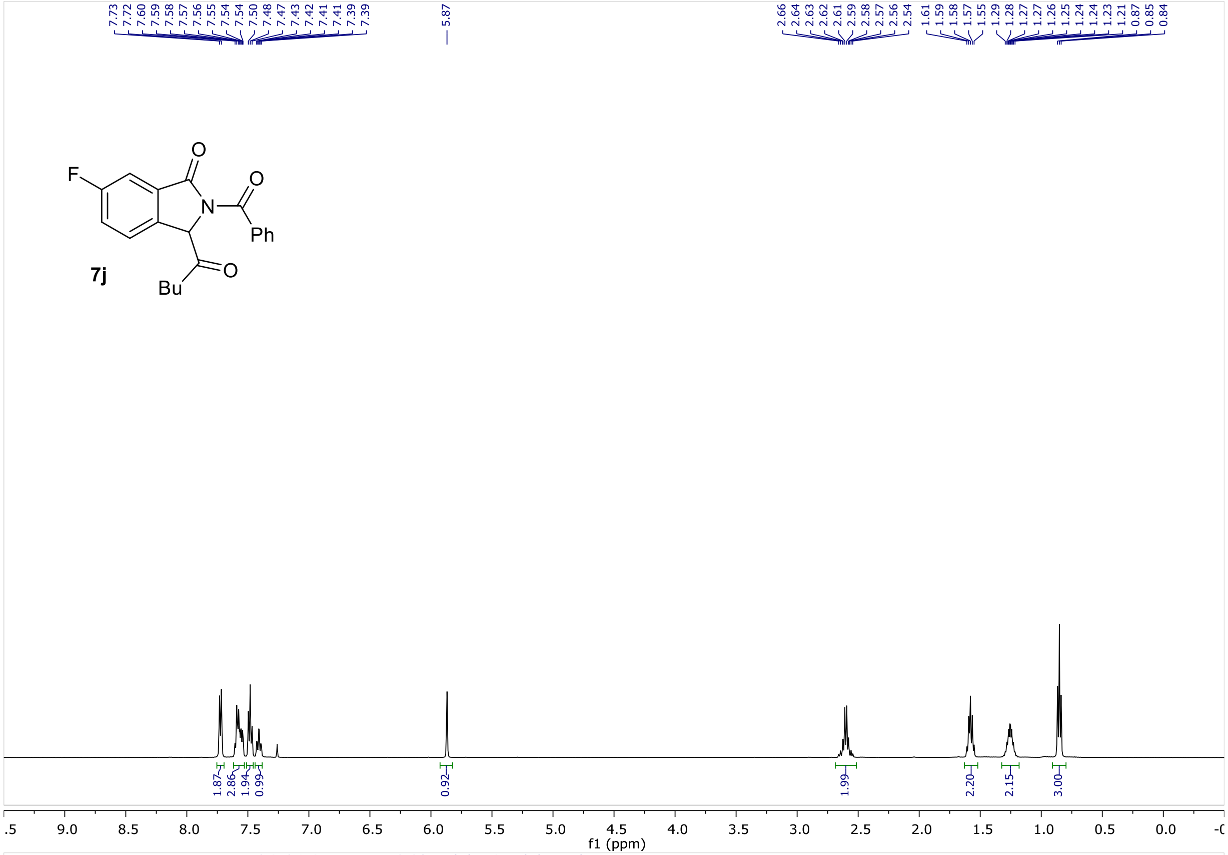

7.73
7.72
7.60
7.59
7.58
7.57
7.56
7.55
7.54
7.54
7.50
7.48
7.47
7.43
7.42
7.41
7.41
7.39
7.39
5.87
2.66
2.64
2.63
2.62
2.61
2.59
2.58
2.57
2.56
2.54
1.61
1.59
1.58
1.57
1.55
1.29
1.28
1.27
1.27
1.26
1.25
1.24
1.24
1.23
1.21
0.87
0.85
0.84
F
O
N
O
Ph
7j
Bu
O
1.87
2.86
1.94
0.99
0.92
1.99
2.20
2.15
3.00
f1 (ppm)

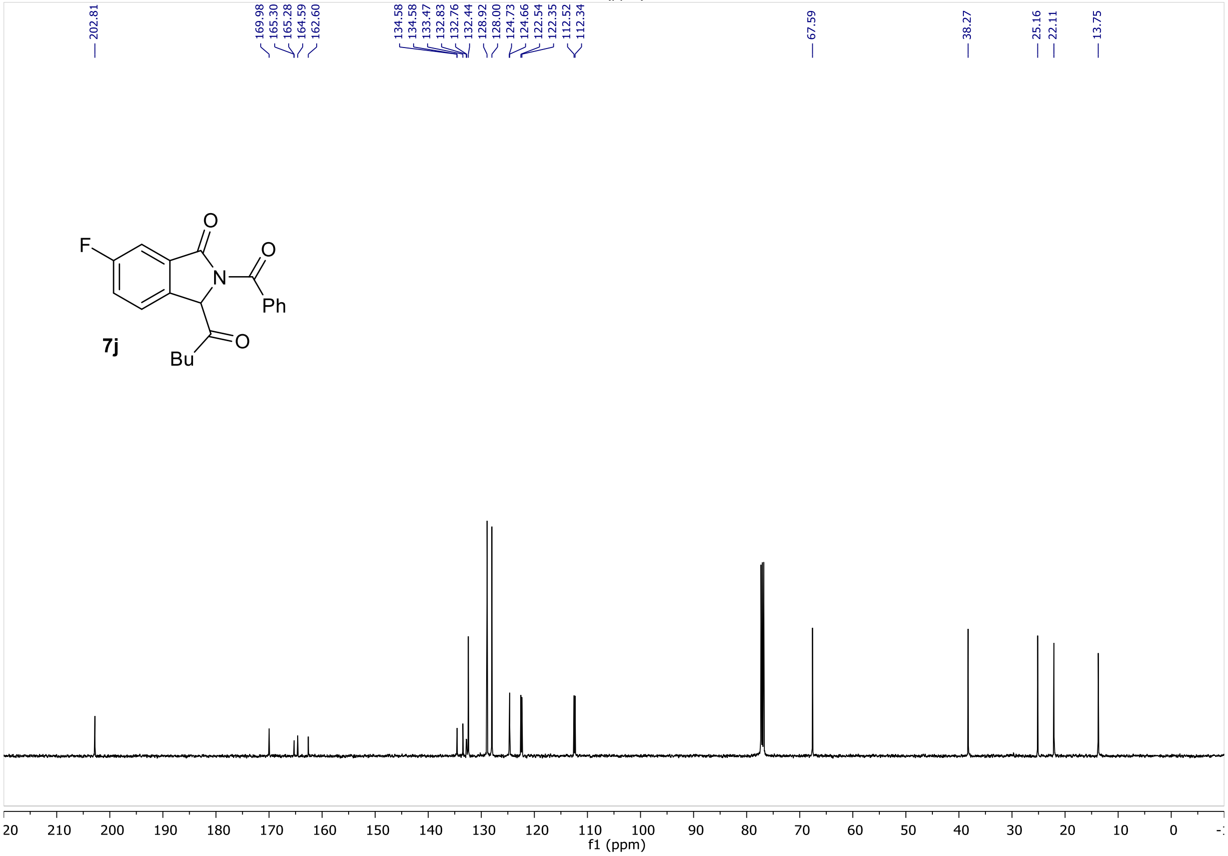

202.81
169.98
165.30
165.28
164.59
162.60
134.58
134.58
133.47
132.83
132.76
132.44
128.92
128.00
124.73
124.66
122.54
122.35
112.52
112.34
67.59
38.27
25.16
22.11
13.75
F
O
N
O
Ph
7j
Bu
O
f1 (ppm)

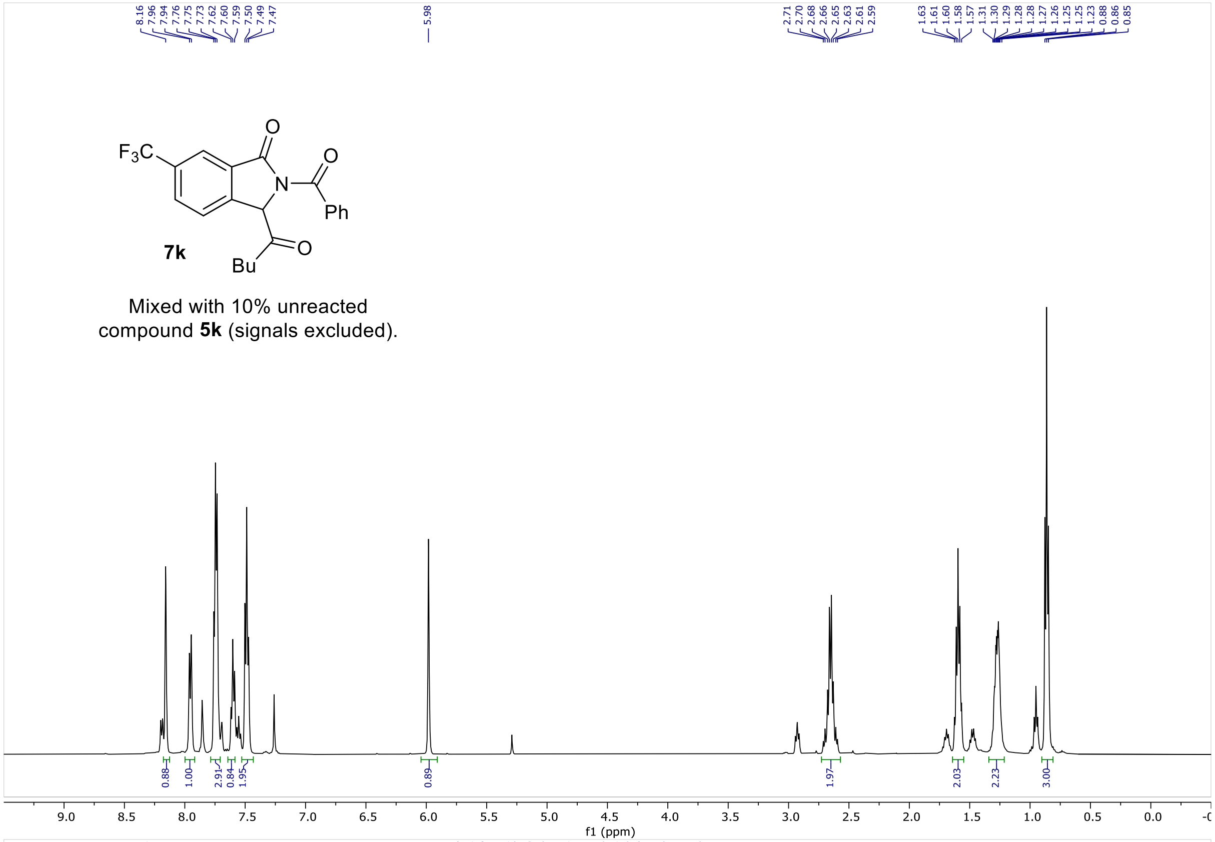
7k
Mixed with 10% unreacted compound 5k (signals excluded).
f1 (ppm)

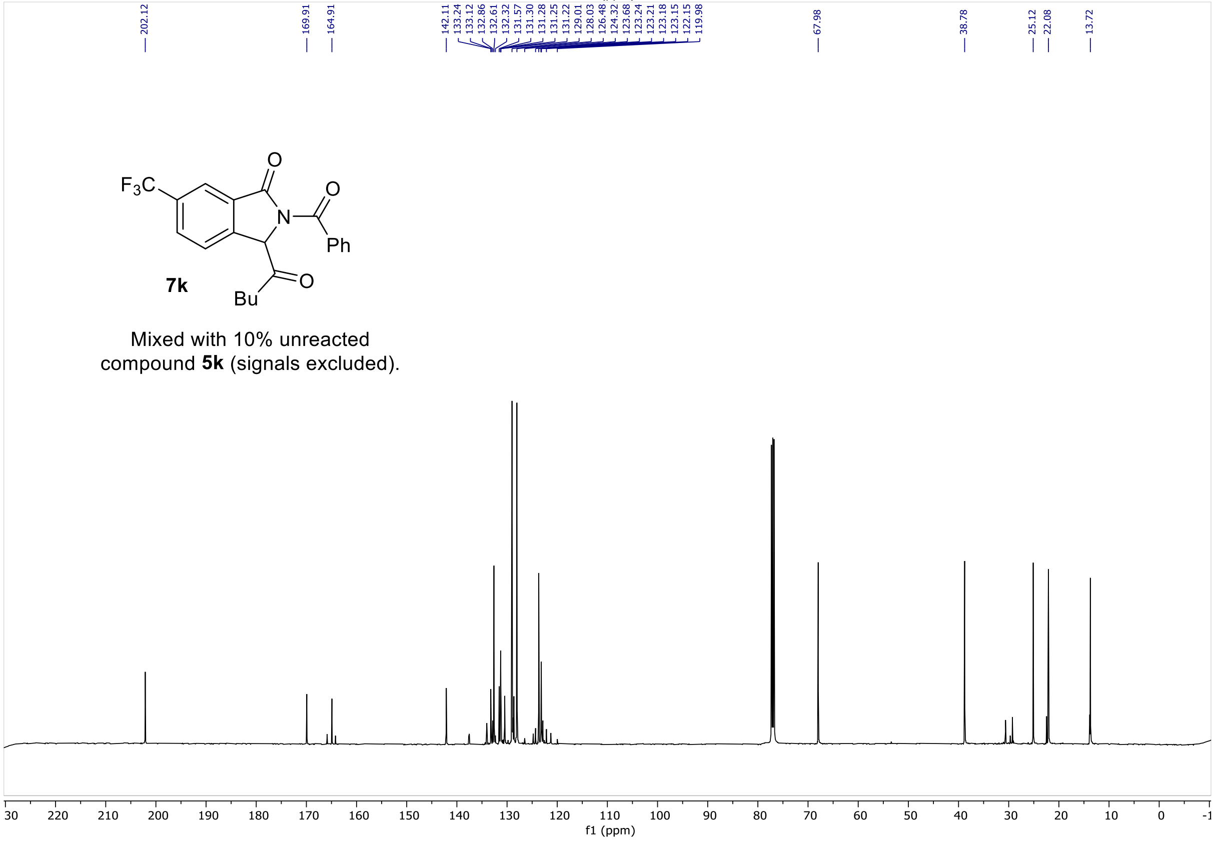
7k
Mixed with 10% unreacted compound 5k (signals excluded).
f1 (ppm)

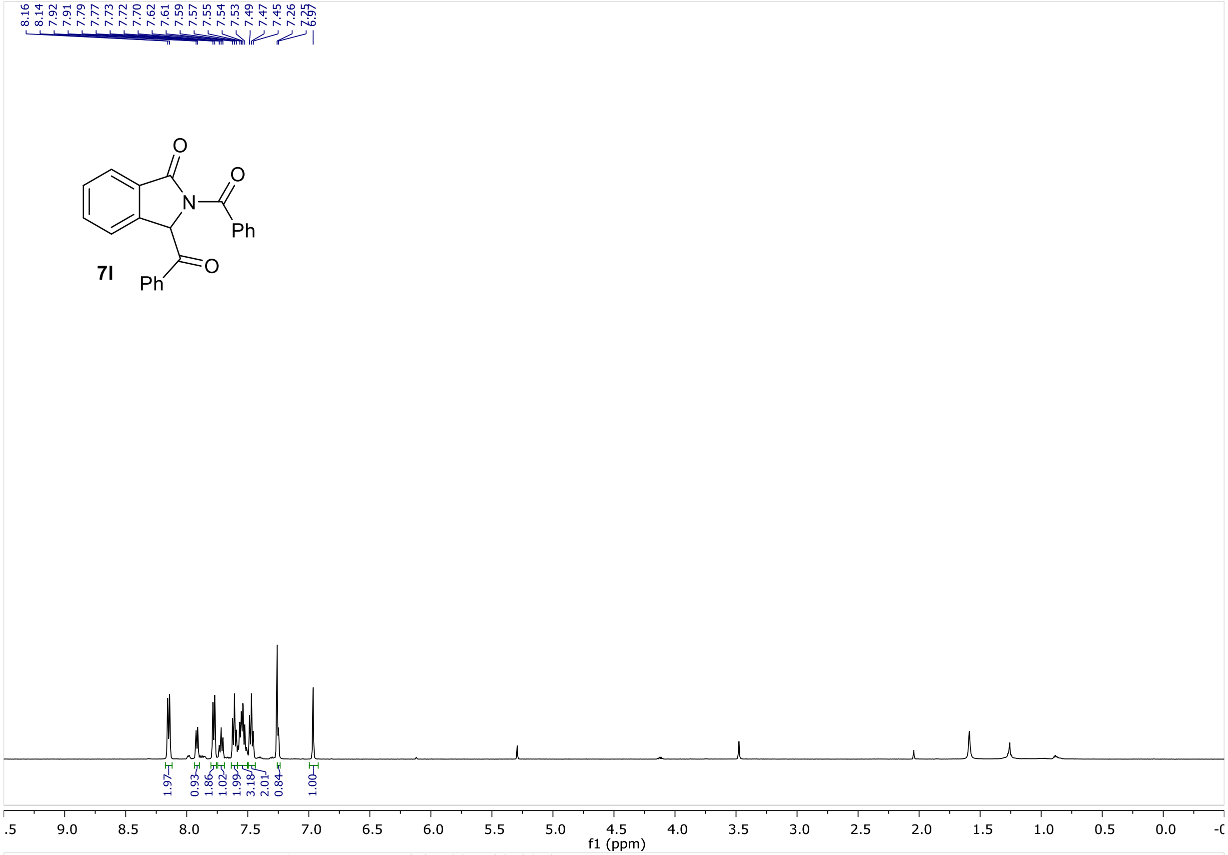

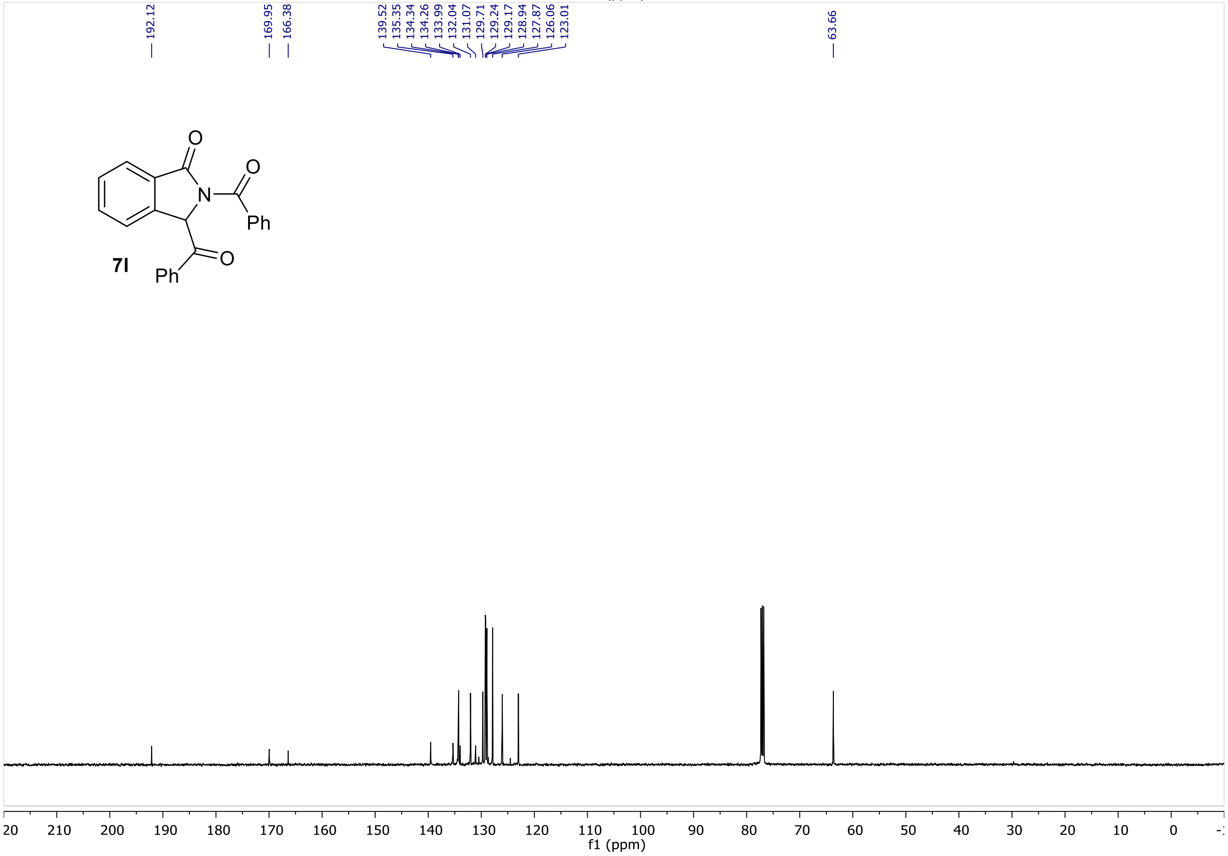

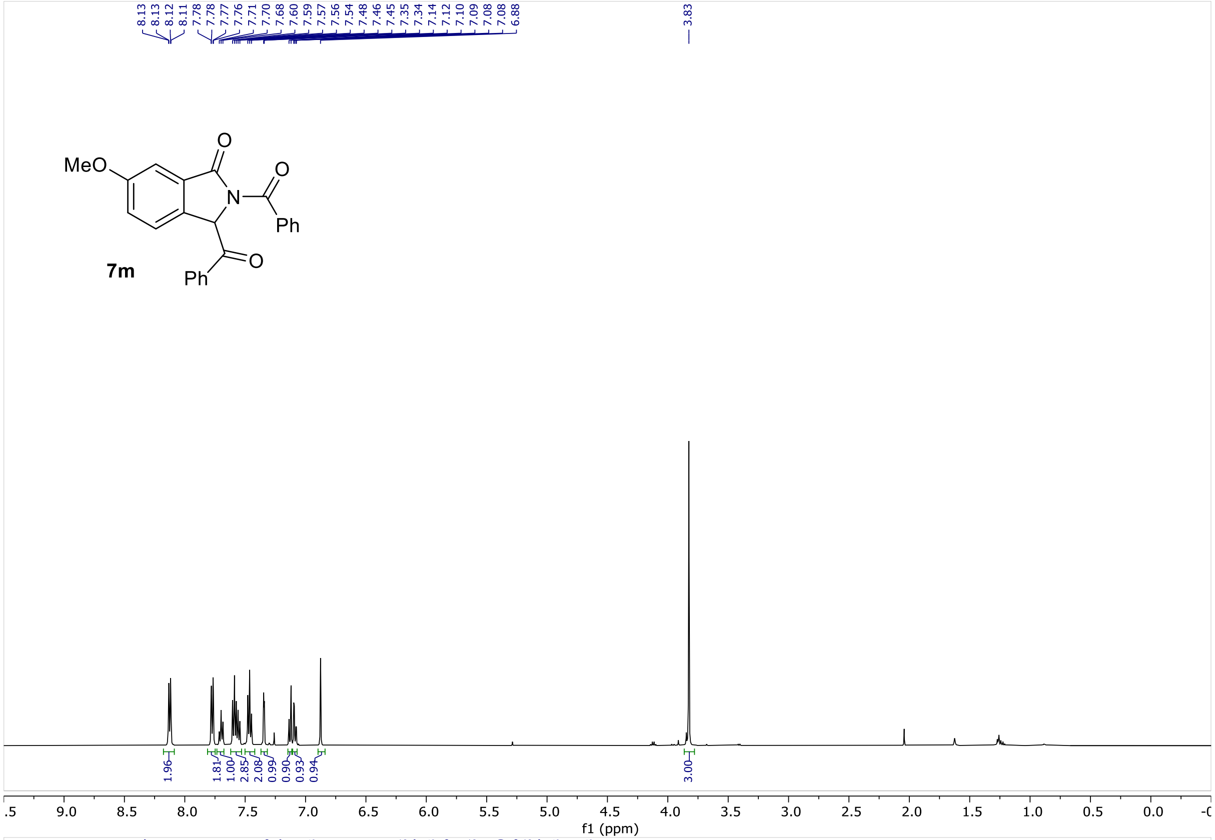
MeO
O
N
O
Ph
O
7m
Ph
8.13
8.13
8.12
8.11
7.78
7.78
7.77
7.76
7.71
7.70
7.68
7.60
7.59
7.57
7.56
7.54
7.48
7.46
7.45
7.35
7.34
7.14
7.12
7.10
7.09
7.08
7.08
6.88
3.83
1.96
1.81
1.00
2.85
2.08
0.99
0.90
0.93
0.94
3.00
f1 (ppm)

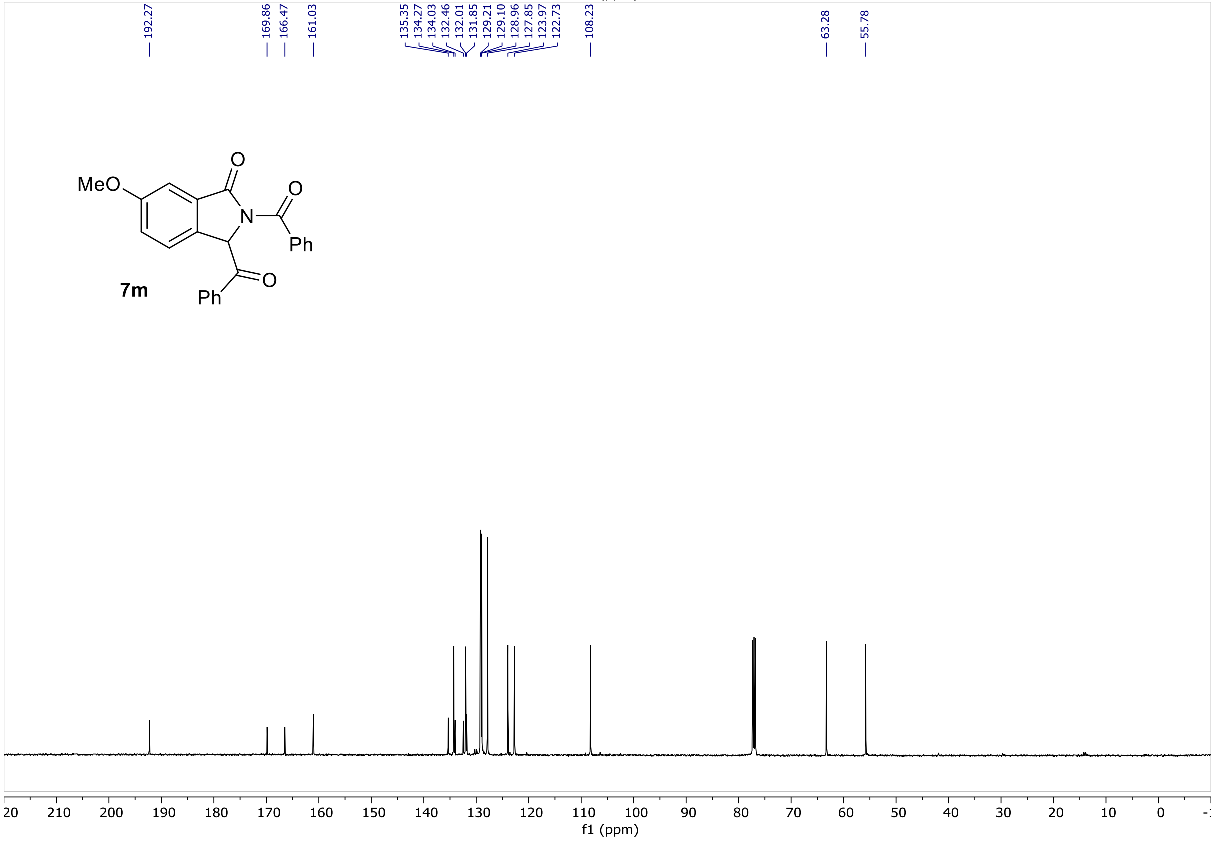
192.27
169.86
166.47
161.03
135.35
134.27
134.03
132.46
132.01
131.85
129.21
129.10
128.96
127.85
123.97
122.73
108.23
63.28
55.78
MeO
O
N
O
Ph
O
7m
Ph
f1 (ppm)

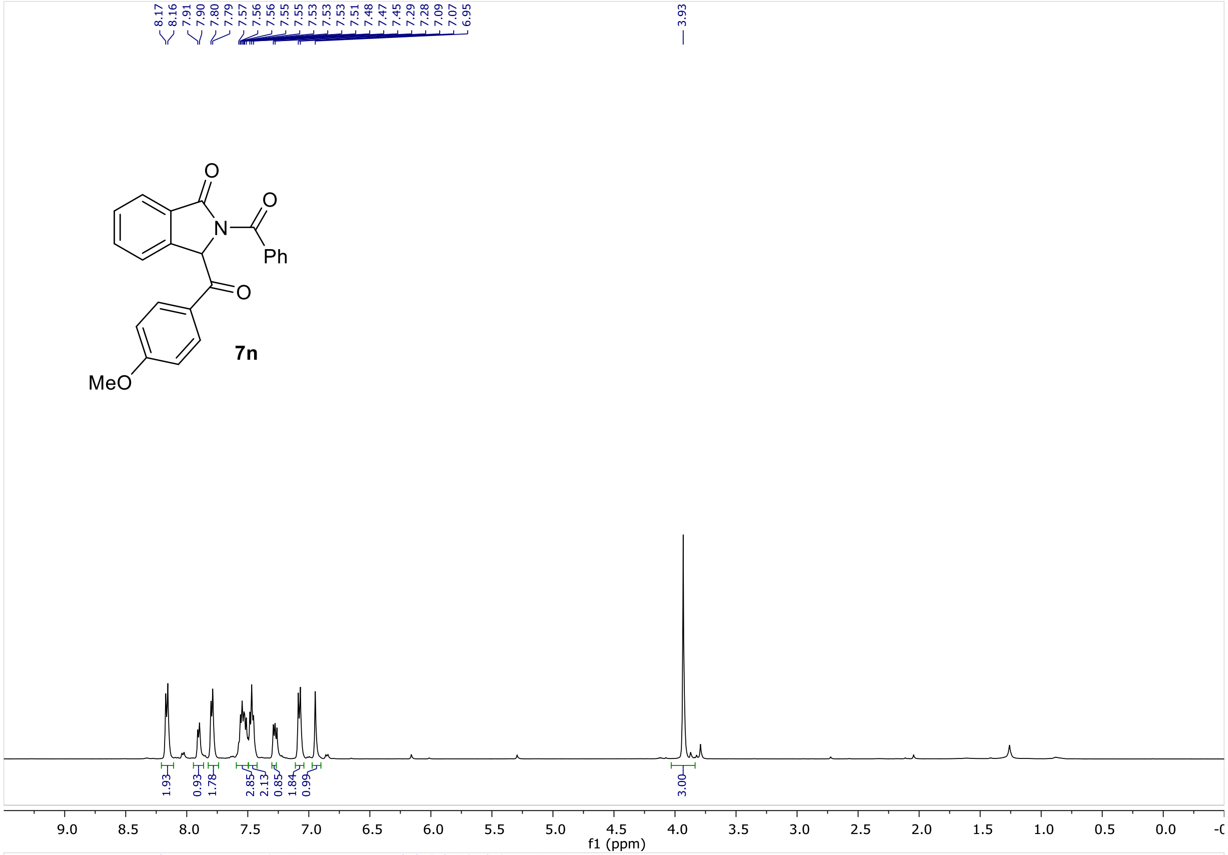
8.17
8.16
7.91
7.90
7.80
7.79
7.57
7.56
7.56
7.55
7.55
7.53
7.53
7.53
7.51
7.48
7.47
7.45
7.29
7.28
7.09
7.07
6.95
3.93
O
O
N
Ph
O
MeO
7n
1.93
0.93
1.78
2.85
2.13
0.85
1.84
0.99
3.00
f1 (ppm)

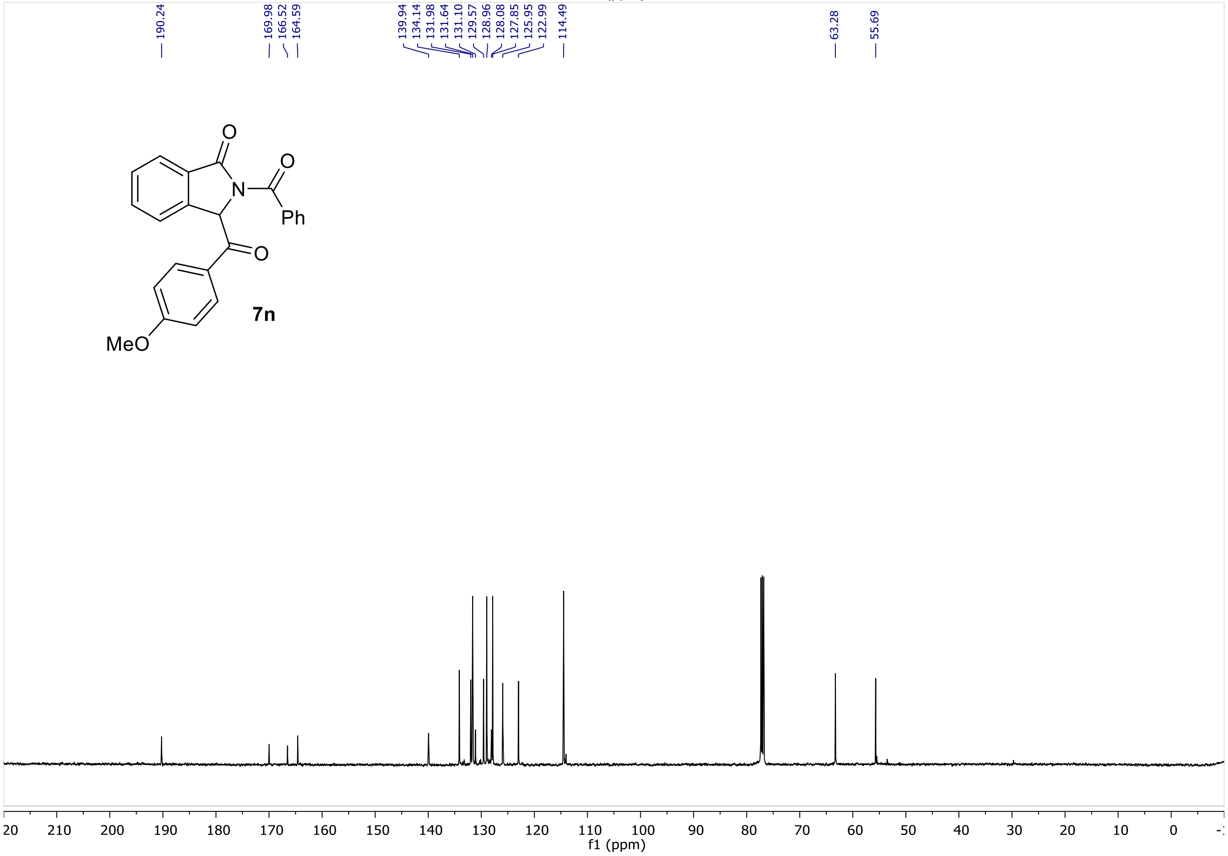
190.24
169.98
166.52
164.59
139.94
134.14
131.98
131.64
131.10
129.57
128.96
128.08
127.85
125.95
122.99
114.49
63.28
55.69
O
O
N
Ph
O
MeO
7n
f1 (ppm)

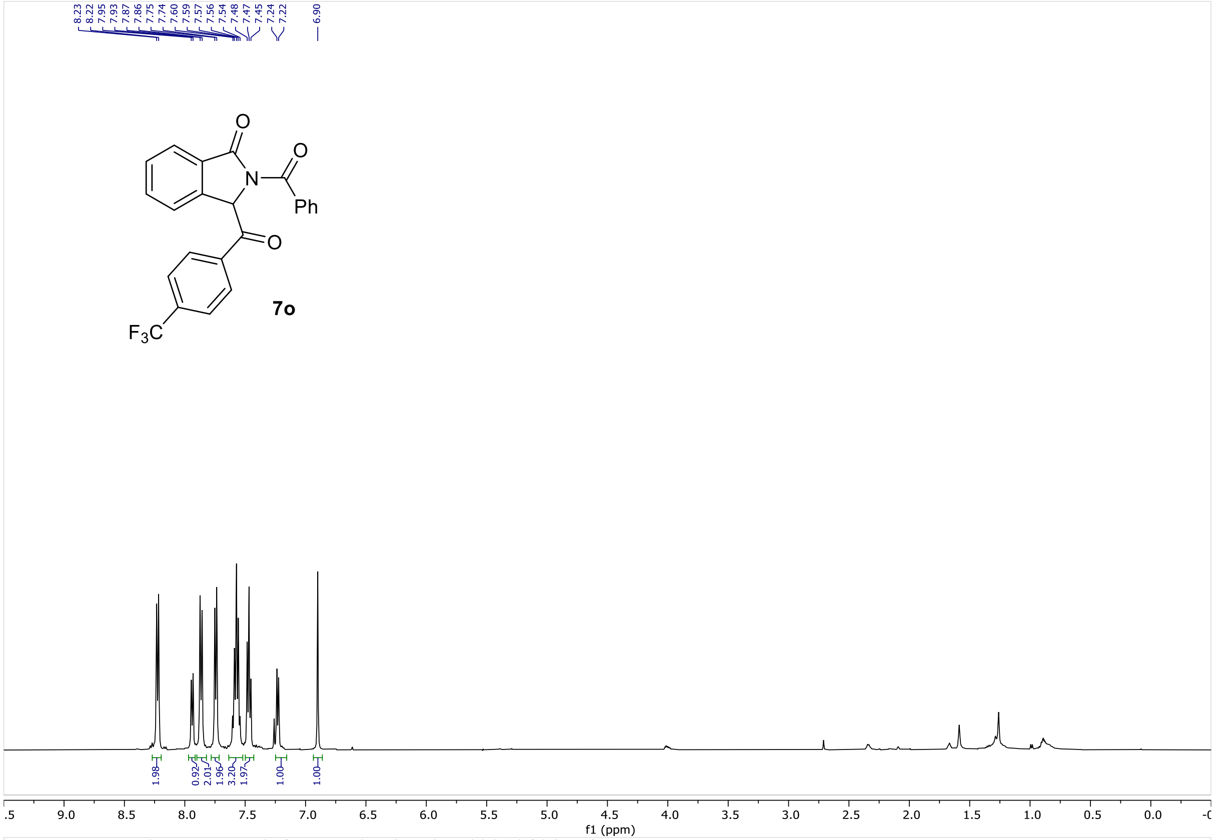
8.23
8.22
7.95
7.93
7.87
7.86
7.75
7.74
7.60
7.59
7.57
7.56
7.54
7.48
7.47
7.45
7.24
7.22
6.90
O
N
O
Ph
O
F3C
7o
1.98
0.92
2.01
1.96
3.20
1.97
1.00
1.00
9.0 8.5 8.0 7.5 7.0 6.5 6.0 5.5 5.0 4.5 4.0 3.5 3.0 2.5 2.0 1.5 1.0 0.5 0.0
f1 (ppm)

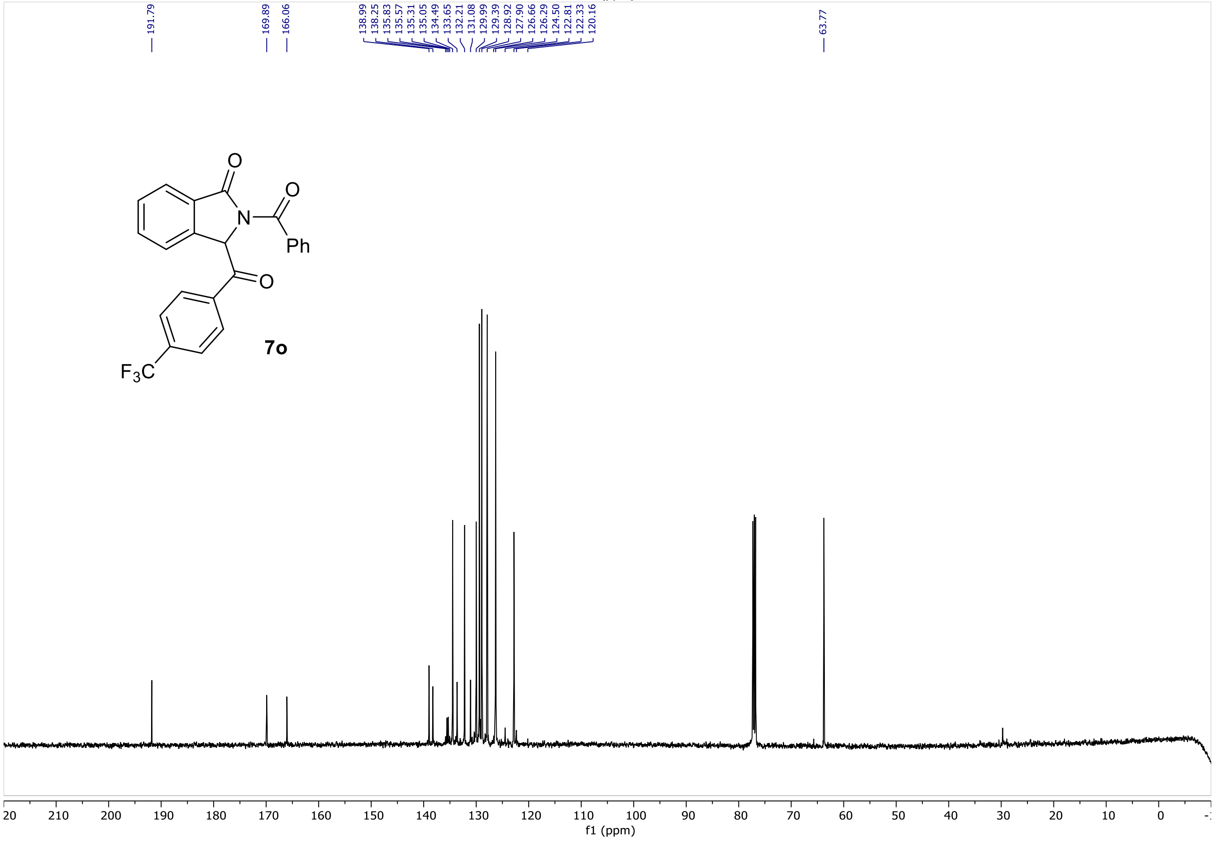
191.79
169.89
166.06
138.99
138.25
135.83
135.57
135.31
135.05
134.49
133.65
132.21
131.08
129.99
129.39
128.92
127.90
126.66
126.29
124.50
122.81
122.33
120.16
63.77
O
N
O
Ph
O
F3C
7o
210 200 190 180 170 160 150 140 130 120 110 100 90 80 70 60 50 40 30 20 10 0
f1 (ppm)

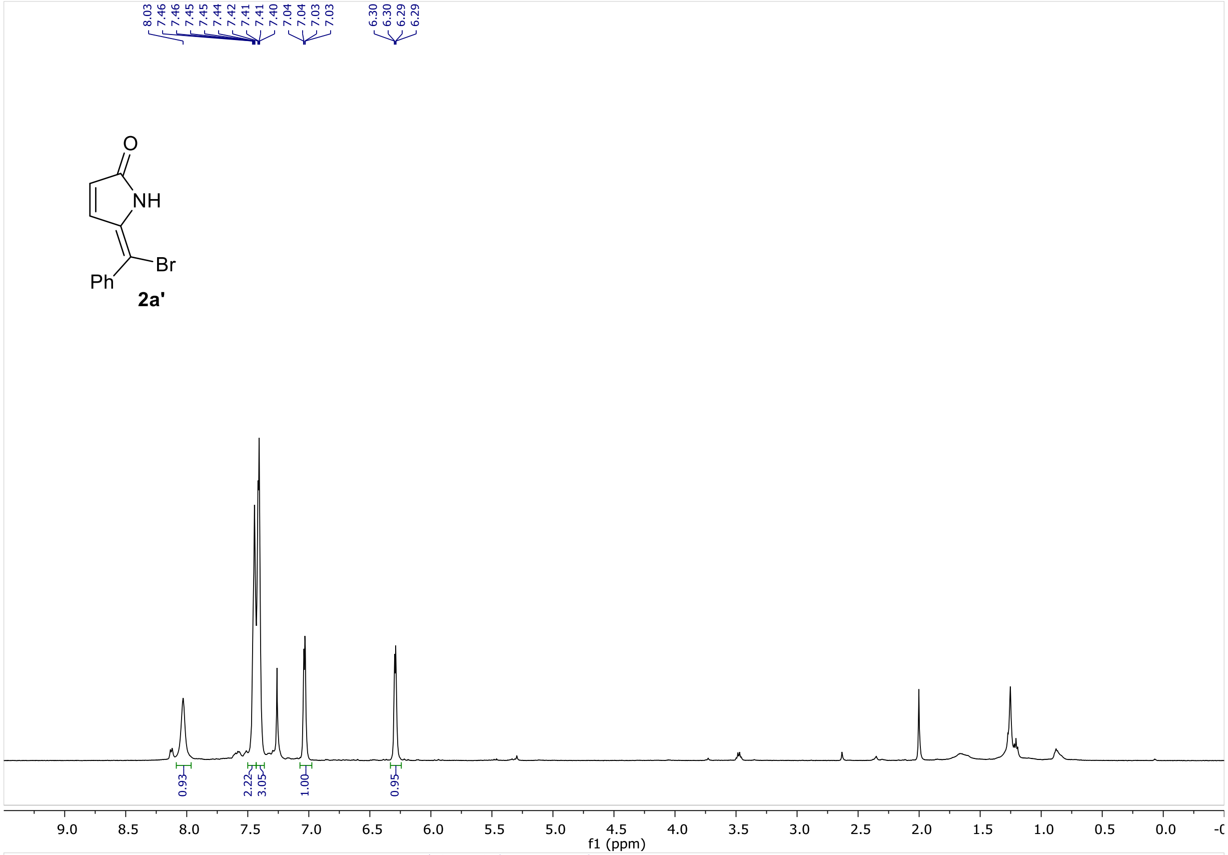
8.03
7.46
7.46
7.45
7.45
7.44
7.42
7.41
7.41
7.40
7.04
7.04
7.03
7.03
6.30
6.30
6.29
6.29
O
NH
Br
Ph
2a'
0.93
2.22
3.05
1.00
0.95
9.0
8.5
8.0
7.5
7.0
6.5
6.0
5.5
5.0
4.5
4.0
3.5
3.0
2.5
2.0
1.5
1.0
0.5
0.0
f1 (ppm)

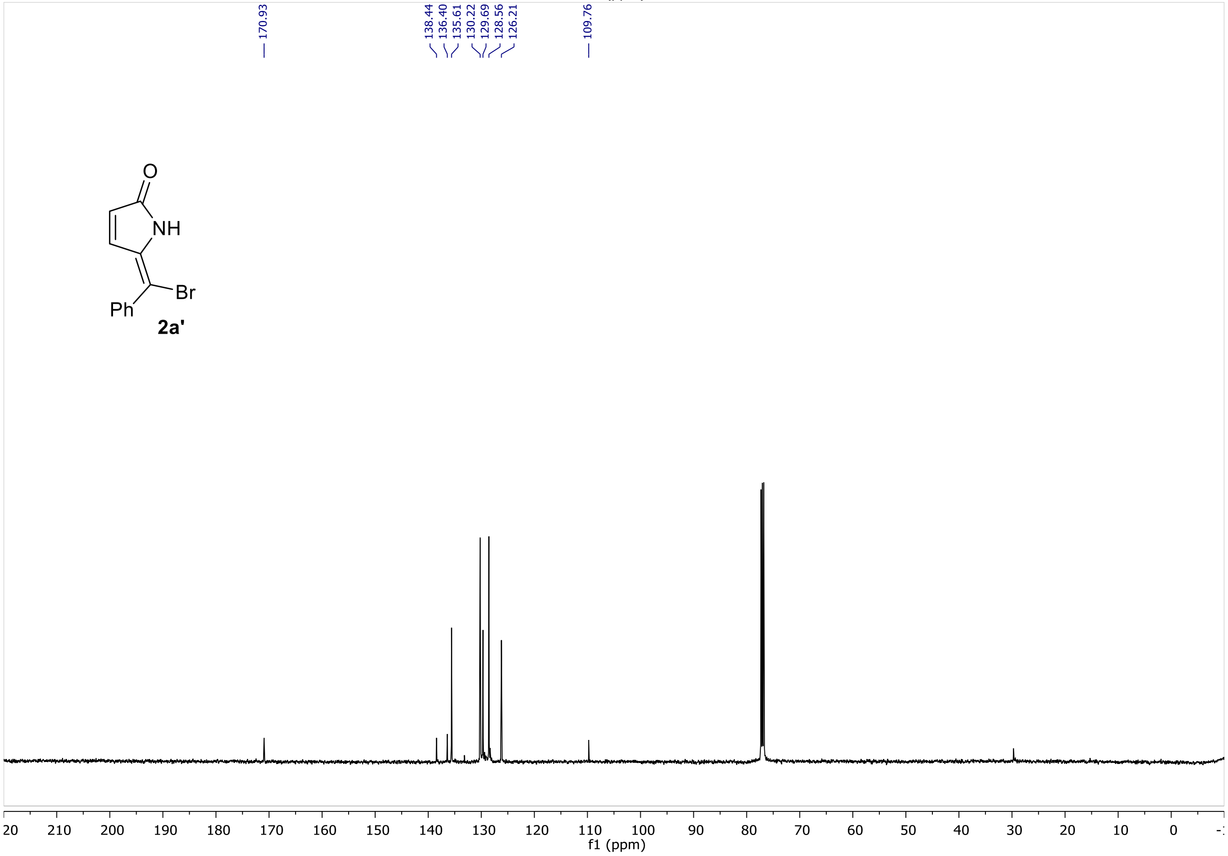
170.93
138.44
136.40
135.61
130.22
129.69
128.56
126.21
109.76
O
NH
Br
Ph
2a'
210
200
190
180
170
160
150
140
130
120
110
100
90
80
70
60
50
40
30
20
10
0
f1 (ppm)

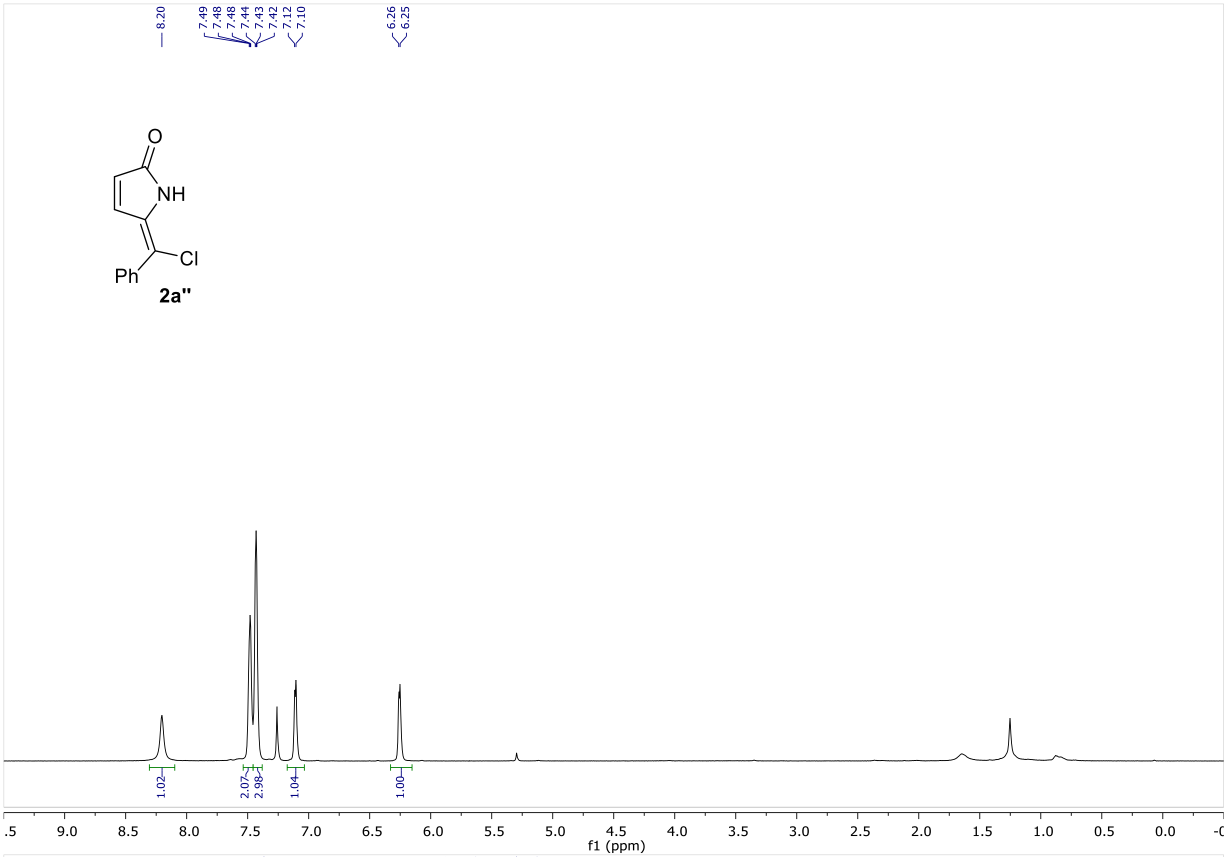
8.20
7.49
7.48
7.48
7.44
7.43
7.42
7.12
7.10
6.26
6.25
O
NH
Cl
Ph
2a''
1.02
2.07
2.98
1.04
1.00
f1 (ppm)

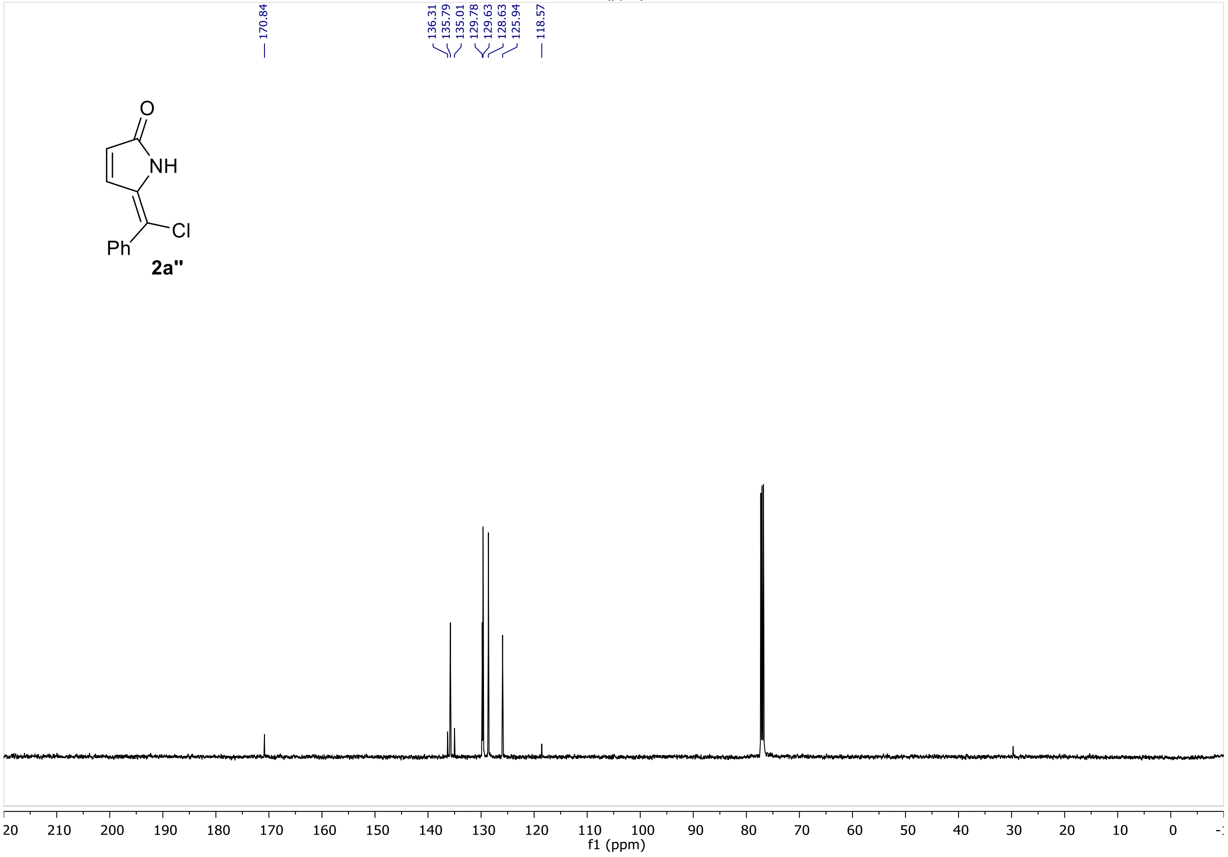
170.84
136.31
135.79
135.01
129.78
129.63
128.63
125.94
118.57
O
NH
Cl
Ph
2a''
f1 (ppm)

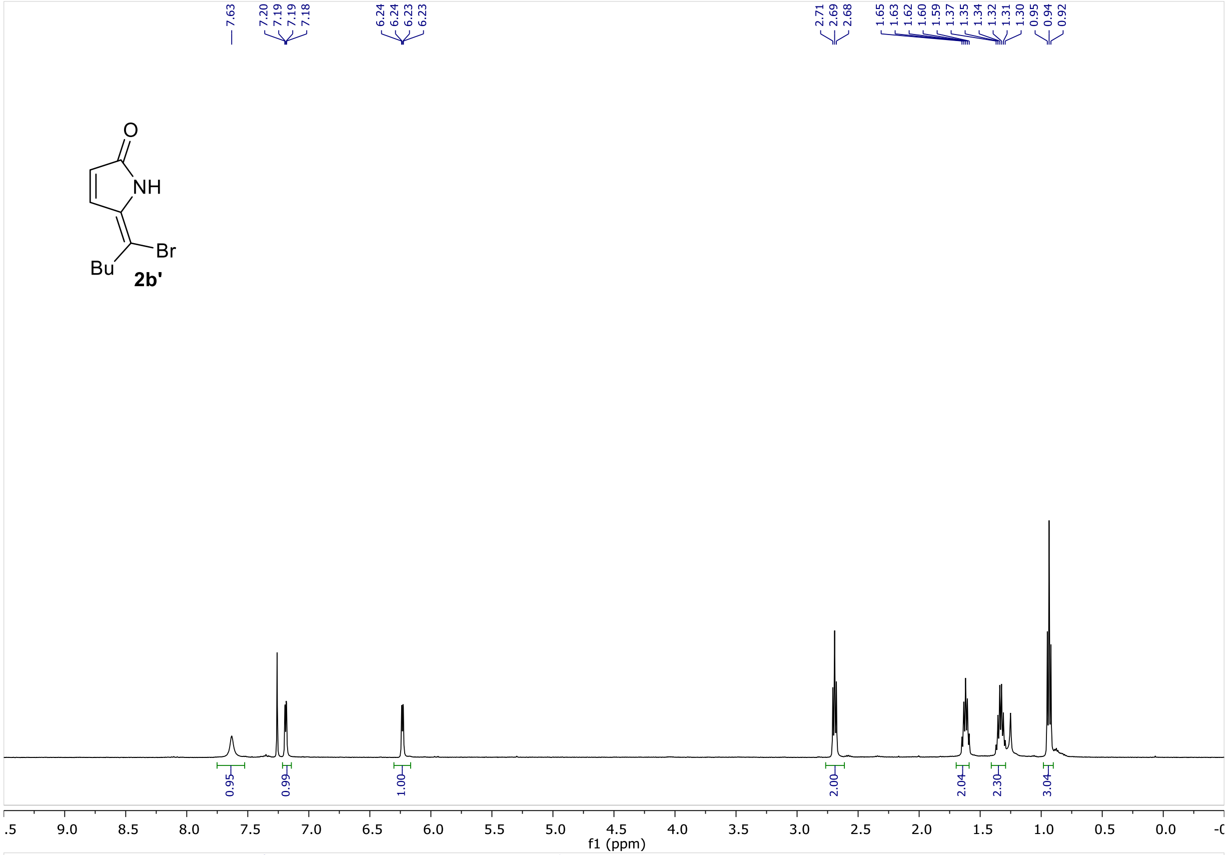
7.63
7.20
7.19
7.19
7.18
6.24
6.24
6.23
6.23
2.71
2.69
2.68
1.65
1.63
1.62
1.60
1.59
1.37
1.35
1.34
1.32
1.31
1.30
0.95
0.94
0.92
O
NH
Br
Bu
2b'
0.95
0.99
1.00
2.00
2.04
2.30
3.04
f1 (ppm)

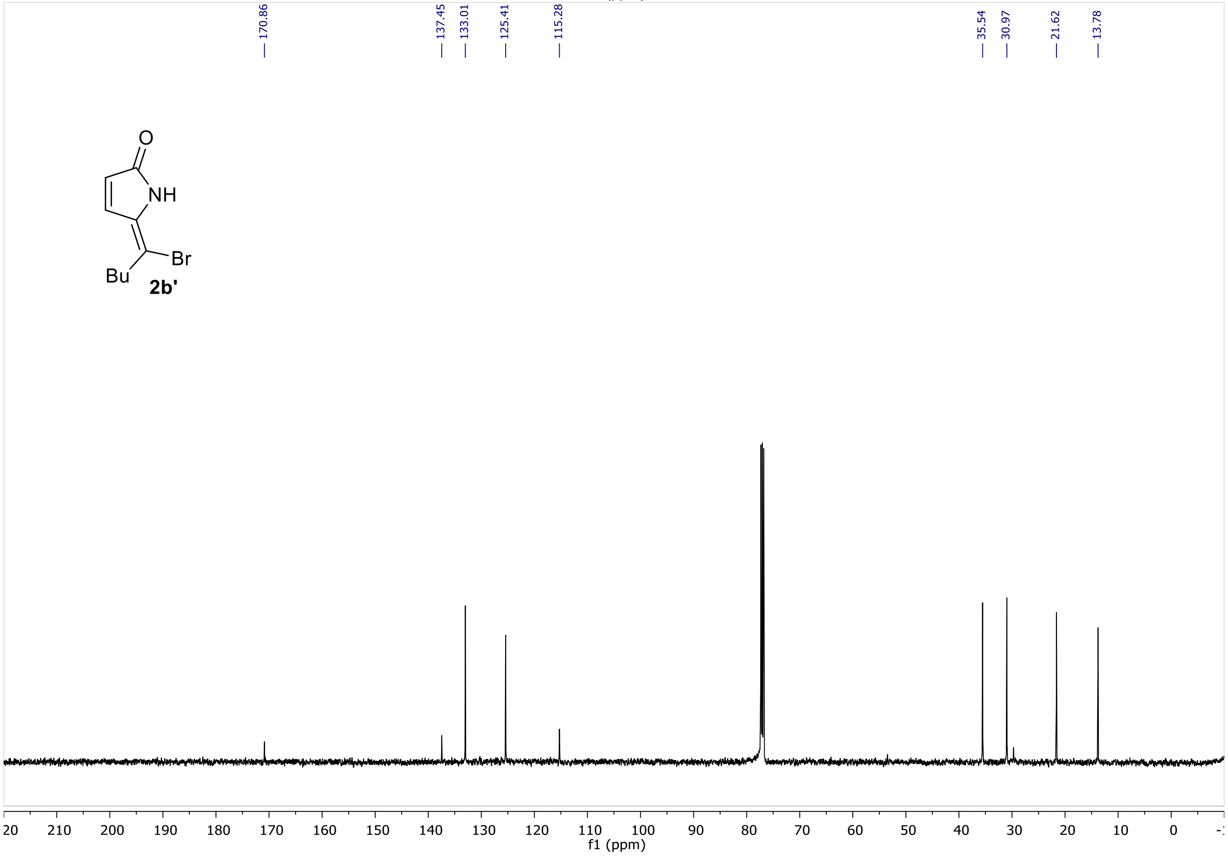
170.86
137.45
133.01
125.41
115.28
35.54
30.97
21.62
13.78
O
NH
Br
Bu
2b'
f1 (ppm)

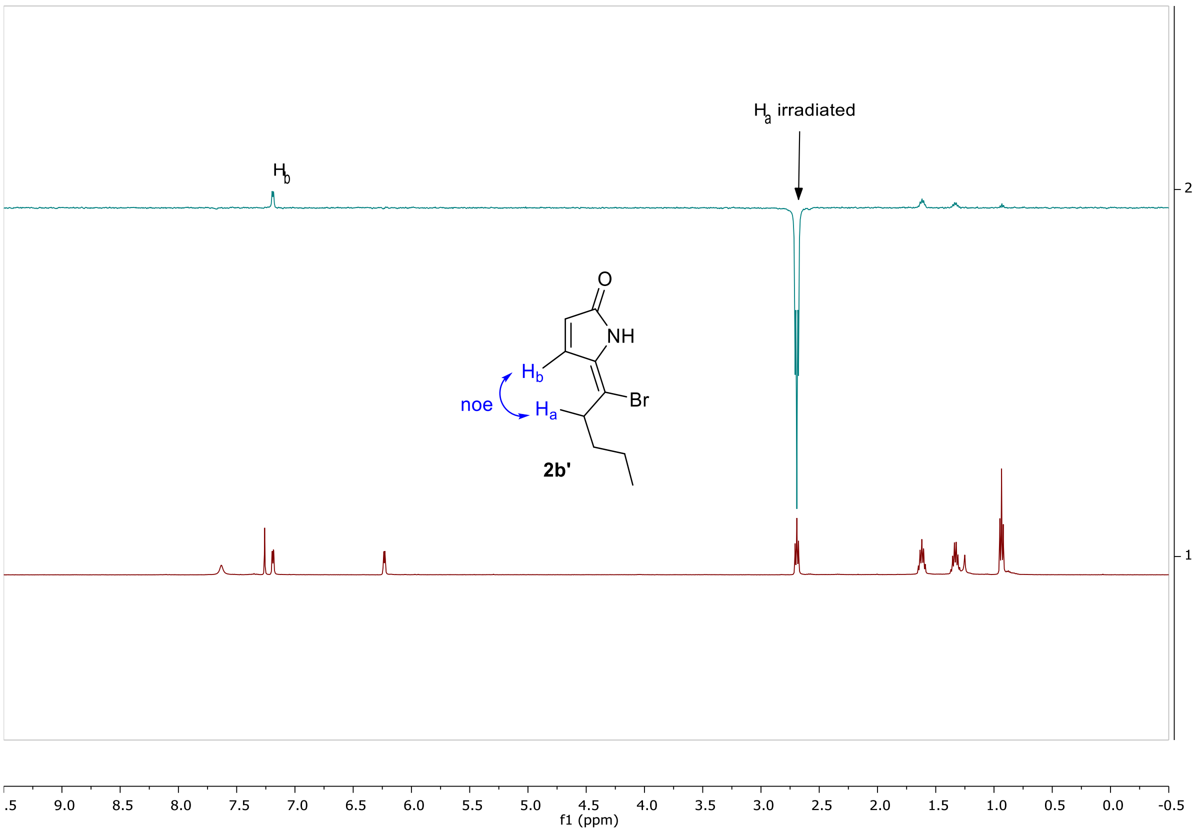

Hb
Ha irradiated
O
NH
Hb
noe
Ha
Br
2b'
2
1
.5
9.0
8.5
8.0
7.5
7.0
6.5
6.0
5.5
5.0
4.5
4.0
3.5
3.0
2.5
2.0
1.5
1.0
0.5
0.0
-0.5
f1 (ppm)

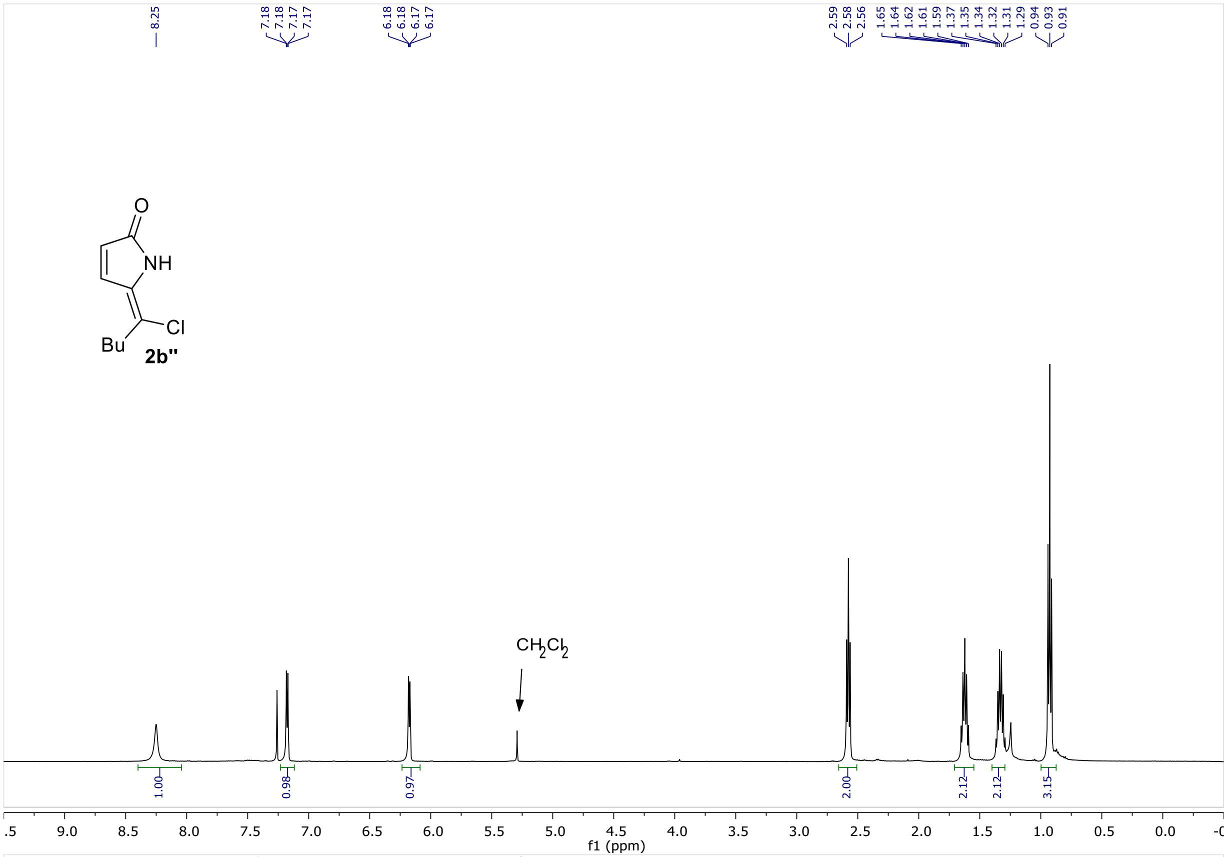
8.25
7.18
7.18
7.17
7.17
6.18
6.18
6.17
6.17
2.59
2.58
2.56
1.65
1.64
1.62
1.61
1.59
1.37
1.35
1.34
1.32
1.31
1.29
0.94
0.93
0.91
O
NH
Cl
Bu
2b''
CH2Cl2
1.00
0.98
0.97
2.00
2.12
2.12
3.15
f1 (ppm)

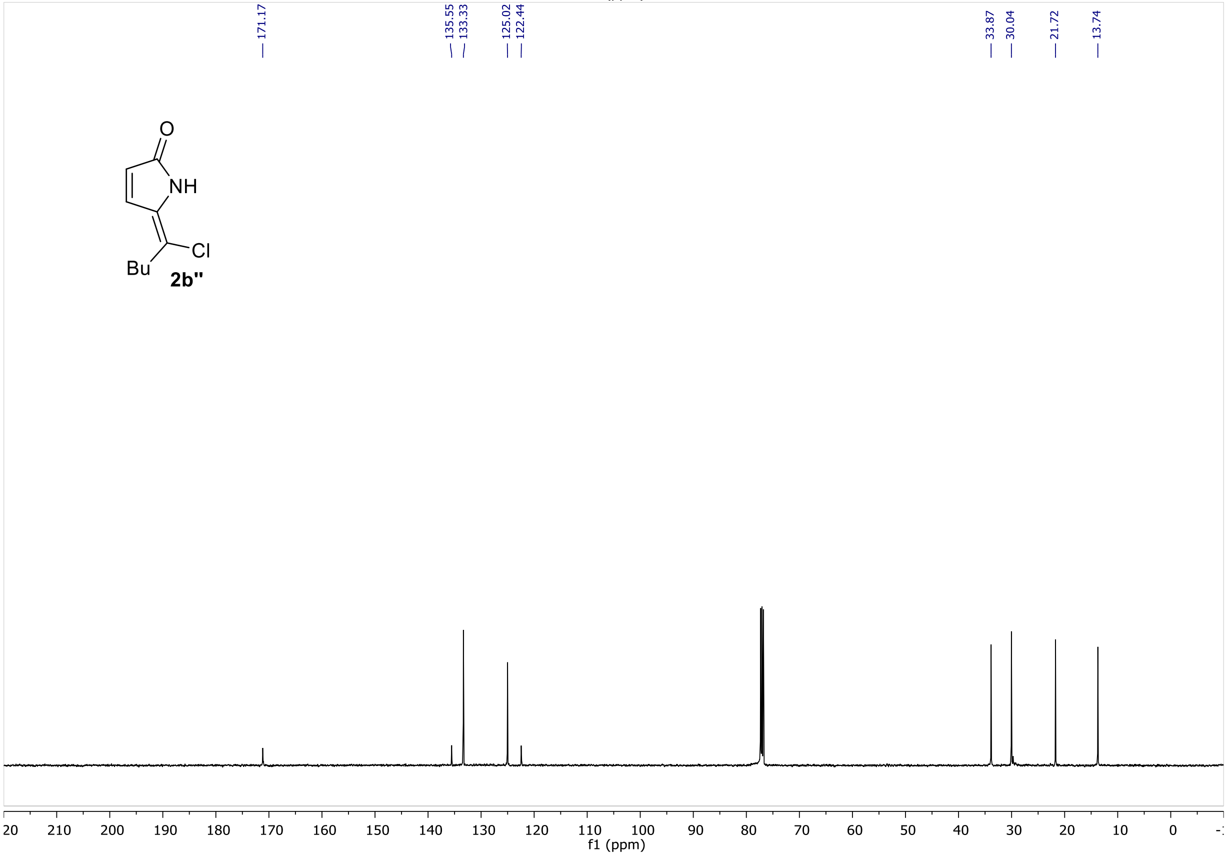
171.17
135.55
133.33
125.02
122.44
33.87
30.04
21.72
13.74
O
NH
Cl
Bu
2b''
f1 (ppm)

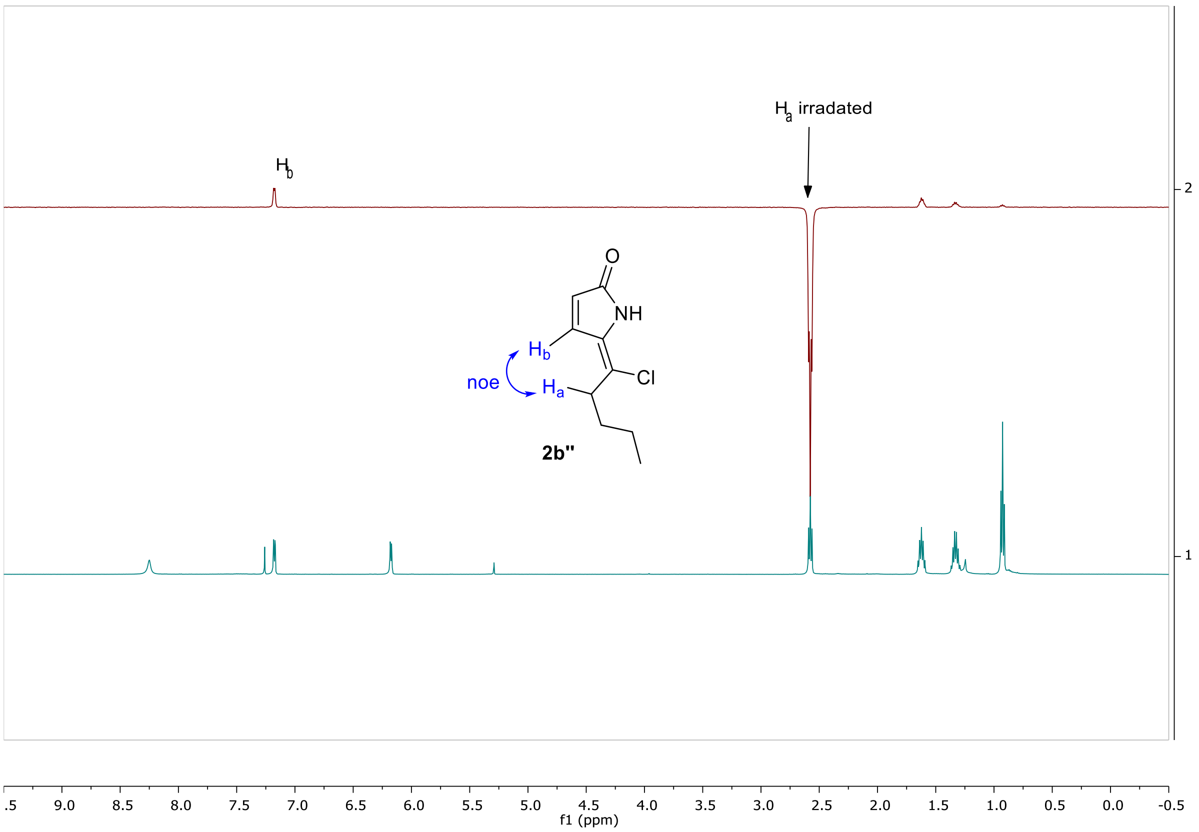
Hb
Ha irradated
O
NH
Hb
noe
Ha
Cl
2b''
2
1
.5 9.0 8.5 8.0 7.5 7.0 6.5 6.0 5.5 5.0 4.5 4.0 3.5 3.0 2.5 2.0 1.5 1.0 0.5 0.0 -0.5
f1 (ppm)

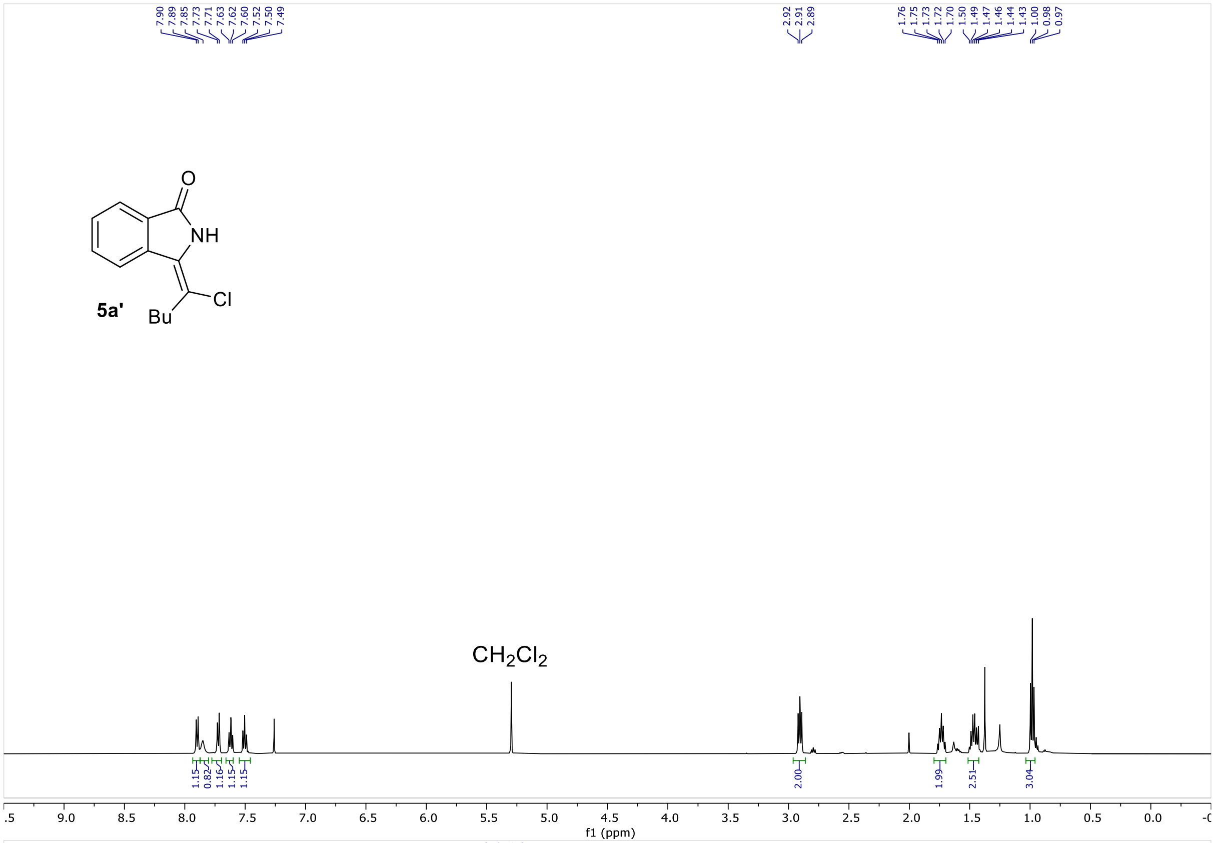
7.90
7.89
7.85
7.73
7.71
7.63
7.62
7.60
7.52
7.50
7.49
2.92
2.91
2.89
1.76
1.75
1.73
1.72
1.70
1.50
1.49
1.47
1.46
1.44
1.43
1.00
0.98
0.97
5a'
NH
O
Cl
Bu
CH2Cl2
1.15
0.82
1.16
1.15
1.15
2.00
1.99
2.51
3.04
f1 (ppm)

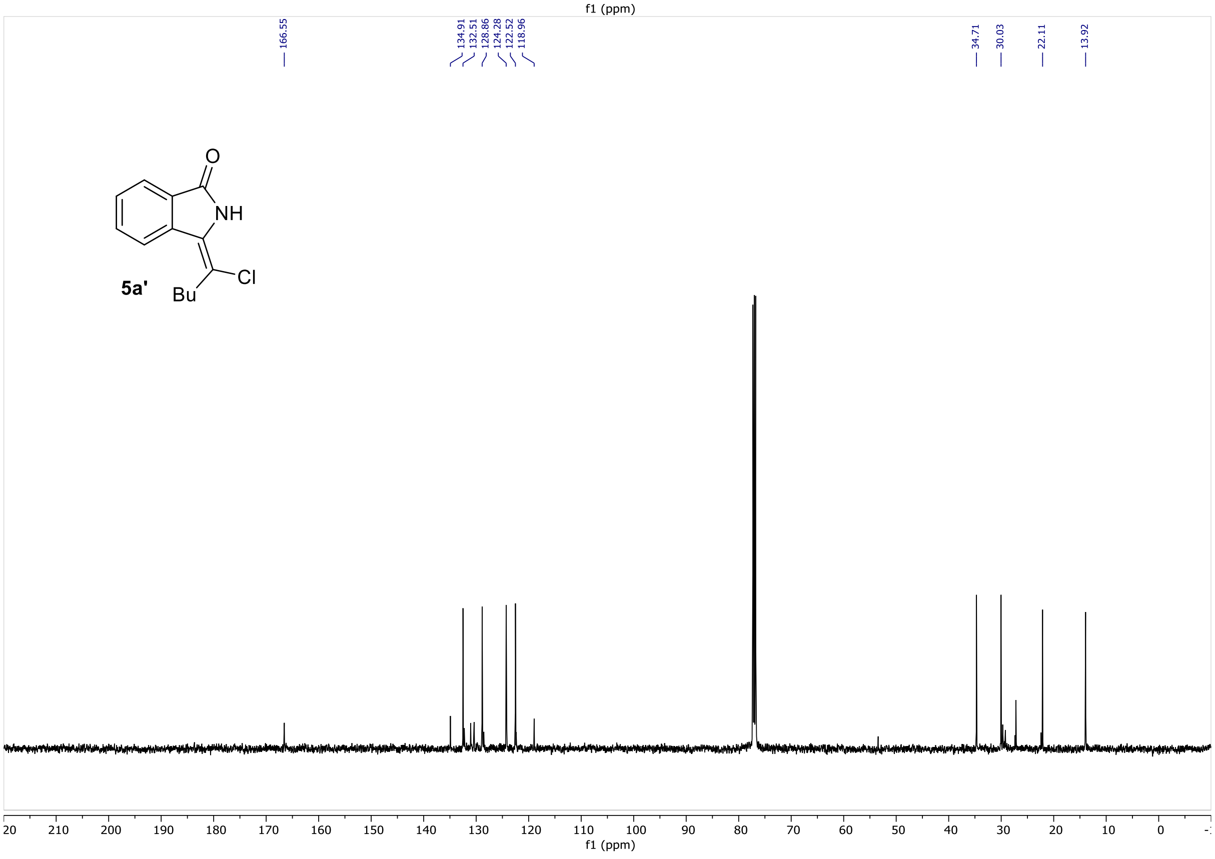
166.55
134.91
132.51
128.86
124.28
122.52
118.96
34.71
30.03
22.11
13.92
5a'
NH
O
Cl
Bu
f1 (ppm)

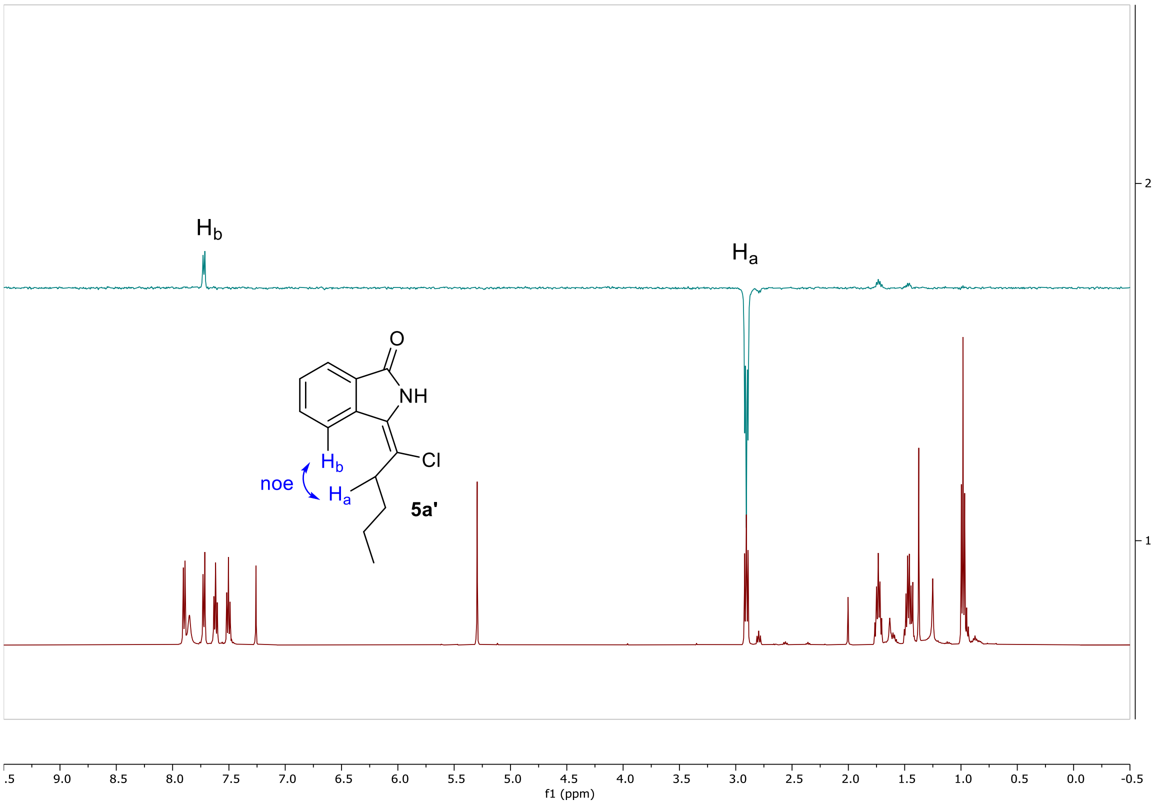

H$_b$
H$_a$
O
NH
Cl
H$_b$
noe
H$_a$
5a'
2
1
.5 9.0 8.5 8.0 7.5 7.0 6.5 6.0 5.5 5.0 4.5 4.0 3.5 3.0 2.5 2.0 1.5 1.0 0.5 0.0 -0.5
f1 (ppm)

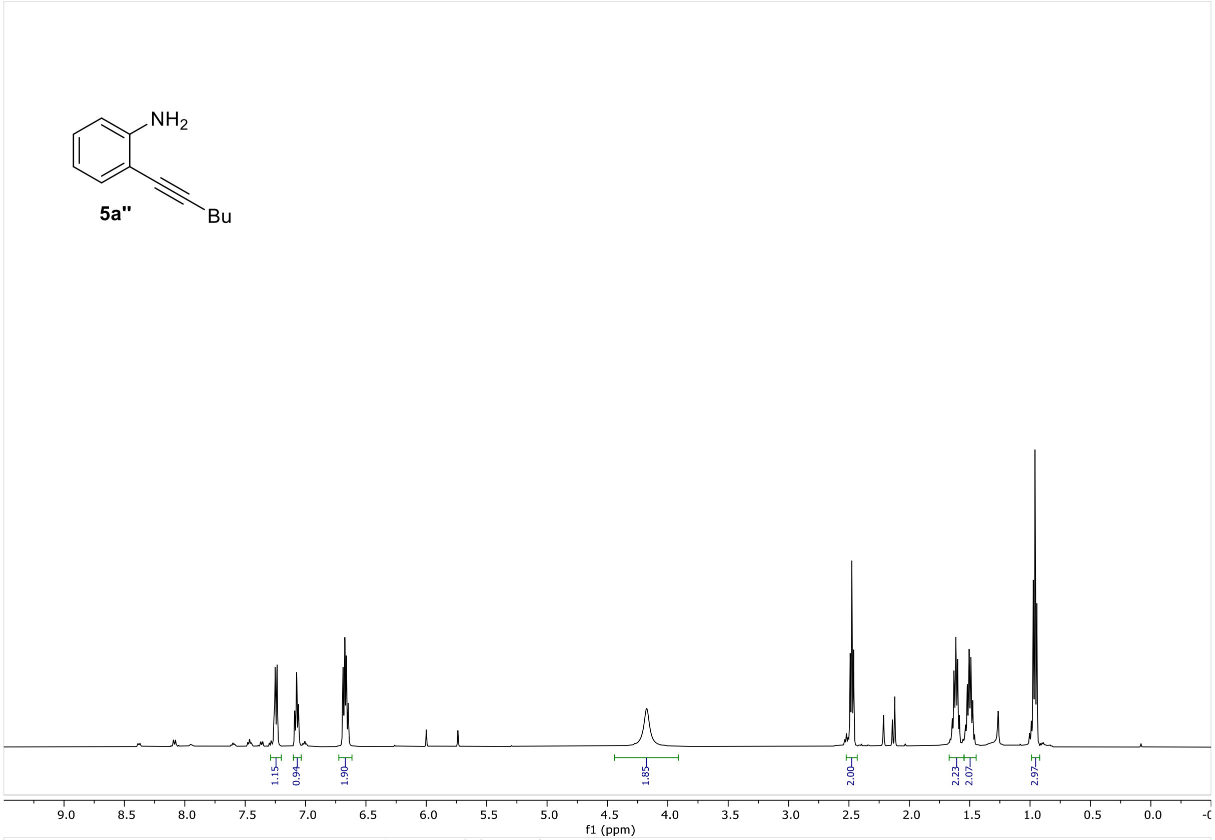
NH2
5a''
Bu
1.15
0.94
1.90
1.85
2.00
2.23
2.07
2.97
9.0 8.5 8.0 7.5 7.0 6.5 6.0 5.5 5.0 4.5 4.0 3.5 3.0 2.5 2.0 1.5 1.0 0.5 0.0
f1 (ppm)

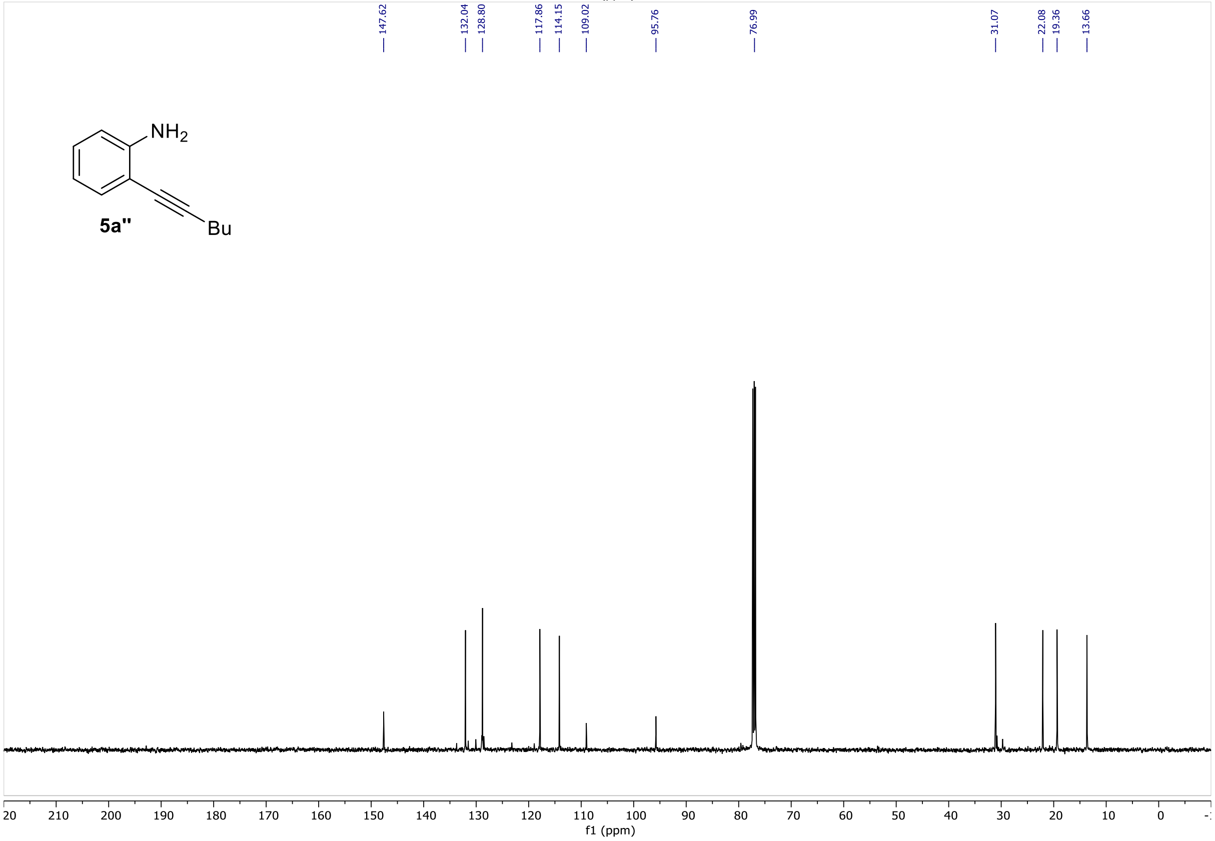
147.62
132.04
128.80
117.86
114.15
109.02
95.76
76.99
31.07
22.08
19.36
13.66
NH2
5a''
Bu
210 200 190 180 170 160 150 140 130 120 110 100 90 80 70 60 50 40 30 20 10 0
f1 (ppm)
